# Differential expression of miRNAs in the presence of B chromosome in the cichlid fish *Astatotilapia latifasciata*

**DOI:** 10.1186/s12864-021-07651-w

**Published:** 2021-05-12

**Authors:** Jordana Inácio Nascimento-Oliveira, Bruno Evaristo Almeida Fantinatti, Ivan Rodrigo Wolf, Adauto Lima Cardoso, Erica Ramos, Nathalie Rieder, Rogerio de Oliveira, Cesar Martins

**Affiliations:** 1 Department of Structural and Functional Biology, Institute of Bioscience at Botucatu, São Paulo State University (UNESP), Botucatu, SP 18618-689 Brazil; 2 Medical School, University of Ninth of July (UNINOVE), Bauru, SP Brazil; 3 Faculty of Mathematics and Natural Sciences, University of Bonn, Bonn, Germany; 4 Department of Biostatistics, Plant Biology, Parasitology and Zoology, Institute of Bioscience at Botucatu, São Paulo State University (UNESP), Botucatu, SP Brazil

**Keywords:** RNA-seq, Supernumerary chromosome, Selfish element, Genomic, Transcriptome, Small noncoding RNAs, Cichlid, Fish, Teleost

## Abstract

**Background:**

B chromosomes (Bs) are extra elements observed in diverse eukaryotes, including animals, plants and fungi. Although Bs were first identified a century ago and have been studied in hundreds of species, their biology is still enigmatic. Recent advances in omics and big data technologies are revolutionizing the B biology field. These advances allow analyses of DNA, RNA, proteins and the construction of interactive networks for understanding the B composition and behavior in the cell. Several genes have been detected on the B chromosomes, although the interaction of B sequences and the normal genome remains poorly understood.

**Results:**

We identified 727 miRNA precursors in the *A. latifasciata* genome, 66% which were novel predicted sequences that had not been identified before. We were able to report the *A. latifasciata*-specific miRNAs and common miRNAs identified in other fish species. For the samples carrying the B chromosome (B^+^), we identified 104 differentially expressed (DE) miRNAs that are down or upregulated compared to samples without B chromosome (B^−^) (*p* < 0.05). These miRNAs share common targets in the brain, muscle and gonads. These targets were used to construct a protein-protein-miRNA network showing the high interaction between the targets of differentially expressed miRNAs in the B^+^ chromosome samples. Among the DE-miRNA targets there are protein-coding genes reported for the B chromosome that are present in the protein-protein-miRNA network. Additionally, Gene Ontology (GO) terms related to nuclear matrix organization and response to stimulus are exclusive to DE miRNA targets of B^+^ samples.

**Conclusions:**

This study is the first to report the connection of B chromosomes and miRNAs in a vertebrate species. We observed that the B chromosome impacts the miRNAs expression in several tissues and these miRNAs target several mRNAs involved with important biological processes.

**Supplementary Information:**

The online version contains supplementary material available at 10.1186/s12864-021-07651-w.

## Background

B chromosomes (Bs) are extra and nonessential elements found in approximately 10 to 15% of karyotyped organisms, ranging from fungi to plants and animals, and do not follow classical Mendelian inheritance patterns [1–5]. The origin, evolution, genome content and morphology of B chromosomes vary among organisms [6].

A proto-B chromosome can emerge from chromosomal rearrangements, partial duplication of A chromosomes (normal chromosomes of the karyotype) or nonmeiotic disjunction [5]. This new element increases its genomic content by insertion of A chromosome sequences copies, including various repetitive DNA classes [7–9], protein-coding genes [2, 10–13], pseudogenes [14], retrogenes [15], organellar DNA sequences [16] and noncoding sequences [17–20]. Duplicated sequences in B chromosomes have been proposed to facilitate its permanence in the host genome. These sequences may help the B chromosome drive during gametogenesis avoiding B elimination [5, 11]. In this way, the characterization of the B genomic content and its effects using genomics and bioinformatics tools is a promising approach to understand this extra element and its relation to the host genome [21, 22].

Regarding to noncoding RNAs, some few sequences have already been reported either in the B chromosome or impacting in the expression of autosomal sequences in the B^+^ samples. Noncoding RNAs exert strong effects on cell biological processes and are potentially related to the presence of the B chromosome [18, 19, 23]. Among noncoding RNAs, microRNAs (miRNAs) (~ 22 nucleotides long) act in the translation control by promoting the degradation or cleavage of mRNAs. That is why they are responsible for the control of important processes, such as development and differentiation, cell cycle regulation, stress and aging, and some diseases such as cancer. Notably, miRNAs are one of the most abundant regulators in the genome and several of them are highly conserved among organisms [24–26].

The biogenesis of miRNAs starts in the nucleus with the transcription of the primary miRNA (pri-miRNA) that has a hairpin structure and is processed to form a RNA duplex, named pre-miRNA. The pre-miRNA is transported to the cytoplasm and processed by the Dicer, that cleaves the RNA duplex into two single RNA molecules, 5p and 3p arms. Only one arm will become the mature sequence while the another one will be degraded [27]. When associated with the Argonaute protein, the mature miRNA interacts with its target based on antisense Watson-Crick pairing that occurs mainly in the 3′ untranslated regions (3’UTR) of mRNAs [28].

The connection between B chromosome and miRNAs has only been investigated in two organisms, an invertebrate and a plant species. The wasp *Nasonia vitripennis* carries a selfish supernumerary chromosome called PSR (Paternal Sex Ratio) that transcribes several small RNAs sequences, such as microRNAs, small interference RNAs and PIWI-Interacting RNAs [18]. In maize, B-derived miRNAs were found to affect A chromosome miRNA expression [23]. However, to the best of our knowledge, these reports are the only two describing small noncoding RNA sequences in the context of B chromosomes. Thus, the impact of B chromosome duplications in the small noncoding RNAs expression is poorly understood.

Among vertebrates, B chromosomes have already been described in approximately 100 fish species [4], corresponding to 16.28% of karyotyped species [29]. Teleost fishes are important for evolutionary studies, especially in the Cichlidae family, due to their rapid adaptive radiation in East African great lakes [30–32]. B chromosomes were detected in several cichlid species [30, 33, 34]. Among them, the African cichlid *Astatotilapia latifasciata*, which carries one or two B chromosomes in both sexes, has been extensively investigated through classical cytogenetics [33, 35], molecular biology [9, 36] and, more recently, genomic approaches [11, 15, 19, 37]. Repetitive elements [9], coding genes [11] and a long noncoding RNA [19] have already been identified in the B chromosome of *A. latifasciata.* Some of these sequences revealed a differential expression in the B^+^ samples suggesting transcription activity and involvement of this extra element into several biological pathways [19, 20, 38].

The *A. latifasciata* B chromosome content has been investigated by comparing sequencing from B^−^ and B^+^ DNA and RNA samples. The B chromosome gene content was first reported through genomic coverage rate analysis based on Illumina high coverage sequencing and 454 Life Sciences sequencing of a microdissected B chromosome [11]. In this work, the microdissected B chromosome sequences were compared to *Metriaclima zebra* reference genome, and the first B-genes of *A. latifasciata* were reported. Later, the *A. latifasciata* draft genome was constructed using Illumina high scale data and identified several duplicated contigs in the B chromosome [37]. The coverage ratio compares coverage of sequenced among B^−^ and B^+^ samples aligned against a reference assembled genome, which allows to identify higher coverage regions on the B^+^ sequencing dataset, that represent duplicated regions in the B chromosome [11, 39].

In this study, large-scale small RNA sequences (sRNAseq) were generated from the brain, muscle and gonads of B^−^ and B^+^ individuals of both sexes of *A. latifasciata*. Using bioinformatics approaches, the *A. latifasciata* miRNA profile was described, and compared with other teleost miRNAs, mainly cichlid species. This allowed the identification of conserved and specific novel miRNAs. In this work, we introduce the application of several bioinformatics tools to investigate miRNA sequences in the context of B chromosomes based on coverage ratio analysis and the generated “B-blocks” as previously reported [11]. B-blocks are putative genomic regions observed on B chromosomes and detected via coverage ratio analysis as a result of NGS read coverage comparison between the two sequenced genomic datasets (B^+^ and B^−^). We described 104 miRNAs that were differentially expressed (DE), either up or downregulated in the presence of the B chromosome compared to samples without B chromosome (the control). These miRNAs have common mRNA targets in the brain, muscle and gonads. Additionally, we found protein coding genes already described in the B chromosome (the B genes) as targets of DE miRNAs. Moreover, a network based on human protein-protein interactions of the DE miRNAs targets highlights the great potential of DE miRNAs in the influence of B chromosomes over several biological processes.

By combining the sRNAseq with the availability of *A. latifasciata* genomic and mRNA transcriptomic data we described the miRNome of this cichlid species. Further, this is the first study that relates the miRNA expression and the B chromosome presence in a vertebrate species. This is also the first report of coding and noncoding interactions related to B chromosome presence.

## Results

### The *A. latifasciata* miRNome

A nonredundant dataset was constructed based on miRBase fish miRNAs to create a miRNA reference list (see the Materials and Methods). The procedure resulted in 1456 precursors and 1234 mature sequences. This fish miRNA reference list was used to identify the miRNAs in the sRNA-seq data and annotate them in the *A. latifasciata* genome. We identified 727 miRNA precursors (pre-miRNAs) throughout the *A. latifasciata* genome (see Additional File 1). Among them, 246 (33.84%) pre-miRNAs have similarity with described miRNAs in miRBase, and they are called known miRNAs. On the other hand, sequences that were not similar to existing miRNAs are called novel. Additionally, novel miRNAs could only present seed similarity to existing miRNAs, indicating new miRNAs probably belonging to an existent miRNA family [40]. The novel *A. latifasciata* pre-miRNAs represent 481 (66.16%) precursor sequences, and 29.31% of them exhibit miRBase seed similarity (nucleotides 2–8 from the 5′ end of the mature miRNA).

**Fig. 1:**
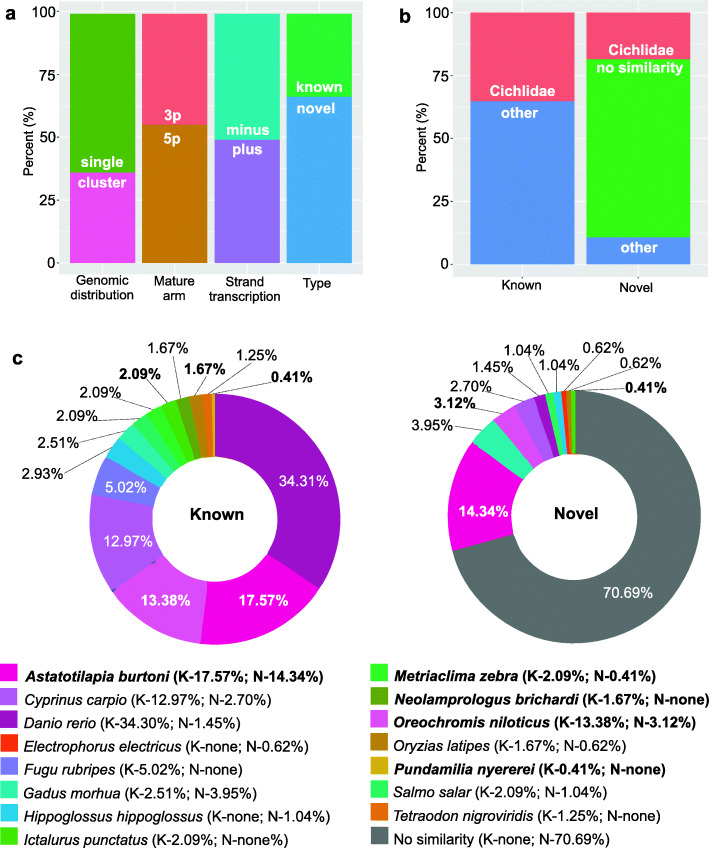
Description of the *A. latifasciata* miRNome. **a** Percentage of miRNA characteristics. Genomic distribution: the miRNA precursor arrangement on the genome; Mature arm: the mature sequence with more reads on RNA-Seq; Strand transcription: precursor transcription strand; Type: if the miRNA is similar to another known miRNA from miRBase (known) or if is probably a new miRNA sequence (novel). **b** Percentage of similarity with cichlids and other fishes. **c** Percentage of miRNA similarity compared with fish sequences from miRBase. Cichlid species are highlighted in bold. K, Known miRNAs; N, novel miRNAs

Clustered miRNAs can be arranged in a 5 kilobase genomic region long and generally are related to the same transcription factors [39]. Here, we considered clustered miRNAs when the sequences were found in the same genomic contig and not exceeded 5 kilobases distance. The clustered miRNAs (pre-miRNAs on the same genome contig) accounted for 232 (31.91%) sequences; the longest cluster contained 9 pre-miRNAs. Also, 495 (36.31%) precursor sequences are single miRNAs in a genomic contig. Usually, one arm (5p or 3p) is highly expressed in the cell, while the other arm can be degraded [27]. Thus, comparing the expression of the arms in the sRNAseq, 55% of pre-miRNAs displayed higher expression in the 5p arm mature sequence. Transcription was identified on the minus strand for 368 (50.62%) pre-miRNAs and on the plus strand for 359 (49.38%) pre-miRNAs. All the results mentioned above are described in the Fig. 1a and b.

The last miRBase release added seven new fish species and 2050 new sequences, summing up 3687 miRNAs sequences (Additional File 11 – Table S3). From these new species in the last release, five are cichlids and contribute to 1300 miRNAs sequences. The miRNA seed sequences from *A. latifasciata* have similarity with 15 fish species (representatives of nine teleost families) in miRBase, corresponding to 246 pre-miRNAs (Additional File 1). The species were verified according to the three first letters of the miRNA ID, which corresponds to the species ID in the animal miRNA nomenclature pattern, as indicated next in the species name. Thirty-five percent of known miRNA seed sequences share similarity with cichlids (*Astatotilapia burtoni* – abu, *Metriaclima zebra* – mze, *Neolamprologus brichardi* - nbr, *Oreochromis niloticus* – oni, and *Pundamilia nyererei* – pny); 65% show similarity with other teleost families (one species of Adrianichthyidae, *Oryzias latipes* – ola; two species of the Cyprinidae family, *Cyprinus carpio* – ccr, and *Danio rerio*, − dre; one species of Gadidae, *Gadus morhua* – gmo; one species of Ictaluridae, *Ictalurus punctatus* – ipu; one species of Pleuronectidae, *Hippoglossus hippoglossus* – hhi; one species of Salmonidae, *Salmo salar* – ssa; and two species of Tetraodontidae, *Fugu rubripes* – fru, and *Tetraodon nigroviridis* – tni) (Fig. 1b and c)*.* Even not being the most representative reference species *Danio rerio* seeds are well represented among the known miRNAs, probably indicating the presence of highly conserved miRNAs in the *A. latifasciata* miRNome (Additional File 9 – Table S3).

Approximately 70% of novel miRNAs are not similar to any seed from miRBase, indicating their potential as either new specific or nondescribed miRNAs (Fig. 1b and c). When considering only the novel miRNAs that present seed similarity from miRBase, 60% are similar to seed sequences from *A. burtoni, M. zebra* and *O. niloticus*, which probably represent exclusive conserved miRNAs families among cichlids (absent or not conserved in other groups).

### Searching for miRNA genes on the B chromosome

We performed three different strategies in order to investigate the miRNA presence in the B chromosome. All strategies were based on DNA and sRNAseq comparison of B^−^ and B^+^ samples (see Material and Methods).

**Fig. 2:**
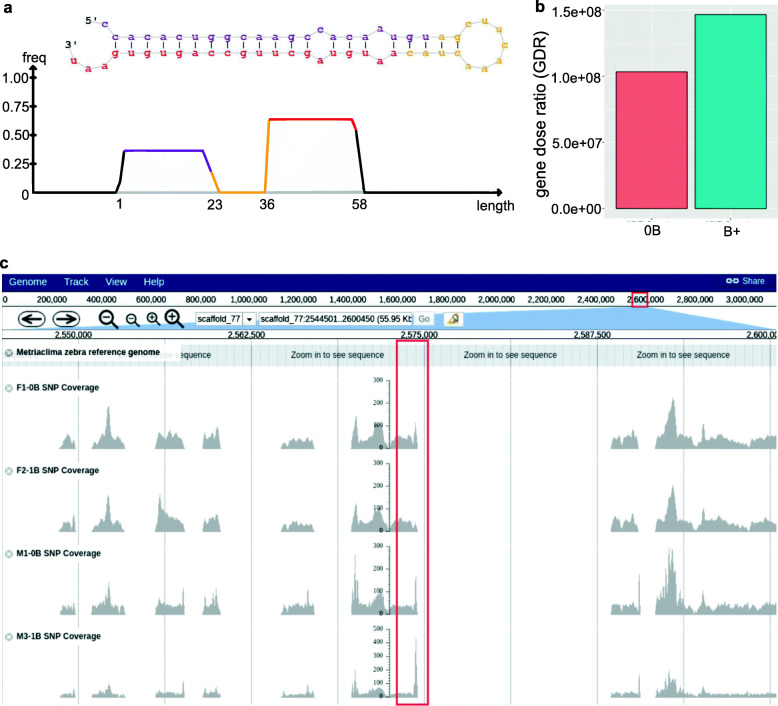
Functional miRNA absence in the B^+^ genome assembly. **a** The novel_2026-B^+^ predicted from the B^+^ assembly. This miRNA has a stem-loop secondary structure. **b** The NODE_313069 qPCR for B^−^ and B^+^ DNA samples. The difference between B^−^ and B^+^ amplification was not significant (*p*-value 13 > 0.05). **c** Predicted novel_2026-B^+^ match with *M. zebra* scafold_77. The *A. latifasciada* genomic sequencing reads are shown below the *M. zebra* reference genome, F1-0B SNP coverage (female B^−^ sample), F-1B SNP coverage (female B^+^ sample), M1-0B SNP coverage (male B^−^ sample), M3-1B SNP coverage (male B^+^ sample). The gray area is the read coverage, the blank spaces show no aligned reads in this region, and the red rectangle highlights the novel_2026-B^+^ NODE_313069 region matching the *M. zebra* assembly

The coverage ratio analysis (first strategy) consists in screening the coverage difference between the B^−^ and B^+^ genomic reads aligned against the *A. latifasciata* assembled genome. This strategy allows us to find segments of A chromosomes that are duplicated on the B chromosome. We did not detect any miRNA gene inside a genomic region with coverage corresponding to the B chromosome (B^+^ blocks). The second method was based on alignments using the sRNAseq reads from B^−^ and B^+^ samples of all tissues that failed to align in the *A. latifasciata* reference genome. Then, we performed a second alignment using these unaligned sRNAseq reads to B^+^ assembled genome (the *A. latifasciata* DNA with B chromosome). This method identified 21 novel miRNA genes, of which 6 were exclusively expressed in B^+^ samples (Additional File 2 and Additional File 3). Thus, ten B^+^ assembly miRNA genes were selected for validation, as they had an adequate length for qPCR primer construction. Only one miRNA gene (called here novel_2026-B^+^, Fig. 2a, Additional File 3) located in contig NODE_313069 from the B^+^ assembly was PCR-amplified (Additional File 4). However, amplification was observed in both B^−^ and B^+^ samples (data not shown). qPCR experiments were performed to confirm that this genomic segment was in both groups of individuals (B^−^ and B^+^). The gene dose ratio (GDR) compares the relative gene copies trough qPCR, which showed that the novel_2026-B^+^ region has not GDR difference in the B^−^ and B^+^ genomes, meaning the same number of copies in both genomes (Fig. 2b). Additionally, novel_2026-B^+^ is similar to scaffold_77 of *M. zebra*, where B^−^ and B^+^ genomic reads are aligned (Fig. 2c). The *A. latifasciata* B^−^ and B^+^ genomes have several assembly gaps that might justify the absence of miRNA alignments. The evidence suggests that novel_2026-B^+^ occurs in the *A. latifasciata* genome but is not present in the B chromosome. The gaps are probably resulting of an assembly bias in the *A. latifasciata* genome, where the short Illumina reads caused the region to be ignored during the assembly (Additional File 4 – Figure S1).

The third approach was predicting miRNAs using the miRBase reference and the sRNAseq, as the previous strategies, but set the genomic “B-blocks” filtered by Jehangir et al. [37] as the reference background. With this strategy we would like to confirm if no duplicated miRNAs were missed in our manual coverage ratio strategy (first strategy). This prediction found 33 pre-miRNAs on the B-blocks. Two miRNAs are similar to mir-2188, and the others are novel (no miRBase similarity) (Additional File 6). These 33 miRNAs did not show interaction with any mRNAs and were not considered for further analysis.

Therefore, based on the results obtained with these three strategies, we did not find strong evidences of miRNAs in the B chromosome. We discuss the limitations further.

### Effects of B chromosomes on miRNA expression

**Fig. 3:**
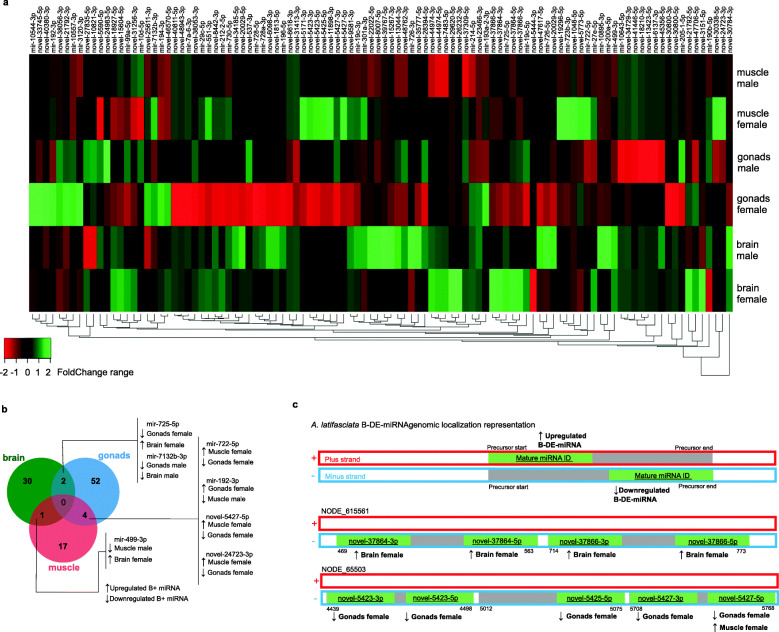
Differential expression analysis. **a** Nonredundant differentially expressed miRNAs in B^+^ samples (green represents upregulated miRNAs and red represents downregulated, considering *p* < 0.05 to > 1.5 fold change). **b** Venn diagram of DE miRNAs among tissues. **c** Representation of two clustered DE-B-miRNAs structural organization in genomic contigs

The differential expression analysis was performed by comparing B^+^ samples against the samples without B chromosome (as the control) in each tissue (brain, gonads and muscle) and each sex (male and female). Several miRNAs were differentially expressed (DE) in B^+^ samples (either up or downregulated), which is why these sequences were called B DE miRNAs (B-DE-miRNAs). The heatmap in the Fig. 3a shows the upregulated sequences (positive FoldChange in green gradient) and downregulated sequences (negative FoldChange in red gradient) in B^+^ samples in each sex and tissue. The profile of DE miRNAs between tissues, sexes and the presence of the B chromosome detected 104 nonredundant miRNAs (Fig. 3a and b). In brain, 12 novel and 2 known B-DE-miRNAs were found in females and 12 novel and 7 known B-DE-miRNAs were detected in males. In gonads, 29 novel and 18 known B-DE-miRNAs were found in females and 8 novel and 3 known B-DE-miRNAs were detected in males. In muscle, 6 novel and 4 known B-DE-miRNAs were found in females and 8 novel and 4 known B-DE-miRNAs were detected in males.

Five genomic regions carry B-DE-miRNAs, forming clustered miRNAs (Fig. 3c and Table 1). The B-DE-miRNAs of the same cluster displayed the same DE pattern in a particular tissue. However, a cluster can have different expression profiles among tissues. Thus, a unique pattern for all tissues was not identified. Considering the genomic contigs, the contig NODE_615561 contained 4 novel mature B-DE-miRNAs that were upregulated in the brains of females. These miRNAs belong to 2 novel pre-miRNAs (novel-37864 and novel-37866) that are similar to mir-27c and mir-23c from *A. burtoni* seeds. In contig NODE_65503, 3 novel miRNA precursors (novel-5423, novel-5425 and novel 37866) with 5 downregulated B-DE-miRNAs were detected in the gonads of females, which are similar to the mir-217 seeds from *C. carpio* and the mir-216a and mir-216b seeds from *A. burtoni.* However, the cluster on contig NODE_65503 also contained a mature B-DE-miRNA that was upregulated in the muscle of females; therefore, a cluster shows DE profiles according to the tissue (Fig. 3c and Table 1). The distance between the pre-miRNAs genes is described in the start and end of pre-miRNAs sequences on Table 1. Although each tissue has a different miRNA expression profile, no difference in *drosha* and *dicer* gene expression was observed in B^+^ samples (Additional File 5).

**Table 1:** Clustered miRNAs composed by downregulated (↓) and upregulated (↑) B-DE-miRNAs in brain (BR), gonad (G), muscle (MU), female (F), male (M). FG, FoldChange

Contig	Pre-miRNA ID	DNA strand	Pre-miRNA start-end	Seed similarity	Mature B-DE-miRNA	Expression in B^+^ samples	FC in B^+^ sampes
NODE_173406	mir-99a	–	9625–9682	nbr-mir-99a	mir-99a-5p	↓ GO_F	−1.23
	novel_13044	+	9626–9684	gmo-mir-100b-5p	novel_13044-3p	↑ BR_M	+ 1.38
NODE_615561	novel_37864	–	499–563	abu-mir-27c	novel_37864-5p	↑ BR_F	+ 2.78
					novel_37864-3p	↑ BR_F	+ 2.49
	novel_37866	–	714–773	abu-mir-23c	novel_37864-5p	↑ BR_F	+ 2.68
					novel_37864-3p	↑ BR_F	+ 2.51
NODE_65503	novel_5423	–	4439–4498	ccr-mir-217	novel_5423-5p	↓ GO_F	−2.16
					novel_5423-3p	↓ GO_F	−1.76
	novel_5425	–	5012–5075	ccr-mir-216a	novel_5425-5p	↓ GO_F	−1.11
	novel_5427	–	5708–5768	ccr-mir-216b	novel_5427-3p	↓ GO_F	−2.19
					novel_5427-5p	↓ GO_F	−1.70
						↑ MU_F	+ 2.50
NODE_843581	mir-194	–	58381–58436	ccr-mir-194	mir-194-3p	↓ MU_M	−2.33
	mir-192	–	58185–58246	ccr-mir-192	mir-192-3p	↓ MU_M	−2.67
NODE_91705	mir-212-2	–	2623–2690	dre-mir-212-2	mir-212-2-5p	↓ GO_F	−1.89
	novel_7483	–	4415–4473	gmo-mir-2184	novel_7483-5p	↑ BR_F	+ 1.27

### The miRNA 3’UTR interaction and protein-protein interaction (PPI) network

The 3’UTR miRNA binding site was chosen to predict the *A. latifasciata* miRNA targets to restrict and avoid false positives results [41]. Other software were tested to miRNA:mRNA prediction, but they showed a huge number of interactions, being a problem to the filtering. The miRNA:mRNA interactions were predicted (Additional File 7) based on the miRNAs and 3’UTRs from the *A. latifasciata* transcriptome. Based on the best scores (< − 0.2, following the software developer recommendations [42], 2,080,942 interactions were identified in the brain, 2,061,604 in gonads and 2,016,807 in muscle.

**Fig. 4:**
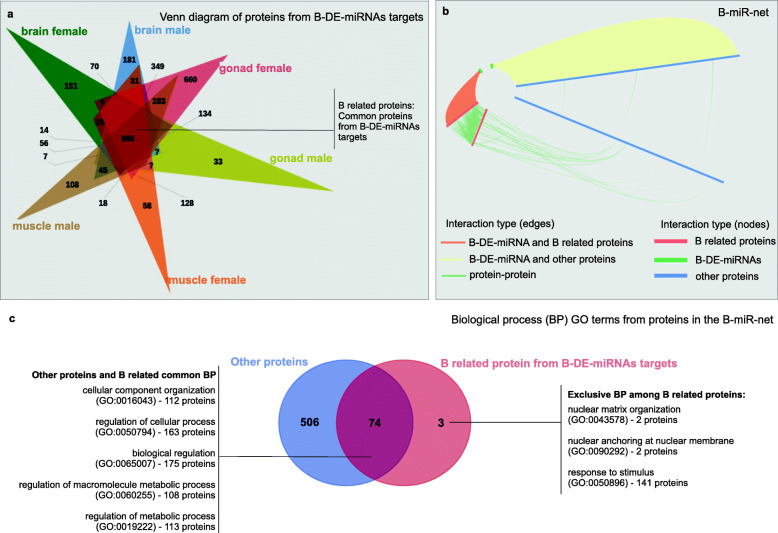
The B-mir-net. **a** Venn diagram of common protein annotation from B-DE-miRNAs targets. **b** The network axis represents the nodes ordered by degree (from low to high beginning from the center), and edges represent the connection between the axis nodes. Axes are duplicated to show the interconnections between its own subjects: red axis, B-related proteins (miRNA targets) and B genes reported in previous study [11]; green axis, mature miRNAs; blue axis, tissue-specific miRNA targets; orange edges, miRNAs interacting with proteins commonly regulated by miRNAs in all analyzed tissues; yellow edges, miRNAs interacting with tissue-specific proteins; green edges, protein-protein interactions. **c** Venn diagram of GO terms on B-related proteins and all proteins from predicted *A. latifasciata* targets

The miRNA:3’UTR interactions detected for the B-DE-miRNAs formed a list composed of B-DE-miRNAs and targets (mRNAs). The protein annotations of these transcripts (Additional File 8) from each tissue are presented in a Venn diagram, and 960 proteins were shared among all compared groups (defined as B-related proteins) (Fig. 4a). The protein-protein interaction (PPI) network from Biogrid was downloaded to evaluate whether B-related proteins had functional interactions (Additional File 9). The PPI indicates the physical and high specific contact of two or more proteins. Due the lack of fish protein-protein interaction data base, the interactions were filtered based on the human set. As reported in several studies, there are several miRNA targets conserved among organisms [43, 44]. In this way, our extrapolation report mostly conserved interactions that could be confirmed by presence in the data bases, as the online TargetScan [42].

The original Biogrid PPI network contained 544,163 interactions (edges) between 24,913 proteins (nodes). After the experimental detection, redundant edges and species filtering procedures, 294,199 edges between 14,540 nodes were retained. Based on the 960 B-related proteins whose genes are B-DE-miRNA targets, and B genes identified in a previous study [11], a subnetwork extraction revealed 177 nodes and 335 edges in the Biogrid filtered network. The 177 nodes from the subnetwork are targets of 57 B-DE-miRNAs from the miRNA:3’UTR analysis. Finally, all miRNA targets and interactions were added to the subnetwork, resulting in 22,477 edges and 281 nodes (considering both proteins and miRNAs), which was called “B-miR-net” (Fig. 4b and Additional File 9).

Despite the ability of B-DE-miRNAs to connect to several mRNAs in B-miR-net, proteins that are regulated in consensus between different tissues are interconnected with each other (Fig. 4b – red axis). Interestingly, among the B-related proteins, connections exist with other proteins that may by indirectly influenced by B-DE-miRNAs (Fig. 4b – edges between red and blue axes). Additionally, 42 of the 102 genes reported on the B chromosome in a previous study [11] are in the B-miR-net, as they are B-DE-miRNA targets or interact with other proteins (Table 2). Nevertheless, 18 of these B-genes exhibit more than 80% of integrity in their coding region, meaning potentially translated proteins. The other 24 B-genes displayed less than 80% of integrity that may generate truncated transcripts what indicates protein translation problems (Table 2 and Additional File 7).

**Table 2:** B-genes present in the B-miR-net. B related proteins – proteins which either are targets of B-DE-miRNAs or interact with these targets. Other proteins: proteins which interact each other and are *A. latifasciata* miRNA targets

	Gene name	Protein Symbol	Integrity (%)
B-related proteins	apoptosis regulator Bcl-2-like	BCL2	100
	coxsackievirus and adenovirus receptor-like	CXAR	100
	spindle and kinetochore-associated protein 1-like	SKA1	100
	kinesin-like protein KIF11-like	KIF11	96.13
	zinc finger protein 771-like	ZNF71	89.37
	serine/threonine-protein kinase RIO3-like	RIOK3	81.57
	ATP-binding cassette sub-family A member 1-like	ABCA1	80.25
	aurora kinase A-B-like	AURKA	77.16
	centromere-associated protein E-like	CENPE	73.92
	zinc finger protein 836-like	ZN836	64.52
	histone-lysine N-methyltransferase MLL3-like	KMT2C	56.16
Other proteins	butyrophilin subfamily 2 member A1-like	BT2A1	100
	butyrophilin-like protein 2-like	BTNL2	100
	myosin-10-like	MYH10	100
	polymeric immunoglobulin receptor-like	PIGR	100
	serine protease 23-like	PRS23	100
	peptide chain release factor 1-like. Mitochondrial-like	RF1ML	100
	VIP peptides-like	VIP	100
	zinc finger protein 879-like	ZN879	100
	ATP-dependent RNA helicase DDX51-like	DDX51	92.22
	CD209 antigen-like	CD209	90.13
	protocadherin-10-like	PCD10	89.58
	heterogeneous nuclear ribonucleoprotein Q-like	HNRPQ	79.90
	leucine-rich repeat-containing protein 30-like	LRC30	71.93
	synaptonemal complex protein 2-like	SYCP2	70.97
	extracellular calcium-sensing receptor-like	CASR	69.46
	protein NLRC3-like	NLRC3	68.24
	guanine nucleotide-binding protein G	GNAI1	67.52
	vascular cell adhesion protein 1-like	VCAM1	65.79
	torsin-1A-interacting protein 2-like	ELOF1	64.25
	zinc finger protein 782-like	ZN782	63.42
	zinc finger protein 678-like	ZN678	63.01
	V-set domain-containing T-cell activation inhibitor 1-like	VTCN1	63.00
	poly [ADP-ribose] polymerase 14-like	PAR14	61.53
	DNA-directed RNA polymerase E subunit 1-like	RPA49	61.01
	endonuclease domain-containing 1 protein-like	ENDD1	58.21
	SAM domain and HD domain-containing protein 1-like	ESPL1	57.51
	targeting protein for Xklp2-A-like	TPX2	52.30
	interferon-induced very large GTPase 1-like	GVIN1	51.55
	sterile alpha motif domain-containing protein 12-like	SAM12	51.27
	xylulose kinase-like	XYLB	50.52
	glucose-6-phosphate 1-dehydrogenase-like	G6PD	50.43

**Fig. 5:**
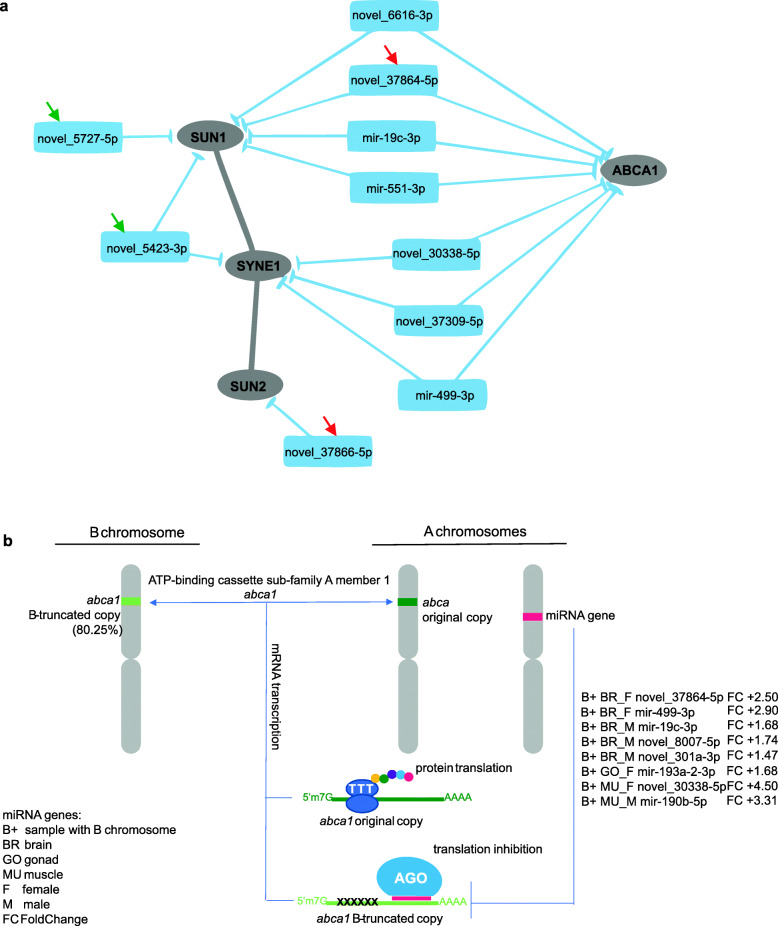
Potential activity of small RNA genes originated from A chromosomes and their action over the B chromosome. **a** B-DE-miRNAs targeting the genes of exclusive B-related protein GO terms. The arrows with the same color indicate clustered miRNAs. **b** The truncated B-genes of the B-miR-net compete to their original A copy and are controlled by B-DE-miRNAs in the way to benefit B chromosome maintenance

A Gene Ontology (GO) analysis was performed (Additional Files 8), and the proteins were analyzed in the groups of (I) B-related proteins (Fig. 4b – red axis) and (II) all proteins whose genes are targets of *A. latifasciata* miRNAs (Fig. 4b – blue axis). The results revealed 74 biological processes that were shared among the analyzed groups, meaning the common processes between B-related proteins and all *A. latifasciata* proteins (Fig. 4c, Additional File 10 – S2). In other words, processes that are commonly present in the cell might be affected by B chromosome presence by miRNA targeting. However, three specific GO terms were found in the B-related protein group, which indicates exclusive processes in the PPI network for genes controlled by the B-DE-miRNAs in all analyzed tissues. These process terms are nuclear matrix organization (GO:0043578), nuclear anchoring at nuclear membrane (GO:0090292), with members of these two terms being the SUN1 (*SUN domain-containing protein 1*), SUN2 (*SUN domain-containing protein 1*) and SYNE1 (*syne1*) proteins and response to stimulus (GO:0050896), with 141 proteins members (Additional File 9 – S1) (Fig. 4c). Furthermore, a B-gene, ATP-binding cassette sub-family A member 1 (*abca1*) with 80.25% of integrity, belongs to response of stimulus biological process. This gene is target of B^+^ upregulated miRNAs in brain, gonad and muscle as describe in the Fig. 5. As highlighted in the Fig. 5a, these two biological processes exclusive of B-related proteins are connected to each other by miRNA targeting.

Furthermore, ABCA1 and SUN2 interact with the proteins CDC42 (cell division control protein 42 homolog) and SKP1 (S-phase kinase associated protein 1), respectively. These two proteins also have GO terms which are common to B-related proteins and all the other proteins. This indicates potential biological processes which occurs in the cells and might be affected by the B chromosome presence. The potential consequences of this interaction under B chromosome presence are developed further in the Discussion section.

## Discussion

In the last few years, B chromosome science has undergone rapid advances due to the wide application of genomics and bioinformatics tools and functional approaches, including analyses of nonmodel species [45]. Here, we advanced the characterization of the miRNome in *A. latifasciata*, with a focus on the B chromosomes. Initial studies described approximately 200 miRNA genes in fish species [32], and later, this number was increased to 400 genes with the advances in large-scale DNA and RNA sequencing [39]. Here, we identified more than 700 pre-miRNAs, 34% of which are known miRNAs identified in miRBase (Fig. 1). A set of new pre-miRNAs in the *A. latifasciata* genome was described, confirming the rapid evolution of genomes in cichlid species [32, 39, 46]. New miRNAs could arise and be lost quickly mainly between related groups [39]. In this manner, the miRNAs could be tightly involved to the diverse adaptation of Cichlidae family [32, 39]. Some of novel pre-miRNA seeds are similar to cichlid seed sequences, and these new miRNAs are probably isomiRs originated from duplications or as products of RNA editing [43]. The new miRNAs are organized in clusters, which are usually controlled by the same factors to act in the same or related pathways [39]. The *A. latifasciata* species-specific isomiRs might perform new functions related to the B chromosome, as we can observe in the Fig. 5a.

Concerning the investigation of small sequences in the B chromosome, it was not possible to describe a pre-miRNA in the B extra element based on the adopted approaches. We pointed two reasons: the miRNAs evolution and the limitations of Illumina sequencing to investigated small sequences with low coverage. One of the main reasons for the lack of miRNAs is probably related to the B chromosome structure, which in *A. latifasciata* originated from a mosaic of duplicated sequences that underwent mutations over time [11]. Even though the difficulty of screening new miRNA sequences in a degenerated element (as the B chromosome), given the limitations of sRNAseq and genomic approaches, we cannot discard to find out small RNAs sequences in the B chromosome. Our results offered a miRNA genomic annotation and expression analysis of a new cichlid fish. This data can improve future studies by association with other genomic sequencing technologies that cover the B chromosome in a better way, such as Pacbio [47] and flow sorting [48].

We observed differences in the expression of miRNAs encoded by the regular chromosomes complement (B-DE-miRNAs) in B chromosome carriers. Similar results were also observed in maize [20]. The mechanism by which B chromosomes affect the expression of A complement miRNA genes is unclear. The presence of active genes related to miRNA biogenesis in the B chromosome might explain these variations. Argonaute (AGO) proteins are essential for processing small RNAs [25, 43], and although AGO-like protein genes have already been observed on the B chromosomes of *Secale cereale* [49], copies of these genes and other genes related to miRNA biogenesis were not found in *A. latifasciata* B chromosomes [11, 37]. Moreover, the expression of A copies of *drosha* and *dicer* genes, the main regulators of miRNA biogenesis, were not affected by B chromosomes (Additional File 5). Thus, the miRNA pathway does not appear to have changes under the B chromosome presence.

The B-DE-RNAs are specific to each tissue (Fig. 3), but their targets are usually the same (Fig. 4a). In other words, we have different B-DE-miRNAs targeting common proteins among the tissues and sex. Further, some of these proteins interacts each other in biological processes with relation to B chromosome presence (Fig. 4c). Among the target functions, the cell cycle pathway has attracted attention, since genes related to the cell cycle and chromosome segregation are registered as located on B chromosomes (for a review, see [50]). What would explain the differential miRNA expression in the presence of the B chromosome? Gene fragments with different degrees of integrity are present in the *A. latifasciata* B chromosome [11]. We suggest, if these truncated gene copies are transcribed, more binding sites will be available to miRNAs, generating regulatory competition with the A chromosome set (Fig. 5) [51]. We reported a B-gene, *abca1,* which has 80% of integrity and is a B-DE-miRNA target (Table 2 and Fig. 5a). Thus, according to the miRNAs controlling these genes, even truncated copies might display increased activity in the cell, further investigations are needed to confirm such assumption. Finally, because the miRNA targets are similar in different tissues, the gene functions affected by their regulation may favor the B chromosome to some extent [5, 14].

## Conclusions

We presented the *A. latifasciata* miRNome and compared it with other available miRNA databases. Additionally, several miRNAs were DE in the brain, gonads and muscle in the B^+^ samples and shared common genes as miRNA targets. The differentially expressed miRNAs detected in the presence of the B chromosome are not the same among the tissues, but the miRNA targets are involved in the same biological processes. Thus, we suggest that the B chromosome influences the cellular environment using miRNAs as a posttranscriptional control process that is probably for its own benefit of B drive and maintenance.

## Materials and methods

### Samples

*A. latifasciata* fishes were obtained from the Integrative Genomics Laboratory fish room at São Paulo State University, Botucatu (SP), Brazil and were genotyped for presence/absence of the B chromosome using the previously developed marker for B chromosomes [52]. The animals with B (B^+^) and without B (B^−^) were kept in different aquariums until the tissue collection procedure. We used four animals to each group: females B^−^, females B^+^, males B^−^ and males B^+^. Twelve animals (four of each group) were used to RNA-seq and twelve to DNA and RNA extraction to PCR, qPCR an RT-qPCR, totalizing 24 animals. The animals were submitted to euthanasia by immersion in eugenol 1% for 3 min, following the tissue extraction (brain, gonads and muscle) by liquid nitrogen flash frozen method. The tissues were stocked in − 80 refrigerators until the RNA and DNA extraction, described below.

### RNA sequencing of small RNAs in brain, gonads and muscle

The total RNA was extracted from the brain and gonads of six males and six females without B chromosomes (B-) and with B chromosomes (B^+^) and the muscles of four males and four females B^−^ and B^+^ using the TRIzol™ (Invitrogen) protocol. The samples were assessed for quality using electrophoresis on an agarose gel and RIN (RNA Integrity Number, > 8) parameters and then sent for small RNA sequencing (sRNAseq). This service was executed by Sequencing Service at LC Sciences - Houston, TX, USA, using a single-end Illumina HiSeq 2000 platform after the library construction with TruSeq® Small RNA Sample Preparation (Illumina). The libraries are deposited in the NCBI database (access numbers SRR13040679-SRR13040710).

### MicroRNome construction and expression analysis

The small RNA libraries were filtered for quality using Fastx-Toolkit (http://hannonlab.cshl.edu/fastx_toolkit/index.html) with the default parameters. Adaptors (TGGAA) and sequences shorter than 17 nucleotides and longer than 27 nucleotides were removed using Cutadapt (https://cutadapt.readthedocs.io/en/stable/) (Additional File 11 - Table S1).

The miRBase fish database [53] (Release 22.1, October 2018) (Additional File 10 - Table S3) was clustered using CD-hit software (default parameters) [54] to create a nonredundant reference list of fish miRNAs.

The filtered small RNA-Seq libraries were used as input for mapping based on default parameters [55] to predict and identify miRNAs (Additional File 11 - Table S2). Then, the nonredundant reference list of fish miRNAs was aligned to the *A. latifasciata* genome assembly [37] using Bowtie2 (v 2.3.3.1) [56]. The final prediction step consists of the submission of the mapper.pl output, the original nonredundant reference list and the aligned reference list to miRDeep2.0.1.2 analysis using default parameters [55]. The miRDeep software allows identifying known and novel miRNAs based on the existence of the miRNAs in the miRBase. The novel miRNAs were analyzed with BLAST on the seed region (2–8 nucleotides) to characterize if the new miRNA belongs to either a known miRNA family or a known miRNA derived [55].

The differential expression analysis was performed using miaRma pipeline [57] with the parameters desoft = EdgeR-Noiseq; filter = yes; cpmvalue = 1; repthreshold = 6; fc_threshold = 0.5; edger_normethod = TMM; replicates = yes; bcvvalue = 0.4; replicatevalue = biological; and noiseq_normethod = tmm. The comparisons were performed separately for each tissue (brain, gonads and muscle), sex (male and female) and the presence or absence of the B chromosome (B^+^/B^−^), always considering the B- as control. The miRNAs with *p* < 0.05 were considered differentially expressed (DE).

### Analysis of B chromosome copies

Three strategies were adopted to identify possible copies of miRNA genes related to the B chromosome. The first was based on “coverage ratio analysis” [11]. The miRNA annotations of the *A. latifasciata* genome were compared to the B^−^ and B^+^ read coverage ratios of the *A. latifasciata* genome. The regions with twice as high coverage read in the B+ genome indicate a duplicated B chromosome sequence copy [11] (i.e.: 60x coverage B^−^ reads and 120x coverage B^+^ reads). The second strategy was to use all miRNA reads (from B^−^ and B^+^ samples) that failed to align the *A. latifasciata* genome during miRNome construction to identify B chromosome-specific miRNAs. The unaligned reads were used for miRNA identification by miRDeep using the B^+^ genome assembly [37], followed by the prediction of miRNA:mRNA interactions by TargetScan (described further below). To analyze if these sequences are exclusive from B chromosome, we performed a BLAST search using *Metriaclima zebra* genome (v0, bouillabase.org, last accessed on October 16, 2020). The scaffold matched from *M. zebra* genome was visualized with *A. latifasciata* B^−^ and B^+^ genomic read alignments on Saci Base Jbrowser (https://sacibase.ibb.unesp.br/index.html, last accessed on October 16, 2020). Finally, the third strategy was based on the duplicated B chromosome blocks (B-blocks) identified in a previous study [37]. The previously predicted miRNAs located on B-blocks were used to predict the miRNA:mRNA interactions with TargetScan (described further below).

### 3’UTR prediction and miRNA:3’UTR interactions

The 3’UTR sequences from the *A. latifasciata* transcriptome [58] were predicted using Transdecoder (v5.5.0) [59]. The annotation file for each transcript was used with Bedtools (v2.26.2) [60] to retrieve the predicted 3’UTR for further analyses of interactions. Additionally, the transcript tissue specificity was retrieved based on observed expression [58] to assess tissue-specific interactions.

The miRNAs (novel and known) identified on miRNome and the 3’UTR from the *A. latifasciata* transcriptome were submitted to TargetScan 6.0 [42] to predict the miRNA:3’UTR interactions. Scores less than − 0.2 were considered significant predictions in accordance with software parameters.

### Genomic PCR, qPCR and RT-qPCR

Genomic DNA was extracted using [61] protocol. The samples were genotyped using the molecular marker for the presence of the B chromosomes [52]. Primers for mature miRNA sequences were constructed using Primer3Plus. Conventional genomic PCR was performed using *Taq* DNA Polymerase (Invitrogen-10,342-053) submitted to cycling at 94 °C for 5 min, followed by 35 cycles (94 °C for 1 min, 50 °C to 60 °C for 45 s, and 72 °C for 10 min) and 72 °C for 10 min. The result was verified by agarose gel electrophoresis (1%). The genomic DNA of eight B^−^ and eight B^+^ samples, including samples from males and females, was used for GDR analysis with qPCR and SYBR Green detection (Ampliqon) to verify the putative miRNAs associated with the B chromosome copy. The gene expression analysis was based on the total RNA extracted from the brain, gonads and muscle of B^−^ and B^+^ of both sexes, and performed in biological triplicates. The samples were converted to cDNA libraries (High-Capacity RNA-to-cDNA™ Kit, Applied Biosystems) and used for RT-qPCR with SYBR Green detection (Ampliqon). The ∆∆C_q_ method was used to analyze relative expression [62]. Q-Gene software [63] was used for normalization with the *ubiquitin-conjugating enzyme* (UBCE) as the reference gene. The primers are described in Additional File 11 - Table S4.

### Network construction and functional analysis

The human PPI network was downloaded from Biogrid to analyze miRNA functions [64]. The network was filtered to retain only nonredundant interactions between human proteins detected by affinity chromatography technology (MI:0004), X-ray crystallography (MI:0114), far western blotting (MI:0047), fluorescent resonance energy transfer (MI:0055), protein complementation assay (MI:0090), experimental interaction detection (MI:0045) and two hybrid (MI:0018) experiments. The annotated protein-coding transcripts [58] commonly regulated by B-DE-miRNAs in each tissue (brain, gonads and muscle), together with B genes reported in a previous study [11], were used to extract a PPI subnetwork from the filtered Biogrid. The miRNA:3’UTR interactions were added to the PPI subnetwork to create the final B-miR-net. The protein list was subjected to a Gene Ontology (GO) analysis using gProfiler to verify exclusive and specific GO terms for proteins derived from B-DE-miRNA targets and the remaining proteins from all *A. latifasciata* targets [65].

## Supplementary Information

**Additional file 1. **miRDeep2 – *Astatotilapia latifasciata* miRNA prediction.

**Additional file 2. **Html file of miRNA prediction. miRDeep2 – miRNA prediction in the B^+^ assembly using B^−^ RNAseq which did not align in the *A. latifasciata* genome.

**Additional file 3. **Html file of miRNA prediction. miRDeep2 – miRNA prediction in the B^+^ assembly using B^+^ RNAseq which did not align in the *A. latifasciata* genome.

**Additional file 4. **Nucleotide sequences (Figure S1) and reads coverage (Figure S2) of pre-miRNAs sequences on the B^+^ assembled genome. **Figure S1.** Sequence descriptions: #primer>NODE_contigXXXX Mzebra scaffold_XX start:end B^−^/B^+^ reads. The primer binding sites are highlighted in bold. **Figure S2.** Genome assembly bias. The red rectangles on the graphs highlight the pre-miRNAs regions in the *M. zebra* genome and *A. latifascita* B^−^ and B^+^ genomic reads datasets. These miRNAs were predicted in the B^+^ assembly using the reads that failed to align in the B^−^ reference assembly expecting to find some B^+^ chromosome miRNA.

**Additional file 5. **Expression of *drosha* and *dicer* genes.

**Additional file 6. **Html files containing miRNA prediction in B-blocks, separated by RNA-seq sample. **S1** miRDeep2 – miRNA prediction in B-blocks using brain female B^−^ RNA-seq. **S2** miRDeep2 – miRNA prediction in B-blocks using brain female B^+^ RNA-seq. **S3** miRDeep2 – miRNA prediction in B-blocks using brain male B^−^ RNA-seq. **S4** miRDeep2 – miRNA prediction in B-blocks using brain male B^+^ RNA-seq. **S5** miRDeep2 – miRNA prediction in B-blocks using gonads female B^−^ RNA-seq. **S6** miRDeep2 – miRNA prediction in B-blocks using gonads female B^+^ RNA-seq. **S7** miRDeep2 – miRNA prediction in B-blocks using gonads male B^−^ RNA-seq. **S8** miRDeep2 – miRNA prediction in B-blocks using gonads male RNA-seq. **S9** miRDeep2 – miRNA prediction in B-blocks using muscle female B^−^ RNA-seq. **S10** miRDeep2 – miRNA prediction in B-blocks using muscle female B^+^ RNA-seq. **S11** miRDeep2 – miRNA prediction in B-blocks using muscle male B^−^ RNA-seq. **S12** miRDeep2 – miRNA prediction in B-blocks using muscle male B^+^ RNA-seq.

**Additional file 7.** Multiple data sheets of samples separated by tissue and sex describing the B-DE-miRNA and its target.

**Additional file 8. **Multiple data sheets of samples separated by tissue and sex describing the B-DE miRNA target gene and protein names – recovered from *A. latifasciata* transcriptome annotation [58].

**Additional file 9. **Folder containing the .tsv files of networks. **S1.tsv** PPI network filtered from the Biogrid database. **S2.tsv** PPI of all proteins from *A. latifasciata* miRNA targets. **S3.tsv** B-related proteins and B genes subnetwork. **S4.tsv** B-miR-net.

**Additional file 10. **GO datasets. **S1 Exclusive** GO terms for proteins derived from B-DE-miRNA targets and B genes. **S2** Common GO terms among B-related proteins and other proteins. **S3** GO terms for all *A. latifasciata* targets in the PPI network.

**Additional file 11. **Samples and primers information. **Table S1.** RNA-seq quality and filtering. **Table S2.** Normalized reads mapped in the genome**. Table S3.** miRBase fish database entries used to create the reference miRNA list. **Table S4.** Primers used for genomic PCR, genomic qPCR and RT-qPCR.

## Data Availability

The *A. latifasciata* B^+^ assemblies and B-blocks are available in a previous study under Bioproject PRJN369442 access [37] and can be visualized on https://sacibase.ibb.unesp.br/. The *A. latifasciata* miRNAs sequences are shown in Additional Files. The targets sequences have been published [58]. The small RNA-seq libraries are deposited in the NCBI database (accession numbers from SRR13040679 to SRR13040710b).

## Abbreviations

3’UTR: 3′ untranslated region
B^−^: Samples without B chromosome
B^+^: Samples with B chromosome
B-blocks: B chromosome sequences
B-DE-miRNAs: Differentially expressed miRNAs in the B^+^ samples
B-miR-net: Protein-protein and B-DE-miRNAs interaction network
C_q_: Quantification cycle
DE miRNAs: Differentially expressed microRNAs
GDR: Gene dose ratio
GO: Gene Ontology
miRNA:mRNA: microRNA and messenger RNA interaction
miRNAs: microRNAs
mRNAs: Messenger RNA
PCR: Polymerase chain reaction
piRNAs: PIWI-Interacting RNAs
PPI: Protein-protein interaction
pre-miRNA: Precursor microRNA
pri-miRNA: Primary microRNA
PSR: Paternal sex ratio
qPCR: Quantitative real-time PCR
RIN: RNA Integrity Number
RNA-seq: RNA sequencing
RT-qPCR: Reverse transcription-qPCR
siRNAs: Small interference RNAs
