# Supplementary material for: Differential expression of miRNAs in the presence of B chromosome in the cichlid fish Astatotilapia latifasciata
Source: BMC Genomics. 2021 May 12;22:344. doi: 10.1186/s12864-021-07651-w (PMC8117508; doi:10.1186/s12864-021-07651-w)
Supplement: Supplementary file 1 — Additional file 1. miRDeep2 – Astatotilapia latifasciata miRNA prediction. [file 12864_2021_7651_MOESM1_ESM.html]

miRDeep2


#### B chromosome impacts cell physiology through the differential regulation of miRNAs in the cichlid fish *Astatotilapia latifasciata*, Nascimento-Oliveira, JIN et al.

| **miRDeep2 - *Astatotilapia latifasciata* miRNA prediction** | . |

## Parameters used

|  |  |
| --- | --- |
| miRDeep2 version | 2.0.1.2 |
  
| Program call | /home/jordana/mirdeep2-master/bin/miRDeep2.pl processed-reads 0bgenome reads\_collapsed\_vs\_genome.arf mirna-ala-2018-CERTO.fa maduros-mirbase-clusteredCERTO.fa mirbase-precursores-clustered2CERTO.fa |||  |  |
| --- | --- |
| Reads | processed-reads |
| Genome | 0bgenome |
| Mappings | reads\_collapsed\_vs\_genome.arf |
| Reference mature miRNAs | mirna-ala-2018-CERTO.fa |
| Other mature miRNAs | maduros-mirbase-clusteredCERTO.fa |

  
  

**Survey of miRDeep2 performance for score cut-offs -10 to 10**


|  | novel miRNAs | | | known miRBase miRNAs | | |  |  |
| --- | --- | --- | --- | --- | --- | --- | --- | --- |
 miRDeep2 scorefor details on how the log-odds score is calculated, see Friedlander et al., Nature Biotechnology, 2008. | predicted by miRDeep2novel miRNA hairpins are here defined by not having any of the reference mature miRNAs mapping perfectly (full length, no mismatches). The numbers show how many novel miRNA hairpins have a score equal to or exceeding the cut-off. | estimated false positivesnumber of false positive miRNA hairpins predicted at this cut-off, as estimated by the miRDeep2 controls (see Friedlander et al., Nature Biotechnology, 2008). Mean and standard deviation is estimated from 100 rounds of permuted controls. | estimated true positivesthe number of true positive miRNA hairpins is estimated as t = total novel miRNAs - false positive novel miRNAs. The percentage of the predicted novel miRNAs that is estimated to be true positives is calculated as p = t / total novel miRNAs. The number of false positives is estimated from 100 rounds of permuted controls. In each of the 100 rounds, t and p are calculated, generating mean and standard deviation of t and p. The variable p can be used as an estimation of miRDeep2 positive predictive value at the score cut-off. | in speciesnumber of reference mature miRNAs for that species given as input to miRDeep2. | in datanumber of reference mature miRNAs for that species that map perfectly (full length, no mismatches) to one or more of precursor candidates that have been excised from the genome by miRDeep2. | detected by miRDeep2number of reference mature miRNAs for that species that map perfectly (full length, no mismatches) to one or more of predicted miRNA hairpins that have a score equal to or exceeding the cut-off. The percentage of reference mature miRNAs in data that is detected by miRDeep2 is calculated as s = reference mature miRNAs detected / reference mature miRNAs in data. s can be used as an estimation of miRDeep2 sensitivity at the score cut-off. | estimated signal-to-noisefor the given score cut-off, the signal-to-noise ratio is estimated as r = total miRNA hairpins reported / mean estimated false positive miRNA hairpins over 100 rounds of permuted controls. | excision gearingthis is the minimum read stack height required for excising a potential miRNA precursor from the genome in this analysis. || 10 326 84 ± 10 242 ± 10 (74 ± 3%) 457 230 181 (79%) 5.8 32 | | | | | | | | |
| 9 326 84 ± 10 242 ± 10 (74 ± 3%) 457 230 181 (79%) 5.8 32 | | | | | | | | |
| 8 326 84 ± 10 242 ± 10 (74 ± 3%) 457 230 181 (79%) 5.8 32 | | | | | | | | |
| 7 326 84 ± 10 242 ± 10 (74 ± 3%) 457 230 181 (79%) 5.8 32 | | | | | | | | |
| 6 327 84 ± 10 243 ± 10 (74 ± 3%) 457 230 181 (79%) 5.8 32 | | | | | | | | |
| 5 342 85 ± 10 257 ± 10 (75 ± 3%) 457 230 196 (85%) 6 32 | | | | | | | | |
| 4 352 87 ± 10 265 ± 10 (75 ± 3%) 457 230 207 (90%) 6.1 32 | | | | | | | | |
| 3 355 105 ± 11 250 ± 11 (70 ± 3%) 457 230 208 (90%) 5.1 32 | | | | | | | | |
| 2 369 116 ± 12 253 ± 12 (69 ± 3%) 457 230 208 (90%) 4.8 32 | | | | | | | | |
| 1 434 128 ± 13 306 ± 13 (71 ± 3%) 457 230 209 (91%) 4.8 32 | | | | | | | | |
| 0 481 181 ± 14 300 ± 14 (62 ± 3%) 457 230 209 (91%) 3.6 32 | | | | | | | | |
| -1 499 281 ± 17 218 ± 17 (44 ± 3%) 457 230 210 (91%) 2.4 32 | | | | | | | | |
| -2 517 368 ± 18 149 ± 18 (29 ± 4%) 457 230 210 (91%) 1.9 32 | | | | | | | | |
| -3 573 447 ± 21 127 ± 21 (22 ± 4%) 457 230 211 (92%) 1.7 32 | | | | | | | | |
| -4 644 502 ± 22 142 ± 22 (22 ± 3%) 457 230 211 (92%) 1.7 32 | | | | | | | | |
| -5 717 547 ± 24 170 ± 24 (24 ± 3%) 457 230 211 (92%) 1.7 32 | | | | | | | | |
| -6 763 584 ± 23 179 ± 23 (23 ± 3%) 457 230 212 (92%) 1.6 32 | | | | | | | | |
| -7 790 613 ± 24 177 ± 24 (22 ± 3%) 457 230 212 (92%) 1.6 32 | | | | | | | | |
| -8 821 639 ± 24 182 ± 24 (22 ± 3%) 457 230 213 (93%) 1.6 32 | | | | | | | | |
| -9 841 658 ± 25 183 ± 25 (22 ± 3%) 457 230 213 (93%) 1.6 32 | | | | | | | | |
| -10 863 676 ± 25 187 ± 25 (22 ± 3%) 457 230 213 (93%) 1.6 32 | | | | | | | | |

  
  
  
  
  

## novel miRNAs predicted by miRDeep2

  


provisional idthis is a provisional miRNA name assigned by miRDeep2. The first part of the id designates the chromosome or genome contig on which the miRNA gene is located. The second part is a running number that is added to avoid identical ids. The running number is incremented by one for each potential miRNA precursor that is excised from the genome. Clicking this field will display a pdf of the structure, read signature and score breakdown of the reported miRNA. | miRDeep2 scorethe log-odds score assigned to the hairpin by miRDeep2 | estimated probability that the miRNA candidate is a true positivethe estimated probability that a predicted novel miRNA with a score of this or higher is a true positive. To see exactly how this probability is estimated, mouse over the 'novel miRNAs, true positives' in the table at the top of the webpage. | rfam alertthis field indicates if the predicted miRNA hairpin has sequence similarity to reference rRNAs or tRNAs. Warnings in this field should overrule the estimated probability that a reported miRNA is a true positive (previous field). | total read countthis is the sum of read counts for the predicted mature, loop and star miRNAs. | mature read countthis is the number of reads that map to the predicted miRNA hairpin and are contained in the sequence covered by the predicted mature miRNA, including 2 nts upstream and 5 nts downstream. | loop read countthis is the number of reads that map to the predicted miRNA hairpin and are contained in the sequence covered by the predicted miRNA loop, including 2 nts upstream and 5 nts downstream. | star read countthis is the number of reads that map to the predicted miRNA hairpin and are contained in the sequence covered by the predicted star miRNA, including 2 nts upstream and 5 nts downstream. | significant randfold p-valuethis field indicates if the estimated randfold p-value of the excised potential miRNA hairpin is equal to or lower than 0.05 (see Bonnet et al., Bioinformatics, 2004). | miRBase miRNAthis field displays the ids of any reference mature miRNAs for the species that map perfectly (full length, no mismatches) to the reported miRNA hairpin. If this is the case, the reported miRNA hairpin is assigned as a known miRNA. If not, it is assigned as a novel miRNA. If more than one reference mature miRNA maps to the miRNA hairpin, then only the id of the reference miRBase miRNA that matches the predicted mature sequence is output. | example miRBase miRNA with the same seedthis field displays the ids of any reference mature miRNAs from related species that have a seed sequence identical to that of the reported mature miRNA. The seed is here defined as nucleotides 2-8 from the 5' end of the mature miRNA. If more than one reference mature miRNA have identical seed, then only the id of the miRNA that occurs last in the input file of reference mature miRNAs from related species is displayed. | UCSC browserif a species name was input to miRDeep2, then clicking this field will initiate a UCSC blat search of the consensus precursor sequence against the reference genome. | NCBI blastnclicking this field will initiate a NCBI blastn search of the consensus precursor sequence against the nr/nt database (non-redundant collection of all NCBI nucleotide sequences). | consensus mature sequencethis is the consensus mature miRNA sequence as inferred from the deep sequencing reads. | consensus star sequencethis is the consensus star miRNA sequence as inferred from the deep sequencing reads. | consensus precursor sequencethis is the consensus precursor miRNA sequence as inferred from the deep sequencing reads. Note that this is the inferred Drosha hairpin product, and therefore does not include substantial flanking genomic sequence as does most miRBase precursors. | precursor coordinateThe given precursor coordinates refer do absolute position in the mapped reference sequence || NODE\_244240\_length\_103669\_cov\_30.213709\_17527 | 1.6e+7 | 0.74 ± 0.03 |  | 32162191 | 32161406 | 16 | 769 | yes |  | abu-miR-26b |  | blast | uucaaguaauccaggauaggcu | ccuaugcuugauuacuugcacu | uucaaguaauccaggauaggcuuucugcaucugcuuuggccuaugcuugauuacuugcacu | NODE\_244240\_length\_103669\_cov\_30.213709:25209..25270:- |
| NODE\_53673\_length\_1530\_cov\_25.384314\_4688 | 1.6e+7 | 0.74 ± 0.03 |  | 31690623 | 31687787 | 6 | 2830 | yes |  | abu-miR-26b |  | blast | uucaaguaauccaggauaggcu | ccuauucgugauuacuugcacu | uucaaguaauccaggauaggcuguguguauccuggauggccuauucgugauuacuugcacu | NODE\_53673\_length\_1530\_cov\_25.384314:1198..1259:+ |
| NODE\_73837\_length\_10885\_cov\_30.296095\_6100 | 1.6e+7 | 0.74 ± 0.03 |  | 31683459 | 31681269 | 1 | 2189 | yes |  | abu-miR-26b |  | blast | uucaaguaauccaggauaggcu | ccuauucuugauuacuuguuuc | uucaaguaauccaggauaggcugguuaacacugcacggccuauucuugauuacuuguuuc | NODE\_73837\_length\_10885\_cov\_30.296095:250..310:+ |
| NODE\_143105\_length\_7717\_cov\_25.466503\_10985 | 8.1e+6 | 0.74 ± 0.03 |  | 15948773 | 15911300 | 539 | 36934 | yes |  | abu-let-7d |  | blast | ugagguaguagguuguauaguu | uauacaacuuacugucuuuccu | ugagguaguagguuguauaguuuuagggucauucccaagcugucagaugacuauacaacuuacugucuuuccu | NODE\_143105\_length\_7717\_cov\_25.466503:3548..3621:- |
| NODE\_630262\_length\_18241\_cov\_27.215340\_38943 | 8.1e+6 | 0.74 ± 0.03 |  | 15948578 | 15911300 | 334 | 36944 | yes |  | abu-let-7d |  | blast | ugagguaguagguuguauaguu | uauacaacuuacugucuuuccu | ugagguaguagguuguauaguuuuagggucguacccuuccugucagauaacuauacaacuuacugucuuuccu | NODE\_630262\_length\_18241\_cov\_27.215340:9707..9780:- |
| NODE\_14422\_length\_15369\_cov\_26.303469\_1430 | 8.1e+6 | 0.74 ± 0.03 |  | 15903195 | 15886047 | 6 | 17142 | yes |  | abu-let-7d |  | blast | ugagguaguagguuguauaguu | cuauacagucuauugccuuccc | ugagguaguagguuguauaguuuggugggugggacugcacccugcucaggugauaacuauacagucuauugccuuccc | NODE\_14422\_length\_15369\_cov\_26.303469:11474..11552:+ |
| NODE\_717828\_length\_18120\_cov\_32.841335\_42720 | 8.1e+6 | 0.74 ± 0.03 |  | 15892423 | 15888978 | 125 | 3320 | yes |  | abu-let-7d |  | blast | ugagguaguagguuguauaguu | cuauacagccuauuaccuuccu | ugagguaguagguuguauaguuugugggauggacucaauccuauucaggugauaacuauacagccuauuaccuuccu | NODE\_717828\_length\_18120\_cov\_32.841335:13609..13686:+ |
| NODE\_34447\_length\_3591\_cov\_32.071568\_3100 | 8.0e+6 | 0.74 ± 0.03 |  | 15825282 | 15822826 | 73 | 2383 | yes |  | abu-let-7d |  | blast | ugagguaguagguuguauaguu | cuauucaaccuacugucuuucc | ugagguaguagguuguauaguuuuagggucauacccacacugggagauaacuauucaaccuacugucuuucc | NODE\_34447\_length\_3591\_cov\_32.071568:557..629:+ |
| NODE\_739733\_length\_16472\_cov\_25.832201\_43467 | 7.8e+6 | 0.74 ± 0.03 |  | 15329779 | 13151540 | 2 | 2178237 | yes |  | ccr-miR-199-5p |  | blast | cccaguguucagacuaccuguuc | acaguagucugcacauugguu | cccaguguucagacuaccuguucaucaggcugcagcugaacaguagucugcacauugguu | NODE\_739733\_length\_16472\_cov\_25.832201:10108..10168:- |
| NODE\_137919\_length\_3800\_cov\_22.979210\_10644 | 7.6e+6 | 0.74 ± 0.03 |  | 14987563 | 14986052 | 27 | 1484 | yes |  | abu-let-7d |  | blast | ugagguaguagguuguauaguu | cuguacaaccuccuagcuuucc | ugagguaguagguuguauaguugagaauuacaccccaggagauaacuguacaaccuccuagcuuucc | NODE\_137919\_length\_3800\_cov\_22.979210:247..314:+ |
| NODE\_173406\_length\_10615\_cov\_25.613283\_13044 | 7.3e+6 | 0.74 ± 0.03 |  | 14377490 | 14376982 | 0 | 508 | yes |  | gmo-miR-100b-5p |  | blast | aacccguagauccgaacuugug | caagcuuguaucuacaggucugc | aacccguagauccgaacuuguguuaagugacaccacaagcuuguaucuacaggucugc | NODE\_173406\_length\_10615\_cov\_25.613283:9626..9684:+ |
| NODE\_817731\_length\_59985\_cov\_31.449245\_45822 | 6.9e+6 | 0.74 ± 0.03 |  | 13619221 | 13607985 | 0 | 11236 | yes |  | gmo-miR-100b-5p |  | blast | aacccguagauccgaacuugug | caagcucguaucuauagguaug | aacccguagauccgaacuuguggugacuggcugcacaagcucguaucuauagguaug | NODE\_817731\_length\_59985\_cov\_31.449245:51836..51893:+ |
| NODE\_109907\_length\_4445\_cov\_21.460968\_8733 | 6.6e+6 | 0.74 ± 0.03 |  | 13122165 | 13110408 | 46 | 11711 | yes |  | gmo-miR-125a-5p |  | blast | ucccugagacccuaacuugug | acggguuaggcucuugggacgc | ucccugagacccuaacuugugagcucucuugaugaaaaaucacggguuaggcucuugggacgc | NODE\_109907\_length\_4445\_cov\_21.460968:3211..3274:- |
| NODE\_8395\_length\_10084\_cov\_28.521816\_892 | 6.6e+6 | 0.74 ± 0.03 |  | 13116665 | 13071491 | 92 | 45082 | yes |  | gmo-miR-125a-5p |  | blast | ucccugagacccuaacuugug | acggguuaggcucucgggagcu | ucccugagacccuaacuugugacguugugcuuucauguccacggguuaggcucucgggagcu | NODE\_8395\_length\_10084\_cov\_28.521816:1361..1423:+ |
| NODE\_817731\_length\_59985\_cov\_31.449245\_45826 | 6.6e+6 | 0.74 ± 0.03 |  | 13081054 | 13071307 | 151 | 9596 | yes |  | gmo-miR-125a-5p |  | blast | ucccugagacccuaacuugug | acggguuggguucuugggagcu | ucccugagacccuaacuugugacguuuugcugugaugugcacggguuggguucuugggagcu | NODE\_817731\_length\_59985\_cov\_31.449245:57062..57124:+ |
| NODE\_255582\_length\_23006\_cov\_27.789360\_18137 | 4.3e+6 | 0.74 ± 0.03 |  | 8579639 | 8574833 | 0 | 4806 | yes |  | gmo-miR-125a-5p |  | blast | ucccugagacccuuaaccugug | caggugagguccuugggaac | ucccugagacccuuaaccugugaugauaugaaaggucacaggugagguccuugggaac | NODE\_255582\_length\_23006\_cov\_27.789360:1205..1263:- |
| NODE\_960524\_length\_34399\_cov\_25.984476\_48215 | 3.4e+6 | 0.74 ± 0.03 |  | 6793713 | 6776697 | 39 | 16977 | yes |  | ssa-miR-21a-5p |  | blast | uagcuuaucagacugguguuggc | caacagcggucuguaagcuggc | uagcuuaucagacugguguuggcuguuggaugugcuuggcaacagcggucuguaagcuggc | NODE\_960524\_length\_34399\_cov\_25.984476:12443..12504:- |
| NODE\_369853\_length\_84\_cov\_31.547619\_24723 | 2.4e+6 | 0.74 ± 0.03 |  | 4715027 | 4707888 | 1578 | 5561 | yes |  |  |  | blast | guugacgaaguauccguggcu | cauggauacaacgucauaucc | cauggauacaacgucauauccuuagguaguggauuggguugacgaaguauccguggcu | NODE\_369853\_length\_84\_cov\_31.547619:76..134:+ |
| NODE\_374673\_length\_37756\_cov\_28.992161\_25156 | 2.3e+6 | 0.74 ± 0.03 |  | 4564570 | 4549297 | 1 | 15272 | yes |  | dre-miR-146b |  | blast | ugagaacugaauuccauagaugg | aucuaugggcucaguucuuuugg | ugagaacugaauuccauagauggugacaucuucaggugucaucuaugggcucaguucuuuugg | NODE\_374673\_length\_37756\_cov\_28.992161:36480..36543:+ |
| NODE\_158712\_length\_5438\_cov\_25.482714\_12076 | 2.1e+6 | 0.74 ± 0.03 |  | 4187393 | 4186943 | 1 | 449 | yes |  | ccr-miR-10c |  | blast | uacccuguagaaccgaauuugu | caaauacgucucuacaggaaua | uacccuguagaaccgaauuugugugaaguacaagcagucgcaaauacgucucuacaggaaua | NODE\_158712\_length\_5438\_cov\_25.482714:303..365:+ |
| NODE\_246094\_length\_13317\_cov\_27.872944\_17741 | 1.7e+6 | 0.74 ± 0.03 |  | 3519463 | 3502536 | 0 | 16927 | no |  | gmo-miR-100b-5p |  | blast | aacccguagauccgaucuugu | caagcucgccucugugggucu | aacccguagauccgaucuuguggcgaaucugacagcacaagcucgccucugugggucu | NODE\_246094\_length\_13317\_cov\_27.872944:5295..5353:- |
| NODE\_540432\_length\_66809\_cov\_33.349190\_34313 | 1.4e+6 | 0.74 ± 0.03 |  | 2875730 | 2875300 | 0 | 430 | yes |  | abu-miR-25 |  | blast | uauugcacuugucccggccugu | agguugggagagguggcaaugcu | agguugggagagguggcaaugcucugugcuugugugguauugcacuugucccggccugu | NODE\_540432\_length\_66809\_cov\_33.349190:61471..61530:+ |
| NODE\_749189\_length\_52450\_cov\_27.729570\_43733 | 1.3e+6 | 0.74 ± 0.03 |  | 2739592 | 2737553 | 0 | 2039 | yes |  | abu-miR-25 |  | blast | uauugcacuugucccggccugu | agguggggaucgguagcaaugcu | agguggggaucgguagcaaugcuguguuccuccagguauugcacuugucccggccugu | NODE\_749189\_length\_52450\_cov\_27.729570:27841..27899:+ |
| NODE\_576206\_length\_29714\_cov\_25.878004\_36135 | 1.1e+6 | 0.74 ± 0.03 |  | 2325199 | 2319355 | 153 | 5691 | yes |  | abu-let-7d |  | blast | ugagguaguaguuuguauaguu | cuauacagucuacugucuuucu | ugagguaguaguuuguauaguuuuaggaucacaccagaucugggagauaacuauacagucuacugucuuucu | NODE\_576206\_length\_29714\_cov\_25.878004:22176..22248:- |
| NODE\_16374\_length\_4783\_cov\_26.798244\_1592 | 1.1e+6 | 0.74 ± 0.03 |  | 2320456 | 2319556 | 153 | 747 | yes |  | abu-let-7d |  | blast | ugagguaguaguuuguauaguu | cuauacagccuacugucuuucu | ugagguaguaguuuguauaguuuuaggaucacaccagaucugggagauaacuauacagccuacugucuuucu | NODE\_16374\_length\_4783\_cov\_26.798244:3856..3928:+ |
| NODE\_255582\_length\_23006\_cov\_27.789360\_18139 | 6.4e+5 | 0.74 ± 0.03 |  | 1260604 | 1256954 | 1541 | 2109 | yes |  | abu-let-7d |  | blast | ugagguaguugguuguaugguu | cuguacaaccuucuagcuuucc | ugagguaguugguuguaugguuucgcauaauaaaaagccuggagauaacuguacaaccuucuagcuuucc | NODE\_255582\_length\_23006\_cov\_27.789360:5207..5277:- |
| NODE\_565393\_length\_4672\_cov\_21.562714\_35558 | 6.4e+5 | 0.74 ± 0.03 |  | 1259123 | 1256954 | 60 | 2109 | yes |  | abu-let-7d |  | blast | ugagguaguugguuguaugguu | cuguacaaccuucuagcuuucc | ugagguaguugguuguaugguuucgcauaauaaacagcauggagauaacuguacaaccuucuagcuuucc | NODE\_565393\_length\_4672\_cov\_21.562714:337..407:+ |
| NODE\_597752\_length\_729\_cov\_15.968450\_37153 | 6.0e+5 | 0.74 ± 0.03 |  | 1183903 | 1182255 | 0 | 1648 | yes |  | ccr-miR-10c |  | blast | uacccuguagauccggauuugu | acaaauucgcuucuaggggagu | uacccuguagauccggauuuguguaaaaaucauuaauaaaaucacaaauucgcuucuaggggagu | NODE\_597752\_length\_729\_cov\_15.968450:386..451:- |
| NODE\_273565\_length\_9072\_cov\_28.341160\_19296 | 5.7e+5 | 0.74 ± 0.03 |  | 1135253 | 1131515 | 16 | 3722 | yes |  | abu-miR-202 |  | blast | uuccuaugcacauacuucuuu | aaagaggcauaaggcaugggaa | uuccuaugcacauacuucuuugagauuuaacuuuaaagaggcauaaggcaugggaa | NODE\_273565\_length\_9072\_cov\_28.341160:3759..3815:+ |
| NODE\_67770\_length\_7203\_cov\_31.656115\_5590 | 5.7e+5 | 0.74 ± 0.03 |  | 1127078 | 1125825 | 0 | 1253 | yes |  |  |  | blast | acccuguagauccgaauuugu | caaauucgcaucuuggggagu | acccuguagauccgaauuuguguaaauacagcagcagccacaaauucgcaucuuggggagu | NODE\_67770\_length\_7203\_cov\_31.656115:6029..6090:+ |
| NODE\_306418\_length\_35347\_cov\_27.355589\_21230 | 5.7e+5 | 0.74 ± 0.03 |  | 1124591 | 1121832 | 955 | 1804 | yes |  | ccr-miR-7a |  | blast | uggaagacuagugauuuuguugu | caacaaaucacagucugccaaa | uggaagacuagugauuuuguuguuuuuaguuagagcaaccgacaacaaaucacagucugccaaa | NODE\_306418\_length\_35347\_cov\_27.355589:22119..22183:+ |
| NODE\_587807\_length\_19476\_cov\_27.154549\_36637 | 5.7e+5 | 0.74 ± 0.03 |  | 1121100 | 1120970 | 0 | 130 | yes |  | ccr-miR-7a |  | blast | uggaagacuagugauuuuguugu | caacaaaucauugucucccuca | uggaagacuagugauuuuguuguuuguuguuuaaaaggacaacaaaucauugucucccuca | NODE\_587807\_length\_19476\_cov\_27.154549:8754..8815:- |
| NODE\_97739\_length\_10382\_cov\_30.511654\_7882 | 5.6e+5 | 0.74 ± 0.03 |  | 1113124 | 1111737 | 28 | 1359 | yes |  | ccr-miR-7a |  | blast | uggaagacuagugauuuuguugu | aacagagucacagucuaccuc | uggaagacuagugauuuuguuguuguaaauucauguuuuugacaacagagucacagucuaccuc | NODE\_97739\_length\_10382\_cov\_30.511654:1933..1997:+ |
| NODE\_298970\_length\_7313\_cov\_33.768631\_20651 | 4.9e+5 | 0.74 ± 0.03 |  | 977035 | 968789 | 0 | 8246 | yes |  | mze-miR-150 |  | blast | acucccaauccuuguaccagugu | cgcuggacggguuuugggggggc | acucccaauccuuguaccagugucuugacaccagugacgcuggacggguuuugggggggc | NODE\_298970\_length\_7313\_cov\_33.768631:6479..6539:- |
| NODE\_206245\_length\_8382\_cov\_28.398233\_15212 | 4.6e+5 | 0.74 ± 0.03 |  | 921226 | 917155 | 1 | 4070 | yes |  | abu-miR-203 |  | blast | gugaaauguuuaggaccacuug | agugguucuagauaguucaaca | agugguucuagauaguucaacaguacagcauucguuugugaaauguuuaggaccacuug | NODE\_206245\_length\_8382\_cov\_28.398233:6136..6195:- |
| NODE\_329976\_length\_33383\_cov\_27.931822\_22557 | 4.6e+5 | 0.74 ± 0.03 |  | 909347 | 893882 | 0 | 15465 | yes |  | abu-miR-203 |  | blast | gugaaauguuuaggaccacuug | agugguucucaacaguucaaca | agugguucucaacaguucaacaguucuuuaacaaaauugugaaauguuuaggaccacuug | NODE\_329976\_length\_33383\_cov\_27.931822:2661..2721:+ |
| NODE\_48958\_length\_5169\_cov\_27.579609\_4254 | 4.1e+5 | 0.74 ± 0.03 |  | 809129 | 767817 | 7 | 41305 | yes |  | abu-miR-30a-5p |  | blast | uguaaacauccuacacucucggc | ccgggagugggacuguuugcacu | uguaaacauccuacacucucggcaucugcccucugguggccgggagugggacuguuugcacu | NODE\_48958\_length\_5169\_cov\_27.579609:408..470:+ |
| NODE\_34447\_length\_3591\_cov\_32.071568\_3102 | 3.7e+5 | 0.74 ± 0.03 |  | 729215 | 728021 | 21 | 1173 | yes |  | abu-let-7d |  | blast | ugagguaguagauuguguaguu | cuauacaaucuauugccuuccc | ugagguaguagauuguguaguuuuaggguagugauuuugcccucuuuaggagauaacuauacaaucuauugccuuccc | NODE\_34447\_length\_3591\_cov\_32.071568:742..820:+ |
| NODE\_533907\_length\_36799\_cov\_27.206146\_33971 | 3.6e+5 | 0.74 ± 0.03 |  | 706812 | 698235 | 8220 | 357 | yes |  | abu-miR-15a |  | blast | uagcagcacguaaauauuggag | ccaguauugaucguacugcu | uagcagcacguaaauauuggaguuaacacucuagcugaagucuccaguauugaucguacugcu | NODE\_533907\_length\_36799\_cov\_27.206146:9057..9120:- |
| NODE\_295423\_length\_9129\_cov\_34.133091\_20472 | 2.8e+5 | 0.74 ± 0.03 |  | 554860 | 546026 | 33 | 8801 | yes |  | abu-miR-30a-5p |  | blast | uguaaacauccuacacucagcu | cugagaggagguuguuuacuug | uguaaacauccuacacucagcuguuauugauugccacagaggcugagaggagguuguuuacuug | NODE\_295423\_length\_9129\_cov\_34.133091:7522..7586:+ |
| NODE\_246094\_length\_13317\_cov\_27.872944\_17739 | 2.7e+5 | 0.74 ± 0.03 |  | 542115 | 537087 | 162 | 4866 | yes |  | abu-let-7d |  | blast | ugagguaguagguuguaugguu | cuguacaaccuucuagcuuucc | ugagguaguagguuguaugguuuagaauuacacccugggaguuaacuguacaaccuucuagcuuucc | NODE\_246094\_length\_13317\_cov\_27.872944:5021..5088:- |
| NODE\_157412\_length\_1687\_cov\_20.292828\_11898 | 2.4e+5 | 0.74 ± 0.03 |  | 483214 | 482877 | 0 | 337 | yes |  | abu-miR-25 |  | blast | uauugcacucgucccggccucc | cagguccgggauguggugcgcu | cagguccgggauguggugcgcuguugucauaucuccccuccaauauugcacucgucccggccucc | NODE\_157412\_length\_1687\_cov\_20.292828:731..796:- |
| NODE\_474461\_length\_15389\_cov\_25.790890\_30338 | 1.5e+5 | 0.74 ± 0.03 |  | 312220 | 312191 | 0 | 29 | yes |  | abu-miR-20a |  | blast | uaaagugcaugagugagcuca | gaguucacuugagcacauuucu | uaaagugcaugagugagcucagguauguaguagcugaguucacuugagcacauuucu | NODE\_474461\_length\_15389\_cov\_25.790890:6155..6212:+ |
| NODE\_65503\_length\_17390\_cov\_27.012823\_5423 | 1.5e+5 | 0.74 ± 0.03 |  | 310082 | 307695 | 0 | 2387 | yes |  | ccr-miR-217 |  | blast | uacugcaucaggaacugauuggc | caacaguaccugaugcauugcc | uacugcaucaggaacugauuggcuaaugcucaauugccaacaguaccugaugcauugcc | NODE\_65503\_length\_17390\_cov\_27.012823:4439..4498:- |
| NODE\_843582\_length\_56377\_cov\_27.843359\_46550 | 1.4e+5 | 0.74 ± 0.03 |  | 279678 | 278764 | 248 | 666 | yes |  | abu-miR-23c |  | blast | aucacauugccagggauuucc | ggguuccuggcaccgugauuu | ggguuccuggcaccgugauuugaugcacaaagagaaacaaaaaucacauugccagggauuucc | NODE\_843582\_length\_56377\_cov\_27.843359:12021..12084:- |
| NODE\_736889\_length\_39316\_cov\_28.766634\_43375 | 1.4e+5 | 0.74 ± 0.03 |  | 279647 | 278054 | 0 | 1593 | yes |  |  |  | blast | uauugcuugagaauacgcguag | cacggguauucuuggguugau | cacggguauucuuggguugauaauacagauguggauguuauugcuugagaauacgcguag | NODE\_736889\_length\_39316\_cov\_28.766634:29010..29070:+ |
| NODE\_24835\_length\_13316\_cov\_31.743092\_2334 | 1.4e+5 | 0.74 ± 0.03 |  | 278186 | 277615 | 32 | 539 | yes |  |  |  | blast | acagcaggcacagacaggcaga | ugccuaucuacacuugcugugc | ugccuaucuacacuugcugugcagaauauccuccaaccuguacagcaggcacagacaggcaga | NODE\_24835\_length\_13316\_cov\_31.743092:12453..12516:- |
| NODE\_65503\_length\_17390\_cov\_27.012823\_5425 | 1.3e+5 | 0.74 ± 0.03 |  | 269355 | 267316 | 69 | 1970 | yes |  | abu-miR-216a |  | blast | aaaucucagcuggcaacuguga | cacaauggccucugggauuaug | aaaucucagcuggcaacugugagucguucacuagcugcucucacaauggccucugggauuaug | NODE\_65503\_length\_17390\_cov\_27.012823:5012..5075:- |
| NODE\_623129\_length\_58502\_cov\_28.402893\_38405 | 1.1e+5 | 0.74 ± 0.03 |  | 234379 | 198949 | 0 | 35430 | yes |  | ola-miR-152 |  | blast | ucagugcauuacagaacuuugu | aaguucugugauacacuuagacu | aaguucugugauacacuuagacucuaacugcccgcagucagugcauuacagaacuuugu | NODE\_623129\_length\_58502\_cov\_28.402893:29294..29353:+ |
| NODE\_737847\_length\_83181\_cov\_28.914331\_43395 | 9.4e+4 | 0.74 ± 0.03 |  | 184967 | 182062 | 10 | 2895 | yes |  | abu-miR-27c |  | blast | uucacaguggcuaaguuccgc | aggacuuagcucgcuccguga | aggacuuagcucgcuccgugaacaguucagugaaguccuguguucacaguggcuaaguuccgc | NODE\_737847\_length\_83181\_cov\_28.914331:30869..30932:+ |
| NODE\_733430\_length\_298\_cov\_12.906040\_43297 | 7.9e+4 | 0.74 ± 0.03 |  | 155701 | 132140 | 294 | 23267 | yes |  | gmo-miR-11231-3p |  | blast | gcugacgguguauccguggcu | cacggaugcaacgucauaucc | cacggaugcaacgucauauccuuagguaguagauugggcugacgguguauccguggcu | NODE\_733430\_length\_298\_cov\_12.906040:37..95:- |
| NODE\_19801\_length\_65\_cov\_21.861538\_2018 | 7.9e+4 | 0.74 ± 0.03 |  | 155701 | 132140 | 294 | 23267 | yes |  | gmo-miR-11231-3p |  | blast | gcugacgguguauccguggcu | cacggaugcaacgucauaucc | cacggaugcaacgucauauccuuagguaguagauugggcugacgguguauccguggcu | NODE\_19801\_length\_65\_cov\_21.861538:34..92:+ |
| NODE\_35815\_length\_1383\_cov\_29.261026\_3187 | 6.2e+4 | 0.74 ± 0.03 |  | 121790 | 121477 | 0 | 313 | yes |  | eel-miR-7552-5p |  | blast | uuacaauuaaaggauauuucuu | aaaugucuuuuaauuguucggu | uuacaauuaaaggauauuucuugcgaaugacucagaaaugucuuuuaauuguucggu | NODE\_35815\_length\_1383\_cov\_29.261026:1289..1346:- |
| NODE\_65503\_length\_17390\_cov\_27.012823\_5427 | 5.9e+4 | 0.74 ± 0.03 |  | 116001 | 114318 | 40 | 1643 | yes |  | abu-miR-216b |  | blast | uaaucucugcaggcaacuguga | acaaucaccuggagagauucu | uaaucucugcaggcaacugugauggugcuuuauuuucucacaaucaccuggagagauucu | NODE\_65503\_length\_17390\_cov\_27.012823:5708..5768:- |
| NODE\_450908\_length\_48195\_cov\_31.381699\_29139 | 5.3e+4 | 0.74 ± 0.03 |  | 104535 | 90769 | 16 | 13750 | yes |  | ccr-miR-132a |  | blast | uaacagucuacagccaugguc | accguggcauuagauuguuacu | accguggcauuagauuguuacuguaguaacagcaccagugguaacagucuacagccaugguc | NODE\_450908\_length\_48195\_cov\_31.381699:12754..12816:+ |
| NODE\_749189\_length\_52450\_cov\_27.729570\_43730 | 5.1e+4 | 0.74 ± 0.03 |  | 101118 | 88912 | 0 | 12206 | yes |  | abu-miR-20a |  | blast | uaaagugcuuauagugcagguag | acugcaauguaagcacuugaag | uaaagugcuuauagugcagguaguguuuugcacaaucuacugcaauguaagcacuugaag | NODE\_749189\_length\_52450\_cov\_27.729570:27549..27609:+ |
| NODE\_91705\_length\_15181\_cov\_29.275410\_7483 | 5.1e+4 | 0.74 ± 0.03 |  | 100945 | 100931 | 3 | 11 | yes |  | gmo-miR-2184-5p |  | blast | aacaguaagaguuuaugugcu | gcacaugggcuuuuacggugca | aacaguaagaguuuaugugcuguucuuuaacucucagcacaugggcuuuuacggugca | NODE\_91705\_length\_15181\_cov\_29.275410:4415..4473:- |
| NODE\_776042\_length\_57406\_cov\_29.438055\_44546 | 5.0e+4 | 0.74 ± 0.03 |  | 98658 | 98580 | 1 | 77 | yes |  | abu-miR-375 |  | blast | uuuguucguucggcucgcguua | acguugagccucacguacaauacc | acguugagccucacguacaauaccugaagaugaaguuuuguucguucggcucgcguua | NODE\_776042\_length\_57406\_cov\_29.438055:44063..44121:- |
| NODE\_540432\_length\_66809\_cov\_33.349190\_34304 | 4.6e+4 | 0.74 ± 0.03 |  | 91340 | 90355 | 1 | 984 | yes |  | abu-miR-20a |  | blast | caaagugcuuacagugcaggua | cugcagugaaggcacuuucagc | caaagugcuuacagugcagguaguuuuguucaauucuacugcagugaaggcacuuucagc | NODE\_540432\_length\_66809\_cov\_33.349190:60462..60522:+ |
| NODE\_749189\_length\_52450\_cov\_27.729570\_43724 | 4.6e+4 | 0.74 ± 0.03 |  | 90811 | 89848 | 6 | 957 | yes |  | abu-miR-20a |  | blast | caaagugcuuacagugcaggua | cugcaguggaggcacuuacagca | caaagugcuuacagugcagguaguacuauggaauaccuacugcaguggaggcacuuacagca | NODE\_749189\_length\_52450\_cov\_27.729570:26829..26891:+ |
| NODE\_540432\_length\_66809\_cov\_33.349190\_34310 | 4.6e+4 | 0.74 ± 0.03 |  | 90480 | 88952 | 665 | 863 | yes |  | abu-miR-20a |  | blast | uaaagugcuuauagugcagguag | acugcaaugugagcacuucaagu | uaaagugcuuauagugcagguagucuucuuacuguuuaaauucuacugcaaugugagcacuucaagu | NODE\_540432\_length\_66809\_cov\_33.349190:61235..61302:+ |
| NODE\_424631\_length\_3119\_cov\_27.136581\_27837 | 4.3e+4 | 0.74 ± 0.03 |  | 85949 | 70097 | 2 | 15850 | yes |  | abu-miR-29d |  | blast | uagcaccauuugaaaucgguu | acugauuucuucuggugcuu | acugauuucuucuggugcuuagagcccgcugaagccuucuagcaccauuugaaaucgguu | NODE\_424631\_length\_3119\_cov\_27.136581:2795..2855:+ |
| NODE\_63321\_length\_37455\_cov\_34.655827\_5294 | 3.6e+4 | 0.74 ± 0.03 |  | 70894 | 67834 | 0 | 3060 | yes |  | abu-miR-29d |  | blast | uagcaccauuugaaaucgguu | accguuuuccucugguguucaga | accguuuuccucugguguucagaguuuccaucaugucuagcaccauuugaaaucgguu | NODE\_63321\_length\_37455\_cov\_34.655827:23759..23817:- |
| NODE\_628735\_length\_9290\_cov\_22.721205\_38716 | 3.6e+4 | 0.74 ± 0.03 |  | 70679 | 70643 | 1 | 35 | no |  | dre-miR-181a-5p |  | blast | aacauucauugcugucgcugggu | ucacugaacaaugagugcaac | aacauucauugcugucgcuggguuggacuguguagaaaagcucacugaacaaugagugcaac | NODE\_628735\_length\_9290\_cov\_22.721205:2372..2434:+ |
| NODE\_616364\_length\_18799\_cov\_29.315123\_37899 | 3.4e+4 | 0.74 ± 0.03 |  | 67911 | 67824 | 1 | 86 | yes |  | abu-miR-29d |  | blast | uagcaccauuugaaaucgguu | cgauuucuuucgguguucaga | cgauuucuuucgguguucagagucugcuucuguuucuagcaccauuugaaaucgguu | NODE\_616364\_length\_18799\_cov\_29.315123:10007..10064:+ |
| NODE\_256492\_length\_111\_cov\_31.306307\_18210 | 2.9e+4 | 0.74 ± 0.03 |  | 57909 | 57846 | 0 | 63 | no |  |  |  | blast | caaauugacaugaaggcgcuuu | aaguggcuuugguugguuucggu | aaguggcuuugguugguuucgguaagaauagguaaacaaauugacaugaaggcgcuuu | NODE\_256492\_length\_111\_cov\_31.306307:42..100:- |
| NODE\_798382\_length\_5716\_cov\_24.112492\_45356 | 2.8e+4 | 0.74 ± 0.03 |  | 56369 | 49948 | 1 | 6420 | no |  |  |  | blast | caaaacacaccgcugucacuuu | aagugacuuugguugucuuugg | aagugacuuugguugucuuugguaaauaccugucaucaaaacacaccgcugucacuuu | NODE\_798382\_length\_5716\_cov\_24.112492:1644..1702:- |
| NODE\_603086\_length\_35849\_cov\_31.646378\_37309 | 2.7e+4 | 0.74 ± 0.03 |  | 54022 | 53578 | 1 | 443 | yes |  | abu-miR-103 |  | blast | agcagcauuguacagggcuauc | agcuucuuuacaguguugccuugu | agcuucuuuacaguguugccuuguggcauggcgaucaagcagcauuguacagggcuauc | NODE\_603086\_length\_35849\_cov\_31.646378:24049..24108:+ |
| NODE\_368289\_length\_16781\_cov\_28.866396\_24630 | 2.3e+4 | 0.74 ± 0.03 |  | 45502 | 45389 | 0 | 113 | yes |  | abu-miR-218b |  | blast | uugugcuugaucuaaccaugugg | aacaugguucuuucaagcacc | uugugcuugaucuaaccauguggcugccagguuccuaucguggaacaugguucuuucaagcacc | NODE\_368289\_length\_16781\_cov\_28.866396:4723..4787:+ |
| NODE\_374443\_length\_20711\_cov\_30.724688\_25147 | 2.3e+4 | 0.74 ± 0.03 |  | 45471 | 45388 | 64 | 19 | yes |  | abu-miR-218b |  | blast | uugugcuugaucuaaccaugugg | caugguuccgucaagcgc | uugugcuugaucuaaccauguggccccgccuacuguaacaucacaugguuccgucaagcgc | NODE\_374443\_length\_20711\_cov\_30.724688:4098..4159:- |
| NODE\_616364\_length\_18799\_cov\_29.315123\_37897 | 1.7e+4 | 0.74 ± 0.03 |  | 34672 | 34034 | 0 | 638 | yes |  | abu-miR-29d |  | blast | uagcaccauuugaaaucagugu | gcugguuucagaugguggcuuaga | gcugguuucagaugguggcuuagaguauugucaucuaucuagcaccauuugaaaucagugu | NODE\_616364\_length\_18799\_cov\_29.315123:9764..9825:+ |
| NODE\_424631\_length\_3119\_cov\_27.136581\_27835 | 1.7e+4 | 0.74 ± 0.03 |  | 34663 | 34034 | 480 | 149 | yes |  | abu-miR-29d |  | blast | uagcaccauuugaaaucagugu | cugguuucacguggugguuuaga | cugguuucacguggugguuuagaugugugcugcucuugucuagcaccauuugaaaucagugu | NODE\_424631\_length\_3119\_cov\_27.136581:2630..2692:+ |
| NODE\_615561\_length\_2028\_cov\_24.085798\_37864 | 1.7e+4 | 0.74 ± 0.03 |  | 33556 | 29868 | 2 | 3686 | yes |  | abu-miR-27c |  | blast | uucacaguggcuaaguucguca | cagaacuuaguugacugcugacc | cagaacuuaguugacugcugaccagggagucuugaauauuuguucacaguggcuaaguucguca | NODE\_615561\_length\_2028\_cov\_24.085798:499..563:- |
| NODE\_327956\_length\_44928\_cov\_30.469461\_22424 | 1.6e+4 | 0.74 ± 0.03 |  | 32678 | 31707 | 0 | 971 | yes |  | abu-miR-135b |  | blast | uauggcuuuuuauuccuacguga | auguaggaguagaagccacu | uauggcuuuuuauuccuacgugaugguagauggguucauguaggaguagaagccacu | NODE\_327956\_length\_44928\_cov\_30.469461:24208..24265:+ |
| NODE\_74278\_length\_21387\_cov\_29.901529\_6137 | 1.5e+4 | 0.74 ± 0.03 |  | 31265 | 31248 | 0 | 17 | yes |  |  |  | blast | aaguacuccagcgucacuuuu | aagugacuuugguucacuuu | aagugacuuugguucacuuugauacgcaguuucuguuaaaguacuccagcgucacuuuu | NODE\_74278\_length\_21387\_cov\_29.901529:20291..20350:+ |
| NODE\_770820\_length\_34348\_cov\_28.646820\_44462 | 1.5e+4 | 0.74 ± 0.03 |  | 29866 | 28200 | 20 | 1646 | yes |  | abu-miR-8160a |  | blast | agaauaaugccagcagucggcc | ccagugcugguguuauugaga | agaauaaugccagcagucggccgugaacucagugucaggccagugcugguguuauugaga | NODE\_770820\_length\_34348\_cov\_28.646820:15374..15434:+ |
| NODE\_892061\_length\_20562\_cov\_28.993046\_47452 | 1.1e+4 | 0.74 ± 0.03 |  | 21717 | 21114 | 0 | 603 | yes |  | abu-miR-153a |  | blast | uugcauagucacaaaaaugauc | ucauucuugugguuugcagcu | ucauucuugugguuugcagcuaguagucuggguccaguugcauagucacaaaaaugauc | NODE\_892061\_length\_20562\_cov\_28.993046:14490..14549:- |
| NODE\_328984\_length\_2216\_cov\_22.881317\_22488 | 1.0e+4 | 0.74 ± 0.03 |  | 20811 | 18929 | 1775 | 107 | yes |  | abu-miR-449b-5p |  | blast | uggcagugucuuagcugguugu | caaucagcaaguauacugccgca | uggcagugucuuagcugguuguugugaggagugagaaugaagcaaucagcaaguauacugccgca | NODE\_328984\_length\_2216\_cov\_22.881317:22..87:+ |
| NODE\_177735\_length\_10371\_cov\_28.828175\_13423 | 7.7e+3 | 0.74 ± 0.03 |  | 15241 | 14056 | 0 | 1185 | no |  |  |  | blast | uagugcuuucuugagguguugc | ccacaucacaaggcauuacuu | uagugcuuucuugagguguugcaugcuauaugccacaucacaaggcauuacuu | NODE\_177735\_length\_10371\_cov\_28.828175:2759..2812:- |
| NODE\_160694\_length\_22589\_cov\_24.513391\_12255 | 7.1e+3 | 0.74 ± 0.03 |  | 13938 | 13557 | 0 | 381 | yes |  | abu-miR-196b |  | blast | uagguaguuucauguuguuggg | cugcaacaugaaacugucuua | uagguaguuucauguuguugggguccauuucuaacucugcaacaugaaacugucuua | NODE\_160694\_length\_22589\_cov\_24.513391:1085..1142:- |
| NODE\_929629\_length\_68456\_cov\_29.587706\_47901 | 6.5e+3 | 0.74 ± 0.03 |  | 12793 | 12655 | 0 | 138 | yes |  | dre-miR-194b |  | blast | uguaacagcaucuccauaugga | ccaguggagcugcuguuaucug | uguaacagcaucuccauauggaaaaacuguggcuuccaguggagcugcuguuaucug | NODE\_929629\_length\_68456\_cov\_29.587706:29412..29469:- |
| NODE\_179284\_length\_8141\_cov\_31.475740\_13558 | 6.2e+3 | 0.74 ± 0.03 |  | 12292 | 12055 | 2 | 235 | yes |  | ccr-miR-18b |  | blast | uaaggugcaucuaguguaguu | uacugcccuaguugccccuucu | uaaggugcaucuaguguaguuggugaaguauucaaagucuacugcccuaguugccccuucu | NODE\_179284\_length\_8141\_cov\_31.475740:7879..7940:+ |
| NODE\_749189\_length\_52450\_cov\_27.729570\_43726 | 6.2e+3 | 0.74 ± 0.03 |  | 12156 | 10328 | 88 | 1740 | yes |  | ccr-miR-18b |  | blast | uaaggugcaucuagugcagau | acugcccuaagugcuccuucu | uaaggugcaucuagugcagauagugaaauagacuagcaccuacugcccuaagugcuccuucu | NODE\_749189\_length\_52450\_cov\_27.729570:26981..27043:+ |
| NODE\_230079\_length\_33713\_cov\_29.460743\_16701 | 5.6e+3 | 0.74 ± 0.03 |  | 11005 | 8920 | 0 | 2085 | yes |  | gmo-miR-723-3p |  | blast | agacaucagauaaaucugugcu | ggcagcuuugugugaugugacu | ggcagcuuugugugaugugacuucugacauaucgaagaagacaucagauaaaucugugcu | NODE\_230079\_length\_33713\_cov\_29.460743:31660..31720:+ |
| NODE\_411932\_length\_653\_cov\_15.464012\_27046 | 4.5e+3 | 0.74 ± 0.03 |  | 8837 | 8204 | 14 | 619 | yes |  |  |  | blast | ucugugucgguggguuuagga | ugaccccaccugcacagaggg | ugaccccaccugcacagagggaguugugaacaugcacuucugugucgguggguuuagga | NODE\_411932\_length\_653\_cov\_15.464012:319..378:+ |
| NODE\_19525\_length\_4371\_cov\_18.886295\_1991 | 4.4e+3 | 0.74 ± 0.03 |  | 8815 | 8267 | 313 | 235 | yes |  |  |  | blast | augaaugacugucuccacgucu | caugaagacggucauucauugu | augaaugacugucuccacgucuguuuugucguccgcagacaugaagacggucauucauugu | NODE\_19525\_length\_4371\_cov\_18.886295:2919..2980:- |
| NODE\_562908\_length\_65\_cov\_106.000000\_35368 | 4.2e+3 | 0.74 ± 0.03 |  | 8338 | 7719 | 0 | 619 | yes |  |  |  | blast | ucugugucgguggguuuagga | ugaccccaccugcacagaggg | ugaccccaccugcacagagggaguugugaacauacacuucugugucgguggguuuagga | NODE\_562908\_length\_65\_cov\_106.000000:51..110:+ |
| NODE\_304900\_length\_11750\_cov\_23.285957\_21128 | 4.2e+3 | 0.74 ± 0.03 |  | 8328 | 8204 | 13 | 111 | yes |  |  |  | blast | ucugugucgguggguuuagga | cugacaccgccugcacagggag | cugacaccgccugcacagggaguugugaacaugcacuucugugucgguggguuuagga | NODE\_304900\_length\_11750\_cov\_23.285957:7627..7685:+ |
| NODE\_203663\_length\_65\_cov\_36.569229\_14999 | 4.0e+3 | 0.74 ± 0.03 |  | 7844 | 7719 | 0 | 125 | yes |  |  |  | blast | ucugugucgguggguuuagga | ugaccccaccugcgcagagg | ugaccccaccugcgcagagggaguugugaacauacacuucugugucgguggguuuagga | NODE\_203663\_length\_65\_cov\_36.569229:19..78:- |
| NODE\_412191\_length\_7299\_cov\_26.010550\_27073 | 3.4e+3 | 0.74 ± 0.03 |  | 6788 | 6787 | 0 | 1 | no |  |  |  | blast | ugcagaacuacggacgcagc | ugugcguguucauggagc | ugcagaacuacggacgcagcuggaauaacagcugugcguguucauggagc | NODE\_412191\_length\_7299\_cov\_26.010550:3686..3736:- |
| NODE\_385508\_length\_7829\_cov\_25.230169\_25774 | 3.4e+3 | 0.74 ± 0.03 |  | 6674 | 6672 | 0 | 2 | yes |  |  |  | blast | ugccccgcggagugugaguga | acucccacaccggggggucagcua | acucccacaccggggggucagcuaauccacucgcugccccgcggagugugaguga | NODE\_385508\_length\_7829\_cov\_25.230169:4733..4788:- |
| NODE\_319513\_length\_12777\_cov\_25.897472\_22022 | 3.2e+3 | 0.74 ± 0.03 |  | 6404 | 5872 | 26 | 506 | no |  |  |  | blast | cuacugugaugaccuguuugaacuc | cauuucaaacacugagu | cuacugugaugaccuguuugaacucucucacugaauaaaaacguugaagacagcauuucaaacacugagu | NODE\_319513\_length\_12777\_cov\_25.897472:3659..3729:+ |
| NODE\_817731\_length\_59985\_cov\_31.449245\_45827 | 3.0e+3 | 0.74 ± 0.03 |  | 5962 | 5946 | 0 | 16 | no |  |  |  | blast | ucaguacacuuguguuugaccu | agcagcacaaaguacuagugag | agcagcacaaaguacuagugagagcgugacucugaaagcucaguacacuuguguuugaccu | NODE\_817731\_length\_59985\_cov\_31.449245:57832..57893:+ |
| NODE\_88227\_length\_1190\_cov\_20.568068\_7196 | 2.8e+3 | 0.74 ± 0.03 |  | 5625 | 5223 | 0 | 402 | no |  | abu-miR-20a |  | blast | caaagugcucacagugcaggua | acugcaguuugugcacuugggg | caaagugcucacagugcagguaguaaacguucaucuacugcaguuugugcacuugggg | NODE\_88227\_length\_1190\_cov\_20.568068:362..420:+ |
| NODE\_1040002\_length\_1064\_cov\_7.121241\_48707 | 2.5e+3 | 0.74 ± 0.03 |  | 5006 | 4999 | 0 | 7 | no |  |  |  | blast | ugcaguagaacuugugag | ucacagacagaugcugaaaccaaa | ugcaguagaacuugugagugggauucuuucacagacagaugcugaaaccaaa | NODE\_1040002\_length\_1064\_cov\_7.121241:1090..1142:- |
| NODE\_860580\_length\_123\_cov\_140.268295\_46933 | 2.5e+3 | 0.74 ± 0.03 |  | 4940 | 4930 | 0 | 10 | no |  |  |  | blast | uacuguagguucaggaau | uuccaaucacuguauuucaau | uuccaaucacuguauuucaauggauccccacugaauacuguagguucaggaau | NODE\_860580\_length\_123\_cov\_140.268295:107..160:+ |
| NODE\_286223\_length\_123\_cov\_44.398373\_19993 | 2.5e+3 | 0.74 ± 0.03 |  | 4931 | 4930 | 0 | 1 | no |  |  |  | blast | uacuguagguucaggaau | uuccaaucacuguaugu | uuccaaucacuguaugucaauggauccccacugaauacuguagguucaggaau | NODE\_286223\_length\_123\_cov\_44.398373:107..160:+ |
| NODE\_815367\_length\_3683\_cov\_18.997284\_45719 | 2.1e+3 | 0.74 ± 0.03 |  | 4320 | 4283 | 3 | 34 | no |  |  |  | blast | uuagucgauacuucaugacccu | guucagaugucucggcgugga | uuagucgauacuucaugacccuguuuuucuguggcucagguucagaugucucggcgugga | NODE\_815367\_length\_3683\_cov\_18.997284:1993..2053:- |
| NODE\_603631\_length\_3071\_cov\_30.263107\_37327 | 2.1e+3 | 0.74 ± 0.03 |  | 4251 | 4244 | 0 | 7 | yes |  |  |  | blast | ugcagaacuacgagcgcagc | ugugcguguucauggagcu | ugcagaacuacgagcgcagcuggaauccacagcugugcguguucauggagcu | NODE\_603631\_length\_3071\_cov\_30.263107:2614..2666:- |
| NODE\_386074\_length\_22054\_cov\_23.038179\_25788 | 2.1e+3 | 0.74 ± 0.03 |  | 4251 | 4244 | 0 | 7 | no |  |  |  | blast | ugcagaacuacgagcgcagc | ugugcguguucauggagcu | ugcagaacuacgagcgcagcugaauccacagcugugcguguucauggagcu | NODE\_386074\_length\_22054\_cov\_23.038179:4412..4463:+ |
| NODE\_551018\_length\_29762\_cov\_27.452423\_34714 | 2.1e+3 | 0.74 ± 0.03 |  | 4218 | 2258 | 0 | 1960 | yes |  |  |  | blast | gcagagcucauauuaaacccu | aggguuuaauaugagcucugcc | gcagagcucauauuaaacccuauuaauaugagcuccauaggguuuaauaugagcucugcc | NODE\_551018\_length\_29762\_cov\_27.452423:1087..1147:+ |
| NODE\_551018\_length\_29762\_cov\_27.452423\_34715 | 2.1e+3 | 0.74 ± 0.03 |  | 4205 | 2245 | 0 | 1960 | yes |  |  |  | blast | gcagagcucauauuaaacccu | aggguuuaauaugagcucugcc | gcagagcucauauuaaacccuauggagcucauauuaauaggguuuaauaugagcucugcc | NODE\_551018\_length\_29762\_cov\_27.452423:1086..1146:- |
| NODE\_618453\_length\_89288\_cov\_30.092106\_38089 | 2.1e+3 | 0.74 ± 0.03 |  | 4120 | 2217 | 1 | 1902 | yes |  |  |  | blast | gcuuugacaauguugcacuacu | cagugcaauaguauugucauagc | gcuuugacaauguugcacuacuguaccauccauucuagcagugcaauaguauugucauagc | NODE\_618453\_length\_89288\_cov\_30.092106:89371..89432:- |
| NODE\_13725\_length\_16239\_cov\_24.088243\_1377 | 1.9e+3 | 0.74 ± 0.03 |  | 3865 | 3858 | 0 | 7 | no |  |  |  | blast | ugcagaacuacgaacggagc | ugugcguguucauggagcu | ugcagaacuacgaacggagcuggaauccauagcugugcguguucauggagcu | NODE\_13725\_length\_16239\_cov\_24.088243:7496..7548:- |
| NODE\_650421\_length\_14855\_cov\_31.549580\_39772 | 1.7e+3 | 0.74 ± 0.03 |  | 3435 | 3002 | 0 | 433 | yes |  | abu-miR-449b-5p |  | blast | aggcagugucuuguuagcuggu | cagcucaccugcugcugcccuc | aggcagugucuuguuagcugguaguuucuuccugugccagcucaccugcugcugcccuc | NODE\_650421\_length\_14855\_cov\_31.549580:9700..9759:+ |
| NODE\_272783\_length\_16124\_cov\_30.892210\_19222 | 1.4e+3 | 0.74 ± 0.03 |  | 2830 | 912 | 1917 | 1 | no |  |  |  | blast | gacuacggaucagaagauucuag | uggacaaauguuuguuu | uggacaaauguuuguuuagaugugaggcauggggccaguggcgcaauggauaacgcaucugacuacggaucagaagauucuag | NODE\_272783\_length\_16124\_cov\_30.892210:8742..8825:- |
| NODE\_133611\_length\_1490\_cov\_30.736914\_10273 | 1.2e+3 | 0.74 ± 0.03 |  | 2528 | 2432 | 87 | 9 | no |  |  |  | blast | uguuccagucugugguguugaagc | uacgaaccgguuuggugacu | uguuccagucugugguguugaagcaguccuguaaagccucugaagacccuucuggccacacuuuuaucugcuuacgaaccgguuuggugacu | NODE\_133611\_length\_1490\_cov\_30.736914:1385..1477:- |
| NODE\_408446\_length\_11063\_cov\_25.603271\_26862 | 1.2e+3 | 0.74 ± 0.03 |  | 2489 | 2297 | 1 | 191 | yes |  | ipu-miR-1788 |  | blast | ggcuuguuuuaaguugccuguga | caggcagcuaaagcaagccu | ggcuuguuuuaaguugccugugaauuuuaucuguuacacaggcagcuaaagcaagccu | NODE\_408446\_length\_11063\_cov\_25.603271:10991..11049:+ |
| NODE\_137989\_length\_3342\_cov\_24.688808\_10651 | 1.2e+3 | 0.74 ± 0.03 |  | 2449 | 2432 | 11 | 6 | no |  |  |  | blast | uguuccagucugugguguugaagc | uacgaaccgguuuggugacu | uguuccagucugugguguugaagcaguccuguaaagccucugaagacgcuucuggccacacuuuuaucugcuuacgaaccgguuuggugacu | NODE\_137989\_length\_3342\_cov\_24.688808:3038..3130:- |
| NODE\_290209\_length\_3102\_cov\_23.207930\_20220 | 1.2e+3 | 0.74 ± 0.03 |  | 2382 | 2341 | 33 | 8 | yes |  |  |  | blast | gcagagcuuuuaaugggc | uauagagcuacaccacugcaguccgg | uauagagcuacaccacugcaguccggauacagcccugcagagcuuuuaaugggc | NODE\_290209\_length\_3102\_cov\_23.207930:3016..3070:- |
| NODE\_300764\_length\_169\_cov\_195.562134\_20769 | 1.2e+3 | 0.74 ± 0.03 |  | 2382 | 2341 | 33 | 8 | yes |  |  |  | blast | gcagagcuuuuaaugggc | uauagagcuacaccacugcaguccgg | uauagagcuacaccacugcaguccggauacagcccugcagagcuuuuaaugggc | NODE\_300764\_length\_169\_cov\_195.562134:34..88:+ |
| NODE\_955713\_length\_2845\_cov\_12.417575\_48165 | 1.2e+3 | 0.74 ± 0.03 |  | 2382 | 2341 | 33 | 8 | no |  |  |  | blast | gcagagcuuuuaaugggc | uauagagcuacaccacugcaguccgg | uauagagcuacaccacugcaguccggauacagcccugcagagcuuuuaaugggc | NODE\_955713\_length\_2845\_cov\_12.417575:2522..2576:+ |
| NODE\_451635\_length\_3086\_cov\_17.013609\_29179 | 1.2e+3 | 0.74 ± 0.03 |  | 2382 | 2341 | 33 | 8 | no |  |  |  | blast | gcagagcuuuuaaugggc | uauagagcuacaccacugcaguccgg | uauagagcuacaccacugcaguccggauacagcccugcagagcuuuuaaugggc | NODE\_451635\_length\_3086\_cov\_17.013609:358..412:+ |
| NODE\_159984\_length\_18690\_cov\_29.799572\_12177 | 1.1e+3 | 0.74 ± 0.03 |  | 2328 | 2274 | 0 | 54 | yes |  |  |  | blast | caaaaaagauguccuguguccu | gacacaggagaucuuuugagc | caaaaaagauguccuguguccuccuguggggcaaaggacacaggagaucuuuugagc | NODE\_159984\_length\_18690\_cov\_29.799572:647..704:- |
| NODE\_268202\_length\_1240\_cov\_18.933065\_18927 | 9.7e+2 | 0.74 ± 0.03 |  | 1908 | 1253 | 0 | 655 | yes |  |  |  | blast | gaggccaauccaugacaagaacu | ucuugugugauuuggcauaccu | gaggccaauccaugacaagaacuuuuaguccaguucuugugugauuuggcauaccu | NODE\_268202\_length\_1240\_cov\_18.933065:526..582:- |
| NODE\_399057\_length\_674\_cov\_24.126112\_26387 | 9.0e+2 | 0.74 ± 0.03 |  | 1770 | 1769 | 0 | 1 | no |  | abu-miR-139 |  | blast | ucuacagugcaugugucu | agcaaagcaguggaaaa | ucuacagugcaugugucugcuuguuagaagcaaagcaguggaaaa | NODE\_399057\_length\_674\_cov\_24.126112:524..569:- |
| NODE\_22775\_length\_1267\_cov\_22.236780\_2170 | 8.8e+2 | 0.74 ± 0.03 |  | 1754 | 1010 | 0 | 744 | no |  |  |  | blast | cacccugaaccaaaaggacuac | uguuuuucuggauccaggguggg | uguuuuucuggauccagggugggugacuuguacagaggcacccugaaccaaaaggacuac | NODE\_22775\_length\_1267\_cov\_22.236780:485..545:- |
| NODE\_6163\_length\_4181\_cov\_61.480267\_675 | 8.3e+2 | 0.74 ± 0.03 |  | 1666 | 1500 | 0 | 166 | no |  |  |  | blast | ucgugauuguuccaaaugucccauu | ugguccauccuguguggcugucacaau | ugguccauccuguguggcugucacaauguucgugauuguuccaaaugucccauu | NODE\_6163\_length\_4181\_cov\_61.480267:99..153:+ |
| NODE\_317207\_length\_17788\_cov\_31.397797\_21792 | 7.7e+2 | 0.74 ± 0.03 |  | 1516 | 1490 | 0 | 26 | yes |  |  |  | blast | uaaaugaggauugcggcuugu | gagccgcaauccucguuuguug | gagccgcaauccucguuuguugugcaauuauguaaaauaaaugaggauugcggcuugu | NODE\_317207\_length\_17788\_cov\_31.397797:13036..13094:- |
| NODE\_134829\_length\_65\_cov\_40.461540\_10345 | 7.4e+2 | 0.74 ± 0.03 |  | 1476 | 1307 | 0 | 169 | no |  |  |  | blast | uaagugcaucugcuuggaccagu | ugguccgacucaugcaagaac | ugguccgacucaugcaagaacuugucucuugaauucaguaagugcaucugcuuggaccagu | NODE\_134829\_length\_65\_cov\_40.461540:58..119:+ |
| NODE\_699947\_length\_951\_cov\_16.044165\_41889 | 7.3e+2 | 0.74 ± 0.03 |  | 1436 | 1398 | 0 | 38 | yes |  |  |  | blast | uguuuuccaagcagaaccgucu | caguucugcuuggaaaacagugu | caguucugcuuggaaaacaguguugcaccacuguuuuccaagcagaaccgucu | NODE\_699947\_length\_951\_cov\_16.044165:463..516:+ |
| NODE\_6394\_length\_65\_cov\_538.215393\_747 | 6.8e+2 | 0.74 ± 0.03 |  | 1355 | 1194 | 159 | 2 | no |  |  |  | blast | uuucauguuaggcuucuucugggu | cuaggagggauagcuggagacagg | uuucauguuaggcuucuucuggguuaggccuuccuguuguucaugguggaagaugggugccuuuguugaccuaggagggauagcuggagacagg | NODE\_6394\_length\_65\_cov\_538.215393:10..104:- |
| NODE\_40695\_length\_11784\_cov\_28.186693\_3554 | 6.4e+2 | 0.74 ± 0.03 |  | 1267 | 793 | 0 | 474 | yes |  | ipu-miR-24 |  | blast | uggcucaguccagcaggaacaa | ugccuacugaacuggauucagu | ugccuacugaacuggauucagugugucuuugcagaacuggcucaguccagcaggaacaa | NODE\_40695\_length\_11784\_cov\_28.186693:4871..4930:- |
| NODE\_139951\_length\_15026\_cov\_24.779049\_10811 | 6.4e+2 | 0.74 ± 0.03 |  | 1273 | 1263 | 2 | 8 | yes |  |  |  | blast | aaaccagcugccuguucucugc | agagaccagagcaucugugauc | agagaccagagcaucugugaucucuggauauaaucugaaaccagcugccuguucucugc | NODE\_139951\_length\_15026\_cov\_24.779049:6493..6552:+ |
| NODE\_382880\_length\_18376\_cov\_32.528896\_25611 | 6.4e+2 | 0.74 ± 0.03 |  | 1267 | 831 | 0 | 436 | yes |  |  |  | blast | agcaccacugaugaacaauggg | guuguuugucaguagugcuagu | agcaccacugaugaacaaugggaacaauuacucgucagcccguuguuugucaguagugcuagu | NODE\_382880\_length\_18376\_cov\_32.528896:11215..11278:- |
| NODE\_400609\_length\_14557\_cov\_25.698084\_26479 | 6.4e+2 | 0.74 ± 0.03 |  | 1260 | 1059 | 1 | 200 | no |  | oni-miR-10805 |  | blast | uguuucuggugucuucuguagc | gucaggaggacaucugaua | gucaggaggacaucugauacaaacuggaaggacccagaauccauccugaggucugauggcaaccuuuuguuucuggugucuucuguagc | NODE\_400609\_length\_14557\_cov\_25.698084:13567..13656:+ |
| NODE\_78666\_length\_15103\_cov\_23.393896\_6407 | 6.1e+2 | 0.74 ± 0.03 |  | 1211 | 1206 | 0 | 5 | yes |  |  |  | blast | ucaucugaaacucuuucacagu | ugugaggugguuucaggacga | ucaucugaaacucuuucacaguuucuaccgugagcugugaggugguuucaggacga | NODE\_78666\_length\_15103\_cov\_23.393896:7143..7199:- |
| NODE\_246208\_length\_4696\_cov\_30.509369\_17760 | 5.8e+2 | 0.74 ± 0.03 |  | 1142 | 768 | 0 | 374 | yes |  |  |  | blast | cuuuauucaagggcaccucuga | agacgugcccuugaauaaaga | agacgugcccuugaauaaagagacugacauuuugucuuuauucaagggcaccucuga | NODE\_246208\_length\_4696\_cov\_30.509369:1559..1616:- |
| NODE\_60579\_length\_569\_cov\_224.813705\_5090 | 5.5e+2 | 0.74 ± 0.03 |  | 1108 | 1104 | 2 | 2 | no |  |  |  | blast | cuccuggcuggcucgcca | uguugugagcuggaaagucaagg | uguugugagcuggaaagucaaggugcuacuuuucaccaaucuccuggcuggcucgcca | NODE\_60579\_length\_569\_cov\_224.813705:28..86:- |
| NODE\_375056\_length\_2485\_cov\_16.542456\_25178 | 5.5e+2 | 0.74 ± 0.03 |  | 1113 | 1092 | 0 | 21 | no |  |  |  | blast | ugacaaugcaguucugaagaaagac | uuuucuugaaguauguaggguaagu | ugacaaugcaguucugaagaaagacaaguguuuucuugaaguauguaggguaagu | NODE\_375056\_length\_2485\_cov\_16.542456:18..73:+ |
| NODE\_313014\_length\_13774\_cov\_29.902571\_21537 | 5.5e+2 | 0.74 ± 0.03 |  | 1087 | 1082 | 0 | 5 | yes |  |  |  | blast | uauuguaucagcagacugucu | gccugaaacugaugcaacaac | uauuguaucagcagacugucuaugcuguuucuccguguagccugaaacugaugcaacaac | NODE\_313014\_length\_13774\_cov\_29.902571:3419..3479:- |
| NODE\_287166\_length\_26153\_cov\_24.486980\_20029 | 5.5e+2 | 0.74 ± 0.03 |  | 1088 | 1032 | 6 | 50 | yes |  |  |  | blast | guccagugauuauaacucuucugc | cuaugauauaauaugaggaca | guccagugauuauaacucuucugcuguucacauccugacacguugugauugcacuaugauauaauaugaggaca | NODE\_287166\_length\_26153\_cov\_24.486980:24076..24150:- |
| NODE\_433245\_length\_65\_cov\_78.753845\_28117 | 5.3e+2 | 0.74 ± 0.03 |  | 1072 | 1057 | 3 | 12 | no |  |  |  | blast | ugagacaaagcuguguggaaggc | uagcauaauguucuguuuuagcu | ugagacaaagcuguguggaaggcauuagccauguugcucggugauagcauaauguucuguuuuagcu | NODE\_433245\_length\_65\_cov\_78.753845:60..127:- |
| NODE\_538217\_length\_17757\_cov\_28.332264\_34185 | 5.2e+2 | 0.74 ± 0.03 |  | 1026 | 696 | 0 | 330 | yes |  |  |  | blast | accuucgcccaccugucugag | ucagacaguuuggcgaagguuu | accuucgcccaccugucugagagugauugcaaugagucucagacaguuuggcgaagguuu | NODE\_538217\_length\_17757\_cov\_28.332264:11499..11559:- |
| NODE\_398500\_length\_63967\_cov\_28.420389\_26369 | 5.2e+2 | 0.74 ± 0.03 |  | 1023 | 693 | 0 | 330 | yes |  |  |  | blast | aauacaacgcgaagaaauaugc | auauuucuucgcguuguguuuu | aauacaacgcgaagaaauaugcacuaguagugcauauuucuucgcguuguguuuu | NODE\_398500\_length\_63967\_cov\_28.420389:15396..15451:- |
| NODE\_287161\_length\_41284\_cov\_34.058327\_20026 | 5.1e+2 | 0.74 ± 0.03 |  | 1022 | 1006 | 2 | 14 | yes |  |  |  | blast | ucugaauggaugcaaacugcauu | uaaaguugaauucuauu | ucugaauggaugcaaacugcauuucguugccuuguacuuuugacaugugcaaugaaaauaaaguugaauucuauu | NODE\_287161\_length\_41284\_cov\_34.058327:25601..25676:+ |
| NODE\_89362\_length\_7947\_cov\_25.921103\_7310 | 5.1e+2 | 0.74 ± 0.03 |  | 997 | 983 | 0 | 14 | yes |  |  |  | blast | uggaggacaccugauaccugg | cagguaucacgguguccuccacc | cagguaucacgguguccuccaccguacagguggaggacaccugauaccugg | NODE\_89362\_length\_7947\_cov\_25.921103:439..490:- |
| NODE\_501079\_length\_20749\_cov\_31.936527\_32047 | 4.9e+2 | 0.74 ± 0.03 |  | 968 | 835 | 0 | 133 | yes |  | oni-miR-10610c |  | blast | aaagcauugcucuaccugcacu | ugcagauggagcgcugcugacu | aaagcauugcucuaccugcacuguguauagugcagauggagcgcugcugacu | NODE\_501079\_length\_20749\_cov\_31.936527:19094..19146:- |
| NODE\_60594\_length\_1916\_cov\_171.119003\_5100 | 4.9e+2 | 0.74 ± 0.03 |  | 986 | 842 | 44 | 100 | no |  |  |  | blast | uugucuagaugcugcuguacacu | ugugaaugcugcuucuug | ugugaaugcugcuucuugugggucccugagcaagaagguagggcugacugcuucgggugauguugucuagaugcugcuguacacu | NODE\_60594\_length\_1916\_cov\_171.119003:10..95:- |
| NODE\_732741\_length\_70032\_cov\_29.792580\_43245 | 4.7e+2 | 0.74 ± 0.03 |  | 935 | 904 | 0 | 31 | yes |  |  |  | blast | auuuauagucaacuacucaga | aggguaguugacuauaaaugc | aggguaguugacuauaaaugcaaugaguccagggcauuuauagucaacuacucaga | NODE\_732741\_length\_70032\_cov\_29.792580:5487..5543:+ |
| NODE\_551141\_length\_75190\_cov\_29.979904\_34729 | 4.7e+2 | 0.74 ± 0.03 |  | 932 | 931 | 0 | 1 | yes |  |  |  | blast | acaacaggagcugaagcugcgu | guugcuucuguuccuguuucu | guugcuucuguuccuguuucugguggaguuaccacaacaggagcugaagcugcgu | NODE\_551141\_length\_75190\_cov\_29.979904:73682..73737:+ |
| NODE\_925763\_length\_33856\_cov\_29.179258\_47855 | 4.6e+2 | 0.74 ± 0.03 |  | 923 | 916 | 0 | 7 | no |  |  |  | blast | ucccgcaucagugauuuuaa | ccuuggcucgugugauu | ccuuggcucgugugauuaggauuaugguuuucuuauaucccgcaucagugauuuuaa | NODE\_925763\_length\_33856\_cov\_29.179258:30786..30843:+ |
| NODE\_468625\_length\_75\_cov\_680.386658\_30066 | 4.3e+2 | 0.74 ± 0.03 |  | 850 | 359 | 0 | 491 | yes |  |  |  | blast | cucuacacugacaguacacugu | acuauacuguugguaugaaggu | cucuacacugacaguacacugucuuuauauuagacacuauacuguugguaugaaggu | NODE\_468625\_length\_75\_cov\_680.386658:75..132:- |
| NODE\_283852\_length\_157\_cov\_125.484077\_19807 | 4.2e+2 | 0.74 ± 0.03 |  | 843 | 842 | 0 | 1 | yes |  |  |  | blast | uugucuagaugcugcuguacacu | aaacagcgguauuuagu | uugucuagaugcugcuguacacugguuccagccuacaacaacacauuaaacagcgguauuuagu | NODE\_283852\_length\_157\_cov\_125.484077:152..216:- |
| NODE\_844612\_length\_8524\_cov\_21.723721\_46573 | 4.1e+2 | 0.74 ± 0.03 |  | 815 | 786 | 0 | 29 | yes |  |  |  | blast | cggaccccaaagcacucacucu | agugaggaaccuggggcccgau | cggaccccaaagcacucacucuggucaaaucaucagcagagugaggaaccuggggcccgau | NODE\_844612\_length\_8524\_cov\_21.723721:3110..3171:- |
| NODE\_618044\_length\_26971\_cov\_25.920063\_38053 | 4.0e+2 | 0.74 ± 0.03 |  | 784 | 782 | 0 | 2 | yes |  |  |  | blast | uaacagcguaccgacuccagc | ugcagucauuaugcuguuaca | ugcagucauuaugcuguuacauacauuuguuguuguaacagcguaccgacuccagc | NODE\_618044\_length\_26971\_cov\_25.920063:5919..5975:+ |
| NODE\_335010\_length\_17137\_cov\_28.842737\_22903 | 4.0e+2 | 0.74 ± 0.03 |  | 783 | 746 | 0 | 37 | yes |  |  |  | blast | ccucugucucccccugccuuugu | aaagggagagagagacgguguaga | ccucugucucccccugccuuugugugcgagaggccagcaaagggagagagagacgguguaga | NODE\_335010\_length\_17137\_cov\_28.842737:16968..17030:- |
| NODE\_211386\_length\_14784\_cov\_30.715910\_15551 | 3.9e+2 | 0.74 ± 0.03 |  | 774 | 696 | 0 | 78 | yes |  | abu-miR-736 |  | blast | guaagacgaacaaaaaguuugu | cagcuuuuuguuuguauuaugu | cagcuuuuuguuuguauuauguuccuacuaaaauguaagacgaacaaaaaguuugu | NODE\_211386\_length\_14784\_cov\_30.715910:2220..2276:+ |
| NODE\_259338\_length\_3754\_cov\_21.700054\_18397 | 3.9e+2 | 0.74 ± 0.03 |  | 773 | 476 | 19 | 278 | yes |  |  |  | blast | uggguccagccuguguccaguu | uaguuccagccuguguccaguu | uaguuccagccuguguccaguuugaguccagucuggguccagucuggguccagccuggguccagccuguguccaguu | NODE\_259338\_length\_3754\_cov\_21.700054:3359..3436:- |
| NODE\_244240\_length\_103669\_cov\_30.213709\_17530 | 3.6e+2 | 0.74 ± 0.03 |  | 727 | 714 | 0 | 13 | no |  |  |  | blast | cagaagagauugugcacugagu | ucggcgcacaauguugaagcu | ucggcgcacaauguugaagcugcuguugaguuuguagcagaagagauugugcacugagu | NODE\_244240\_length\_103669\_cov\_30.213709:25362..25421:- |
| NODE\_43176\_length\_130\_cov\_201.530762\_3749 | 3.5e+2 | 0.74 ± 0.03 |  | 694 | 693 | 0 | 1 | yes |  | eel-miR-11053-5p |  | blast | uccaugagugggguaaccugaaca | ucagccucuugguggagcucugg | uccaugagugggguaaccugaacacuuuguucagccucuugguggagcucugg | NODE\_43176\_length\_130\_cov\_201.530762:92..145:- |
| NODE\_487237\_length\_926\_cov\_72.533478\_31050 | 3.5e+2 | 0.74 ± 0.03 |  | 692 | 521 | 0 | 171 | yes |  | abu-miR-15a |  | blast | gagcagcacccugagccucucu | gcgggcucagugagcugugagg | gagcagcacccugagccucucuguagaccaguggaagaaggcgggcucagugagcugugagg | NODE\_487237\_length\_926\_cov\_72.533478:26..88:- |
| NODE\_523764\_length\_5629\_cov\_25.588203\_33446 | 3.4e+2 | 0.74 ± 0.03 |  | 681 | 586 | 0 | 95 | yes |  |  |  | blast | acaagccgaaaugcguugguca | ccaucauguuuuggcuucuggc | ccaucauguuuuggcuucuggccuucaucagggucauggccacaagccgaaaugcguugguca | NODE\_523764\_length\_5629\_cov\_25.588203:1900..1963:- |
| NODE\_618044\_length\_26971\_cov\_25.920063\_38056 | 3.4e+2 | 0.74 ± 0.03 |  | 671 | 482 | 0 | 189 | yes |  |  |  | blast | uaacagcauaaugacugcagucc | gcuggagucgguacgcuguuac | gcuggagucgguacgcuguuacaacaacaaauguauguaacagcauaaugacugcagucc | NODE\_618044\_length\_26971\_cov\_25.920063:5915..5975:- |
| NODE\_273105\_length\_6672\_cov\_26.730215\_19240 | 3.3e+2 | 0.74 ± 0.03 |  | 653 | 566 | 0 | 87 | yes |  |  |  | blast | cgaugcaaguccugagucugagc | cagccccaucacuugccucaga | cgaugcaaguccugagucugagcuucguucucagccccaucacuugccucaga | NODE\_273105\_length\_6672\_cov\_26.730215:6163..6216:+ |
| NODE\_1101960\_length\_65\_cov\_124.676926\_48943 | 3.3e+2 | 0.74 ± 0.03 |  | 662 | 633 | 19 | 10 | no |  |  |  | blast | uuugguagaagcuugguaacacc | gugacaaccaacuagcaccccucuca | gugacaaccaacuagcaccccucucaggaagcucuaggcaagguuacccgccagacuucgguuugguagaagcuugguaacacc | NODE\_1101960\_length\_65\_cov\_124.676926:42..126:+ |
| NODE\_213520\_length\_329\_cov\_14.884499\_15743 | 3.2e+2 | 0.74 ± 0.03 |  | 644 | 639 | 0 | 5 | no |  |  |  | blast | uugugaguccuugaggagcaggc | ccaaccucggcuccuuuau | uugugaguccuugaggagcaggcaugcguucgcagcaugccaaccucggcuccuuuau | NODE\_213520\_length\_329\_cov\_14.884499:240..298:- |
| NODE\_81486\_length\_39672\_cov\_31.367388\_6631 | 3.0e+2 | 0.74 ± 0.03 |  | 585 | 550 | 0 | 35 | yes |  | ola-miR-133-5p |  | blast | ugcugguacuguucuacuuccu | gagcuggaccauuacagcacc | ugcugguacuguucuacuuccucugcuacauauacccagagcuggaccauuacagcacc | NODE\_81486\_length\_39672\_cov\_31.367388:3788..3847:- |
| NODE\_319315\_length\_10503\_cov\_30.863943\_21996 | 2.8e+2 | 0.74 ± 0.03 |  | 556 | 544 | 3 | 9 | yes |  |  |  | blast | uuguaagaaugcagugugaacaagc | ugcacuguuaauuuagc | uuguaagaaugcagugugaacaagcugcagcuuauaaacuguugacuuaaucaacagguuuguuugcacuguuaauuuagc | NODE\_319315\_length\_10503\_cov\_30.863943:10039..10120:+ |
| NODE\_370392\_length\_51393\_cov\_26.036484\_24772 | 2.6e+2 | 0.74 ± 0.03 |  | 530 | 526 | 0 | 4 | yes |  |  |  | blast | aaugcuguuguuuuaauaaucu | uuauuaaacagcaguauaca | aaugcuguuguuuuaauaaucuguagaaaaauaagccugcuuauuaaacagcaguauaca | NODE\_370392\_length\_51393\_cov\_26.036484:23321..23381:+ |
| NODE\_338688\_length\_220\_cov\_73.845451\_22982 | 2.6e+2 | 0.74 ± 0.03 |  | 527 | 519 | 0 | 8 | yes |  |  |  | blast | gaggaucucauuguugaagucaaga | ucagacuucaguuuauggau | gaggaucucauuguugaagucaagagauaguguugucuucagggcaguuucagacuucaguuuauggau | NODE\_338688\_length\_220\_cov\_73.845451:192..261:+ |
| NODE\_365572\_length\_2072\_cov\_21.078669\_24504 | 2.6e+2 | 0.74 ± 0.03 |  | 517 | 502 | 0 | 15 | yes |  |  |  | blast | ugaaggccugacuguggcaaccu | uugccacuacaggccuucaguu | uugccacuacaggccuucaguuucauacaggagaaacugaaggccugacuguggcaaccu | NODE\_365572\_length\_2072\_cov\_21.078669:129..189:- |
| NODE\_276524\_length\_31568\_cov\_29.318520\_19489 | 2.6e+2 | 0.74 ± 0.03 |  | 520 | 519 | 0 | 1 | no |  |  |  | blast | cauccuguuuuaguacuaugcaccu | uguguggcuaugcuaacagga | cauccuguuuuaguacuaugcaccugcuucuuauuacggguguguggcuaugcuaacagga | NODE\_276524\_length\_31568\_cov\_29.318520:19241..19302:- |
| NODE\_27951\_length\_3778\_cov\_35.373741\_2628 | 2.4e+2 | 0.74 ± 0.03 |  | 504 | 503 | 0 | 1 | no |  |  |  | blast | auccacuguagugaccccuaaagg | acgaggaggcagcuauggauag | acgaggaggcagcuauggauaggauaagauggaggcagaugauccacuguagugaccccuaaagg | NODE\_27951\_length\_3778\_cov\_35.373741:2471..2536:- |
| NODE\_214485\_length\_74\_cov\_2322.567627\_15853 | 2.4e+2 | 0.74 ± 0.03 |  | 510 | 385 | 110 | 15 | no |  |  |  | blast | uuaagguuaggauuugguugcggg | ugagguggggucugaacuguggg | ugagguggggucugaacuguggguaccgguaugaggagagaagggggacaggacuggaugguggguuaagguuaggauuugguugcggg | NODE\_214485\_length\_74\_cov\_2322.567627:46..135:+ |
| NODE\_904529\_length\_12541\_cov\_25.146160\_47617 | 2.3e+2 | 0.74 ± 0.03 |  | 468 | 402 | 14 | 52 | yes |  |  |  | blast | ucuguaucgguggguuuagga | ugacuccgccugcacagaggga | ugacuccgccugcacagagggaguugugaacaugcacuucuguaucgguggguuuagga | NODE\_904529\_length\_12541\_cov\_25.146160:3786..3845:+ |
| NODE\_317154\_length\_46243\_cov\_32.842659\_21782 | 2.3e+2 | 0.74 ± 0.03 |  | 460 | 434 | 0 | 26 | yes |  | abu-miR-148b |  | blast | uuaguucuuaguuacucugacu | ucagugagcuaagaaccuagacu | uuaguucuuaguuacucugacuuacugccuacuuguuaauauagucagugagcuaagaaccuagacu | NODE\_317154\_length\_46243\_cov\_32.842659:23885..23952:+ |
| NODE\_5441\_length\_25439\_cov\_31.317701\_614 | 2.3e+2 | 0.74 ± 0.03 |  | 465 | 191 | 0 | 274 | yes |  |  |  | blast | cagguuguccuguuacucuuu | caaggguaacaggacaaccugu | caaggguaacaggacaaccugucuaaagacaaacagguuguccuguuacucuuu | NODE\_5441\_length\_25439\_cov\_31.317701:14431..14485:- |
| NODE\_346978\_length\_68096\_cov\_30.827200\_23449 | 2.2e+2 | 0.74 ± 0.03 |  | 436 | 380 | 0 | 56 | yes |  |  |  | blast | gaggacaggaccgggauuccga | cagaauggcgagucguccucc | gaggacaggaccgggauuccgagcucuccugucgaaacgguacucagaauggcgagucguccucc | NODE\_346978\_length\_68096\_cov\_30.827200:44007..44072:- |
| NODE\_915340\_length\_10018\_cov\_24.630964\_47785 | 2.1e+2 | 0.74 ± 0.03 |  | 440 | 439 | 0 | 1 | no |  |  |  | blast | guuuucuuuucuucuuaaa | uaggaugacgggacagagcu | uaggaugacgggacagagcucuuuguuuucuuuucuucuuaaa | NODE\_915340\_length\_10018\_cov\_24.630964:7679..7722:+ |
| NODE\_239826\_length\_7731\_cov\_32.569782\_17180 | 2.1e+2 | 0.74 ± 0.03 |  | 419 | 404 | 0 | 15 | yes |  |  |  | blast | augucacucuuaaagcaucugu | agcugcaguuugaauggcauuu | augucacucuuaaagcaucuguuccguguuacagcugcaguuugaauggcauuu | NODE\_239826\_length\_7731\_cov\_32.569782:7307..7361:+ |
| NODE\_36421\_length\_1398\_cov\_16.993563\_3257 | 2.0e+2 | 0.74 ± 0.03 |  | 408 | 216 | 1 | 191 | yes |  |  |  | blast | gacaccgcagacucagagcauu | uguucugggacuguggaugcucu | uguucugggacuguggaugcucuggaaaaacaaacaaacaaacaaacagacaccgcagacucagagcauu | NODE\_36421\_length\_1398\_cov\_16.993563:1165..1235:+ |
| NODE\_478880\_length\_167\_cov\_45.562874\_30784 | 2.0e+2 | 0.74 ± 0.03 |  | 407 | 404 | 0 | 3 | yes |  |  |  | blast | acaacaugcgucucaguccuca | agggcucagggccguccuguugu | agggcucagggccguccuguugucaugguaucacaugcaugacaacaugcgucucaguccuca | NODE\_478880\_length\_167\_cov\_45.562874:156..219:- |
| NODE\_311231\_length\_74\_cov\_50.121620\_21413 | 2.0e+2 | 0.74 ± 0.03 |  | 433 | 432 | 0 | 1 | no |  |  |  | blast | uuguauaugaaaaagugcuuuggc | caacagguuucuauuaa | caacagguuucuauuaaauguuguaauuuguauaugaaaaagugcuuuggc | NODE\_311231\_length\_74\_cov\_50.121620:10..61:+ |
| NODE\_153899\_length\_3879\_cov\_23.442383\_11678 | 1.9e+2 | 0.74 ± 0.03 |  | 394 | 378 | 0 | 16 | yes |  |  |  | blast | gcggcugagagaacaacuccugc | ccgugguuacaucucagccaaca | gcggcugagagaacaacuccugccauuagccggccgugguuacaucucagccaaca | NODE\_153899\_length\_3879\_cov\_23.442383:2506..2562:+ |
| NODE\_881979\_length\_25202\_cov\_30.581581\_47327 | 1.9e+2 | 0.74 ± 0.03 |  | 381 | 323 | 0 | 58 | yes |  | oni-miR-10580 |  | blast | cacggagcaagacugcagauucu | cauucugcagcuuugcucugua | cacggagcaagacugcagauucucugcuugugugccaugugcagcauucugcagcuuugcucugua | NODE\_881979\_length\_25202\_cov\_30.581581:3144..3210:- |
| NODE\_571662\_length\_359\_cov\_46.080780\_35916 | 1.9e+2 | 0.74 ± 0.03 |  | 393 | 379 | 3 | 11 | no |  |  |  | blast | uauccagguaagcugagacugcuuc | gcacagaucagcaguccgcuggagc | gcacagaucagcaguccgcuggagccaccgagggugagcgcggccccgcccuuugagguacguuauccagguaagcugagacugcuuc | NODE\_571662\_length\_359\_cov\_46.080780:152..240:- |
| NODE\_994988\_length\_14518\_cov\_26.038160\_48463 | 1.9e+2 | 0.74 ± 0.03 |  | 377 | 366 | 0 | 11 | yes |  | oni-miR-10756 |  | blast | cauuuccugugcucauuggcc | accagugagcuguaaggaaauuua | cauuuccugugcucauuggccauuccucccaaaugaccagugagcuguaaggaaauuua | NODE\_994988\_length\_14518\_cov\_26.038160:10887..10946:- |
| NODE\_679492\_length\_65\_cov\_18.369230\_41057 | 1.9e+2 | 0.74 ± 0.03 |  | 418 | 412 | 3 | 3 | yes |  |  |  | blast | ugugaacagaucuugaagaaaaaa | ucuuucauucaaaccacuuuaca | ugugaacagaucuugaagaaaaaaaagaaaacaaaacauacuuuucuuuucucuuucauucaaaccacuuuaca | NODE\_679492\_length\_65\_cov\_18.369230:17..91:- |
| NODE\_73580\_length\_6074\_cov\_21.501646\_6072 | 1.9e+2 | 0.74 ± 0.03 |  | 375 | 356 | 0 | 19 | yes |  |  |  | blast | uagacacuuucuucccucgcu | ggacgggacgggaugugucuga | ggacgggacgggaugugucugaagguugccugauuuuuagacacuuucuucccucgcu | NODE\_73580\_length\_6074\_cov\_21.501646:3331..3389:- |
| NODE\_687041\_length\_11553\_cov\_26.289968\_41446 | 1.8e+2 | 0.74 ± 0.03 |  | 369 | 143 | 11 | 215 | yes |  |  |  | blast | ccugcucucugaugaccgcggc | cacggacagauacggagcaga | ccugcucucugaugaccgcggcgaauugagagaaaucgccacggacagauacggagcaga | NODE\_687041\_length\_11553\_cov\_26.289968:6740..6800:+ |
| NODE\_366217\_length\_12078\_cov\_29.766022\_24531 | 1.8e+2 | 0.74 ± 0.03 |  | 391 | 381 | 0 | 10 | no |  |  |  | blast | aaagggaggacuugaacc | gaaacgucuucaagaaacuuaa | gaaacgucuucaagaaacuuaaaaacauuaaaagugaugaaaggaaagggaggacuugaacc | NODE\_366217\_length\_12078\_cov\_29.766022:5300..5362:+ |
| NODE\_87057\_length\_3885\_cov\_27.354441\_7028 | 1.8e+2 | 0.74 ± 0.03 |  | 389 | 387 | 0 | 2 | no |  |  |  | blast | gcagaagggcaaaagcuc | aacuuuggccauuuauau | gcagaagggcaaaagcucacccagaacuuuggccauuuauau | NODE\_87057\_length\_3885\_cov\_27.354441:1484..1526:+ |
| NODE\_148886\_length\_3948\_cov\_20.033941\_11375 | 1.8e+2 | 0.74 ± 0.03 |  | 366 | 126 | 0 | 240 | no |  |  |  | blast | uuucccucugcugaugaaacc | aaucagcagcacgagguaaacac | uuucccucugcugaugaaacccgcugcagcuuucuccuggaaucagcagcacgagguaaacac | NODE\_148886\_length\_3948\_cov\_20.033941:2032..2095:+ |
| NODE\_629987\_length\_5573\_cov\_20.851965\_38886 | 1.8e+2 | 0.74 ± 0.03 |  | 399 | 123 | 4 | 272 | no |  |  |  | blast | uagaaauggcuucauguuacaaaca | uagugauacaaaaacugcauuuu | uagugauacaaaaacugcauuuuuuuuuuaauuaaugaaaaaauaaauaaaaauagaaauggcuucauguuacaaaca | NODE\_629987\_length\_5573\_cov\_20.851965:2977..3055:- |
| NODE\_661702\_length\_31525\_cov\_26.808311\_40380 | 1.7e+2 | 0.74 ± 0.03 |  | 327 | 326 | 0 | 1 | yes |  | ccr-miR-124b |  | blast | ucaagguccacugugaacacu | cauucaccgcgugccuuaau | cauucaccgcgugccuuaauugacugauuuuaaaucaagguccacugugaacacu | NODE\_661702\_length\_31525\_cov\_26.808311:4720..4775:- |
| NODE\_166587\_length\_156\_cov\_427.692322\_12628 | 1.6e+2 | 0.74 ± 0.03 |  | 338 | 121 | 215 | 2 | no |  |  |  | blast | uuggcgucucgggaguaaaagacc | ccagcugcagagaggagauaaaggg | uuggcgucucgggaguaaaagaccgugcagaugucuuuggugauaauggcaggcuggaugcaaugguccagcugcagagaggagauaaaggg | NODE\_166587\_length\_156\_cov\_427.692322:0..92:+ |
| NODE\_211784\_length\_7340\_cov\_29.774523\_15604 | 1.6e+2 | 0.74 ± 0.03 |  | 334 | 250 | 0 | 84 | no |  |  |  | blast | acgugaauggcgaucucuaugu | uaaagauaucaacauucauaca | acgugaauggcgaucucuauguugcuugcugcccgagcaguaaagauaucaacauucauaca | NODE\_211784\_length\_7340\_cov\_29.774523:4503..4565:+ |
| NODE\_5057\_length\_10758\_cov\_28.897379\_537 | 1.6e+2 | 0.74 ± 0.03 |  | 310 | 307 | 0 | 3 | yes |  | gmo-miR-11211-3p |  | blast | uuugguaccacagucuccgcu | caggacugugguaccaaaccc | caggacugugguaccaaacccauccagcagguuugguaccacagucuccgcu | NODE\_5057\_length\_10758\_cov\_28.897379:8113..8165:+ |
| NODE\_413592\_length\_23125\_cov\_26.332756\_27198 | 1.5e+2 | 0.74 ± 0.03 |  | 312 | 309 | 1 | 2 | yes |  |  |  | blast | uggaugagaauuuauggccugaca | cacgccguuucuucccucgc | cacgccguuucuucccucgcugcaucgcaagacaaaauaugugugcgaugcaauguggaugagaauuuauggccugaca | NODE\_413592\_length\_23125\_cov\_26.332756:23358..23437:- |
| NODE\_450831\_length\_46186\_cov\_34.881611\_29134 | 1.5e+2 | 0.74 ± 0.03 |  | 300 | 189 | 0 | 111 | yes |  |  |  | blast | uucuuuugggagaagcagcagg | cgcugcuucucccaaaaguacu | cgcugcuucucccaaaaguacugaagcagcaauguucuuuugggagaagcagcagg | NODE\_450831\_length\_46186\_cov\_34.881611:28221..28277:- |
| NODE\_124732\_length\_231\_cov\_53.593075\_9742 | 1.5e+2 | 0.74 ± 0.03 |  | 300 | 136 | 1 | 163 | yes |  |  |  | blast | uuuccagaguguauagucucgc | gagacuauacuuucagggauc | gagacuauacuuucagggaucauuucuauaggcuuugccuugagaaauguguuuccagaguguauagucucgc | NODE\_124732\_length\_231\_cov\_53.593075:223..296:- |
| NODE\_12085\_length\_17489\_cov\_31.685802\_1227 | 1.5e+2 | 0.74 ± 0.03 |  | 288 | 192 | 0 | 96 | yes |  | abu-miR-193-5p |  | blast | agggucuuugaggaugugcuga | gcaguuguccccaaagaucagc | agggucuuugaggaugugcugauugggagcaugacagcagcaguuguccccaaagaucagc | NODE\_12085\_length\_17489\_cov\_31.685802:11413..11474:- |
| NODE\_99952\_length\_8907\_cov\_29.034130\_7978 | 1.4e+2 | 0.74 ± 0.03 |  | 290 | 162 | 0 | 128 | yes |  |  |  | blast | aacagcaagaaucucaacaagu | uuguugagauucuugcuguucu | aacagcaagaaucucaacaaguuuauuugguucaacuuguugagauucuugcuguucu | NODE\_99952\_length\_8907\_cov\_29.034130:7867..7925:+ |
| NODE\_479273\_length\_340\_cov\_704.655884\_30809 | 1.4e+2 | 0.74 ± 0.03 |  | 286 | 280 | 0 | 6 | yes |  |  |  | blast | cacugcuggcgcugauacuuu | aggugucagcgcgagcaguggc | aggugucagcgcgagcaguggcggagcgaguguuaaagccacugcuggcgcugauacuuu | NODE\_479273\_length\_340\_cov\_704.655884:333..393:+ |
| NODE\_99952\_length\_8907\_cov\_29.034130\_7979 | 1.4e+2 | 0.74 ± 0.03 |  | 286 | 158 | 0 | 128 | yes |  |  |  | blast | aacagcaagaaucucaacaagu | uuguugagauucuugcuguucu | aacagcaagaaucucaacaaguugaaccaaauaaacuuguugagauucuugcuguucu | NODE\_99952\_length\_8907\_cov\_29.034130:7865..7923:- |
| NODE\_528829\_length\_28753\_cov\_28.107502\_33685 | 1.4e+2 | 0.74 ± 0.03 |  | 303 | 301 | 0 | 2 | no |  |  |  | blast | ggcuugcuccguccacu | cugguauugcaguaguuc | ggcuugcuccguccacuccacacaugaaucugguauugcaguaguuc | NODE\_528829\_length\_28753\_cov\_28.107502:24155..24202:- |
| NODE\_659373\_length\_5508\_cov\_21.989288\_40198 | 1.4e+2 | 0.74 ± 0.03 |  | 285 | 278 | 0 | 7 | yes |  |  |  | blast | ugcagaacuacgaagcagc | ugugcguguucauggagcu | ugcagaacuacgaagcagcuggaauccacagcugugcguguucauggagcu | NODE\_659373\_length\_5508\_cov\_21.989288:3606..3657:+ |
| NODE\_487806\_length\_675\_cov\_12.234074\_31076 | 1.4e+2 | 0.74 ± 0.03 |  | 284 | 278 | 0 | 6 | no |  |  |  | blast | ugcagaacuacgaagcagc | ugugcguguucauggagcu | ugcagaacuacgaagcagcuggaauccacagcugugcguguucauggagcu | NODE\_487806\_length\_675\_cov\_12.234074:510..561:+ |
| NODE\_762842\_length\_28848\_cov\_26.993587\_44166 | 1.4e+2 | 0.74 ± 0.03 |  | 286 | 285 | 0 | 1 | no |  |  |  | blast | uuugaucaaagcaaggccccgagc | ucgaggagcuucuuccaccgagc | ucgaggagcuucuuccaccgagcccacuuugaucaaagcaaggccccgagc | NODE\_762842\_length\_28848\_cov\_26.993587:13413..13464:- |
| NODE\_478161\_length\_16258\_cov\_25.415857\_30693 | 1.3e+2 | 0.74 ± 0.03 |  | 278 | 277 | 0 | 1 | no |  |  |  | blast | aaucaaagucuuugggu | cccugagagauuuggag | aaucaaagucuuuggguugucuucucaugacuugcgcugcagcccugagagauuuggag | NODE\_478161\_length\_16258\_cov\_25.415857:13907..13966:- |
| NODE\_81238\_length\_2792\_cov\_141.863892\_6616 | 1.3e+2 | 0.74 ± 0.03 |  | 273 | 255 | 3 | 15 | no |  |  |  | blast | acugaaguuauucuggggcacccu | guguugaaaauguagacuuucuuuu | guguugaaaauguagacuuucuuuuggccgacauggcugaaggcacugaaguuauucuggggcacccu | NODE\_81238\_length\_2792\_cov\_141.863892:848..916:- |
| NODE\_811930\_length\_34643\_cov\_28.364460\_45660 | 1.3e+2 | 0.74 ± 0.03 |  | 267 | 241 | 0 | 26 | yes |  |  |  | blast | uuucuuuuuuucuccugacaga | ccucaggagaaaaaaugauaaau | ccucaggagaaaaaaugauaaaugauaaaauuccuaaaagccuuuucuuuuucuuuuuuucuccugacaga | NODE\_811930\_length\_34643\_cov\_28.364460:2496..2567:- |
| NODE\_459789\_length\_20351\_cov\_29.716820\_29631 | 1.3e+2 | 0.74 ± 0.03 |  | 259 | 252 | 0 | 7 | yes |  |  |  | blast | gcugcaggcgcuggagagcuuc | agcuccucccgcuccagcagguu | agcuccucccgcuccagcagguugugaucuuggcugagcagcugcaggcgcuggagagcuuc | NODE\_459789\_length\_20351\_cov\_29.716820:10962..11024:+ |
| NODE\_116495\_length\_253\_cov\_20.901186\_9047 | 1.3e+2 | 0.74 ± 0.03 |  | 252 | 159 | 20 | 73 | yes |  | oni-miR-10696 |  | blast | auggaguguaaccagagguuugauu | ucagcuucauccgacauuaacaggc | ucagcuucauccgacauuaacaggcgagaguaggggccaggaaaaggccugccaugcuccaacucaaauggaguguaaccagagguuugauu | NODE\_116495\_length\_253\_cov\_20.901186:222..314:+ |
| NODE\_295706\_length\_22420\_cov\_29.557449\_20490 | 1.3e+2 | 0.74 ± 0.03 |  | 268 | 265 | 0 | 3 | no |  |  |  | blast | uuguagauauagccaagaacgacu | ugcacccgguuguucacgaaaacaug | ugcacccgguuguucacgaaaacaugaaaucuucuugucucauuguagauauagccaagaacgacu | NODE\_295706\_length\_22420\_cov\_29.557449:20643..20709:- |
| NODE\_650038\_length\_41408\_cov\_28.728651\_39724 | 1.2e+2 | 0.74 ± 0.03 |  | 263 | 251 | 0 | 12 | no |  |  |  | blast | ugacugcucaguggcacggcuca | cagccuccucugucggucauga | cagccuccucugucggucaugacugaaccagacaugacugcucaguggcacggcuca | NODE\_650038\_length\_41408\_cov\_28.728651:16630..16687:- |
| NODE\_844531\_length\_14409\_cov\_31.280102\_46570 | 1.2e+2 | 0.74 ± 0.03 |  | 247 | 229 | 0 | 18 | yes |  |  |  | blast | aaacugaccccagagccgcuga | agcggcuuuggggucaguuugu | aaacugaccccagagccgcugaauggaauucaugcagcggcuuuggggucaguuugu | NODE\_844531\_length\_14409\_cov\_31.280102:349..406:+ |
| NODE\_650572\_length\_7410\_cov\_24.068556\_39806 | 1.2e+2 | 0.74 ± 0.03 |  | 255 | 247 | 0 | 8 | no |  | mze-miR-33b |  | blast | uaauguacaagucuggugagcuacu | gauucucagauuuucaguuguguucu | gauucucagauuuucaguuguguucuuugaguugguuauaauguacaagucuggugagcuacu | NODE\_650572\_length\_7410\_cov\_24.068556:3398..3461:- |
| NODE\_619556\_length\_22707\_cov\_31.124807\_38236 | 1.2e+2 | 0.74 ± 0.03 |  | 245 | 218 | 2 | 25 | yes |  |  |  | blast | cacagaguccugaccuaauuc | auuagaguggagacugugaggc | cacagaguccugaccuaauucauagaacaccuuugggaugaauuagaguggagacugugaggc | NODE\_619556\_length\_22707\_cov\_31.124807:8716..8779:+ |
| NODE\_464352\_length\_37913\_cov\_25.046686\_29887 | 1.2e+2 | 0.74 ± 0.03 |  | 255 | 252 | 0 | 3 | no |  |  |  | blast | ugaauuucagcguagcgcugacc | gucggcacucgcagaaacacacuc | gucggcacucgcagaaacacacucaaaacgugauugugugaauuuagugaauuucagcguagcgcugacc | NODE\_464352\_length\_37913\_cov\_25.046686:29496..29566:+ |
| NODE\_912144\_length\_26918\_cov\_26.152203\_47741 | 1.2e+2 | 0.74 ± 0.03 |  | 242 | 236 | 0 | 6 | yes |  |  |  | blast | uuuggaugcuugggaauucuca | agaauucccaagcauccaaaau | uuuggaugcuugggaauucucagugugacguugagaauucccaagcauccaaaau | NODE\_912144\_length\_26918\_cov\_26.152203:25020..25075:- |
| NODE\_96515\_length\_8572\_cov\_27.190388\_7756 | 1.2e+2 | 0.74 ± 0.03 |  | 241 | 234 | 0 | 7 | yes |  |  |  | blast | aaacucuucuugucagaaccccg | gggccugauaagaagaguuucc | aaacucuucuugucagaaccccgcccacuucaaugggggccugauaagaagaguuucc | NODE\_96515\_length\_8572\_cov\_27.190388:7752..7810:+ |
| NODE\_88353\_length\_13191\_cov\_32.398983\_7217 | 1.2e+2 | 0.74 ± 0.03 |  | 245 | 229 | 0 | 16 | yes |  |  |  | blast | aauaagaccaauaugaccaagu | acauacauauggucuuacuga | aauaagaccaauaugaccaaguacuaaaugcauucuguacauacauauggucuuacuga | NODE\_88353\_length\_13191\_cov\_32.398983:13112..13171:- |
| NODE\_667785\_length\_34944\_cov\_26.957161\_40635 | 1.1e+2 | 0.74 ± 0.03 |  | 235 | 171 | 0 | 64 | no |  | abu-miR-15a |  | blast | cagcagcagcucagugacgaug | cugucaucugagcggccggau | cugucaucugagcggccggauguccugaucagugaagacagcagcagcucagugacgaug | NODE\_667785\_length\_34944\_cov\_26.957161:33741..33801:+ |
| NODE\_316894\_length\_18918\_cov\_23.988899\_21767 | 1.1e+2 | 0.74 ± 0.03 |  | 231 | 220 | 0 | 11 | yes |  |  |  | blast | cagacucguugagcuggcucaca | ugggcucgcucggcagccugcaa | ugggcucgcucggcagccugcaaacauugaucucugcagacucguugagcuggcucaca | NODE\_316894\_length\_18918\_cov\_23.988899:16334..16393:+ |
| NODE\_8395\_length\_10084\_cov\_28.521816\_893 | 1.1e+2 | 0.74 ± 0.03 |  | 239 | 238 | 0 | 1 | no |  |  |  | blast | ucuguacaugaugugcuugacc | agcagcacagagggaugucgagagc | agcagcacagagggaugucgagagcgugacuaugacagcucuguacaugaugugcuugacc | NODE\_8395\_length\_10084\_cov\_28.521816:2188..2249:+ |
| NODE\_1005605\_length\_18789\_cov\_25.052372\_48533 | 1.1e+2 | 0.74 ± 0.03 |  | 229 | 190 | 0 | 39 | yes |  |  |  | blast | ccaagaugugaaguuugccacu | uggcagaaccccaucucugugg | uggcagaaccccaucucuguggccauauucgcucacggccaagaugugaaguuugccacu | NODE\_1005605\_length\_18789\_cov\_25.052372:9536..9596:+ |
| NODE\_106232\_length\_8611\_cov\_25.181976\_8442 | 1.1e+2 | 0.74 ± 0.03 |  | 238 | 230 | 1 | 7 | no |  |  |  | blast | ucagugcugauggcgacugagc | uugcucgcccucagcaacgacg | uugcucgcccucagcaacgacgugucuguaaagccucagugcugauggcgacugagc | NODE\_106232\_length\_8611\_cov\_25.181976:3766..3823:- |
| NODE\_707551\_length\_20208\_cov\_28.583284\_42187 | 1.1e+2 | 0.74 ± 0.03 |  | 228 | 198 | 0 | 30 | yes |  |  |  | blast | ucaacaagcgcuuguguguucu | aagcgcgagccuugaauugauc | aagcgcgagccuugaauugauccggugaaagaguugacgaucaacaagcgcuuguguguucu | NODE\_707551\_length\_20208\_cov\_28.583284:19998..20060:+ |
| NODE\_517395\_length\_17946\_cov\_32.805248\_33029 | 1.1e+2 | 0.74 ± 0.03 |  | 233 | 120 | 0 | 113 | yes |  |  |  | blast | ugcacuguuagaaaaccuugc | uuugguuuuucugcaguacgua | uuugguuuuucugcaguacguacaacuaauugaauuuaugcacuguuagaaaaccuugc | NODE\_517395\_length\_17946\_cov\_32.805248:9483..9542:+ |
| NODE\_715991\_length\_1221\_cov\_24.533169\_42551 | 1.1e+2 | 0.74 ± 0.03 |  | 231 | 184 | 0 | 47 | no |  |  |  | blast | uguauccggacugcagugguguagc | ucggcagugugguucgcagacgucugc | ucggcagugugguucgcagacgucugcccauugaaagcucugcagggcuguauccggacugcagugguguagc | NODE\_715991\_length\_1221\_cov\_24.533169:705..778:+ |
| NODE\_388295\_length\_11960\_cov\_26.054264\_25973 | 1.1e+2 | 0.74 ± 0.03 |  | 221 | 163 | 0 | 58 | yes |  |  |  | blast | aauugggacuguuguggacguc | cguccacaguagucccaguuuc | aauugggacuguuguggacguccuuaacuugugcagccguccacaguagucccaguuuc | NODE\_388295\_length\_11960\_cov\_26.054264:2705..2764:- |
| NODE\_55829\_length\_3232\_cov\_30.118193\_4860 | 1.1e+2 | 0.74 ± 0.03 |  | 221 | 213 | 1 | 7 | yes |  |  |  | blast | uguugagcagaggauuucuuugg | caaagagguuccugcuuuaucuacagc | uguugagcagaggauuucuuugggguuacauuauuuuccaaagagguuccugcuuuaucuacagc | NODE\_55829\_length\_3232\_cov\_30.118193:526..591:+ |
| NODE\_372357\_length\_8577\_cov\_31.955345\_24983 | 1.1e+2 | 0.74 ± 0.03 |  | 232 | 144 | 22 | 66 | no |  |  |  | blast | uaccaagggcucagagauuaacuc | ugguaaucggagcacucggugc | uaccaagggcucagagauuaacucgagaagauguggagggugaagacaacaguggucucagugguaaucggagcacucggugc | NODE\_372357\_length\_8577\_cov\_31.955345:4888..4971:- |
| NODE\_581740\_length\_21017\_cov\_31.139601\_36349 | 1.1e+2 | 0.74 ± 0.03 |  | 244 | 240 | 3 | 1 | no |  |  |  | blast | uguuggaauuuuaaagaaaa | uuucuuacuuuuuuccugcuguuga | uguuggaauuuuaaagaaaauggaauauguagccaauaaaauagauuuuuguuuugguuauuuucuuacuuuuuuccugcuguuga | NODE\_581740\_length\_21017\_cov\_31.139601:1963..2049:- |
| NODE\_309383\_length\_73\_cov\_308.972595\_21332 | 1.1e+2 | 0.74 ± 0.03 |  | 296 | 270 | 7 | 19 | no |  |  |  | blast | uaaguccguagcucaaagaugaacu | uacuuagggauucaaau | uacuuagggauucaaauuaagucaaaaaguucuuaccguuagugcagaaaauaaguccguagcucaaagaugaacu | NODE\_309383\_length\_73\_cov\_308.972595:25..101:- |
| NODE\_305204\_length\_10557\_cov\_24.591930\_21184 | 1.0e+2 | 0.74 ± 0.03 |  | 210 | 209 | 0 | 1 | no |  |  |  | blast | uucgaaguucgggugcggugaggcc | ccgcuucacucaaacugag | uucgaaguucgggugcggugaggccuaaaacugauggccgcuucacucaaacugag | NODE\_305204\_length\_10557\_cov\_24.591930:428..484:+ |
| NODE\_500742\_length\_10339\_cov\_29.786245\_32030 | 1.0e+2 | 0.74 ± 0.03 |  | 194 | 179 | 0 | 15 | yes |  | oni-miR-10611 |  | blast | uuucccuuuuucacuccuuucc | caaggcguaaaaaaggguuugu | caaggcguaaaaaaggguuugugucauuuuucacucuucacuuucccuuuuucacuccuuucc | NODE\_500742\_length\_10339\_cov\_29.786245:688..751:- |
| NODE\_295636\_length\_16811\_cov\_24.376123\_20486 | 1.0e+2 | 0.74 ± 0.03 |  | 198 | 184 | 0 | 14 | yes |  |  |  | blast | uggaggaucagugcugugccu | caucgcucugcuccgcugcc | uggaggaucagugcugugccucccugagaggcaucgcucugcuccgcugcc | NODE\_295636\_length\_16811\_cov\_24.376123:1489..1540:+ |
| NODE\_859305\_length\_5007\_cov\_13.324346\_46903 | 1.0e+2 | 0.74 ± 0.03 |  | 218 | 215 | 2 | 1 | no |  |  |  | blast | auucaugagauucuguccggagac | uccuuugcgcagauucagcaugucggc | uccuuugcgcagauucagcaugucggcccaaaaugcaauaaggagcaauaaaacagccauucaugagauucuguccggagac | NODE\_859305\_length\_5007\_cov\_13.324346:1833..1915:+ |
| NODE\_211540\_length\_271\_cov\_64.575645\_15568 | 1.0e+2 | 0.74 ± 0.03 |  | 218 | 215 | 2 | 1 | no |  |  |  | blast | auucaugagauucuguccggagac | uccuuugcgcagauucagcaugucggc | uccuuugcgcagauucagcaugucggcccaaaaugcaauaaggagcaauaaaacagccauucaugagauucuguccggagac | NODE\_211540\_length\_271\_cov\_64.575645:42..124:- |
| NODE\_122490\_length\_4447\_cov\_24.984484\_9548 | 1.0e+2 | 0.74 ± 0.03 |  | 218 | 215 | 2 | 1 | no |  |  |  | blast | auucaugagauucuguccggagac | uccuuugcgcagauucagcaugucggc | uccuuugcgcagauucagcaugucggcccaaaaugcaauaaggagcaauaaaacagccauucaugagauucuguccggagac | NODE\_122490\_length\_4447\_cov\_24.984484:4231..4313:- |
| NODE\_635560\_length\_31730\_cov\_30.409706\_39069 | 9.7e+1 | 0.74 ± 0.03 |  | 192 | 42 | 0 | 150 | yes |  |  |  | blast | ucucugcuuucucucuaccagg | uuggugugggaagaaaguagagc | uuggugugggaagaaaguagagcuaaguucugaccccaucucugcuuucucucuaccagg | NODE\_635560\_length\_31730\_cov\_30.409706:21859..21919:+ |
| NODE\_691354\_length\_3535\_cov\_24.868458\_41607 | 9.7e+1 | 0.74 ± 0.03 |  | 213 | 210 | 2 | 1 | no |  |  |  | blast | uucuggacuaguuuuaucu | acaacaacugguguacgaacu | uucuggacuaguuuuaucuuggaguugaggcacaaaggguuuuaccaaaacugcugcaagaacaacaacugguguacgaacu | NODE\_691354\_length\_3535\_cov\_24.868458:1138..1220:- |
| NODE\_159748\_length\_8115\_cov\_33.378559\_12162 | 9.7e+1 | 0.74 ± 0.03 |  | 200 | 199 | 0 | 1 | no |  |  |  | blast | ugguaaagcuuugucccugaggu | aacagacaugcuuaugcc | ugguaaagcuuugucccugagguaguugaaaaaccccaguuggcuaaagcguugggguuuuuuguuaacagacaugcuuaugcc | NODE\_159748\_length\_8115\_cov\_33.378559:4718..4802:+ |
| NODE\_777120\_length\_79213\_cov\_28.738705\_44705 | 9.3e+1 | 0.74 ± 0.03 |  | 185 | 144 | 39 | 2 | yes |  |  |  | blast | gaagagggcuauguggucagaca | caaaccucugucuucuuc | caaaccucugucuucuucagauuauccugacuaugagcuggauccuaaauaugaagagggcuauguggucagaca | NODE\_777120\_length\_79213\_cov\_28.738705:79116..79191:+ |
| NODE\_345812\_length\_10942\_cov\_30.590111\_23387 | 8.9e+1 | 0.74 ± 0.03 |  | 174 | 165 | 0 | 9 | yes |  |  |  | blast | uggcagcacaugaugauuuga | caaaacaugaugugcugcuaga | uggcagcacaugaugauuugaagcgauucagcacaucugcaaaacaugaugugcugcuaga | NODE\_345812\_length\_10942\_cov\_30.590111:7587..7648:- |
| NODE\_261437\_length\_3391\_cov\_24.532587\_18647 | 8.9e+1 | 0.74 ± 0.03 |  | 167 | 161 | 0 | 6 | yes |  | gmo-miR-11252-3p |  | blast | uuagagggcuggaccaauaagcu | cuuuuuggugcaguccucuaca | uuagagggcuggaccaauaagcuaauaaagagauaaaauuaauauugaggucagcuuuuuggugcaguccucuaca | NODE\_261437\_length\_3391\_cov\_24.532587:1824..1900:- |
| NODE\_491304\_length\_4123\_cov\_31.826097\_31413 | 8.7e+1 | 0.74 ± 0.03 |  | 169 | 138 | 0 | 31 | yes |  |  |  | blast | aaagccgacaacucacagauu | aucugugagauguuggcuuuc | aucugugagauguuggcuuucagccacagcggcauguugggcgagaaagccgacaacucacagauu | NODE\_491304\_length\_4123\_cov\_31.826097:3466..3532:+ |
| NODE\_100328\_length\_37307\_cov\_31.060150\_8006 | 8.7e+1 | 0.74 ± 0.03 |  | 172 | 127 | 0 | 45 | yes |  |  |  | blast | ugccuaugucugcugugcaaca | agugcacaguuagaacaggccu | agugcacaguuagaacaggccugcccaccuacauggguguugccuaugucugcugugcaaca | NODE\_100328\_length\_37307\_cov\_31.060150:14487..14549:- |
| NODE\_475395\_length\_53320\_cov\_28.361834\_30417 | 8.7e+1 | 0.74 ± 0.03 |  | 177 | 116 | 0 | 61 | no |  |  |  | blast | augcagagccgccuucugagccu | ccuggaguagcaccugcagcca | augcagagccgccuucugagccugucauguucaaucaggccuggaguagcaccugcagcca | NODE\_475395\_length\_53320\_cov\_28.361834:8574..8635:- |
| NODE\_507791\_length\_7198\_cov\_16.655321\_32522 | 8.5e+1 | 0.74 ± 0.03 |  | 186 | 185 | 0 | 1 | no |  |  |  | blast | auguccagaaccugaaguc | cccggucuuugaacugccu | cccggucuuugaacugccuguggaagacaaucaacccacucauucacaguauguccagaaccugaaguc | NODE\_507791\_length\_7198\_cov\_16.655321:5072..5141:- |
| NODE\_356848\_length\_16706\_cov\_31.098827\_23966 | 8.2e+1 | 0.74 ± 0.03 |  | 160 | 153 | 0 | 7 | yes |  |  |  | blast | uuuguuuguuucuauucuuugcu | aaagaacagaaacaaacagaa | uuuguuuguuucuauucuuugcucagucugcugagcaaagaacagaaacaaacagaa | NODE\_356848\_length\_16706\_cov\_31.098827:9299..9356:+ |
| NODE\_51403\_length\_8504\_cov\_27.146519\_4459 | 8.1e+1 | 0.74 ± 0.03 |  | 157 | 155 | 0 | 2 | yes |  |  |  | blast | ucaccccucucucucucuccu | gagggaggugggagggguggaga | ucaccccucucucucucuccuggaguuagaggaggaggagggaggugggagggguggaga | NODE\_51403\_length\_8504\_cov\_27.146519:8200..8260:- |
| NODE\_387420\_length\_32930\_cov\_32.582874\_25914 | 7.8e+1 | 0.74 ± 0.03 |  | 155 | 129 | 0 | 26 | no |  | oni-miR-10563 |  | blast | ugacaggucaauagagagaaga | ccucucuuuuuuguccugaugc | ugacaggucaauagagagaagauguguauuugaauuccucucuuuuuuguccugaugc | NODE\_387420\_length\_32930\_cov\_32.582874:17179..17237:+ |
| NODE\_433780\_length\_65\_cov\_25.092308\_28175 | 7.8e+1 | 0.74 ± 0.03 |  | 164 | 163 | 0 | 1 | yes |  |  |  | blast | uauguuacacuguugaaaugaugg | caucucccgugcaacauauuuauuu | uauguuacacuguugaaaugaugggccaacucaucucccgugcaacauauuuauuu | NODE\_433780\_length\_65\_cov\_25.092308:81..137:- |
| NODE\_576859\_length\_42602\_cov\_28.136402\_36169 | 7.7e+1 | 0.74 ± 0.03 |  | 161 | 109 | 1 | 51 | no |  |  |  | blast | agcagaauggaggagaacucg | aguuuuucuucuucucugcaga | agcagaauggaggagaacucguguccccagcugugguuuccuuuaauggaguuuuucuucuucucugcaga | NODE\_576859\_length\_42602\_cov\_28.136402:31895..31966:- |
| NODE\_101242\_length\_2575\_cov\_25.072233\_8092 | 7.6e+1 | 0.74 ± 0.03 |  | 148 | 133 | 0 | 15 | yes |  |  |  | blast | ugcagauggagcgcugcugacu | aaggcagugcucuaccugcacu | aaggcagugcucuaccugcacuguguauagugcagauggagcgcugcugacu | NODE\_101242\_length\_2575\_cov\_25.072233:1506..1558:- |
| NODE\_983416\_length\_31818\_cov\_30.330065\_48377 | 7.5e+1 | 0.74 ± 0.03 |  | 148 | 142 | 0 | 6 | yes |  |  |  | blast | aagagcgucaaaucaaaugga | caguugauugugacacuugugu | aagagcgucaaaucaaauggagcugucucagggauguccaguugauugugacacuugugu | NODE\_983416\_length\_31818\_cov\_30.330065:24920..24980:- |
| NODE\_676647\_length\_100\_cov\_384.290009\_40970 | 7.1e+1 | 0.74 ± 0.03 |  | 167 | 162 | 4 | 1 | no |  |  |  | blast | acgcuuaguaggaccacaaaguu | cuuuuggucaaauacauuacacauu | cuuuuggucaaauacauuacacauuuguccuuuuugaaacacauaugcacaaaaugcaaguaacgcuuaguaggaccacaaaguu | NODE\_676647\_length\_100\_cov\_384.290009:27..112:- |
| NODE\_58628\_length\_2492\_cov\_31.966694\_4995 | 7.0e+1 | 0.74 ± 0.03 |  | 138 | 106 | 0 | 32 | yes |  |  |  | blast | ucaccaaacuguccuuggaua | ucggaggauguuuugguuuggc | ucaccaaacuguccuuggauaguuggaagaaauugcugucggaggauguuuugguuuggc | NODE\_58628\_length\_2492\_cov\_31.966694:1251..1311:- |
| NODE\_509698\_length\_61822\_cov\_30.197180\_32544 | 7.0e+1 | 0.74 ± 0.03 |  | 135 | 87 | 0 | 48 | yes |  |  |  | blast | ccuguuccagauguugcccuc | agggugacagcuggaacaggu | agggugacagcuggaacaggugaugugaaggacgcaucccacccagcauggcgccuguuccagauguugcccuc | NODE\_509698\_length\_61822\_cov\_30.197180:58163..58237:- |
| NODE\_331709\_length\_20512\_cov\_27.546265\_22680 | 6.9e+1 | 0.74 ± 0.03 |  | 132 | 124 | 0 | 8 | yes |  |  |  | blast | uucacaucccaggaggcacuga | gugccuccugggaugugaau | gugccuccugggaugugaaucugaguagauucacaucccaggaggcacuga | NODE\_331709\_length\_20512\_cov\_27.546265:9985..10036:- |
| NODE\_331709\_length\_20512\_cov\_27.546265\_22677 | 6.9e+1 | 0.74 ± 0.03 |  | 132 | 124 | 0 | 8 | yes |  |  |  | blast | uucacaucccaggaggcacuga | gugccuccugggaugugaau | gugccuccugggaugugaaucuacucagauucacaucccaggaggcacuga | NODE\_331709\_length\_20512\_cov\_27.546265:9988..10039:+ |
| NODE\_422367\_length\_49472\_cov\_29.010834\_27603 | 6.8e+1 | 0.74 ± 0.03 |  | 141 | 117 | 0 | 24 | no |  |  |  | blast | ucauugucuguuggcuucccuuc | gaggaaagcaaccaagacacu | gaggaaagcaaccaagacacugggcaucauccugggcgcguucauugucuguuggcuucccuuc | NODE\_422367\_length\_49472\_cov\_29.010834:8100..8164:+ |
| NODE\_670780\_length\_5273\_cov\_19.867249\_40811 | 6.8e+1 | 0.74 ± 0.03 |  | 135 | 132 | 0 | 3 | yes |  |  |  | blast | uuucucgguaacaguccauuuaagc | uuaaaugcgcuguaauacuuguca | uuucucgguaacaguccauuuaagcgcaagaaguuccaguuuaaaugcgcuguaauacuuguca | NODE\_670780\_length\_5273\_cov\_19.867249:1197..1261:- |
| NODE\_644851\_length\_47515\_cov\_28.577627\_39482 | 6.7e+1 | 0.74 ± 0.03 |  | 131 | 111 | 0 | 20 | yes |  |  |  | blast | uacagaagaggauuaggcccacu | ugggcccagaauuccucuucugu | uacagaagaggauuaggcccacuagaaaaagaaagugggcccagaauuccucuucugu | NODE\_644851\_length\_47515\_cov\_28.577627:34435..34493:+ |
| NODE\_983416\_length\_31818\_cov\_30.330065\_48378 | 6.6e+1 | 0.74 ± 0.03 |  | 143 | 142 | 0 | 1 | no |  |  |  | blast | aagagcgucaaaucaaaugga | ucuaacuugaaagcaugcc | ucuaacuugaaagcaugccuuacacagacaagagcgucaaaucaaaugga | NODE\_983416\_length\_31818\_cov\_30.330065:24959..25009:- |
| NODE\_463036\_length\_32999\_cov\_28.302191\_29787 | 6.5e+1 | 0.74 ± 0.03 |  | 138 | 137 | 0 | 1 | no |  |  |  | blast | ugaacuggaccuuuucuugccu | aguaaguuaagcuccagccagc | aguaaguuaagcuccagccagcagcucucaggcugaacuggaccuuuucuugccu | NODE\_463036\_length\_32999\_cov\_28.302191:15218..15273:+ |
| NODE\_562319\_length\_13508\_cov\_29.571735\_35346 | 6.5e+1 | 0.74 ± 0.03 |  | 138 | 137 | 0 | 1 | no |  |  |  | blast | cuguaacugaagcgcagacuuggc | uagugugcugugguaca | cuguaacugaagcgcagacuuggcccagguguggcauagugugcugugguaca | NODE\_562319\_length\_13508\_cov\_29.571735:8518..8571:+ |
| NODE\_369104\_length\_4804\_cov\_23.074312\_24651 | 6.4e+1 | 0.74 ± 0.03 |  | 126 | 118 | 0 | 8 | yes |  |  |  | blast | gaacucccaguauccucacagc | cugugcgcuacccggagcuuaa | gaacucccaguauccucacagccacaacuaccaggcugugcgcuacccggagcuuaa | NODE\_369104\_length\_4804\_cov\_23.074312:978..1035:- |
| NODE\_112434\_length\_758\_cov\_80.699211\_8850 | 6.3e+1 | 0.74 ± 0.03 |  | 130 | 91 | 0 | 39 | no |  |  |  | blast | ugagcucggaaagcaaaucuugccu | uaagaagcguuucuggggcuccgauu | uaagaagcguuucuggggcuccgauucgccggaacaucgggagugagcucggaaagcaaaucuugccu | NODE\_112434\_length\_758\_cov\_80.699211:652..720:- |
| NODE\_252178\_length\_123\_cov\_125.439026\_18004 | 6.1e+1 | 0.74 ± 0.03 |  | 151 | 150 | 0 | 1 | no |  |  |  | blast | auacuugaaaucugggaaccuucc | uaagauguaacaggguu | uaagauguaacaggguuacaagcaauacuagccuccaaagucaggaaaguuacgaaauacuugaaaucugggaaccuucc | NODE\_252178\_length\_123\_cov\_125.439026:96..176:- |
| NODE\_255441\_length\_119\_cov\_49.310925\_18123 | 6.1e+1 | 0.74 ± 0.03 |  | 130 | 127 | 0 | 3 | yes |  |  |  | blast | acuaaacucuggcugcaauugc | uuugcagcuauuuguuuauuc | uuugcagcuauuuguuuauucguauauuugagauugacuaaacucuggcugcaauugc | NODE\_255441\_length\_119\_cov\_49.310925:10..68:+ |
| NODE\_628906\_length\_6644\_cov\_30.155930\_38726 | 5.9e+1 | 0.74 ± 0.03 |  | 114 | 54 | 0 | 60 | yes |  |  |  | blast | aguggcacguggagaccuggac | caggucuccacaugccacuga | aguggcacguggagaccuggacccuuccaggucuccacaugccacuga | NODE\_628906\_length\_6644\_cov\_30.155930:1901..1949:+ |
| NODE\_124395\_length\_16798\_cov\_29.836647\_9711 | 5.8e+1 | 0.74 ± 0.03 |  | 126 | 118 | 0 | 8 | no |  |  |  | blast | cgcgggaugugugcgaucugaca | agcagaucacgcacaaucacg | agcagaucacgcacaaucacgcacggacacgcagacagagcgcgcgggaugugugcgaucugaca | NODE\_124395\_length\_16798\_cov\_29.836647:4916..4981:+ |
| NODE\_871085\_length\_21924\_cov\_28.946589\_47089 | 5.8e+1 | 0.74 ± 0.03 |  | 117 | 92 | 0 | 25 | yes |  |  |  | blast | uaaccagagcucuugauuuauc | uaaaucauuggcaccuggugacu | uaaccagagcucuugauuuaucuuuugauuuugcaacuuuaaaucauuggcaccuggugacu | NODE\_871085\_length\_21924\_cov\_28.946589:4005..4067:- |
| NODE\_128862\_length\_18763\_cov\_25.828119\_10011 | 5.8e+1 | 0.74 ± 0.03 |  | 113 | 106 | 1 | 6 | yes |  |  |  | blast | gaaacggaccccauaaccagcu | ugcauuauggguuccguuacaa | gaaacggaccccauaaccagcuggggcucagaaucacagcugcauuauggguuccguuacaa | NODE\_128862\_length\_18763\_cov\_25.828119:9180..9242:- |
| NODE\_140268\_length\_8766\_cov\_21.932808\_10821 | 5.8e+1 | 0.74 ± 0.03 |  | 113 | 97 | 1 | 15 | yes |  |  |  | blast | ucgucucugcucucggguccuuc | aggcccgaggcucgcagagugu | aggcccgaggcucgcagagugucgggauuaaaaugagaaaccuuaaaugcucgucucugcucucggguccuuc | NODE\_140268\_length\_8766\_cov\_21.932808:3837..3910:+ |
| NODE\_470507\_length\_123\_cov\_231.967484\_30113 | 5.6e+1 | 0.74 ± 0.03 |  | 154 | 152 | 1 | 1 | no |  |  |  | blast | uauuggauuccacauuucuuacag | ugugcugagugaaucuaguuaaaag | ugugcugagugaaucuaguuaaaaguguguuucccguuucgcccacauauuggauuccacauuucuuacag | NODE\_470507\_length\_123\_cov\_231.967484:105..176:+ |
| NODE\_420725\_length\_1561\_cov\_13.609865\_27538 | 5.4e+1 | 0.74 ± 0.03 |  | 106 | 99 | 0 | 7 | yes |  |  |  | blast | ccuuccccggcguguacuuga | aaguggaaaccgggagaaugug | aaguggaaaccgggagaauguguccgcaucuauguucaccuuccccggcguguacuuga | NODE\_420725\_length\_1561\_cov\_13.609865:993..1052:- |
| NODE\_516550\_length\_63184\_cov\_25.322027\_32884 | 5.4e+1 | 0.74 ± 0.03 |  | 105 | 100 | 0 | 5 | yes |  |  |  | blast | ucugaaacauguacgugugaca | ucacacuaaaauguuucagguc | ucacacuaaaauguuucaggucaucaauucguuugaucugaaacauguacgugugaca | NODE\_516550\_length\_63184\_cov\_25.322027:1064..1122:- |
| NODE\_865274\_length\_2098\_cov\_20.701143\_47056 | 5.4e+1 | 0.74 ± 0.03 |  | 107 | 90 | 0 | 17 | yes |  |  |  | blast | cucuugaacugaacuaggacu | auucugguucugcucaugaac | auucugguucugcucaugaaccagaucucugagugauccucuugaacugaacuaggacu | NODE\_865274\_length\_2098\_cov\_20.701143:1447..1506:- |
| NODE\_1049000\_length\_42433\_cov\_27.435345\_48762 | 5.4e+1 | 0.74 ± 0.03 |  | 105 | 69 | 0 | 36 | yes |  |  |  | blast | acaggccccguuucucauguag | acaugagaaacugggacuguu | acaugagaaacugggacuguugucgagggcccuacauaacaggccccguuucucauguag | NODE\_1049000\_length\_42433\_cov\_27.435345:27576..27636:- |
| NODE\_417308\_length\_2186\_cov\_27.373741\_27414 | 5.4e+1 | 0.74 ± 0.03 |  | 112 | 92 | 3 | 17 | no |  |  |  | blast | caucaccucgucuccucuguuu | ccagaggaggaguugugcugcag | ccagaggaggaguugugcugcaggcggaccgagggcaccugcaucaccucgucuccucuguuu | NODE\_417308\_length\_2186\_cov\_27.373741:1591..1654:- |
| NODE\_1032297\_length\_84705\_cov\_29.139343\_48683 | 5.2e+1 | 0.74 ± 0.03 |  | 112 | 111 | 0 | 1 | no |  |  |  | blast | acggcuguguccucucugcaga | agcugaguggaucugcugcuca | agcugaguggaucugcugcucagcccugaauccuugaucagaguuauugacggcuguguccucucugcaga | NODE\_1032297\_length\_84705\_cov\_29.139343:34671..34742:+ |
| NODE\_51964\_length\_11686\_cov\_26.693222\_4584 | 5.2e+1 | 0.74 ± 0.03 |  | 99 | 58 | 1 | 40 | yes |  |  |  | blast | aaacagcuccgcgucaguuucu | aacugacgcggagcuguuuuga | aaacagcuccgcgucaguuucuuuguuuuaaagaaacugacgcggagcuguuuuga | NODE\_51964\_length\_11686\_cov\_26.693222:7909..7965:+ |
| NODE\_51964\_length\_11686\_cov\_26.693222\_4585 | 5.1e+1 | 0.74 ± 0.03 |  | 98 | 58 | 0 | 40 | yes |  |  |  | blast | aaacagcuccgcgucaguuucu | aacugacgcggagcuguuuuga | aaacagcuccgcgucaguuucuuuaaaacaaagaaacugacgcggagcuguuuuga | NODE\_51964\_length\_11686\_cov\_26.693222:7906..7962:- |
| NODE\_19188\_length\_32887\_cov\_29.349895\_1948 | 5.0e+1 | 0.74 ± 0.03 |  | 117 | 116 | 0 | 1 | no |  |  |  | blast | uaucgauucacuucuuccuuugcc | ucaaaggauccuuagaaa | ucaaaggauccuuagaaaguggaacagguugaacuucccuguaucgauucacuucuuccuuugcc | NODE\_19188\_length\_32887\_cov\_29.349895:8200..8265:- |
| NODE\_66414\_length\_1536\_cov\_19.555338\_5467 | 5.0e+1 | 0.74 ± 0.03 |  | 109 | 107 | 0 | 2 | no |  |  |  | blast | aauccgcucagcuguucgcacugc | cacugggaaacagcagcaucaugaac | aauccgcucagcuguucgcacugcggggaaacacugggaaacagcagcaucaugaac | NODE\_66414\_length\_1536\_cov\_19.555338:985..1042:- |
| NODE\_478501\_length\_2811\_cov\_21.604767\_30761 | 4.9e+1 | 0.74 ± 0.03 |  | 115 | 96 | 18 | 1 | no |  |  |  | blast | uccuggugaauaguccugcacu | gacaagaacaccuucac | gacaagaacaccuucacguggacuucagaugcacaagcgagcuuugaagaaguaaagaaacuccuggugaauaguccugcacu | NODE\_478501\_length\_2811\_cov\_21.604767:315..398:+ |
| NODE\_502492\_length\_34030\_cov\_29.276669\_32147 | 4.9e+1 | 0.74 ± 0.03 |  | 119 | 111 | 0 | 8 | no |  |  |  | blast | ucugacuuugugaaucacuagg | uuugugauuugcaaaacugcucu | uuugugauuugcaaaacugcucucuguguuuacgguuacaucugacuuugugaaucacuagg | NODE\_502492\_length\_34030\_cov\_29.276669:18559..18621:+ |
| NODE\_531958\_length\_72\_cov\_40.763889\_33901 | 4.8e+1 | 0.74 ± 0.03 |  | 163 | 162 | 0 | 1 | no |  |  |  | blast | uauguuacacuguugaaaugaugg | caucucccgugcaacauauuuauuu | uauguuacacuguugaaaugaugggccaacucaucucccgugcaacauauuuauuu | NODE\_531958\_length\_72\_cov\_40.763889:8..64:- |
| NODE\_131080\_length\_7845\_cov\_24.421160\_10133 | 4.8e+1 | 0.74 ± 0.03 |  | 95 | 89 | 0 | 6 | yes |  |  |  | blast | gcagaaucaaucaugacugau | acagacaugagugguguugacc | acagacaugagugguguugaccaucaaaucuaaaucuuggcagaaucaaucaugacugau | NODE\_131080\_length\_7845\_cov\_24.421160:7517..7577:+ |
| NODE\_824567\_length\_25513\_cov\_30.081919\_46017 | 4.8e+1 | 0.74 ± 0.03 |  | 94 | 76 | 0 | 18 | yes |  |  |  | blast | uccacacacuguccugcuuacc | guaaguguggcuguguggugcu | uccacacacuguccugcuuaccauuaaaguugaauauugguaaguguggcuguguggugcu | NODE\_824567\_length\_25513\_cov\_30.081919:824..885:- |
| NODE\_973146\_length\_84880\_cov\_28.550789\_48328 | 4.7e+1 | 0.74 ± 0.03 |  | 94 | 85 | 0 | 9 | yes |  |  |  | blast | uccuuucuucugucucugcaga | accagaggcagaugauagaca | accagaggcagaugauagacaguggaugcugccguguagcugcaaucaccuguccuuucuucugucucugcaga | NODE\_973146\_length\_84880\_cov\_28.550789:39068..39142:- |
| NODE\_451843\_length\_48367\_cov\_29.719893\_29192 | 4.7e+1 | 0.74 ± 0.03 |  | 90 | 87 | 0 | 3 | yes |  |  |  | blast | acugaagaaaucaccuggauga | auucaggugauuucuucaguguc | acugaagaaaucaccuggaugacugacugaaaaugcaacauucaggugauuucuucaguguc | NODE\_451843\_length\_48367\_cov\_29.719893:19926..19988:+ |
| NODE\_659880\_length\_5871\_cov\_23.014990\_40237 | 4.6e+1 | 0.74 ± 0.03 |  | 125 | 124 | 0 | 1 | no |  |  |  | blast | uucuuaucaguuugggcgaga | uauguuugagugucaaua | uauguuugagugucaauaaaauuuuucgaaagaaaaaucuguucuuaucaguuugggcgaga | NODE\_659880\_length\_5871\_cov\_23.014990:5542..5604:+ |
| NODE\_196646\_length\_112\_cov\_132.098221\_14669 | 4.6e+1 | 0.74 ± 0.03 |  | 100 | 96 | 0 | 4 | no |  |  |  | blast | ugcgauuaaaucucucugugguacc | ugaagugugggauuuggcaacgccacc | ugcgauuaaaucucucugugguaccauuacccauggggugguauggugaagugugggauuuggcaacgccacc | NODE\_196646\_length\_112\_cov\_132.098221:83..156:- |
| NODE\_438360\_length\_2624\_cov\_14.277821\_28497 | 4.6e+1 | 0.74 ± 0.03 |  | 100 | 96 | 0 | 4 | no |  |  |  | blast | ugcgauuaaaucucucugugguacc | ugaagugugggauuuggcaacgccacc | ugcgauuaaaucucucugugguaccauuacccauggggugguauggugaagugugggauuuggcaacgccacc | NODE\_438360\_length\_2624\_cov\_14.277821:326..399:- |
| NODE\_876065\_length\_1424\_cov\_10.813202\_47178 | 4.5e+1 | 0.74 ± 0.03 |  | 117 | 116 | 0 | 1 | no |  |  |  | blast | uauuagugugacuauaauauccu | uauucuuauagcacauuuuuguagu | uauuagugugacuauaauauccuaccuuauacaauuauucuuauagcacauuuuuguagu | NODE\_876065\_length\_1424\_cov\_10.813202:1063..1123:+ |
| NODE\_608601\_length\_9786\_cov\_23.449213\_37579 | 4.5e+1 | 0.74 ± 0.03 |  | 87 | 66 | 1 | 20 | yes |  |  |  | blast | gacucaguagaccugucuuugg | aaggacaggcagacugaggacc | aaggacaggcagacugaggaccgguaccccucccaggauugugaaggacucaguagaccugucuuugg | NODE\_608601\_length\_9786\_cov\_23.449213:9447..9515:+ |
| NODE\_205670\_length\_3002\_cov\_32.979679\_15109 | 4.5e+1 | 0.74 ± 0.03 |  | 89 | 88 | 0 | 1 | yes |  |  |  | blast | aauuugauuucugacauuugu | caugauguccaaaaucaaacu | aauuugauuucugacauuuguggcguaacuugacacaugauguccaaaaucaaacu | NODE\_205670\_length\_3002\_cov\_32.979679:2162..2218:- |
| NODE\_108005\_length\_3872\_cov\_25.723658\_8583 | 4.5e+1 | 0.74 ± 0.03 |  | 92 | 76 | 0 | 16 | yes |  |  |  | blast | auggaggaauucagaguucgacu | ugucccucugucuuucuccaga | auggaggaauucagaguucgacuuucuuacauucugcaaaaaugacaaaaacguugucccucugucuuucuccaga | NODE\_108005\_length\_3872\_cov\_25.723658:400..476:- |
| NODE\_517655\_length\_22574\_cov\_21.997210\_33046 | 4.4e+1 | 0.74 ± 0.03 |  | 85 | 79 | 0 | 6 | yes |  |  |  | blast | uuuagguucuugaacacguuuc | aacaucuucaggaaccugaaa | uuuagguucuugaacacguuucaagauggcagcugaaacaucuucaggaaccugaaa | NODE\_517655\_length\_22574\_cov\_21.997210:554..611:+ |
| NODE\_154606\_length\_13006\_cov\_26.223434\_11698 | 4.4e+1 | 0.74 ± 0.03 |  | 87 | 79 | 0 | 8 | yes |  |  |  | blast | caucagcguacagcuuaacacc | ugcuaaucugaacggugugc | ugcuaaucugaacggugugcaacguaguuugcaucagcguacagcuuaacacc | NODE\_154606\_length\_13006\_cov\_26.223434:7160..7213:- |
| NODE\_462924\_length\_49097\_cov\_28.299225\_29772 | 4.3e+1 | 0.74 ± 0.03 |  | 85 | 83 | 1 | 1 | yes |  |  |  | blast | ucagugacugucagugaaccu | gcuuucacugcagucaccuuca | ucagugacugucagugaaccuccuugagaugcucuguuaggcuuucacugcagucaccuuca | NODE\_462924\_length\_49097\_cov\_28.299225:42304..42366:+ |
| NODE\_528735\_length\_19007\_cov\_25.772610\_33684 | 4.3e+1 | 0.74 ± 0.03 |  | 92 | 74 | 1 | 17 | no |  |  |  | blast | ucugaacacgucgcaguccugu | ucgucugugacauuuucacuga | ucgucugugacauuuucacugacauguggugacucgcggugcucugaacacgucgcaguccugu | NODE\_528735\_length\_19007\_cov\_25.772610:10326..10390:- |
| NODE\_190700\_length\_20213\_cov\_28.675703\_14126 | 4.2e+1 | 0.74 ± 0.03 |  | 82 | 66 | 0 | 16 | yes |  |  |  | blast | accccagucugguugcgagggc | cuucgccacaguucugggguu | cuucgccacaguucugggguuagccuuuccuaauuuaaccccagucugguugcgagggc | NODE\_190700\_length\_20213\_cov\_28.675703:6352..6411:- |
| NODE\_948106\_length\_16263\_cov\_28.865093\_48097 | 4.1e+1 | 0.74 ± 0.03 |  | 88 | 76 | 0 | 12 | yes |  |  |  | blast | acaaacuuaaagaaguccugucg | acgggacuuuucuaaauugaga | acgggacuuuucuaaauugagagauaaaaggucuucacaaacuuaaagaaguccugucg | NODE\_948106\_length\_16263\_cov\_28.865093:697..756:+ |
| NODE\_6893\_length\_7898\_cov\_26.144341\_802 | 4.1e+1 | 0.74 ± 0.03 |  | 90 | 82 | 0 | 8 | no |  |  |  | blast | ugugaccucaguaaaccaggagu | ugcugguuucugucugguuuca | ugugaccucaguaaaccaggaguuuacagucuccgcugcugguuucugucugguuuca | NODE\_6893\_length\_7898\_cov\_26.144341:863..921:+ |
| NODE\_459453\_length\_54739\_cov\_33.289009\_29620 | 4.0e+1 | 0.74 ± 0.03 |  | 81 | 68 | 0 | 13 | yes |  |  |  | blast | ucgcuagccgaccuguaagcu | uuagcaaguuagcugguggca | ucgcuagccgaccuguaagcugauaccauucaauguuagcaaguuagcugguggca | NODE\_459453\_length\_54739\_cov\_33.289009:15764..15820:+ |
| NODE\_725071\_length\_72140\_cov\_28.177156\_42963 | 3.8e+1 | 0.74 ± 0.03 |  | 83 | 81 | 0 | 2 | no |  |  |  | blast | ucaauaaucuccacaguguca | uagcuguggaggcuaugcug | uagcuguggaggcuaugcugcaguucugauagcuaaucuucaauaaucuccacaguguca | NODE\_725071\_length\_72140\_cov\_28.177156:10041..10101:+ |
| NODE\_230970\_length\_24312\_cov\_26.586542\_16755 | 3.8e+1 | 0.74 ± 0.03 |  | 73 | 44 | 0 | 29 | yes |  |  |  | blast | uuuguuacaacugcuggaauuu | auuccaucauuuguaauaaagu | auuccaucauuuguaauaaaguaacuguaacauuuuacuuuguuacaacugcuggaauuu | NODE\_230970\_length\_24312\_cov\_26.586542:14464..14524:+ |
| NODE\_367234\_length\_24561\_cov\_28.211269\_24578 | 3.7e+1 | 0.74 ± 0.03 |  | 74 | 73 | 0 | 1 | yes |  |  |  | blast | cuuguuguuuucuguuuugu | aaaauagaaaucaacaguacac | cuuguuguuuucuguuuuguauuuacaaggcaaaauagaaaucaacaguacac | NODE\_367234\_length\_24561\_cov\_28.211269:1996..2049:+ |
| NODE\_530411\_length\_52662\_cov\_32.665298\_33801 | 3.6e+1 | 0.74 ± 0.03 |  | 89 | 87 | 1 | 1 | no |  |  |  | blast | auuccucuuucgaccugcagg | gcagguuucagaagagu | gcagguuucagaagaguaauaaaaaagaauuguagcacgugaucucaaugacauccacuucauaauauuccucuuucgaccugcagg | NODE\_530411\_length\_52662\_cov\_32.665298:42958..43045:+ |
| NODE\_643947\_length\_12221\_cov\_29.509043\_39433 | 3.6e+1 | 0.74 ± 0.03 |  | 85 | 51 | 4 | 30 | no |  |  |  | blast | uauccaugauuucuugcuguuuggc | uaaaguagcagugaaaacgaggac | uauccaugauuucuugcuguuuggcauguacccuaguguguccgcagguguauacgagcggaguguaaaguagcagugaaaacgaggac | NODE\_643947\_length\_12221\_cov\_29.509043:10372..10461:- |
| NODE\_8717\_length\_262\_cov\_456.431305\_917 | 3.5e+1 | 0.74 ± 0.03 |  | 85 | 74 | 10 | 1 | no |  |  |  | blast | uaugaaugcaaugaugacaaauuu | uucuauaucgcaguuuucaaaaugaaa | uucuauaucgcaguuuucaaaaugaaauggauuacgcugguaggagccgguguaugcuucauugucuaugaaugcaaugaugacaaauuu | NODE\_8717\_length\_262\_cov\_456.431305:226..316:+ |
| NODE\_359699\_length\_7690\_cov\_24.210274\_24159 | 3.5e+1 | 0.74 ± 0.03 |  | 78 | 77 | 0 | 1 | no |  |  |  | blast | uuggcagucagucguucagaugggc | accuuugacguuugacu | accuuugacguuugacuguguuaagugugggcuagagaugaccccacgagaccuccuaaguuggcagucagucguucagaugggc | NODE\_359699\_length\_7690\_cov\_24.210274:3565..3650:+ |
| NODE\_708941\_length\_7715\_cov\_29.285158\_42344 | 3.4e+1 | 0.74 ± 0.03 |  | 74 | 70 | 3 | 1 | no |  |  |  | blast | uggacgagaaugggcgccgaggc | acuccagcgucugaggcguccaccucc | acuccagcgucugaggcguccaccuccacaaugaacugccgguugagaucgggguggacgagaaugggcgccgaggc | NODE\_708941\_length\_7715\_cov\_29.285158:521..598:- |
| NODE\_737847\_length\_83181\_cov\_28.914331\_43400 | 3.4e+1 | 0.74 ± 0.03 |  | 67 | 64 | 0 | 3 | yes |  |  |  | blast | uucacggagcgagcuaaguccu | ggaacuuagccacugugaac | ggaacuuagccacugugaacacaggacuucacugaacuguucacggagcgagcuaaguccu | NODE\_737847\_length\_83181\_cov\_28.914331:30869..30930:- |
| NODE\_137450\_length\_13921\_cov\_36.369225\_10620 | 3.4e+1 | 0.74 ± 0.03 |  | 58 | 41 | 0 | 17 | yes |  | gmo-miR-8825-5p |  | blast | uaggucugcuuuucauaaguca | cuuaugaaaagcagaccuacu | uaggucugcuuuucauaagucauguaaacuuaagacuuaugaaaagcagaccuacu | NODE\_137450\_length\_13921\_cov\_36.369225:11333..11389:+ |
| NODE\_91622\_length\_10605\_cov\_23.174635\_7465 | 3.3e+1 | 0.74 ± 0.03 |  | 61 | 38 | 0 | 23 | yes |  | abu-miR-29c-5p |  | blast | uugauuucuguaaacaguccu | ugacuguuagaagagauuaagc | ugacuguuagaagagauuaagcaacuuuuacagcauguugauuucuguaaacaguccu | NODE\_91622\_length\_10605\_cov\_23.174635:1398..1456:+ |
| NODE\_344862\_length\_14443\_cov\_27.982622\_23277 | 3.3e+1 | 0.74 ± 0.03 |  | 65 | 57 | 0 | 8 | yes |  |  |  | blast | aacguuugugaguucucagaga | ucugagaacucacaaacuucu | aacguuugugaguucucagagaagguugacgcucugagaacucacaaacuucu | NODE\_344862\_length\_14443\_cov\_27.982622:2202..2255:- |
| NODE\_618794\_length\_50945\_cov\_29.062656\_38108 | 3.1e+1 | 0.74 ± 0.03 |  | 64 | 38 | 0 | 26 | yes |  |  |  | blast | uuugaagcuuuguguagaugu | uccugaucaaagcuccaaguu | uuugaagcuuuguguagaugugacaucauaugacauccugaucaaagcuccaaguu | NODE\_618794\_length\_50945\_cov\_29.062656:32741..32797:+ |
| NODE\_660795\_length\_38565\_cov\_31.789473\_40297 | 3.1e+1 | 0.74 ± 0.03 |  | 70 | 67 | 0 | 3 | yes |  |  |  | blast | caacacacuguuuccuaacacu | uauguuagggagcagucuauu | uauguuagggagcagucuauuguguccaucaggcaacacacuguuuccuaacacu | NODE\_660795\_length\_38565\_cov\_31.789473:27899..27954:+ |
| NODE\_603144\_length\_12151\_cov\_21.547773\_37314 | 3.0e+1 | 0.74 ± 0.03 |  | 58 | 57 | 0 | 1 | yes |  |  |  | blast | acaggcuguaauuccacugagc | gcaguggagcgacggccugugg | gcaguggagcgacggccuguggagugagcuacaggcuguaauuccacugagc | NODE\_603144\_length\_12151\_cov\_21.547773:4317..4369:- |
| NODE\_770793\_length\_43385\_cov\_28.406454\_44459 | 3.0e+1 | 0.74 ± 0.03 |  | 59 | 56 | 0 | 3 | yes |  |  |  | blast | ccgguccagcccuguauggucu | gccugccaggucuggucugcacu | ccgguccagcccuguauggucuaguuugguucuguguagccugccaggucuggucugcacu | NODE\_770793\_length\_43385\_cov\_28.406454:29579..29640:- |
| NODE\_909954\_length\_60400\_cov\_30.659140\_47706 | 2.6e+1 | 0.74 ± 0.03 |  | 52 | 50 | 0 | 2 | yes |  |  |  | blast | gccaccuuuacucugauacacu | ugugucauuuguaaagguuacc | gccaccuuuacucugauacacugucuagcuguuugugugucauuuguaaagguuacc | NODE\_909954\_length\_60400\_cov\_30.659140:46257..46314:+ |
| NODE\_665744\_length\_48565\_cov\_27.044641\_40495 | 2.6e+1 | 0.74 ± 0.03 |  | 55 | 50 | 0 | 5 | no |  | abu-miR-138 |  | blast | ugcuggugaaacuugugcaacu | uugcucacaaugcuucaccugg | ugcuggugaaacuugugcaacuugggacuuguugguugcucacaaugcuucaccugg | NODE\_665744\_length\_48565\_cov\_27.044641:21887..21944:- |
| NODE\_725971\_length\_67601\_cov\_31.484993\_43027 | 2.5e+1 | 0.74 ± 0.03 |  | 47 | 42 | 0 | 5 | yes |  |  |  | blast | uuuuugacuuaaacccuccgagg | cggaggguuuaagucaaaaaa | cggaggguuuaagucaaaaaagggucccuuuuuugacuuaaacccuccgagg | NODE\_725971\_length\_67601\_cov\_31.484993:18084..18136:+ |
| NODE\_401870\_length\_15457\_cov\_29.738501\_26568 | 2.4e+1 | 0.74 ± 0.03 |  | 45 | 43 | 0 | 2 | yes |  |  |  | blast | ggggcgagagagaaagagcga | ugcucuuucucucucuccgcuu | ugcucuuucucucucuccgcuugaguggugagcagacaaggggcgagagagaaagagcga | NODE\_401870\_length\_15457\_cov\_29.738501:4230..4290:- |
| NODE\_296120\_length\_103\_cov\_285.922333\_20510 | 2.3e+1 | 0.74 ± 0.03 |  | 63 | 55 | 0 | 8 | no |  | oni-miR-10926 |  | blast | aucccagcugccaaaaacuaa | guuuucuccacuuuggaugccu | guuuucuccacuuuggaugccuccaguggguucuggcaaauaccacuggaucccagcugccaaaaacuaa | NODE\_296120\_length\_103\_cov\_285.922333:113..183:- |
| NODE\_594870\_length\_8019\_cov\_23.941639\_36893 | 2.0e+1 | 0.74 ± 0.03 |  | 40 | 38 | 1 | 1 | yes |  |  |  | blast | agucccaaaacacugguuguag | ugcaacuguuuuaaggaucuga | ugcaacuguuuuaaggaucugagaaacuacugaacacuucagucccaaaacacugguuguag | NODE\_594870\_length\_8019\_cov\_23.941639:5185..5247:+ |
| NODE\_258397\_length\_61623\_cov\_29.437498\_18324 | 1.2e+1 | 0.74 ± 0.03 |  | 48 | 46 | 0 | 2 | no |  |  |  | blast | agaaaugacuugcgaauaaagu | uuuguucccaugccauuuaugc | agaaaugacuugcgaauaaaguucucuuuaacacuuuguucccaugccauuuaugc | NODE\_258397\_length\_61623\_cov\_29.437498:25126..25182:+ |
| NODE\_699947\_length\_951\_cov\_16.044165\_41891 | 6.1 | 0.74 ± 0.03 |  | 694 | 692 | 0 | 2 | yes |  | gmo-miR-11239-3p |  | blast | cgguucugcuuggaaaacagug | uguuuuccaagcagaacugucu | cgguucugcuuggaaaacaguggugcaacacuguuuuccaagcagaacugucu | NODE\_699947\_length\_951\_cov\_16.044165:460..513:- |
| NODE\_621944\_length\_8639\_cov\_23.054867\_38371 | 5.8 | 0.75 ± 0.03 |  | 489 | 392 | 0 | 97 | yes |  | oni-miR-10619 |  | blast | aucgagagcguucugacuuacu | aguaggucagaggguucucga | aguaggucagaggguucucgaaacgucuaccgcuccaccaucgagagcguucugacuuacu | NODE\_621944\_length\_8639\_cov\_23.054867:4959..5020:+ |
| NODE\_615561\_length\_2028\_cov\_24.085798\_37866 | 5.6 | 0.75 ± 0.03 |  | 16046 | 10795 | 1 | 5250 | yes |  | abu-miR-23c |  | blast | aucacauugccagagacuacu | ugugucucuggcaagaugauu | ugugucucuggcaagaugauuugggccacaaaguugaaaucacauugccagagacuacu | NODE\_615561\_length\_2028\_cov\_24.085798:714..773:- |
| NODE\_611322\_length\_4859\_cov\_18.376415\_37712 | 5.4 | 0.75 ± 0.03 |  | 802 | 802 | 0 | 0 | yes |  | eel-miR-11054-3p |  | blast | cuacucugcaggguucuacucu | ggugcuacucugcaggguucua | ggugcuacucugcaggguucuacugugcaugguucuacucugcaggguucuacugugcaugguucuacucugcaggguucuacucu | NODE\_611322\_length\_4859\_cov\_18.376415:1951..2037:- |
| NODE\_140717\_length\_15202\_cov\_25.003815\_10831 | 5.3 | 0.75 ± 0.03 |  | 93860 | 65316 | 154 | 28390 | yes |  | abu-miR-219-5p |  | blast | ugauuguccaaacgcaauucu | agaauuguguauggacaucugu | ugauuguccaaacgcaauucuugaguaaacucauauucaaccccaagaauuguguauggacaucugu | NODE\_140717\_length\_15202\_cov\_25.003815:6403..6470:- |
| NODE\_436380\_length\_33484\_cov\_29.284464\_28341 | 5.3 | 0.75 ± 0.03 |  | 1232136 | 1222508 | 0 | 9628 | yes |  | dre-miR-338-3p |  | blast | uccagcaucagugauuuuguu | aacaacauccuggugcugccugagu | aacaacauccuggugcugccugaguaguugucacaaacuccagcaucagugauuuuguu | NODE\_436380\_length\_33484\_cov\_29.284464:22896..22955:+ |
| NODE\_111439\_length\_10491\_cov\_28.027262\_8838 | 5.3 | 0.75 ± 0.03 |  | 854951 | 763241 | 31 | 91679 | yes |  | abu-miR-22a |  | blast | aagcugccagcugaagaacu | aguucuucacuggcaagcuuu | aguucuucacuggcaagcuuuauguuucuguacacaugcuaaagcugccagcugaagaacu | NODE\_111439\_length\_10491\_cov\_28.027262:3863..3924:- |
| NODE\_207375\_length\_15949\_cov\_31.924007\_15267 | 5.3 | 0.75 ± 0.03 |  | 86 | 80 | 0 | 6 | yes |  | ccr-miR-460-5p |  | blast | ucugcauuuauuuaauccucug | uggauggauggagggau | uggauggauggagggauaaaugauaaacucucugcauuuauuuaauccucug | NODE\_207375\_length\_15949\_cov\_31.924007:11798..11850:+ |
| NODE\_784519\_length\_58360\_cov\_27.882898\_44974 | 5.2 | 0.75 ± 0.03 |  | 18260 | 15605 | 0 | 2655 | yes |  | ola-miR-31 |  | blast | aggcaagauguuggcauagcu | ugcuaugccgacaaauugccauc | aggcaagauguuggcauagcuguugaguugaaaacccugcuaugccgacaaauugccauc | NODE\_784519\_length\_58360\_cov\_27.882898:55961..56021:+ |
| NODE\_303539\_length\_39784\_cov\_31.875326\_20972 | 5.2 | 0.75 ± 0.03 |  | 37993 | 37801 | 54 | 138 | yes |  | abu-miR-19c |  | blast | ugugcaaacccaugcaaaacu | agcuuugcagggugggcagucagc | agcuuugcagggugggcagucagccugugugcauaaggaccagcugugcaaacccaugcaaaacu | NODE\_303539\_length\_39784\_cov\_31.875326:22968..23033:- |
| NODE\_122954\_length\_12001\_cov\_28.862261\_9581 | 5.2 | 0.75 ± 0.03 |  | 288181 | 287671 | 78 | 432 | yes |  | abu-miR-15a |  | blast | uagcagcacguaaauauuggc | cccaauauuagcagugcugcuu | uagcagcacguaaauauuggcgugugagaauagaccccaaccccaauauuagcagugcugcuu | NODE\_122954\_length\_12001\_cov\_28.862261:9360..9423:- |
| NODE\_568677\_length\_6138\_cov\_24.072174\_35809 | 5.2 | 0.75 ± 0.03 |  | 39 | 39 | 0 | 0 | yes |  | oni-miR-10805 |  | blast | gguuucuguaggugaaccug | ggauuaucugcagccgcccu | gguuucuguaggugaaccugucccaggauuaucugcagccgcccu | NODE\_568677\_length\_6138\_cov\_24.072174:5463..5508:- |
| NODE\_748548\_length\_19274\_cov\_26.242191\_43679 | 5.1 | 0.75 ± 0.03 |  | 87 | 44 | 0 | 43 | yes |  | ipu-miR-7563c |  | blast | auucagagacagacacauaagu | ugugucugucucuaaaucaua | ugugucugucucuaaaucauauuggguaugauucagagacagacacauaagu | NODE\_748548\_length\_19274\_cov\_26.242191:8403..8455:+ |
| NODE\_35228\_length\_12450\_cov\_30.672209\_3151 | 5.1 | 0.75 ± 0.03 |  | 1222624 | 1222432 | 0 | 192 | yes |  | dre-miR-338-3p |  | blast | uccagcaucagugauuuuguu | aacaacauccugaugcugucu | aacaacauccugaugcugucuggguauguaaagcagaacuccagcaucagugauuuuguu | NODE\_35228\_length\_12450\_cov\_30.672209:9202..9262:+ |
| NODE\_540432\_length\_66809\_cov\_33.349190\_34307 | 5.1 | 0.75 ± 0.03 |  | 55760 | 55540 | 0 | 220 | yes |  | abu-miR-19c |  | blast | ugugcaaaucuaugcaaaacu | aguuuugcguaguugcgcugc | aguuuugcguaguugcgcugcaagaagaaauaaguugugcaaaucuaugcaaaacu | NODE\_540432\_length\_66809\_cov\_33.349190:61062..61118:+ |
| NODE\_650421\_length\_14855\_cov\_31.549580\_39767 | 5.0 | 0.75 ± 0.03 |  | 348 | 266 | 0 | 82 | yes |  | oni-miR-10917 |  | blast | acagcugucagcugcacuccu | cggcaguguacugcugcuggcu | cggcaguguacugcugcuggcugugcuauuugacugacagcugucagcugcacuccu | NODE\_650421\_length\_14855\_cov\_31.549580:9229..9286:+ |
| NODE\_817731\_length\_59985\_cov\_31.449245\_45830 | 4.8 | 0.75 ± 0.03 |  | 74 | 53 | 0 | 21 | yes |  | gmo-miR-99-3p |  | blast | caaguucggaucuacggguu | uaccuauagauacgagcuugu | uaccuauagauacgagcuugugcagccagucaccacaaguucggaucuacggguu | NODE\_817731\_length\_59985\_cov\_31.449245:51836..51891:- |
| NODE\_540432\_length\_66809\_cov\_33.349190\_34311 | 4.8 | 0.75 ± 0.03 |  | 121779 | 121735 | 3 | 41 | yes |  | abu-miR-19c |  | blast | ugugcaaauccaugcaaaacu | aguuuugcugguuugcuuucagc | aguuuugcugguuugcuuucagcuuuuaaauguacugcugugcaaauccaugcaaaacu | NODE\_540432\_length\_66809\_cov\_33.349190:61365..61424:+ |
| NODE\_605150\_length\_9835\_cov\_25.334520\_37357 | 4.7 | 0.75 ± 0.03 |  | 20743 | 19729 | 10 | 1004 | yes |  | ccr-miR-130a |  | blast | uagugcaauauugcuuauagggu | ccccuaucaguauugccucugc | ccccuaucaguauugccucugcuuucacuagaguuaacagaguagugcaauauugcuuauagggu | NODE\_605150\_length\_9835\_cov\_25.334520:5088..5153:+ |
| NODE\_615561\_length\_2028\_cov\_24.085798\_37862 | 4.6 | 0.75 ± 0.03 |  | 319640 | 319628 | 0 | 12 | yes |  | ipu-miR-24 |  | blast | uggcucaguucagcaggaac | gugcuuucugagcugacagc | gugcuuucugagcugacagcaguuugaucaaauacacuggcucaguucagcaggaac | NODE\_615561\_length\_2028\_cov\_24.085798:249..306:- |
| NODE\_608704\_length\_15335\_cov\_28.955200\_37592 | 4.5 | 0.75 ± 0.03 |  | 113 | 113 | 0 | 0 | yes |  | ssa-miR-462b-3p |  | blast | ucuggaugucaaccugccuugc | aggcagguugacagcuaaaag | aggcagguugacagcuaaaagcacaggaauacuuuaagucuggaugucaaccugccuugc | NODE\_608704\_length\_15335\_cov\_28.955200:8427..8487:- |
| NODE\_843781\_length\_10278\_cov\_32.790329\_46551 | 4.5 | 0.75 ± 0.03 |  | 65 | 65 | 0 | 0 | yes |  | ssa-miR-1-5p |  | blast | ucauacuuuguagggucuuguc | agggacuagacaaaaugaau | ucauacuuuguagggucuugucugcauuuauuguagagggacuagacaaaaugaau | NODE\_843781\_length\_10278\_cov\_32.790329:5546..5602:- |
| NODE\_73664\_length\_7116\_cov\_25.140388\_6086 | 4.5 | 0.75 ± 0.03 |  | 77 | 77 | 0 | 0 | yes |  | oni-miR-10811 |  | blast | augugauugaaguccucuua | agaguuugucauuuuua | augugauugaaguccucuuaccugggugugcaggcgcaguagugcccugcucuguaguaagaguuugucauuuuua | NODE\_73664\_length\_7116\_cov\_25.140388:3629..3705:+ |
| NODE\_187593\_length\_9863\_cov\_32.301937\_13885 | 4.4 | 0.75 ± 0.03 |  | 157 | 157 | 0 | 0 | yes |  | oni-miR-10949 |  | blast | acauggaaaugcugucaucucg | cgaugccaccacuuccaccguca | acauggaaaugcugucaucucgugccaucccucggcaauauucccgcgaugccaccacuuccaccguca | NODE\_187593\_length\_9863\_cov\_32.301937:1368..1437:- |
| NODE\_70528\_length\_3614\_cov\_31.043995\_5773 | 4.4 | 0.75 ± 0.03 |  | 1042 | 998 | 0 | 44 | yes |  | gmo-miR-459-5p |  | blast | ucaguaacaaggauucauccu | aggaaaucacuguuacuggagg | ucaguaacaaggauucauccuguugugcugcagacucacaggaaaucacuguuacuggagg | NODE\_70528\_length\_3614\_cov\_31.043995:2670..2731:- |
| NODE\_49787\_length\_7431\_cov\_25.815369\_4324 | 4.1 | 0.75 ± 0.03 |  | 332 | 308 | 0 | 24 | yes |  | gmo-miR-11238-5p |  | blast | augcaucugucuuaauugcucu | agagcauaagagagacguguagg | agagcauaagagagacguguagguagucauaguccaugcaucugucuuaauugcucu | NODE\_49787\_length\_7431\_cov\_25.815369:6181..6238:- |
| NODE\_809330\_length\_50824\_cov\_32.210808\_45623 | 3.9 | 0.70 ± 0.03 |  | 138 | 133 | 0 | 5 | yes |  | ssa-miR-430a-5p |  | blast | caccugaauuacucugucuuagu | gcagacaguuuuuucaggacu | caccugaauuacucugucuuaguuuuaucugugcagacaguuuuuucaggacu | NODE\_809330\_length\_50824\_cov\_32.210808:11815..11868:- |
| NODE\_470352\_length\_38440\_cov\_32.599663\_30091 | 3.7 | 0.70 ± 0.03 |  | 144 | 143 | 0 | 1 | yes |  | dre-miR-7148-5p |  | blast | cuggaaaugauuucuuaggcuu | aagucaaggauuauuuccgagc | cuggaaaugauuucuuaggcuuauuauaaaaacuuuuaauuaagucaaggauuauuuccgagc | NODE\_470352\_length\_38440\_cov\_32.599663:29628..29691:- |
| NODE\_373244\_length\_60022\_cov\_29.876278\_25069 | 3.5 | 0.70 ± 0.03 |  | 49592 | 49582 | 10 | 0 | yes |  | abu-miR-22a |  | blast | aagcugccaguugaagagcugu | agcucuccacagagggcagugagg | aagcugccaguugaagagcuguuguguguaaccaugacaacaaagcucuccacagagggcagugagg | NODE\_373244\_length\_60022\_cov\_29.876278:24180..24247:- |
| NODE\_340632\_length\_45962\_cov\_28.372873\_23050 | 2.8 | 0.69 ± 0.03 |  | 162 | 150 | 0 | 12 | yes |  |  |  | blast | accgaucuaaucugaauguga | ucacauucagauuagaucgguu | ucacauucagauuagaucgguuacugguuaaccgaucuaaucugaauguga | NODE\_340632\_length\_45962\_cov\_28.372873:37624..37675:- |
| NODE\_340632\_length\_45962\_cov\_28.372873\_23047 | 2.7 | 0.69 ± 0.03 |  | 162 | 150 | 0 | 12 | yes |  |  |  | blast | accgaucuaaucugaauguga | ucacauucagauuagaucgguu | ucacauucagauuagaucgguuaaccaguaaccgaucuaaucugaauguga | NODE\_340632\_length\_45962\_cov\_28.372873:37624..37675:+ |
| NODE\_518263\_length\_30190\_cov\_33.180458\_33084 | 2.7 | 0.69 ± 0.03 |  | 197 | 120 | 0 | 77 | yes |  |  |  | blast | aguuuacugagauguccacugc | uggacaucucaguaaacucaga | aguuuacugagauguccacugcuuagaucgcuaagcaguggacaucucaguaaacucaga | NODE\_518263\_length\_30190\_cov\_33.180458:25375..25435:+ |
| NODE\_518263\_length\_30190\_cov\_33.180458\_33085 | 2.6 | 0.69 ± 0.03 |  | 197 | 120 | 0 | 77 | yes |  |  |  | blast | aguuuacugagauguccacugc | uggacaucucaguaaacucaga | aguuuacugagauguccacugcuuagcgaucuaagcaguggacaucucaguaaacucaga | NODE\_518263\_length\_30190\_cov\_33.180458:25371..25431:- |
| NODE\_491503\_length\_18414\_cov\_24.107582\_31473 | 2.5 | 0.69 ± 0.03 |  | 622 | 586 | 0 | 36 | yes |  |  |  | blast | acaagccgaaaugcguugguca | ccaacgcguuucagcuuguggccu | ccaacgcguuucagcuuguggccuucaucagggucaucacauucggccacaagccgaaaugcguugguca | NODE\_491503\_length\_18414\_cov\_24.107582:6689..6759:+ |
| NODE\_28835\_length\_510\_cov\_45.484314\_2698 | 2.3 | 0.69 ± 0.03 |  | 49 | 49 | 0 | 0 | yes |  |  |  | blast | uaaccaucauggugucacuga | agugacaccaugaugguuaca | uaaccaucauggugucacugauauuuccaucagugacaccaugaugguuaca | NODE\_28835\_length\_510\_cov\_45.484314:342..394:+ |
| NODE\_491503\_length\_18414\_cov\_24.107582\_31475 | 2.2 | 0.69 ± 0.03 |  | 434 | 339 | 0 | 95 | yes |  |  |  | blast | ucucuuacauuaucugaacccu | aggguucagauaauguaagaga | aggguucagauaauguaagagagcgagcugagcucucuuacauuaucugaacccu | NODE\_491503\_length\_18414\_cov\_24.107582:11481..11536:+ |
| NODE\_104176\_length\_12051\_cov\_32.732635\_8288 | 2.1 | 0.69 ± 0.03 |  | 761 | 754 | 5 | 2 | yes |  |  |  | blast | uccucggcuggcagcgcgagcucc | gcugcuccagcuccggcucgcugagc | gcugcuccagcuccggcucgcugagcugcuccagcuccggcuccggcucgcugagcugcuccucggcuggcagcgcgagcucc | NODE\_104176\_length\_12051\_cov\_32.732635:2741..2824:- |
| NODE\_491503\_length\_18414\_cov\_24.107582\_31478 | 2.1 | 0.69 ± 0.03 |  | 409 | 323 | 0 | 86 | yes |  |  |  | blast | ucucuuacauuaucugaacccu | aggguucagauaauguaagaga | aggguucagauaauguaagagagcucagcucgcucucuuacauuaucugaacccu | NODE\_491503\_length\_18414\_cov\_24.107582:11481..11536:- |
| NODE\_536934\_length\_13540\_cov\_21.433235\_34100 | 2.1 | 0.69 ± 0.03 |  | 278 | 128 | 0 | 150 | yes |  |  |  | blast | ugucagcgucauggcgucgcucc | ccgacuccacggugcugacauc | ugucagcgucauggcgucgcuccgucucagugaccaggccgacuccacggugcugacauc | NODE\_536934\_length\_13540\_cov\_21.433235:4193..4253:+ |
| NODE\_401063\_length\_41881\_cov\_29.225328\_26505 | 2.1 | 0.69 ± 0.03 |  | 43 | 40 | 0 | 3 | yes |  |  |  | blast | aaacccaauucaaagcgucggu | uccgacgcuuugaauuggguuu | uccgacgcuuugaauuggguuugaugaucucaucaaacccaauucaaagcgucggu | NODE\_401063\_length\_41881\_cov\_29.225328:10336..10392:+ |
| NODE\_553093\_length\_9114\_cov\_26.603907\_34863 | 2.0 | 0.69 ± 0.03 |  | 150 | 143 | 4 | 3 | yes |  |  |  | blast | ugugugcacaaaucugauuggu | accaaucagauguguucacaga | accaaucagauguguucacagacgugugugugugugugugcacaaaucugauuggu | NODE\_553093\_length\_9114\_cov\_26.603907:7276..7332:+ |
| NODE\_413017\_length\_12136\_cov\_28.515326\_27146 | 2.0 | 0.69 ± 0.03 |  | 119 | 119 | 0 | 0 | yes |  |  |  | blast | auguugcaaaauguaagagcuu | gcucucacauuuugcaagauau | auguugcaaaauguaagagcuuguaaaauguaagauucuacaagcucucacauuuugcaagauau | NODE\_413017\_length\_12136\_cov\_28.515326:9453..9518:+ |
| NODE\_809330\_length\_50824\_cov\_32.210808\_45624 | 2.0 | 0.69 ± 0.03 |  | 133 | 133 | 0 | 0 | yes |  | ssa-miR-430a-5p |  | blast | caccugaauuacucugucuuagu | ugugacccaggauggcccucagcugaa | ugugacccaggauggcccucagcugaaaugcacaccugcuuucaccugaauuacucugucuuagu | NODE\_809330\_length\_50824\_cov\_32.210808:11845..11910:- |
| NODE\_564439\_length\_22142\_cov\_28.304941\_35459 | 1.9 | 0.71 ± 0.03 |  | 181 | 180 | 0 | 1 | yes |  |  |  | blast | gacugcucgagugggaaaucu | aggcuccccccgagucagugucg | gacugcucgagugggaaaucucccguggggcacauuaccggaggcuccccccgagucagugucg | NODE\_564439\_length\_22142\_cov\_28.304941:6053..6117:- |
| NODE\_912144\_length\_26918\_cov\_26.152203\_47740 | 1.9 | 0.71 ± 0.03 |  | 255 | 251 | 0 | 4 | yes |  |  |  | blast | uuuggaugcuugggaauucuca | agaauucccaagcauccaaa | uuuggaugcuugggaauucucaacgucacacugagaauucccaagcauccaaa | NODE\_912144\_length\_26918\_cov\_26.152203:25022..25075:+ |
| NODE\_1092385\_length\_20468\_cov\_23.397108\_48913 | 1.9 | 0.71 ± 0.03 |  | 82 | 81 | 1 | 0 | yes |  |  |  | blast | uccuugcuguuccucugucuccu | gggagagaggaggagaggaug | gggagagaggaggagaggaugaaauaaaagugcugaauccuugcuguuccucugucuccu | NODE\_1092385\_length\_20468\_cov\_23.397108:1986..2046:+ |
| NODE\_554860\_length\_10112\_cov\_18.971716\_35010 | 1.9 | 0.71 ± 0.03 |  | 224 | 221 | 0 | 3 | yes |  |  |  | blast | uuccuccuuucuuucugucucc | acagguggcugaggaggaagag | uuccuccuuucuuucugucucccuccuccuccccugggacagguggcugaggaggaagag | NODE\_554860\_length\_10112\_cov\_18.971716:3172..3232:+ |
| NODE\_172166\_length\_9313\_cov\_27.678514\_12970 | 1.8 | 0.71 ± 0.03 |  | 70 | 69 | 0 | 1 | yes |  |  |  | blast | ugucacacagcagcuguucuga | cagcguuggcaugacucc | cagcguuggcaugacuccuaucaccgugucacacagcagcuguucuga | NODE\_172166\_length\_9313\_cov\_27.678514:5411..5459:- |
| NODE\_529517\_length\_57822\_cov\_27.030973\_33731 | 1.8 | 0.71 ± 0.03 |  | 51 | 51 | 0 | 0 | yes |  |  |  | blast | uuugaaacuagagaauuuucau | gaaaauucucuaguuucaagau | uuugaaacuagagaauuuucauuuuccccuuauuucaagguaaaauaucuugaugaugaaacaagugaaaauucucuaguuucaagau | NODE\_529517\_length\_57822\_cov\_27.030973:27130..27218:- |
| NODE\_138223\_length\_3327\_cov\_20.481815\_10680 | 1.8 | 0.71 ± 0.03 |  | 152 | 150 | 0 | 2 | yes |  |  |  | blast | acacuuguuuauuggcugggccu | caucugggcaaacacauuugggg | acacuuguuuauuggcugggccugauuuggggcccauccagggcccuucauuagcccaucugggcaaacacauuugggg | NODE\_138223\_length\_3327\_cov\_20.481815:2020..2099:+ |
| NODE\_315490\_length\_4860\_cov\_26.403498\_21637 | 1.8 | 0.71 ± 0.03 |  | 93 | 92 | 0 | 1 | yes |  |  |  | blast | uccugggcuuuguaguagcugcu | cgaggccuggaggugca | uccugggcuuuguaguagcugcucacuggauuagugugcagcucacgaggccuggaggugca | NODE\_315490\_length\_4860\_cov\_26.403498:4709..4771:- |
| NODE\_426211\_length\_36411\_cov\_26.205130\_27960 | 1.8 | 0.71 ± 0.03 |  | 239 | 181 | 0 | 58 | yes |  |  |  | blast | uugguaauuuggagaacucauu | aucgaguucuccaaguuac | aucgaguucuccaaguuaccaaaauaggcagccauuuugguaauuuggagaacucauu | NODE\_426211\_length\_36411\_cov\_26.205130:20524..20582:- |
| NODE\_65804\_length\_2618\_cov\_27.849886\_5444 | 1.8 | 0.71 ± 0.03 |  | 126 | 92 | 0 | 34 | yes |  |  |  | blast | augacgagagcagugcugugaga | acaacacugcucucguuucugu | augacgagagcagugcugugagauauuucucacaacacugcucucguuucugu | NODE\_65804\_length\_2618\_cov\_27.849886:2220..2273:+ |
| NODE\_490034\_length\_41039\_cov\_27.471210\_31256 | 1.7 | 0.71 ± 0.03 |  | 481 | 478 | 0 | 3 | yes |  |  |  | blast | ccacugcaacagcaacuguag | ugcugcugcugccgcca | ugcugcugcugccgccaccgcaacugccacugcaacagcaacuguag | NODE\_490034\_length\_41039\_cov\_27.471210:26579..26626:- |
| NODE\_330600\_length\_4189\_cov\_21.100979\_22622 | 1.7 | 0.71 ± 0.03 |  | 64 | 63 | 1 | 0 | yes |  |  |  | blast | acuccagggauuagaucgagcc | cucuggcuaaacucuggauuaa | acuccagggauuagaucgagcccacagcugagggccuggcucuggcuaaacucuggauuaa | NODE\_330600\_length\_4189\_cov\_21.100979:430..491:+ |
| NODE\_725652\_length\_22260\_cov\_24.279514\_43004 | 1.7 | 0.71 ± 0.03 |  | 166 | 163 | 0 | 3 | yes |  |  |  | blast | acugagcugcuggauuaagaga | ccucucaagccagcaguuccuc | acugagcugcuggauuaagagacgauggauguuuggccucucaagccagcaguuccuc | NODE\_725652\_length\_22260\_cov\_24.279514:15675..15733:+ |
| NODE\_10193\_length\_19426\_cov\_32.763821\_1048 | 1.7 | 0.71 ± 0.03 |  | 213 | 211 | 0 | 2 | yes |  |  |  | blast | ucggacuauaaggcgcacuguc | uaagugcgccuuauaguccaaaa | ucggacuauaaggcgcacugucgauuuuggagauuuuaagugcgccuuauaguccaaaa | NODE\_10193\_length\_19426\_cov\_32.763821:18529..18588:+ |
| NODE\_319655\_length\_177\_cov\_1756.073486\_22050 | 1.7 | 0.71 ± 0.03 |  | 5872 | 5870 | 0 | 2 | yes |  |  |  | blast | cgguuucucgaacccucgccc | ggcgacggggccccguc | ggcgacggggccccguccggcaaacacgguuucucgaacccucgccc | NODE\_319655\_length\_177\_cov\_1756.073486:69..116:- |
| NODE\_105094\_length\_1273\_cov\_27.922232\_8343 | 1.7 | 0.71 ± 0.03 |  | 302 | 171 | 0 | 131 | yes |  |  |  | blast | gcgggcucagugagcugugagg | gagcagcacccugagccucu | gagcagcacccugagccucuccgcagaccaguggaagaaggcgggcucagugagcugugagg | NODE\_105094\_length\_1273\_cov\_27.922232:923..985:+ |
| NODE\_259850\_length\_64569\_cov\_30.972200\_18468 | 1.7 | 0.71 ± 0.03 |  | 300 | 299 | 0 | 1 | yes |  |  |  | blast | uagcccuguacaaugcugcuu | aggcagcacuguaaaga | uagcccuguacaaugcugcuugaacaugauuagacaaggcagcacuguaaaga | NODE\_259850\_length\_64569\_cov\_30.972200:27613..27666:+ |
| NODE\_304026\_length\_32795\_cov\_29.007227\_21020 | 1.7 | 0.71 ± 0.03 |  | 171 | 171 | 0 | 0 | yes |  |  |  | blast | ucaguugcugcugcgcuccca | ggagcuuggcagcgguggaua | ucaguugcugcugcgcucccaguuguuauugugggagcuuggcagcgguggaua | NODE\_304026\_length\_32795\_cov\_29.007227:20247..20301:+ |
| NODE\_397862\_length\_109\_cov\_148.440369\_26332 | 1.6 | 0.71 ± 0.03 |  | 155 | 150 | 0 | 5 | yes |  |  |  | blast | acacuuguuuauuggcugggccu | ucugggcaaacacacuuggggcc | acacuuguuuauuggcugggccugauuuggggcccauccagggcccgucauuagcccaucugggcaaacacacuuggggcc | NODE\_397862\_length\_109\_cov\_148.440369:86..167:+ |
| NODE\_792632\_length\_3993\_cov\_27.974707\_45240 | 1.6 | 0.71 ± 0.03 |  | 112 | 111 | 0 | 1 | yes |  |  |  | blast | agcucugucucugcucgcugucu | agacagcagccgagcagcacu | agacagcagccgagcagcacugcagugagcugcagcucugucucugcucgcugucu | NODE\_792632\_length\_3993\_cov\_27.974707:2788..2844:- |
| NODE\_448543\_length\_9120\_cov\_24.827522\_29026 | 1.6 | 0.71 ± 0.03 |  | 85 | 84 | 1 | 0 | yes |  |  |  | blast | augcaaucgagaagcuaaugaaucu | auuggggagcuggcggcgcugcucugg | auuggggagcuggcggcgcugcucugggccugggcgccauccccacagguguggagcucagaguaccaugcaaucgagaagcuaaugaaucu | NODE\_448543\_length\_9120\_cov\_24.827522:4404..4496:- |
| NODE\_313327\_length\_28454\_cov\_31.164862\_21547 | 1.6 | 0.71 ± 0.03 |  | 364 | 347 | 0 | 17 | yes |  |  |  | blast | ugaccagagucugaaaaccuc | ugcguuuuccucucugguuuug | ugaccagagucugaaaaccuccacuuugucaguuugugcguuuuccucucugguuuug | NODE\_313327\_length\_28454\_cov\_31.164862:9967..10025:- |
| NODE\_154587\_length\_62671\_cov\_27.122370\_11693 | 1.6 | 0.71 ± 0.03 |  | 64 | 58 | 0 | 6 | yes |  |  |  | blast | ucugggcgaccuggcuaaaggu | uuuuggucagcggcgccacggcu | uuuuggucagcggcgccacggcuuauauugccuuaaaacgucugggcgaccuggcuaaaggu | NODE\_154587\_length\_62671\_cov\_27.122370:8011..8073:+ |
| NODE\_108120\_length\_2051\_cov\_19.742565\_8594 | 1.6 | 0.71 ± 0.03 |  | 2037 | 2036 | 0 | 1 | yes |  |  |  | blast | ugaccccgccugcacagaggga | ucugugucagugaguuuagga | ugaccccgccugcacagagggaguuguggacauccaguucugugucagugaguuuagga | NODE\_108120\_length\_2051\_cov\_19.742565:554..613:+ |
| NODE\_290679\_length\_20662\_cov\_26.437906\_20248 | 1.6 | 0.71 ± 0.03 |  | 407 | 407 | 0 | 0 | yes |  |  |  | blast | cagcucugcagcucugcagcu | cugcuuuagaguccagacacag | cugcuuuagaguccagacacagcgcugcagcucugcagcgcugcagcucugcagcucugcagcu | NODE\_290679\_length\_20662\_cov\_26.437906:19308..19372:- |
| NODE\_300514\_length\_63425\_cov\_33.798157\_20754 | 1.6 | 0.71 ± 0.03 |  | 129 | 129 | 0 | 0 | yes |  |  |  | blast | agaagcauaggccaucagcu | cuaauguccuauucuuucag | agaagcauaggccaucagcuaacauuugcugacauuuagcuaauguccuauucuuucag | NODE\_300514\_length\_63425\_cov\_33.798157:9283..9342:+ |
| NODE\_224262\_length\_11070\_cov\_25.794489\_16259 | 1.6 | 0.71 ± 0.03 |  | 99 | 99 | 0 | 0 | yes |  |  |  | blast | acaucugucacacgagcucagg | ugagcacaugugacagaugcca | ugagcacaugugacagaugccacagcuucuccagacucucucacaucugucacacgagcucagg | NODE\_224262\_length\_11070\_cov\_25.794489:1814..1878:+ |
| NODE\_623838\_length\_24971\_cov\_25.840694\_38454 | 1.6 | 0.71 ± 0.03 |  | 62 | 62 | 0 | 0 | yes |  |  |  | blast | ucccucgcucucucucuggagu | gcgcagagagagggggaggcagggcu | gcgcagagagagggggaggcagggcuguggucuuacgagucccucgcucucucucuggagu | NODE\_623838\_length\_24971\_cov\_25.840694:15435..15496:- |
| NODE\_395770\_length\_7962\_cov\_28.601357\_26232 | 1.6 | 0.71 ± 0.03 |  | 75 | 75 | 0 | 0 | yes |  |  |  | blast | uuuccaucccuccuuugcacuc | ggagggguagcgguggagaau | ggagggguagcgguggagaaucguaaccuccuccuuugauuuccaucccuccuuugcacuc | NODE\_395770\_length\_7962\_cov\_28.601357:4063..4124:- |
| NODE\_97416\_length\_13971\_cov\_23.819269\_7833 | 1.5 | 0.71 ± 0.03 |  | 234 | 234 | 0 | 0 | yes |  |  |  | blast | ugcucucacucugcuccgccu | gcagugcagaguggagcuga | gcagugcagaguggagcugagugccuuccuuuucugcucucacucugcuccgccu | NODE\_97416\_length\_13971\_cov\_23.819269:4897..4952:+ |
| NODE\_479052\_length\_25577\_cov\_27.990187\_30800 | 1.5 | 0.71 ± 0.03 |  | 21609 | 14539 | 0 | 7070 | yes |  |  |  | blast | uaggcagugucguuagcugauu | aaucacuaacuacacuaccagu | uaggcagugucguuagcugauuguucuuacacgcuaaucacuaacuacacuaccagu | NODE\_479052\_length\_25577\_cov\_27.990187:4187..4244:+ |
| NODE\_246342\_length\_40918\_cov\_27.697321\_17777 | 1.5 | 0.71 ± 0.03 |  | 525 | 525 | 0 | 0 | yes |  |  |  | blast | uccuauguccaguggaauaucugc | ggcuguucccuggaugagguca | ggcuguucccuggaugaggucaagcucuccuauguccaguggaauaucugc | NODE\_246342\_length\_40918\_cov\_27.697321:27763..27814:+ |
| NODE\_109698\_length\_19126\_cov\_30.995766\_8719 | 1.5 | 0.71 ± 0.03 |  | 93 | 93 | 0 | 0 | yes |  |  |  | blast | ugcgguugguuaguagccuuc | cggcggcgaaaccagccgcaca | cggcggcgaaaccagccgcacauuuacgguggcgaugcugugcgguugguuaguagccuuc | NODE\_109698\_length\_19126\_cov\_30.995766:10400..10461:+ |
| NODE\_256070\_length\_11354\_cov\_27.420116\_18192 | 1.5 | 0.71 ± 0.03 |  | 422 | 422 | 0 | 0 | yes |  |  |  | blast | uggcuuccuguuuccuguga | aguuuucacaggaguccuggg | uggcuuccuguuuccugugaugcugucaguuuucacaggaguccuggg | NODE\_256070\_length\_11354\_cov\_27.420116:8270..8318:+ |
| NODE\_1018186\_length\_21796\_cov\_30.058496\_48596 | 1.5 | 0.71 ± 0.03 |  | 149 | 139 | 0 | 10 | yes |  |  |  | blast | gcagaccgacaaguauaauuau | auuauccaggucggccugaaggg | gcagaccgacaaguauaauuauuguucuuugucgauacaauuauccaggucggccugaaggg | NODE\_1018186\_length\_21796\_cov\_30.058496:379..441:+ |
| NODE\_256070\_length\_11354\_cov\_27.420116\_18190 | 1.5 | 0.71 ± 0.03 |  | 582 | 422 | 0 | 160 | yes |  |  |  | blast | uggcuuccuguuuccuguga | ucacagguuucugggaguccu | uggcuuccuguuuccugugaugcugucaguuuucacagguuucugggaguccu | NODE\_256070\_length\_11354\_cov\_27.420116:6803..6856:+ |
| NODE\_4506\_length\_838\_cov\_62.906921\_501 | 1.5 | 0.71 ± 0.03 |  | 149 | 139 | 0 | 10 | yes |  |  |  | blast | gcagaccgacaaguauaauuau | auuauccaggucggccugaaggg | gcagaccgacaaguauaauuauuguucuuugucgauacaauuauccaggucggccugaaggg | NODE\_4506\_length\_838\_cov\_62.906921:460..522:- |
| NODE\_244196\_length\_20053\_cov\_27.406773\_17522 | 1.4 | 0.71 ± 0.03 |  | 1434 | 1366 | 0 | 68 | yes |  |  |  | blast | uauauucagguggaugugugc | ucacgggucuaucugaaagcuc | uauauucagguggaugugugcaguauauguaaauucacgggucuaucugaaagcuc | NODE\_244196\_length\_20053\_cov\_27.406773:17149..17205:+ |
| NODE\_91705\_length\_15181\_cov\_29.275410\_7485 | 1.4 | 0.71 ± 0.03 |  | 473 | 472 | 0 | 1 | yes |  |  |  | blast | gacuggaggacagagugaucccu | cuccccuccuuccaaga | gacuggaggacagagugaucccugauaagaugagcagggagcuccuccccuccuuccaaga | NODE\_91705\_length\_15181\_cov\_29.275410:10892..10953:- |
| NODE\_285709\_length\_1972\_cov\_25.332150\_19958 | 1.4 | 0.71 ± 0.03 |  | 390 | 390 | 0 | 0 | yes |  |  |  | blast | uuagaaacgacugcaucugugugc | acgagaugaguuauaugacc | uuagaaacgacugcaucugugugcugacgagaugaguuauaugacc | NODE\_285709\_length\_1972\_cov\_25.332150:36..82:+ |
| NODE\_663669\_length\_26565\_cov\_22.492151\_40441 | 1.4 | 0.71 ± 0.03 |  | 386 | 386 | 0 | 0 | yes |  |  |  | blast | agaggguggugagauucugaca | ucagugguuaccucccuguuu | agaggguggugagauucugacacgucuuugagcugucagugguuaccucccuguuu | NODE\_663669\_length\_26565\_cov\_22.492151:3622..3678:- |
| NODE\_225692\_length\_41595\_cov\_27.980598\_16355 | 1.4 | 0.71 ± 0.03 |  | 2979 | 2976 | 3 | 0 | yes |  |  |  | blast | aaagcugugaggcccugcu | aagggccauaacagccagca | aagggccauaacagccagcacuacuuguuauucuuuggucuucuuguaguucuauaaagcugugaggcccugcu | NODE\_225692\_length\_41595\_cov\_27.980598:912..986:+ |
| NODE\_246122\_length\_32846\_cov\_26.905712\_17747 | 1.3 | 0.71 ± 0.03 |  | 76 | 76 | 0 | 0 | yes |  |  |  | blast | uuugauguuaacccuccccagg | uggggggggugacaugcauaug | uggggggggugacaugcauauguuuaaaauguaucacacuuaaguuugauguuaacccuccccagg | NODE\_246122\_length\_32846\_cov\_26.905712:1517..1583:+ |
| NODE\_543893\_length\_2537\_cov\_27.462357\_34468 | 1.3 | 0.71 ± 0.03 |  | 164 | 163 | 0 | 1 | yes |  |  |  | blast | uaacucagacgaacaggaggccgcc | cguucauuugggucagg | cguucauuugggucaggugccaucuaauaacucagacgaacaggaggccgcc | NODE\_543893\_length\_2537\_cov\_27.462357:387..439:- |
| NODE\_178059\_length\_3177\_cov\_28.171230\_13437 | 1.3 | 0.71 ± 0.03 |  | 164 | 163 | 0 | 1 | yes |  |  |  | blast | uaacucagacgaacaggaggccgcc | cguucauuugggucagg | cguucauuugggucaggugccaucuaauaacucagacgaacaggaggccgcc | NODE\_178059\_length\_3177\_cov\_28.171230:2302..2354:+ |
| NODE\_251831\_length\_7899\_cov\_21.237879\_17982 | 1.3 | 0.71 ± 0.03 |  | 164 | 163 | 0 | 1 | yes |  |  |  | blast | uaacucagacgaacaggaggccgcc | cguucauuugggucagg | cguucauuugggucaggugccaucuaauaacucagacgaacaggaggccgcc | NODE\_251831\_length\_7899\_cov\_21.237879:1532..1584:- |
| NODE\_347227\_length\_31694\_cov\_26.835678\_23466 | 1.3 | 0.71 ± 0.03 |  | 164 | 164 | 0 | 0 | yes |  |  |  | blast | uuaggcuucauccaugguugga | cagucacggauuuagccuggac | cagucacggauuuagccuggaccuuaaaauauugaauuuuuaggcuucauccaugguugga | NODE\_347227\_length\_31694\_cov\_26.835678:22426..22487:- |
| NODE\_790385\_length\_112381\_cov\_31.130333\_45143 | 1.3 | 0.71 ± 0.03 |  | 66 | 66 | 0 | 0 | yes |  |  |  | blast | ucacacuaccacugcuguuuu | aacaguggugguagugugacg | aacaguggugguagugugacggucuguacaucacacuaccacugcuguuuu | NODE\_790385\_length\_112381\_cov\_31.130333:62414..62465:+ |
| NODE\_749175\_length\_25680\_cov\_29.913980\_43709 | 1.3 | 0.71 ± 0.03 |  | 227 | 218 | 0 | 9 | yes |  |  |  | blast | augaaguccuuggauccagugcu | ugcagugguaccugacuuccu | augaaguccuuggauccagugcuagaccaacacuggugcagugguaccugacuuccu | NODE\_749175\_length\_25680\_cov\_29.913980:11527..11584:- |
| NODE\_690780\_length\_21147\_cov\_22.963305\_41552 | 1.3 | 0.71 ± 0.03 |  | 354 | 354 | 0 | 0 | yes |  |  |  | blast | uaugcaccuggcguaugcaccu | gcguaugcaccuggcguaug | gcguaugcaccuggcguaugcaccuggcguaugcaccuggcguaugcaccu | NODE\_690780\_length\_21147\_cov\_22.963305:19395..19446:- |
| NODE\_261354\_length\_35573\_cov\_29.768814\_18638 | 1.3 | 0.71 ± 0.03 |  | 8606 | 8606 | 0 | 0 | yes |  |  |  | blast | uugauuuuuggaacugg | gguucuguuaaug | gguucuguuaaugauuguuggugauugauuuuuggaacugg | NODE\_261354\_length\_35573\_cov\_29.768814:18155..18196:- |
| NODE\_74936\_length\_2081\_cov\_22.481018\_6186 | 1.2 | 0.71 ± 0.03 |  | 130 | 130 | 0 | 0 | yes |  |  |  | blast | caggacaugugagcgccggucu | gcugugcgaacacgugugccagguu | gcugugcgaacacgugugccagguuuaacauaaaaauaacaggacaugugagcgccggucu | NODE\_74936\_length\_2081\_cov\_22.481018:645..706:- |
| NODE\_463564\_length\_6102\_cov\_27.234840\_29819 | 1.2 | 0.71 ± 0.03 |  | 1361 | 1361 | 0 | 0 | yes |  |  |  | blast | gugcagaucuugguggu | uccaagagguggcu | gugcagaucuuggugguagcagcaacuauuccaagagguggcu | NODE\_463564\_length\_6102\_cov\_27.234840:4689..4732:- |
| NODE\_1005605\_length\_18789\_cov\_25.052372\_48534 | 1.2 | 0.71 ± 0.03 |  | 191 | 190 | 1 | 0 | yes |  |  |  | blast | ccaagaugugaaguuugccacu | ugguggacuuccacuugguu | ccaagaugugaaguuugccacugcucugguguacgcucucagcuucuucugcccucacauccaagcccugguggacuuccacuugguu | NODE\_1005605\_length\_18789\_cov\_25.052372:9574..9662:+ |
| NODE\_88347\_length\_259\_cov\_60.451736\_7216 | 1.2 | 0.71 ± 0.03 |  | 6787 | 6787 | 0 | 0 | yes |  |  |  | blast | ugcagaacuacggacgcagc | ugugcguguucacagag | ugcagaacuacggacgcagcuggaauccacagcugugcguguucacagag | NODE\_88347\_length\_259\_cov\_60.451736:116..166:+ |
| NODE\_478482\_length\_24284\_cov\_26.436213\_30759 | 1.2 | 0.71 ± 0.03 |  | 699 | 503 | 0 | 196 | yes |  |  |  | blast | uuuguucuccugcacugcugu | ugugugcugcagacuaaacuagu | ugugugcugcagacuaaacuaguuuccagcugguuuguucuccugcacugcugu | NODE\_478482\_length\_24284\_cov\_26.436213:22217..22271:+ |
| NODE\_18140\_length\_4410\_cov\_26.652155\_1813 | 1.1 | 0.71 ± 0.03 |  | 88 | 74 | 0 | 14 | yes |  |  |  | blast | ugucugcacuucgaucacagcu | ugcuguggcgaaggacagagaca | ugucugcacuucgaucacagcugaugguuuaaaagcugcuguggcgaaggacagagaca | NODE\_18140\_length\_4410\_cov\_26.652155:584..643:- |
| NODE\_207640\_length\_20790\_cov\_30.320875\_15283 | 1.1 | 0.71 ± 0.03 |  | 302 | 302 | 0 | 0 | yes |  |  |  | blast | uagcccuguacaaugcugcuu | gcagcaccguaaagaggcugaa | uagcccuguacaaugcugcuugaucaagacagcacaaggcagcaccguaaagaggcugaa | NODE\_207640\_length\_20790\_cov\_30.320875:1813..1873:- |
| NODE\_453458\_length\_1942\_cov\_27.479918\_29307 | 1.1 | 0.71 ± 0.03 |  | 272 | 186 | 5 | 81 | yes |  |  |  | blast | ccgaggaggacagaggacggcu | ugacggucucuuucuuccucaca | ccgaggaggacagaggacggcuuguggucaccuguaaacacugacggucucuuucuuccucaca | NODE\_453458\_length\_1942\_cov\_27.479918:1192..1256:- |
| NODE\_422814\_length\_1454\_cov\_21.495186\_27635 | 1.1 | 0.71 ± 0.03 |  | 156 | 143 | 13 | 0 | yes |  |  |  | blast | uaucucagcucuguuacu | uggugggauaagguggg | uggugggauaaggugggccuuugagaugagcauguucugcugccaccucagggguagcuaucucagcucuguuacu | NODE\_422814\_length\_1454\_cov\_21.495186:528..604:+ |
| NODE\_175539\_length\_974\_cov\_7.546201\_13201 | 1.1 | 0.71 ± 0.03 |  | 192 | 191 | 0 | 1 | yes |  |  |  | blast | uguucugggacuguggaugcucu | agacaccgccaacccagcgcaaaaagc | uguucugggacuguggaugcucuggaaaaacaaacaaacaaacagacaccgccaacccagcgcaaaaagc | NODE\_175539\_length\_974\_cov\_7.546201:497..567:- |
| NODE\_73783\_length\_9524\_cov\_27.044939\_6098 | 1.0 | 0.71 ± 0.03 |  | 179 | 134 | 0 | 45 | yes |  |  |  | blast | ugucaaucugauucuguguguc | cacacacagauacagaggugacu | cacacacagauacagaggugacugcacaucagcuccuugucaaucugauucuguguguc | NODE\_73783\_length\_9524\_cov\_27.044939:3118..3177:- |
| NODE\_100328\_length\_37307\_cov\_31.060150\_8007 | 1.0 | 0.71 ± 0.03 |  | 2076 | 1822 | 41 | 213 | yes |  | oni-miR-10952 |  | blast | uuguaugaugaucaacaagggacu | uuuugaacuaucugaca | uuguaugaugaucaacaagggacuuaauacugaucacguaugaugucaccuuuugaacuaucugaca | NODE\_100328\_length\_37307\_cov\_31.060150:16928..16995:- |
| NODE\_608435\_length\_107138\_cov\_31.929838\_37565 | 1.0 | 0.71 ± 0.03 |  | 66 | 66 | 0 | 0 | yes |  |  |  | blast | aauggcccgagucagcgacuug | aguuggcugcccggccaaucu | aguuggcugcccggccaaucugagaaauuaggguagaauggcccgagucagcgacuug | NODE\_608435\_length\_107138\_cov\_31.929838:11500..11558:+ |
| NODE\_370933\_length\_1051\_cov\_75.604187\_24810 | 1.0 | 0.71 ± 0.03 |  | 389 | 377 | 0 | 12 | yes |  |  |  | blast | uucaggcuugucugagcagcaaca | acuguucaccaggcaucuaacu | acuguucaccaggcaucuaacuguuuuaauguccgguucaggcuugucugagcagcaaca | NODE\_370933\_length\_1051\_cov\_75.604187:162..222:- |
| NODE\_424631\_length\_3119\_cov\_27.136581\_27836 | 0.9 | 0.62 ± 0.03 |  | 34035 | 34034 | 1 | 0 | no |  | abu-miR-29d |  | blast | uagcaccauuugaaaucagugu | agcagcagugagugaguguuagg | uagcaccauuugaaaucaguguucuuggggagagcaggacagcagcagugagugaguguuagg | NODE\_424631\_length\_3119\_cov\_27.136581:2670..2733:+ |
| NODE\_241673\_length\_20865\_cov\_31.453390\_17290 | 0.9 | 0.62 ± 0.03 |  | 117 | 117 | 0 | 0 | yes |  |  |  | blast | acaguaaaaguugcgucuuacu | gaagauaaacuuuuacuuuaa | acaguaaaaguugcgucuuacugcugcaaccaaaacauuucagaagauaaacuuuuacuuuaa | NODE\_241673\_length\_20865\_cov\_31.453390:3461..3524:+ |
| NODE\_461044\_length\_42254\_cov\_27.870947\_29683 | 0.9 | 0.62 ± 0.03 |  | 274 | 255 | 6 | 13 | yes |  |  |  | blast | ucucucuugcaacagucccacu | acuaugggagagauacaaau | ucucucuugcaacagucccacuagcaacaccugggcuuugugggauuacuaugggagagauacaaau | NODE\_461044\_length\_42254\_cov\_27.870947:29057..29124:- |
| NODE\_437235\_length\_49973\_cov\_29.757549\_28394 | 0.9 | 0.62 ± 0.03 |  | 288 | 163 | 0 | 125 | yes |  |  |  | blast | augagcaagguaacuugccggacc | accuacgcucaaacacuc | augagcaagguaacuugccggaccugcagugguccggcugggaaaccuacgcucaaacacuc | NODE\_437235\_length\_49973\_cov\_29.757549:27160..27222:+ |
| NODE\_361009\_length\_12352\_cov\_25.616661\_24304 | 0.9 | 0.62 ± 0.03 |  | 475 | 475 | 0 | 0 | yes |  |  |  | blast | ccauucagaaugccagagccuccuc | ggaggcuucacgngaagguu | ggaggcuucacgngaagguuauugaaaccauucagaaugccagagccuccuc | NODE\_361009\_length\_12352\_cov\_25.616661:1923..1975:- |
| NODE\_142402\_length\_3903\_cov\_32.640789\_10930 | 0.9 | 0.62 ± 0.03 |  | 35 | 35 | 0 | 0 | yes |  |  |  | blast | ucugaaccaugagccauuuug | gaauggcccauugggcaggag | gaauggcccauugggcaggaggagaaacguucuguuguuucugaaccaugagccauuuug | NODE\_142402\_length\_3903\_cov\_32.640789:962..1022:- |
| NODE\_243582\_length\_3390\_cov\_12.560767\_17464 | 0.8 | 0.62 ± 0.03 |  | 79 | 48 | 31 | 0 | yes |  |  |  | blast | uuugaacgcauggcuguaguuugcc | caaagcaagaagugcguaug | uuugaacgcauggcuguaguuugccaguggcaaaaauggacuggguuuuucaucugauuccuuuacucacuggcaaagcaagaagugcguaug | NODE\_243582\_length\_3390\_cov\_12.560767:415..508:+ |
| NODE\_709020\_length\_35243\_cov\_28.810913\_42350 | 0.8 | 0.62 ± 0.03 |  | 109 | 109 | 0 | 0 | yes |  |  |  | blast | cagaccucugugauuggcucgc | aggcuagccacacugguaugcg | aggcuagccacacugguaugcgcucguacauuuacaagcagaccucugugauuggcucgc | NODE\_709020\_length\_35243\_cov\_28.810913:15262..15322:- |
| NODE\_412784\_length\_6493\_cov\_22.596334\_27124 | 0.8 | 0.62 ± 0.03 |  | 60 | 56 | 0 | 4 | no |  | ipu-miR-7565 |  | blast | uuccugcugaacugagccag | acagcaggcacaggaga | uuccugcugaacugagccagccgacgucacacacugauuaucagcacagcaggcacaggaga | NODE\_412784\_length\_6493\_cov\_22.596334:784..846:+ |
| NODE\_476986\_length\_11166\_cov\_24.496597\_30583 | 0.8 | 0.62 ± 0.03 |  | 125 | 124 | 0 | 1 | yes |  |  |  | blast | gaagcauccugagagcuguaga | gccacaguucucagugugcucu | gccacaguucucagugugcucugauaaaacugaagcauccugagagcuguaga | NODE\_476986\_length\_11166\_cov\_24.496597:4208..4261:+ |
| NODE\_91757\_length\_737\_cov\_10.583447\_7493 | 0.8 | 0.62 ± 0.03 |  | 194 | 194 | 0 | 0 | yes |  |  |  | blast | auguccagaaccugaaguc | ccuggucuuuggacugcu | ccuggucuuuggacugcuuguggaugacaaucaacccacucauucacaauauguccagaaccugaaguc | NODE\_91757\_length\_737\_cov\_10.583447:538..607:+ |
| NODE\_707066\_length\_67983\_cov\_30.521925\_42154 | 0.8 | 0.62 ± 0.03 |  | 201 | 183 | 0 | 18 | yes |  |  |  | blast | cauaaguguacugugugguagu | ucacacugugcauuuuuacguu | ucacacugugcauuuuuacguuugauauauuugaucuucagaugcauaaguguacugugugguagu | NODE\_707066\_length\_67983\_cov\_30.521925:25173..25239:- |
| NODE\_37923\_length\_1659\_cov\_1726.063843\_3336 | 0.8 | 0.62 ± 0.03 |  | 276 | 274 | 2 | 0 | no |  | oni-miR-10602 |  | blast | uccgagaagaccgaaccccggcg | ccgguauuuagccuuagauggagu | ccgguauuuagccuuagauggaguuuaccacccacuuugggcugcauucccaaacaacccgacuccgagaagaccgaaccccggcg | NODE\_37923\_length\_1659\_cov\_1726.063843:1551..1637:- |
| NODE\_810982\_length\_11566\_cov\_25.246672\_45640 | 0.8 | 0.62 ± 0.03 |  | 21535 | 21535 | 0 | 0 | yes |  |  |  | blast | accaggugcuguaagcu | cuuauucccuucuuugugu | cuuauucccuucuuuguguuuuuuccaccuagaaacaccaggugcuguaagcu | NODE\_810982\_length\_11566\_cov\_25.246672:9747..9800:- |
| NODE\_819352\_length\_6437\_cov\_28.091812\_45873 | 0.8 | 0.62 ± 0.03 |  | 1738 | 1732 | 0 | 6 | yes |  |  |  | blast | caccgguaccaugauaacuga | ucaguuaucauaguacuguacu | ucaguuaucauaguacuguacuugagagggacacagcaccgguaccaugauaacuga | NODE\_819352\_length\_6437\_cov\_28.091812:2617..2674:+ |
| NODE\_747739\_length\_50010\_cov\_30.973465\_43669 | 0.8 | 0.62 ± 0.03 |  | 126 | 122 | 0 | 4 | yes |  |  |  | blast | cucuuuuaaaagcaaguuacaccu | uucaagcuuguucauaac | uucaagcuuguucauaacuggagcuuccuggcaucaccaccagguggcucuuuuaaaagcaaguuacaccu | NODE\_747739\_length\_50010\_cov\_30.973465:12577..12648:+ |
| NODE\_658018\_length\_29345\_cov\_27.206612\_40051 | 0.7 | 0.62 ± 0.03 |  | 50 | 50 | 0 | 0 | yes |  |  |  | blast | caccaggauuaagcuguggagg | uccaugucuucuugcugag | uccaugucuucuugcugagauugggaaauauuugugacucaccaggauuaagcuguggagg | NODE\_658018\_length\_29345\_cov\_27.206612:11184..11245:+ |
| NODE\_109698\_length\_19126\_cov\_30.995766\_8720 | 0.7 | 0.62 ± 0.03 |  | 93 | 93 | 0 | 0 | yes |  |  |  | blast | ugcgguugguuaguagccuuc | aggcacuauugccucaug | ugcgguugguuaguagccuucaaacuuugaggcacuauugccucaug | NODE\_109698\_length\_19126\_cov\_30.995766:10440..10487:+ |
| NODE\_78715\_length\_30245\_cov\_32.456009\_6412 | 0.7 | 0.62 ± 0.03 |  | 490 | 443 | 0 | 47 | yes |  |  |  | blast | ucuucaaaccacugaaaucuga | acuccgguggaaugaaggaua | ucuucaaaccacugaaaucugauuagcacuuuacacagacuccgguggaaugaaggaua | NODE\_78715\_length\_30245\_cov\_32.456009:29773..29832:+ |
| NODE\_27449\_length\_11469\_cov\_26.540588\_2576 | 0.7 | 0.62 ± 0.03 |  | 2958 | 1755 | 3 | 1200 | yes |  |  |  | blast | agaagugaugagcucuucauguc | gagagaacuuuucacaacucugauc | agaagugaugagcucuucaugucucuucucugacuaaaugacuguggagagaacuuuucacaacucugauc | NODE\_27449\_length\_11469\_cov\_26.540588:2972..3043:+ |
| NODE\_72437\_length\_846\_cov\_18.249409\_5985 | 0.7 | 0.62 ± 0.03 |  | 5293 | 5293 | 0 | 0 | yes |  |  |  | blast | ugcagaacuaugaacgcagc | ugugcauguucauggag | ugcagaacuaugaacgcagcuggaauccacagcugugcauguucauggag | NODE\_72437\_length\_846\_cov\_18.249409:355..405:- |
| NODE\_148513\_length\_6111\_cov\_27.591721\_11327 | 0.7 | 0.62 ± 0.03 |  | 41 | 41 | 0 | 0 | yes |  |  |  | blast | uauguuaauaugcaugcaucacu | uggucauguguguuagcaugac | uauguuaauaugcaugcaucacugugauaacugaccaaaguggucauguguguuagcaugac | NODE\_148513\_length\_6111\_cov\_27.591721:1790..1852:- |
| NODE\_33327\_length\_1777\_cov\_33.723690\_2995 | 0.7 | 0.62 ± 0.03 |  | 5638 | 5604 | 11 | 23 | yes |  |  |  | blast | aauggugguauuguaaagcgcu | agcgcuauauaaaugcaaucc | aauggugguauuguaaagcgcuuugaggggucccgaaaagcgcuauauaaaugcaaucc | NODE\_33327\_length\_1777\_cov\_33.723690:1473..1532:- |
| NODE\_775422\_length\_5997\_cov\_30.301151\_44538 | 0.7 | 0.62 ± 0.03 |  | 33443 | 32932 | 0 | 511 | no |  | dre-miR-181a-5p |  | blast | cacauucaucgcugucgguggg | cucgccgggcaaugaaugaca | cacauucaucgcugucgguggguuaguaaugccauaaacucgccgggcaaugaaugaca | NODE\_775422\_length\_5997\_cov\_30.301151:2405..2464:+ |
| NODE\_635492\_length\_20057\_cov\_31.494041\_39049 | 0.6 | 0.62 ± 0.03 |  | 241 | 238 | 3 | 0 | yes |  |  |  | blast | gcucgacggauuguuguggacaguc | uuguccaaacaggacuugggcaa | gcucgacggauuguuguggacagucugcuugcagauugugacauuuuaugcuugcaagagacaugauuguccaaacaggacuugggcaa | NODE\_635492\_length\_20057\_cov\_31.494041:4755..4844:- |
| NODE\_145273\_length\_11012\_cov\_30.882038\_11146 | 0.6 | 0.62 ± 0.03 |  | 59 | 59 | 0 | 0 | yes |  |  |  | blast | ucagacacugcuaacuucagc | aagaguuucucaguguuuacga | aagaguuucucaguguuuacgaauguuuuuauuuguucagacacugcuaacuucagc | NODE\_145273\_length\_11012\_cov\_30.882038:5454..5511:- |
| NODE\_593635\_length\_14922\_cov\_21.954296\_36850 | 0.5 | 0.62 ± 0.03 |  | 88 | 88 | 0 | 0 | yes |  |  |  | blast | acacauguggucucuuucugca | cagugucagaugaaauggguuu | cagugucagaugaaauggguuugaguuuuucugacacauguggucucuuucugca | NODE\_593635\_length\_14922\_cov\_21.954296:9716..9771:- |
| NODE\_49653\_length\_8502\_cov\_31.480005\_4304 | 0.5 | 0.62 ± 0.03 |  | 279 | 279 | 0 | 0 | yes |  |  |  | blast | uucuugacugcagccugagcc | uuuugguuacagucaagcaagc | uucuugacugcagccugagccagcugcuccaugugguuuugguuacagucaagcaagc | NODE\_49653\_length\_8502\_cov\_31.480005:4236..4294:+ |
| NODE\_25459\_length\_335\_cov\_271.122375\_2391 | 0.5 | 0.62 ± 0.03 |  | 5627 | 5486 | 0 | 141 | yes |  |  |  | blast | uaagaaucuguggggcuuuccu | uggcaccgacagguuucauacuc | uggcaccgacagguuucauacuccuuaagaaucuguggggcuuuccu | NODE\_25459\_length\_335\_cov\_271.122375:65..112:- |
| NODE\_566122\_length\_5300\_cov\_18.131887\_35680 | 0.4 | 0.62 ± 0.03 |  | 145 | 145 | 0 | 0 | yes |  |  |  | blast | ugacacuauggcugcagcuccagc | caucacugcaccauaugcaagu | ugacacuauggcugcagcuccagcgaagguacaucacugcaccauaugcaagu | NODE\_566122\_length\_5300\_cov\_18.131887:1109..1162:+ |
| NODE\_234929\_length\_12195\_cov\_20.155474\_16924 | 0.4 | 0.62 ± 0.03 |  | 135 | 135 | 0 | 0 | yes |  |  |  | blast | aguggauuguguuuuuu | auacagcacuuccucacagu | auacagcacuuccucacaguguaguagcucacgcuagaaacacaguggauuguguuuuuu | NODE\_234929\_length\_12195\_cov\_20.155474:11527..11587:- |
| NODE\_630262\_length\_18241\_cov\_27.215340\_38944 | 0.4 | 0.62 ± 0.03 |  | 15911386 | 15911300 | 86 | 0 | no |  | abu-let-7d |  | blast | ugagguaguagguuguauaguu | cggauuaccugcaugaacggggacc | cggauuaccugcaugaacggggaccugaaaggacuuuguuuuuaaagacuguccuuuggggugagguaguagguuguauaguu | NODE\_630262\_length\_18241\_cov\_27.215340:9758..9841:- |
| NODE\_529742\_length\_5748\_cov\_29.569763\_33745 | 0.4 | 0.62 ± 0.03 |  | 7890 | 7890 | 0 | 0 | yes |  |  |  | blast | uacagaacuacgaacacagc | ugugcguguucauggaa | uacagaacuacgaacacagcuggaauccauagcugugcguguucauggaa | NODE\_529742\_length\_5748\_cov\_29.569763:1330..1380:- |
| NODE\_354914\_length\_4311\_cov\_19.208073\_23835 | 0.3 | 0.62 ± 0.03 |  | 69 | 69 | 0 | 0 | yes |  |  |  | blast | uuguuuggcaaaggcuugagc | ucaaccuuuggcaagguaaga | uuguuuggcaaaggcuugagcagcugaacacaaaauguucaaccuuuggcaagguaaga | NODE\_354914\_length\_4311\_cov\_19.208073:2216..2275:- |
| NODE\_314507\_length\_13423\_cov\_23.732996\_21594 | 0.3 | 0.62 ± 0.03 |  | 47 | 47 | 0 | 0 | yes |  |  |  | blast | cugaugauguguuguug | acaacgcaucagauuuc | acaacgcaucagauuucugguaagaugcuucagucagauucacugacugugcucagcacugaugauguguuguug | NODE\_314507\_length\_13423\_cov\_23.732996:12877..12952:- |
| NODE\_340014\_length\_279\_cov\_14.974911\_23033 | 0.3 | 0.62 ± 0.03 |  | 1743 | 1733 | 0 | 10 | yes |  |  |  | blast | uaccaugaaaaucuggacugaaauc | uaaggaccucaccucauguacgcc | uaccaugaaaaucuggacugaaaucagguaaguuacaacuuaacuuaccguaugacgguaaggaccucaccucauguacgcc | NODE\_340014\_length\_279\_cov\_14.974911:61..143:- |
| NODE\_80670\_length\_2746\_cov\_16.622360\_6578 | 0.3 | 0.62 ± 0.03 |  | 60 | 60 | 0 | 0 | yes |  |  |  | blast | cgcgcucauuggauaacgacuu | guuauuagccaaugaaugugca | cgcgcucauuggauaacgacuugucaaucgcaaguuauuagccaaugaaugugca | NODE\_80670\_length\_2746\_cov\_16.622360:2655..2710:+ |
| NODE\_15690\_length\_2771\_cov\_24.233490\_1550 | 0.2 | 0.62 ± 0.03 |  | 50 | 50 | 0 | 0 | no |  | gmo-miR-11243-3p |  | blast | ucagggagaauuuguggcaugu | gcuucugcgacagaugagugaca | ucagggagaauuuguggcauguuuguuggcgcuucugcgacagaugagugaca | NODE\_15690\_length\_2771\_cov\_24.233490:1001..1054:+ |
| NODE\_566768\_length\_28237\_cov\_26.092113\_35777 | 0.2 | 0.62 ± 0.03 |  | 138 | 131 | 1 | 6 | yes |  |  |  | blast | cacuuaguacagaucuuaucacu | agugaggucgguacuaaggcu | cacuuaguacagaucuuaucacuguauucucacauaaaucuacagugaggucgguacuaaggcu | NODE\_566768\_length\_28237\_cov\_26.092113:16100..16164:- |
| NODE\_844335\_length\_994\_cov\_17.324949\_46567 | 0.2 | 0.62 ± 0.03 |  | 359 | 353 | 0 | 6 | yes |  |  |  | blast | ugggaucuuucuuuggcuu | ugagcuaggaucuuauaauc | ugagcuaggaucuuauaaucauuauuaaggaggcaaauuggccuccaauugucaauauaagugggaucuuucuuuggcuu | NODE\_844335\_length\_994\_cov\_17.324949:451..531:+ |
| NODE\_61435\_length\_11576\_cov\_22.588200\_5171 | 0.2 | 0.62 ± 0.03 |  | 242 | 241 | 1 | 0 | yes |  |  |  | blast | uuguagaccuggaauugcucc | agcaaauccaucucacacaaca | agcaaauccaucucacacaacaugcagaaggcuuaaguuguagaccuggaauugcucc | NODE\_61435\_length\_11576\_cov\_22.588200:4206..4264:+ |
| NODE\_575362\_length\_13461\_cov\_35.437782\_36086 | 0.2 | 0.62 ± 0.03 |  | 30147 | 30145 | 2 | 0 | no |  | abu-miR-727b-3p |  | blast | guugaggcgaguugaagacu | ucccgucaacgaucagcucugcuc | guugaggcgaguugaagacuaaaaaugcuguacagauacaaggaucagcugguuugucccgucaacgaucagcucugcuc | NODE\_575362\_length\_13461\_cov\_35.437782:1679..1759:+ |
| NODE\_682202\_length\_10305\_cov\_29.894032\_41184 | 0.1 | 0.62 ± 0.03 |  | 61 | 59 | 1 | 1 | yes |  |  |  | blast | uuccuguggagcuauuaugga | ucuuguuucugcagcac | uuccuguggagcuauuauggaugggauguccauauuuauagaaauccguuuugaaguuggaagaagccaucuuguuucugcagcac | NODE\_682202\_length\_10305\_cov\_29.894032:7430..7516:+ |
| NODE\_956217\_length\_18488\_cov\_28.132681\_48185 | 0.1 | 0.62 ± 0.03 |  | 86 | 75 | 11 | 0 | yes |  |  |  | blast | uacagugacugaucaggcaguac | ccugaaaccugaaggcugaga | uacagugacugaucaggcaguacaguuucaggaaccguuuaacugaggcaggauggcaaccugaaaccugaaaccugaaggcugaga | NODE\_956217\_length\_18488\_cov\_28.132681:11482..11569:- |
| NODE\_382880\_length\_18376\_cov\_32.528896\_25612 | 0 | 0.62 ± 0.03 |  | 837 | 831 | 5 | 1 | yes |  |  |  | blast | agcaccacugaugaacaauggg | uuuucagugggagaaag | uuuucagugggagaaaguuuucgucacucuuccucucaugacugaaaccagcaccacugaugaacaauggg | NODE\_382880\_length\_18376\_cov\_32.528896:11256..11327:- |
| NODE\_453588\_length\_30907\_cov\_25.778885\_29324 | 0 | 0.62 ± 0.03 |  | 66 | 66 | 0 | 0 | yes |  |  |  | blast | uuucuuaauucugucucacucu | ggugugaaagaguuauggaugga | ggugugaaagaguuauggauggacagauggagaugucauuuucucgaguuuuucuuaauucugucucacucu | NODE\_453588\_length\_30907\_cov\_25.778885:1952..2024:- |
| NODE\_478719\_length\_58328\_cov\_30.010321\_30780 | 0 | 0.62 ± 0.03 |  | 2327 | 2316 | 11 | 0 | yes |  |  |  | blast | ucaagaguugguaguucaucu | augacaagcucauucuuggcagc | augacaagcucauucuuggcagcuaugcuucaaagaguaacaauaacauuaugggcgaucguggcucaagaguugguaguucaucu | NODE\_478719\_length\_58328\_cov\_30.010321:53044..53130:- |

  
  
  

## mature miRBase miRNAs detected by miRDeep2

  


tag idthis is a tag id assigned by miRDeep2. The first part of the id designates the chromosome or genome contig on which the miRNA gene is located. The second part is a running number that is added to avoid identical ids. The running number is incremented by one for each potential miRNA precursor that is excised from the genome. Clicking this field will display a pdf of the structure, read signature and score breakdown of the miRNA. | miRDeep2 scorethe log-odds score assigned to the hairpin by miRDeep2 | estimated probability that the miRNA is a true positivethe estimated probability that a predicted miRNA with a score of this or higher is a true positive. To see exactly how this probability is estimated, mouse over the 'novel miRNAs, true positives' in the table at the top of the webpage. For miRBase miRNAs, this reflects the support that the data at hand lends to the miRNA. | rfam alertthis field indicates if the miRNA hairpin has sequence similarity to reference rRNAs or tRNAs. Warnings in this field should overrule the estimated probability that a reported miRNA is a true positive (previous field). | predicted mature seq. in accordance with miRBase mature seq.If the predicted miRDeep2 sequence overlaps with the miRBase annotated mature sequence than this is indicated by 'TRUE'. If the predicted miRDeep2 star sequence overlaps with the miRBase annotated mature sequence this is inidicated by 'STAR'. | total read countthis is the sum of read counts for the mature, loop and star miRNAs. | mature read countthis is the number of reads that map to the miRNA hairpin and are contained in the sequence covered by the consensus mature miRNA, including 2 nts upstream and 5 nts downstream. | loop read countthis is the number of reads that map to the miRNA hairpin and are contained in the sequence covered by the consensus miRNA loop, including 2 nts upstream and 5 nts downstream. | star read countthis is the number of reads that map to the miRNA hairpin and are contained in the sequence covered by the consensus star miRNA, including 2 nts upstream and 5 nts downstream. | significant randfold p-valuethis field indicates if the estimated randfold p-value of the miRNA hairpin is equal to or lower than 0.05 (see Bonnet et al., Bioinformatics, 2004). | mature miRBase miRNAthis field displays the ids of any reference mature miRNAs for the species that map perfectly (full length, no mismatches) to the reported miRNA hairpin. If this is the case, the reported miRNA hairpin is assigned as a known miRNA. If not, it is assigned as a novel miRNA. If more than one reference mature miRNA maps to the miRNA hairpin, then only the id of the reference miRBase miRNA that matches the predicted mature sequence is output. | example miRBase miRNA with the same seedthis field displays the ids of any reference mature miRNAs from related species that have a seed sequence identical to that of the reported mature miRNA. The seed is here defined as nucleotides 2-8 from the 5' end of the mature miRNA. If more than one reference mature miRNA have identical seed, then only the id of the miRNA that occurs last in the input file of reference mature miRNAs from related species is displayed. | UCSC browserif a species name was input to miRDeep2, then clicking this field will initiate a UCSC blat search of the consensus precursor sequence against the reference genome. | NCBI blastnclicking this field will initiate a NCBI blastn search of the consensus precursor sequence against the nr/nt database (non-redundant collection of all NCBI nucleotide sequences). | consensus mature sequencethis is the consensus mature miRNA sequence as inferred from the deep sequencing reads. | consensus star sequencethis is the consensus star miRNA sequence as inferred from the deep sequencing reads. | consensus precursor sequencethis is the consensus precursor miRNA sequence as inferred from the deep sequencing reads. Note that this is the inferred Drosha hairpin product, and therefore does not include substantial flanking genomic sequence as does most miRBase precursors. | precursor coordinateThe given precursor coordinates refer do absolute position in the mapped reference sequence || NODE\_323800\_length\_42250\_cov\_31.654675\_22154 | 1.6e+7 | 0.74 ± 0.03 |  | STAR | 32134609 | 32108421 | 570 | 25618 | yes | dre-miR-26a-3p | abu-miR-26b |  | blast | uucaaguaauccaggauaggcu | ccuauucgggaugacuugguuc | uucaaguaauccaggauaggcuuguuaaaguggggaaagccuauucgggaugacuugguuc | NODE\_323800\_length\_42250\_cov\_31.654675:4361..4422:+ |
| NODE\_769019\_length\_3524\_cov\_19.447502\_44330 | 7.9e+6 | 0.74 ± 0.03 |  | STAR | 15633534 | 13151306 | 4 | 2482224 | yes | Aca-Mir-199-P1-v2\_3p | ccr-miR-199-5p |  | blast | cccaguguucagacuaccuguuc | acaguagucugcacauugguu | cccaguguucagacuaccuguucaggaucuuacugguguacaguagucugcacauugguu | NODE\_769019\_length\_3524\_cov\_19.447502:2586..2646:+ |
| NODE\_817731\_length\_59985\_cov\_31.449245\_45824 | 7.6e+6 | 0.74 ± 0.03 |  | STAR | 14982332 | 14981272 | 20 | 1040 | yes | ssa-let-7a-3p | abu-let-7d |  | blast | ugagguaguagguuguauaguu | cuguacagccuccuagcuuucc | ugagguaguagguuguauaguucagagugacaccacaggagauaacuguacagccuccuagcuuucc | NODE\_817731\_length\_59985\_cov\_31.449245:52349..52416:+ |
| NODE\_50037\_length\_7124\_cov\_30.293514\_4340 | 7.5e+6 | 0.74 ± 0.03 |  | STAR | 14885618 | 14643782 | 125 | 241711 | yes | abu-miR-126-5p | abu-miR-126-3p |  | blast | ucguaccgugaguaauaaugca | cauuauuacuuuugguacgcg | cauuauuacuuuugguacgcgcuaugccacucucaacucguaccgugaguaauaaugca | NODE\_50037\_length\_7124\_cov\_30.293514:4929..4988:- |
| NODE\_33036\_length\_9596\_cov\_29.807629\_2940 | 4.3e+6 | 0.74 ± 0.03 |  | STAR | 8568585 | 8566338 | 0 | 2247 | yes | ola-miR-125a-3p | gmo-miR-125a-5p |  | blast | ucccugagacccuuaaccugug | acaggugagguccucgggaac | ucccugagacccuuaaccugugaggucaaagcaggucacaggugagguccucgggaac | NODE\_33036\_length\_9596\_cov\_29.807629:2800..2858:+ |
| NODE\_347176\_length\_38458\_cov\_26.791616\_23459 | 3.4e+6 | 0.74 ± 0.03 |  | STAR | 6798186 | 6779273 | 23 | 18890 | yes | ssa-miR-21b-3p | ssa-miR-21a-5p |  | blast | uagcuuaucagacugguguuggc | cgacaacagucuguaggcugu | uagcuuaucagacugguguuggcuguuaagauugcaaggcgacaacagucuguaggcugu | NODE\_347176\_length\_38458\_cov\_26.791616:36121..36181:- |
| NODE\_436535\_length\_5246\_cov\_24.373238\_28358 | 3.1e+6 | 0.74 ± 0.03 |  | STAR | 6238511 | 6238128 | 203 | 180 | yes | ssa-miR-1-5p | abu-miR-206 |  | blast | uggaauguaaagaaguauguau | acauacuucuuuauguacccau | acauacuucuuuauguacccauaugaacauaugauagcuauggaauguaaagaaguauguau | NODE\_436535\_length\_5246\_cov\_24.373238:4825..4887:- |
| NODE\_88815\_length\_2463\_cov\_24.178238\_7241 | 2.7e+6 | 0.74 ± 0.03 |  | TRUE | 5353968 | 5352934 | 30 | 1004 | yes | abu-miR-1 | abu-miR-206 |  | blast | uggaauguaaagaaguauguau | acauacuucuuuauaugcccaua | acauacuucuuuauaugcccauaugaacaagagcaacuauggaauguaaagaaguauguau | NODE\_88815\_length\_2463\_cov\_24.178238:682..743:+ |
| NODE\_506320\_length\_10016\_cov\_21.301718\_32498 | 2.3e+6 | 0.74 ± 0.03 |  | STAR | 4653459 | 4535004 | 0 | 118455 | yes | ssa-miR-143-5p | abu-miR-143 |  | blast | ugagaugaagcacuguagcucg | ggugcagugcugcaucucugguc | ggugcagugcugcaucucuggucaguugguagucugagaugaagcacuguagcucg | NODE\_506320\_length\_10016\_cov\_21.301718:5294..5350:- |
| NODE\_66567\_length\_3308\_cov\_24.986094\_5487 | 2.2e+6 | 0.74 ± 0.03 |  | TRUE | 4416768 | 3165953 | 0 | 1250815 | yes | Cte-Mir-9\_5p | dre-miR-9-5p |  | blast | ucuuugguuaucuagcuguau | uaaagcuagauaaccgaaagu | ucuuugguuaucuagcuguaugaguguucugcucgucauaaagcuagauaaccgaaagu | NODE\_66567\_length\_3308\_cov\_24.986094:15..74:- |
| NODE\_244332\_length\_6934\_cov\_20.491491\_17541 | 2.2e+6 | 0.74 ± 0.03 |  | TRUE | 4415882 | 3165953 | 12 | 1249917 | yes | Cte-Mir-9\_5p | dre-miR-9-5p |  | blast | ucuuugguuaucuagcuguau | uaaagcuagauaaccgaaagu | ucuuugguuaucuagcuguaugagugacguacauucuucauaaagcuagauaaccgaaagu | NODE\_244332\_length\_6934\_cov\_20.491491:5388..5449:- |
| NODE\_291214\_length\_21711\_cov\_26.518124\_20297 | 2.2e+6 | 0.74 ± 0.03 |  | TRUE | 4415873 | 3165953 | 3 | 1249917 | yes | Cte-Mir-9\_5p | dre-miR-9-5p |  | blast | ucuuugguuaucuagcuguau | uaaagcuagauaaccgaaagu | ucuuugguuaucuagcuguaugagugauguacauucuucauaaagcuagauaaccgaaagu | NODE\_291214\_length\_21711\_cov\_26.518124:12400..12461:- |
| NODE\_261965\_length\_3956\_cov\_25.583164\_18679 | 2.2e+6 | 0.74 ± 0.03 |  | TRUE | 4378871 | 3165442 | 0 | 1213429 | yes | Cte-Mir-9\_5p | dre-miR-9-5p |  | blast | ucuuugguuaucuagcuguau | uaaagcuagauaaccgaaagu | ucuuugguuaucuagcuguaugaguguuaaauaccugucauaaagcuagauaaccgaaagu | NODE\_261965\_length\_3956\_cov\_25.583164:2111..2172:- |
| NODE\_158693\_length\_38410\_cov\_31.448738\_12073 | 2.2e+6 | 0.74 ± 0.03 |  | TRUE | 4378796 | 3165442 | 0 | 1213354 | yes | Cte-Mir-9\_5p | dre-miR-9-5p |  | blast | ucuuugguuaucuagcuguau | uaaagcuagauaaccgaaagu | ucuuugguuaucuagcuguaugagugauaaucaugcaucauaaagcuagauaaccgaaagu | NODE\_158693\_length\_38410\_cov\_31.448738:18898..18959:- |
| NODE\_654140\_length\_10930\_cov\_25.100000\_39928 | 2.1e+6 | 0.74 ± 0.03 |  | TRUE | 4259572 | 4031030 | 0 | 228542 | yes | Aca-Mir-181-P1a\_5p | dre-miR-181a-5p |  | blast | aacauucaacgcugucggugagu | accaucgaccguugacuguacc | aacauucaacgcugucggugaguuugagcucugacugaaaaccaucgaccguugacuguacc | NODE\_654140\_length\_10930\_cov\_25.100000:8637..8699:+ |
| NODE\_628735\_length\_9290\_cov\_22.721205\_38714 | 2.1e+6 | 0.74 ± 0.03 |  | TRUE | 4212968 | 4031083 | 0 | 181885 | yes | Aca-Mir-181-P1a\_5p | dre-miR-181a-5p |  | blast | aacauucaacgcugucggugagu | accaucgaccguugacugugccc | aacauucaacgcugucggugaguuugguauggacacaaaaaaccaucgaccguugacugugccc | NODE\_628735\_length\_9290\_cov\_22.721205:2135..2199:+ |
| NODE\_628735\_length\_9290\_cov\_22.721205\_38712 | 2.1e+6 | 0.74 ± 0.03 |  | TRUE | 4212968 | 4031083 | 0 | 181885 | no | Aca-Mir-181-P1a\_5p | dre-miR-181a-5p |  | blast | aacauucaacgcugucggugagu | accaucgaccguugacugugccc | aacauucaacgcugucggugaguuugguauggacacaaaaaaccaucgaccguugacugugccc | NODE\_628735\_length\_9290\_cov\_22.721205:1952..2016:+ |
| NODE\_213164\_length\_25703\_cov\_27.894566\_15727 | 2.1e+6 | 0.74 ± 0.03 |  | STAR | 4188676 | 4186987 | 0 | 1689 | yes | dre-miR-10b-3p | ccr-miR-10c |  | blast | uacccuguagaaccgaauuugu | acagauucgauucuaggggagu | uacccuguagaaccgaauuugugugaugaaaccagauucacagauucgauucuaggggagu | NODE\_213164\_length\_25703\_cov\_27.894566:4878..4939:- |
| NODE\_33541\_length\_42464\_cov\_30.625048\_3026 | 2.1e+6 | 0.74 ± 0.03 |  | TRUE | 4162539 | 4031091 | 38 | 131410 | yes | Aca-Mir-181-P1a\_5p | dre-miR-181a-5p |  | blast | aacauucaacgcugucggugagu | accaucgaccguugacuguacc | aacauucaacgcugucggugaguuugagcuaaauugaaagaaaaaaaaccaucgaccguugacuguacc | NODE\_33541\_length\_42464\_cov\_30.625048:4106..4175:+ |
| NODE\_436535\_length\_5246\_cov\_24.373238\_28356 | 2.1e+6 | 0.74 ± 0.03 |  | STAR | 4141762 | 4093994 | 40 | 47728 | yes | ola-miR-133-5p | abu-miR-133a |  | blast | uugguccccuucaaccagcugu | agcugguaaaauggaaccaaau | agcugguaaaauggaaccaaaucaccucuugaauggauuugguccccuucaaccagcugu | NODE\_436535\_length\_5246\_cov\_24.373238:1149..1209:- |
| NODE\_382886\_length\_10328\_cov\_29.012491\_25614 | 2.1e+6 | 0.74 ± 0.03 |  | STAR | 4141590 | 4093994 | 175 | 47421 | yes | ola-miR-133-5p | abu-miR-133a |  | blast | uugguccccuucaaccagcugu | agcugguaaaauggaaccaaau | agcugguaaaauggaaccaaaucaacuguucaauggauuugguccccuucaaccagcugu | NODE\_382886\_length\_10328\_cov\_29.012491:3992..4052:- |
| NODE\_775422\_length\_5997\_cov\_30.301151\_44536 | 2.0e+6 | 0.74 ± 0.03 |  | TRUE | 4035791 | 4031286 | 2 | 4503 | yes | Aca-Mir-181-P1a\_5p | dre-miR-181a-5p |  | blast | aacauucaacgcugucggugagu | accaucgaguguugaguguacc | aacauucaacgcugucggugaguuugugacucagagaaaaccaucgaguguugaguguacc | NODE\_775422\_length\_5997\_cov\_30.301151:989..1050:+ |
| NODE\_806611\_length\_57927\_cov\_26.436584\_45616 | 1.7e+6 | 0.74 ± 0.03 |  | STAR | 3469859 | 3463170 | 1 | 6688 | no | ssa-miR-128-1-5p | dre-miR-128-3p |  | blast | ucacagugaaccggucucuuu | cggggccgggacgcugucugaga | cggggccgggacgcugucugagaggccucuaugaaucucacagugaaccggucucuuu | NODE\_806611\_length\_57927\_cov\_26.436584:23573..23631:- |
| NODE\_165549\_length\_25301\_cov\_26.792656\_12473 | 1.7e+6 | 0.74 ± 0.03 |  | TRUE | 3466743 | 3463146 | 100 | 3497 | yes | dre-miR-128-3p | dre-miR-128-3p |  | blast | ucacagugaaccggucucuuu | gggggccguuacacugucaga | gggggccguuacacugucagagauguagucugagggucucacagugaaccggucucuuu | NODE\_165549\_length\_25301\_cov\_26.792656:23109..23168:+ |
| NODE\_18233\_length\_5886\_cov\_25.828577\_1820 | 1.6e+6 | 0.74 ± 0.03 |  | TRUE | 3292816 | 3279601 | 3 | 13212 | yes | Cli-Let-7-P12\_5p | abu-let-7d |  | blast | ugagguaguaguuugugcuguu | cugcgcaagcuacugccuugcu | ugagguaguaguuugugcuguugguuggguugagacacugcccgcuauggagaugacugcgcaagcuacugccuugcu | NODE\_18233\_length\_5886\_cov\_25.828577:601..679:+ |
| NODE\_261965\_length\_3956\_cov\_25.583164\_18680 | 1.6e+6 | 0.74 ± 0.03 |  | TRUE | 3165446 | 3165442 | 3 | 1 | no | Cte-Mir-9\_5p | dre-miR-9-5p |  | blast | ucuuugguuaucuagcuguau | ugucgggcuacagcauca | ugucgggcuacagcaucagagagugugagagagggggucuguuuuugucuuugguuaucuagcuguau | NODE\_261965\_length\_3956\_cov\_25.583164:2151..2219:- |
| NODE\_330003\_length\_22733\_cov\_28.230062\_22570 | 1.4e+6 | 0.74 ± 0.03 |  | STAR | 2862722 | 2850319 | 20 | 12383 | yes | dre-miR-27b-5p | abu-miR-27c |  | blast | uucacaguggcuaaguucugc | agagcuuagcugauuggugaac | agagcuuagcugauuggugaacagugauugauucccucuuuguucacaguggcuaaguucugc | NODE\_330003\_length\_22733\_cov\_28.230062:11877..11940:- |
| NODE\_845088\_length\_51147\_cov\_29.451210\_46604 | 1.4e+6 | 0.74 ± 0.03 |  | TRUE | 2824671 | 2732961 | 27 | 91683 | yes | abu-miR-22a | abu-miR-22a |  | blast | aagcugccagcugaagaacugu | aguucuucacuggcaagcuuu | aguucuucacuggcaagcuuuauguccucauguaccagcuaaagcugccagcugaagaacugu | NODE\_845088\_length\_51147\_cov\_29.451210:36942..37005:- |
| NODE\_717828\_length\_18120\_cov\_32.841335\_42718 | 1.2e+6 | 0.74 ± 0.03 |  | TRUE | 2486818 | 2483518 | 0 | 3300 | yes | abu-let-7e | abu-let-7d |  | blast | ugagguaguagauugaauaguu | cuauacaaucuacugucuuucc | ugagguaguagauugaauaguugugggguuuacguccuccuuuugagauaacuauacaaucuacugucuuucc | NODE\_717828\_length\_18120\_cov\_32.841335:13280..13353:+ |
| NODE\_14422\_length\_15369\_cov\_26.303469\_1428 | 1.2e+6 | 0.74 ± 0.03 |  | TRUE | 2431940 | 2429022 | 0 | 2918 | yes | abu-let-7e | abu-let-7d |  | blast | ugagguaguagauugaauaguu | cuauacaaucuacugucuuucc | ugagguaguagauugaauaguugugggguuguguaaccucuuuuuuaaauaacuauacaaucuacugucuuucc | NODE\_14422\_length\_15369\_cov\_26.303469:11242..11316:+ |
| NODE\_808688\_length\_4716\_cov\_24.903095\_45620 | 1.1e+6 | 0.74 ± 0.03 |  | TRUE | 2338350 | 1973167 | 82 | 365101 | yes | abu-miR-30a-5p | abu-miR-30a-5p |  | blast | uguaaacauccuugacuggaagcu | cuuucagucggauguuugcagc | uguaaacauccuugacuggaagcugguguuaguuucuggggcuuucagucggauguuugcagc | NODE\_808688\_length\_4716\_cov\_24.903095:3724..3787:+ |
| NODE\_34239\_length\_42430\_cov\_28.169903\_3082 | 1.1e+6 | 0.74 ± 0.03 |  | TRUE | 2336600 | 1973178 | 52 | 363370 | yes | abu-miR-30a-5p | abu-miR-30a-5p |  | blast | uguaaacauccuugacuggaagcu | cuuucagucggauguuugcagc | uguaaacauccuugacuggaagcugagguuuugacagcgagggcuuucagucggauguuugcagc | NODE\_34239\_length\_42430\_cov\_28.169903:37421..37486:+ |
| NODE\_818714\_length\_30787\_cov\_27.310358\_45845 | 1.0e+6 | 0.74 ± 0.03 |  | TRUE | 2102020 | 2100347 | 0 | 1673 | yes | abu-miR-204 | abu-miR-204 |  | blast | uucccuuugucauccuaugccu | gcagggacagcaaaggggagc | uucccuuugucauccuaugccuggagcugucuguaaggcagggacagcaaaggggagc | NODE\_818714\_length\_30787\_cov\_27.310358:17396..17454:- |
| NODE\_479011\_length\_27543\_cov\_30.564100\_30793 | 9.8e+5 | 0.74 ± 0.03 |  | TRUE | 1940468 | 1939447 | 0 | 1021 | yes | abu-miR-204 | abu-miR-204 |  | blast | uucccuuugucauccuaugccu | gcagggacagcaaagggaugc | uucccuuugucauccuaugccuggaacuuggauaaggcagggacagcaaagggaugc | NODE\_479011\_length\_27543\_cov\_30.564100:9327..9384:- |
| NODE\_660656\_length\_9710\_cov\_27.013800\_40291 | 9.8e+5 | 0.74 ± 0.03 |  | TRUE | 1939655 | 1939480 | 0 | 175 | yes | abu-miR-204 | abu-miR-204 |  | blast | uucccuuugucauccuaugccu | gcugggaaggcaaagggacgg | uucccuuugucauccuaugccuggauuaauacaaaaggggcugggaaggcaaagggacgg | NODE\_660656\_length\_9710\_cov\_27.013800:451..511:- |
| NODE\_412784\_length\_6493\_cov\_22.596334\_27128 | 9.8e+5 | 0.74 ± 0.03 |  | TRUE | 1929429 | 1913697 | 1 | 15731 | yes | abu-miR-27c | abu-miR-27c |  | blast | uucacagugguuaaguucugc | caggacuuaacccacaugugaaca | caggacuuaacccacaugugaacagugagugucugccauguucacagugguuaaguucugc | NODE\_412784\_length\_6493\_cov\_22.596334:2208..2269:- |
| NODE\_626612\_length\_3706\_cov\_22.327307\_38552 | 8.3e+5 | 0.74 ± 0.03 |  | TRUE | 1639119 | 1479109 | 0 | 160010 | yes | abu-miR-222 | abu-miR-221 |  | blast | agcuacaucuggcuacugggucu | ugcucaguagucaguguagauc | ugcucaguagucaguguagauccuguggggcuggcagcagcuacaucuggcuacugggucu | NODE\_626612\_length\_3706\_cov\_22.327307:929..990:- |
| NODE\_729766\_length\_55210\_cov\_28.171492\_43108 | 8.1e+5 | 0.74 ± 0.03 |  | STAR | 1606267 | 1479175 | 0 | 127092 | yes | ssa-miR-222a-5p | abu-miR-221 |  | blast | agcuacaucuggcuacugggucu | ugcucaguaggcaguguagauc | ugcucaguaggcaguguagauccuguguagcaaucagcagcuacaucuggcuacugggucu | NODE\_729766\_length\_55210\_cov\_28.171492:31594..31655:- |
| NODE\_322962\_length\_59330\_cov\_30.457020\_22129 | 7.7e+5 | 0.74 ± 0.03 |  | TRUE | 1522719 | 1522290 | 22 | 407 | yes | abu-miR-206 | abu-miR-206 |  | blast | uggaauguaaggaagugugugg | acaugcuuccuuauauccccaua | acaugcuuccuuauauccccauauuaauacgccacuuauggaauguaaggaagugugugg | NODE\_322962\_length\_59330\_cov\_30.457020:36053..36113:+ |
| NODE\_91705\_length\_15181\_cov\_29.275410\_7476 | 7.5e+5 | 0.74 ± 0.03 |  | STAR | 1488238 | 1395038 | 8 | 93192 | yes | Xtr-Mir-132-P2\_3p | dre-miR-212-5p |  | blast | accuuggcuuuagacugcuuacu | uaacagucuacagucauggcu | accuuggcuuuagacugcuuacugcuuauacaguaucaaaguacaguaacagucuacagucauggcu | NODE\_91705\_length\_15181\_cov\_29.275410:2623..2690:+ |
| NODE\_729766\_length\_55210\_cov\_28.171492\_43106 | 6.7e+5 | 0.74 ± 0.03 |  | STAR | 1315119 | 1152403 | 0 | 162716 | yes | dre-miR-221-5p | abu-miR-221 |  | blast | agcuacauugucugcuggguuu | accuggcauacaauguagauuu | accuggcauacaauguagauuucugugugguucaauucuacagcuacauugucugcuggguuu | NODE\_729766\_length\_55210\_cov\_28.171492:31130..31193:- |
| NODE\_34239\_length\_42430\_cov\_28.169903\_3084 | 6.4e+5 | 0.74 ± 0.03 |  | TRUE | 1273218 | 1271745 | 1 | 1472 | yes | Aca-Mir-30-P2a\_5p | abu-miR-30a-5p |  | blast | uguaaacauccuacacucucagcu | cugggagagggguguuuacgcu | uguaaacauccuacacucucagcuguguugucauggagcugggagagggguguuuacgcu | NODE\_34239\_length\_42430\_cov\_28.169903:37756..37816:+ |
| NODE\_143105\_length\_7717\_cov\_25.466503\_10983 | 6.2e+5 | 0.74 ± 0.03 |  | TRUE | 1221505 | 1181208 | 136 | 40161 | yes | Cli-Let-7-P8\_5p | abu-let-7d |  | blast | ugagguaguagguugugugguu | cuauacaaccuacugccuuccc | ugagguaguagguugugugguuucaggguugugauuuuaccccaucaggagcuaacuauacaaccuacugccuuccc | NODE\_143105\_length\_7717\_cov\_25.466503:2997..3074:- |
| NODE\_630262\_length\_18241\_cov\_27.215340\_38942 | 6.0e+5 | 0.74 ± 0.03 |  | TRUE | 1181209 | 1181208 | 0 | 1 | yes | Cli-Let-7-P8\_5p | abu-let-7d |  | blast | ugagguaguagguugugugguu | uacccagccuguagggcc | uacccagccuguagggccuuuacugauguucguacggggugagguaguagguugugugguu | NODE\_630262\_length\_18241\_cov\_27.215340:9167..9228:- |
| NODE\_128862\_length\_18763\_cov\_25.828119\_10008 | 5.7e+5 | 0.74 ± 0.03 |  | TRUE | 1124328 | 1123799 | 153 | 376 | yes | abu-miR-462 | abu-miR-462 |  | blast | uaacggaacccauaaugcagcug | gcugguuaugggguccguuucc | uaacggaacccauaaugcagcugugauucugagccccagcugguuaugggguccguuucc | NODE\_128862\_length\_18763\_cov\_25.828119:9183..9243:+ |
| NODE\_626612\_length\_3706\_cov\_22.327307\_38550 | 5.4e+5 | 0.74 ± 0.03 |  | TRUE | 1075763 | 1072886 | 0 | 2877 | yes | abu-miR-221 | abu-miR-221 |  | blast | agcuacauugucugcuggguuu | accuggcauacaguguaggaaucugu | accuggcauacaguguaggaaucugugugugucagucuacagcuacauugucugcuggguuu | NODE\_626612\_length\_3706\_cov\_22.327307:539..601:- |
| NODE\_671686\_length\_27699\_cov\_27.903570\_40888 | 5.3e+5 | 0.74 ± 0.03 |  | STAR | 1048966 | 1037688 | 0 | 11278 | yes | ssa-miR-7a-6-3p | ccr-miR-7a |  | blast | uggaagacuagugauuuuguugu | caacaagucccagucugccucu | uggaagacuagugauuuuguugugacgcugaucaaagaacaacaagucccagucugccucu | NODE\_671686\_length\_27699\_cov\_27.903570:17799..17860:+ |
| NODE\_246211\_length\_22333\_cov\_31.187391\_17764 | 5.2e+5 | 0.74 ± 0.03 |  | TRUE | 1036636 | 1013299 | 1128 | 22209 | yes | abu-miR-200a | abu-miR-200a |  | blast | uaacacugucugguaacgaugu | caucuuaccugacagugcugga | caucuuaccugacagugcuggacuguacuacuguuguucuaacacugucugguaacgaugu | NODE\_246211\_length\_22333\_cov\_31.187391:16168..16229:- |
| NODE\_506320\_length\_10016\_cov\_21.301718\_32495 | 5.0e+5 | 0.74 ± 0.03 |  | TRUE | 988881 | 488142 | 4 | 500735 | yes | Aca-Mir-145\_5p | gmo-miR-145-5p |  | blast | guccaguuuucccaggaaucccu | ggauuccuggaaauacuguucu | guccaguuuucccaggaaucccuugaccuaucagaaagggggauuccuggaaauacuguucu | NODE\_506320\_length\_10016\_cov\_21.301718:4958..5020:- |
| NODE\_246211\_length\_22333\_cov\_31.187391\_17766 | 4.3e+5 | 0.74 ± 0.03 |  | STAR | 851531 | 847608 | 0 | 3923 | yes | dre-miR-200b-5p | abu-miR-429b |  | blast | uaauacugccugguaaugaugau | caucuuacgaggcagcauugga | caucuuacgaggcagcauuggauaucaucacuuucucuaauacugccugguaaugaugau | NODE\_246211\_length\_22333\_cov\_31.187391:16355..16415:- |
| NODE\_450908\_length\_48195\_cov\_31.381699\_29138 | 3.8e+5 | 0.74 ± 0.03 |  | TRUE | 751799 | 658602 | 5 | 93192 | yes | Aca-Mir-132-P2\_5p | dre-miR-212-5p |  | blast | accuuggcucuagacugcuuacu | uaacagucuacagucauggcu | accuuggcucuagacugcuuacugcuaaaacuccuccaaaguacaguaacagucuacagucauggcu | NODE\_450908\_length\_48195\_cov\_31.381699:12478..12545:+ |
| NODE\_629377\_length\_60300\_cov\_32.229584\_38792 | 2.9e+5 | 0.74 ± 0.03 |  | TRUE | 584725 | 583019 | 9 | 1697 | yes | Bta-Mir-205-P1\_5p | ipu-miR-205 |  | blast | uccuucauuccaccggagucu | uuucaguggugugaaguguaaga | uccuucauuccaccggagucuguaucuguauuugaccagauuucaguggugugaaguguaaga | NODE\_629377\_length\_60300\_cov\_32.229584:22604..22667:+ |
| NODE\_78715\_length\_30245\_cov\_32.456009\_6413 | 2.9e+5 | 0.74 ± 0.03 |  | TRUE | 583011 | 582245 | 2 | 764 | yes | Bta-Mir-205-P1\_5p | ipu-miR-205 |  | blast | uccuucauuccaccggagucu | gauuucagugguuugaagagua | uccuucauuccaccggagucuguguaaagugcuaaucagauuucagugguuugaagagua | NODE\_78715\_length\_30245\_cov\_32.456009:29770..29830:- |
| NODE\_322962\_length\_59330\_cov\_30.457020\_22131 | 2.7e+5 | 0.74 ± 0.03 |  | STAR | 543782 | 543520 | 81 | 181 | yes | ssa-miR-133b-5p | dre-miR-133c-3p |  | blast | uuugguccccuucaaccagcu | gcuggucaaacggaaccaaguc | gcuggucaaacggaaccaagucagguguuucugugagguuugguccccuucaaccagcu | NODE\_322962\_length\_59330\_cov\_30.457020:36915..36974:+ |
| NODE\_156184\_length\_43736\_cov\_27.921209\_11780 | 2.6e+5 | 0.74 ± 0.03 |  | TRUE | 522048 | 447297 | 35 | 74716 | yes | abu-miR-2188 | abu-miR-2188 |  | blast | aagguccaaccucacauguccu | gcugugugaggucagaccuauc | aagguccaaccucacauguccugugcggcugaaggaaggcugugugaggucagaccuauc | NODE\_156184\_length\_43736\_cov\_27.921209:2429..2489:+ |
| NODE\_126256\_length\_9493\_cov\_22.245338\_9878 | 2.6e+5 | 0.74 ± 0.03 |  | STAR | 516553 | 492795 | 2 | 23756 | yes | Bta-Mir-153-P2\_5p | abu-miR-153a |  | blast | uugcauagucacaaaagugauc | ucauuuuugugauguugcagcu | ucauuuuugugauguugcagcuaguaauaugagcccaguugcauagucacaaaagugauc | NODE\_126256\_length\_9493\_cov\_22.245338:6905..6965:- |
| NODE\_525082\_length\_27855\_cov\_28.354012\_33533 | 2.6e+5 | 0.74 ± 0.03 |  | STAR | 516551 | 492795 | 0 | 23756 | yes | Bta-Mir-153-P2\_5p | abu-miR-153a |  | blast | uugcauagucacaaaagugauc | ucauuuuugugauguugcagcu | ucauuuuugugauguugcagcuaguaauauaagcccaguugcauagucacaaaagugauc | NODE\_525082\_length\_27855\_cov\_28.354012:21880..21940:+ |
| NODE\_876410\_length\_25327\_cov\_33.273937\_47190 | 2.4e+5 | 0.74 ± 0.03 |  | TRUE | 473565 | 472275 | 1 | 1289 | yes | abu-miR-724 | abu-miR-724 |  | blast | uuaaagggaauuugcgacuguu | cagccacaccuuccuuuuaaga | uuaaagggaauuugcgacuguuaaucaaaccauucgaacagccacaccuuccuuuuaaga | NODE\_876410\_length\_25327\_cov\_33.273937:18674..18734:+ |
| NODE\_619512\_length\_85982\_cov\_31.709206\_38196 | 2.4e+5 | 0.74 ± 0.03 |  | TRUE | 473397 | 472106 | 2 | 1289 | yes | abu-miR-724 | abu-miR-724 |  | blast | uuaaagggaauuugcgacuguu | cagccacaccuuccuuuuaaga | uuaaagggaauuugcgacuguuagucaaaacauuagaacagccacaccuuccuuuuaaga | NODE\_619512\_length\_85982\_cov\_31.709206:57980..58040:+ |
| NODE\_760618\_length\_35970\_cov\_25.822741\_44022 | 2.2e+5 | 0.74 ± 0.03 |  | TRUE | 442615 | 216450 | 0 | 226165 | yes | pny-miR-144 |  |  | blast | ggauaucaucuuauacuguaagu | cuacaguauagaugauguac | ggauaucaucuuauacuguaaguuuaaaaaagagacacuacaguauagaugauguac | NODE\_760618\_length\_35970\_cov\_25.822741:2974..3031:+ |
| NODE\_843582\_length\_56377\_cov\_27.843359\_46546 | 2.1e+5 | 0.74 ± 0.03 |  | STAR | 417716 | 406573 | 0 | 11143 | yes | fru-miR-24-5p | ipu-miR-24 |  | blast | uggcucaguucagcaggaac | ugccuacugaacugguaucagu | ugccuacugaacugguaucaguguuauaccugaaaacuggcucaguucagcaggaac | NODE\_843582\_length\_56377\_cov\_27.843359:10509..10566:- |
| NODE\_330003\_length\_22733\_cov\_28.230062\_22568 | 2.0e+5 | 0.74 ± 0.03 |  | TRUE | 411490 | 406590 | 7 | 4893 | yes | ipu-miR-24 | ipu-miR-24 |  | blast | uggcucaguucagcaggaac | ugccuacugagcugauaauc | ugccuacugagcugauaaucaguccuacagaaucacuggcucaguucagcaggaac | NODE\_330003\_length\_22733\_cov\_28.230062:8413..8469:- |
| NODE\_412784\_length\_6493\_cov\_22.596334\_27126 | 2.0e+5 | 0.74 ± 0.03 |  | STAR | 409514 | 406251 | 13 | 3250 | yes | ssa-miR-24a-4-5p | ipu-miR-24 |  | blast | uggcucaguucagcaggaac | ugccugcugugcugauaauc | ugccugcugugcugauaaucagugugugacgucggcuggcucaguucagcaggaac | NODE\_412784\_length\_6493\_cov\_22.596334:783..839:- |
| NODE\_207640\_length\_20790\_cov\_30.320875\_15281 | 1.9e+5 | 0.74 ± 0.03 |  | TRUE | 387004 | 382809 | 0 | 4195 | yes | abu-miR-103 | abu-miR-103 |  | blast | agcagcauuguacagggcuauga | agccucuuuacggugcugccuugu | agccucuuuacggugcugccuugugcugucuugaucaagcagcauuguacagggcuauga | NODE\_207640\_length\_20790\_cov\_30.320875:1816..1876:+ |
| NODE\_540637\_length\_11690\_cov\_27.516083\_34333 | 1.9e+5 | 0.74 ± 0.03 |  | TRUE | 374170 | 284733 | 107 | 89330 | yes | Sha-Mir-129-P1\_5p | dre-miR-722 |  | blast | cuuuuugcggucugggcuugc | aagcccuuaccccaaaaagcau | cuuuuugcggucugggcuugcugugcauacaugaaccugggaagcccuuaccccaaaaagcau | NODE\_540637\_length\_11690\_cov\_27.516083:11060..11123:- |
| NODE\_374989\_length\_4954\_cov\_32.948727\_25176 | 1.8e+5 | 0.74 ± 0.03 |  | TRUE | 366064 | 284721 | 67 | 81276 | yes | Sha-Mir-129-P1\_5p | dre-miR-722 |  | blast | cuuuuugcggucugggcuugc | aagcccuuaccccaaaaagcau | cuuuuugcggucugggcuugcugucaugugucuauccaggaagcccuuaccccaaaaagcau | NODE\_374989\_length\_4954\_cov\_32.948727:2180..2242:+ |
| NODE\_91705\_length\_15181\_cov\_29.275410\_7478 | 1.8e+5 | 0.74 ± 0.03 |  | TRUE | 365326 | 261362 | 4 | 103960 | yes | abu-miR-132-5p | abu-miR-132-5p |  | blast | accguggcuuuagauuguuacu | uaacagucuacagccaugguc | accguggcuuuagauuguuacucuagcaacagcaccaugguaacagucuacagccaugguc | NODE\_91705\_length\_15181\_cov\_29.275410:3024..3085:+ |
| NODE\_64838\_length\_18235\_cov\_35.798466\_5387 | 1.7e+5 | 0.74 ± 0.03 |  | TRUE | 347367 | 283196 | 21 | 64150 | yes | Dre-Mir-142-P1-v2\_5p | dre-miR-142b-5p |  | blast | cauaaaguagaaagcacuacu | uguaguguuuccuacuuuaugg | cauaaaguagaaagcacuacuaaacuuuacuacacaguguaguguuuccuacuuuaugg | NODE\_64838\_length\_18235\_cov\_35.798466:7558..7617:- |
| NODE\_573665\_length\_57135\_cov\_28.812794\_35995 | 1.7e+5 | 0.74 ± 0.03 |  | TRUE | 346419 | 282204 | 65 | 64150 | yes | Dre-Mir-142-P1-v2\_5p | dre-miR-142b-5p |  | blast | cauaaaguagaaagcacuacu | uguaguguuuccuacuuuaugg | cauaaaguagaaagcacuacuaaacuccucgccacaguguaguguuuccuacuuuaugg | NODE\_573665\_length\_57135\_cov\_28.812794:50434..50493:- |
| NODE\_576206\_length\_29714\_cov\_25.878004\_36133 | 1.7e+5 | 0.74 ± 0.03 |  | STAR | 335736 | 303398 | 21 | 32317 | yes | ssa-let-7h-3p | abu-let-7d |  | blast | ugagguaguaaguuguguuguu | cuauacaacuuacugccuuccu | ugagguaguaaguuguguuguuguuggggaucaggauugugcaccccguuaaggagauaacuauacaacuuacugccuuccu | NODE\_576206\_length\_29714\_cov\_25.878004:21904..21986:- |
| NODE\_737847\_length\_83181\_cov\_28.914331\_43397 | 1.6e+5 | 0.74 ± 0.03 |  | STAR | 331111 | 324019 | 0 | 7092 | yes | dre-miR-24b-5p | ipu-miR-24 |  | blast | uggcucaguucagcaggaac | ugccuacugagcugauaacagu | ugccuacugagcugauaacaguuagauguuaacaagcacuggcucaguucagcaggaac | NODE\_737847\_length\_83181\_cov\_28.914331:33078..33137:+ |
| NODE\_128862\_length\_18763\_cov\_25.828119\_10010 | 1.6e+5 | 0.74 ± 0.03 |  | TRUE | 329445 | 317881 | 7 | 11557 | yes | abu-miR-731-5p | abu-miR-731-5p |  | blast | aaugacacguuuucucccggauu | aaccgggaauuucgugucagcc | aaugacacguuuucucccggauugcugugagcaggaaacgcaaccgggaauuucgugucagcc | NODE\_128862\_length\_18763\_cov\_25.828119:9637..9700:+ |
| NODE\_303539\_length\_39784\_cov\_31.875326\_20970 | 1.6e+5 | 0.74 ± 0.03 |  | TRUE | 319232 | 315336 | 0 | 3896 | yes | abu-miR-25 | abu-miR-25 |  | blast | cauugcacuugucucggucuga | aggcggagacuugggcaauugcc | aggcggagacuugggcaauugccgggcaucccagagggcauugcacuugucucggucuga | NODE\_303539\_length\_39784\_cov\_31.875326:22783..22843:- |
| NODE\_682820\_length\_16829\_cov\_28.683998\_41260 | 1.6e+5 | 0.74 ± 0.03 |  | TRUE | 314225 | 311591 | 0 | 2634 | yes | abu-miR-101a | abu-miR-101a |  | blast | uacaguacugugauaacugaag | ucaguuaucacagugcugaugc | ucaguuaucacagugcugaugcuguccccaucgaagguacaguacugugauaacugaag | NODE\_682820\_length\_16829\_cov\_28.683998:11888..11947:- |
| NODE\_369113\_length\_11667\_cov\_26.980629\_24654 | 1.5e+5 | 0.74 ± 0.03 |  | TRUE | 300026 | 284982 | 21 | 15023 | yes | Sha-Mir-129-P1\_5p | dre-miR-722 |  | blast | cuuuuugcggucugggcuugc | aagcccuuaccccaaaaaguau | cuuuuugcggucugggcuugcuguuccuaaggcaguagccaggaagcccuuaccccaaaaaguau | NODE\_369113\_length\_11667\_cov\_26.980629:5407..5472:+ |
| NODE\_533907\_length\_36799\_cov\_27.206146\_33973 | 1.5e+5 | 0.74 ± 0.03 |  | TRUE | 297453 | 297343 | 0 | 110 | yes | abu-miR-15a | abu-miR-15a |  | blast | uagcagcacggaaugguuugu | caggccauacugugcugccgca | uagcagcacggaaugguuuguggguuauacugagaugcaggccauacugugcugccgca | NODE\_533907\_length\_36799\_cov\_27.206146:9344..9403:- |
| NODE\_824524\_length\_44939\_cov\_30.778477\_46015 | 1.4e+5 | 0.74 ± 0.03 |  | STAR | 286312 | 264897 | 9 | 21406 | yes | Ocu-Mir-214\_5p |  |  | blast | acagcaggcacagacaggcaga | ugccugucuacacuugcugugc | ugccugucuacacuugcugugcagaccuucugcuccuguacagcaggcacagacaggcaga | NODE\_824524\_length\_44939\_cov\_30.778477:1051..1112:+ |
| NODE\_679149\_length\_21682\_cov\_30.839037\_41042 | 1.3e+5 | 0.74 ± 0.03 |  | STAR | 273443 | 273395 | 0 | 48 | yes | ssa-miR-456-5p | abu-miR-456 |  | blast | caggcugguuagaugguuguca | gcaggcaucuucccagccuaca | gcaggcaucuucccagccuacauguagauccaggaaucugcaggcugguuagaugguuguca | NODE\_679149\_length\_21682\_cov\_30.839037:3380..3442:- |
| NODE\_843581\_length\_95893\_cov\_29.638430\_46539 | 1.3e+5 | 0.74 ± 0.03 |  | TRUE | 272528 | 270690 | 22 | 1816 | no | Dre-Mir-192-P1\_5p | gmo-miR-192-5p |  | blast | augaccuaugaauugacagcc | ccugucaguucuguaggccacu | augaccuaugaauugacagccagugauuuccagccucugccugucaguucuguaggccacu | NODE\_843581\_length\_95893\_cov\_29.638430:58185..58246:- |
| NODE\_159082\_length\_9699\_cov\_26.073822\_12101 | 1.3e+5 | 0.74 ± 0.03 |  | TRUE | 264366 | 264319 | 0 | 47 | yes | fru-miR-10d | ccr-miR-10c |  | blast | uacccuguagaaccgaaugugu | cagauuggguucuuggggagucu | uacccuguagaaccgaauguguguggagcugccucagucacagauuggguucuuggggagucu | NODE\_159082\_length\_9699\_cov\_26.073822:2548..2611:- |
| NODE\_737847\_length\_83181\_cov\_28.914331\_43393 | 1.3e+5 | 0.74 ± 0.03 |  | STAR | 261978 | 260950 | 5 | 1023 | yes | ssa-miR-23a-5p | abu-miR-23c |  | blast | aucacauugccagggauuucc | gggauuccuggcagagugauuu | gggauuccuggcagagugauuugguugugauguaauguaaaucacauugccagggauuucc | NODE\_737847\_length\_83181\_cov\_28.914331:29901..29962:+ |
| NODE\_108926\_length\_1673\_cov\_21.221758\_8648 | 1.3e+5 | 0.74 ± 0.03 |  | TRUE | 261652 | 260058 | 0 | 1594 | yes | ola-miR-137 |  |  | blast | uauugcuugagaauacgcguag | cacggguauucuuggguugau | cacggguauucuuggguugauaauacagaugucgauguuauugcuugagaauacgcguag | NODE\_108926\_length\_1673\_cov\_21.221758:1212..1272:- |
| NODE\_158815\_length\_31362\_cov\_32.049549\_12085 | 1.2e+5 | 0.74 ± 0.03 |  | TRUE | 254885 | 194350 | 1 | 60534 | yes | Dno-Mir-1388\_5p | abu-miR-1388 |  | blast | aggacuguccaaccugagaau | aucucagguucgucagcccauga | aggacuguccaaccugagaauggugauuauaggcucaaucucagguucgucagcccauga | NODE\_158815\_length\_31362\_cov\_32.049549:4355..4415:- |
| NODE\_159462\_length\_14900\_cov\_25.055771\_12144 | 1.2e+5 | 0.74 ± 0.03 |  | STAR | 235443 | 205686 | 5 | 29752 | yes | dre-miR-140-5p | abu-miR-140 |  | blast | accacaggguagaaccacggacg | cagugguuuuacccuaugguag | cagugguuuuacccuaugguaggugacaucaugcuguucuaccacaggguagaaccacggacg | NODE\_159462\_length\_14900\_cov\_25.055771:7440..7503:- |
| NODE\_113246\_length\_73161\_cov\_29.120228\_8893 | 1.1e+5 | 0.74 ± 0.03 |  | TRUE | 226122 | 225645 | 1 | 476 | yes | Hsa-Mir-193-P2a-v2\_3p | abu-miR-365 |  | blast | uaaugccccuaaaaauccuuau | agggacuuuuaggggcagcugug | agggacuuuuaggggcagcuguguuuuauuaacccagucauaaugccccuaaaaauccuuau | NODE\_113246\_length\_73161\_cov\_29.120228:27143..27205:+ |
| NODE\_36833\_length\_4245\_cov\_23.718021\_3285 | 1.1e+5 | 0.74 ± 0.03 |  | TRUE | 216651 | 211930 | 1 | 4720 | yes | Xtr-Mir-499\_5p | abu-miR-499 |  | blast | uuaagacuugcagugauguuu | aacaucacuuuaagucugugcu | uuaagacuugcagugauguuuagggcaaugaucacaugaacaucacuuuaagucugugcu | NODE\_36833\_length\_4245\_cov\_23.718021:1243..1303:- |
| NODE\_122954\_length\_12001\_cov\_28.862261\_9583 | 1.0e+5 | 0.74 ± 0.03 |  | TRUE | 209421 | 208427 | 3 | 991 | yes | abu-miR-15b | dre-miR-15c |  | blast | uagcagcgcaucaugguuuga | cgaaccauaauuugcugcuuua | uagcagcgcaucaugguuugaaacaauguggaaaaggugcgaaccauaauuugcugcuuua | NODE\_122954\_length\_12001\_cov\_28.862261:9589..9650:- |
| NODE\_40695\_length\_11784\_cov\_28.186693\_3556 | 1.0e+5 | 0.74 ± 0.03 |  | TRUE | 207922 | 207594 | 5 | 323 | yes | dre-miR-27e | abu-miR-27c |  | blast | uucacaguggcuaaguucagu | agaacuuagcucauuagugagc | agaacuuagcucauuagugagcauuacacaaaaaaggaaauuguucacaguggcuaaguucagu | NODE\_40695\_length\_11784\_cov\_28.186693:5396..5460:- |
| NODE\_843582\_length\_56377\_cov\_27.843359\_46548 | 1.0e+5 | 0.74 ± 0.03 |  | TRUE | 206411 | 206135 | 25 | 251 | yes | dre-miR-27e | abu-miR-27c |  | blast | uucacaguggcuaaguucagu | agagcuuagcuaauuggugagc | agagcuuagcuaauuggugagcauugaucccugcuauguguuguucacaguggcuaaguucagu | NODE\_843582\_length\_56377\_cov\_27.843359:11392..11456:- |
| NODE\_139951\_length\_15026\_cov\_24.779049\_10810 | 1.0e+5 | 0.74 ± 0.03 |  | TRUE | 202508 | 201762 | 446 | 300 | yes | abu-miR-737 | abu-miR-737 |  | blast | guuuuuuuagguuuugauuuuu | aaucaaaaccuaaagaaaaua | guuuuuuuagguuuugauuuuuguagcguuggacgagaaaaucaaaaccuaaagaaaaua | NODE\_139951\_length\_15026\_cov\_24.779049:636..696:+ |
| NODE\_388526\_length\_6635\_cov\_21.866014\_25987 | 9.3e+4 | 0.74 ± 0.03 |  | STAR | 183932 | 177023 | 0 | 6909 | yes | abu-miR-135a-3p | abu-miR-135b |  | blast | uauggcuuuuuauuccuaucuga | uauagggauggaagccaugc | uauggcuuuuuauuccuaucugacuguacuuaugguucauauagggauggaagccaugc | NODE\_388526\_length\_6635\_cov\_21.866014:1395..1454:- |
| NODE\_888968\_length\_19186\_cov\_25.020849\_47410 | 8.9e+4 | 0.74 ± 0.03 |  | TRUE | 176377 | 161840 | 8 | 14529 | yes | abu-miR-139 | abu-miR-139 |  | blast | ucuacagugcaugugucuccagu | uggagacccagcucuguugga | ucuacagugcaugugucuccaguauguuagugaugcuacuggagacccagcucuguugga | NODE\_888968\_length\_19186\_cov\_25.020849:2889..2949:+ |
| NODE\_267094\_length\_15764\_cov\_28.790663\_18892 | 8.1e+4 | 0.74 ± 0.03 |  | STAR | 159787 | 151122 | 1 | 8664 | yes | dre-miR-455-3p | abu-miR-455 |  | blast | uaugugcccuuggacuacauc | gcaguccaugggcauauacacu | uaugugcccuuggacuacaucguggaagccagcaccaugcaguccaugggcauauacacu | NODE\_267094\_length\_15764\_cov\_28.790663:8024..8084:+ |
| NODE\_361068\_length\_55440\_cov\_31.381025\_24308 | 8.1e+4 | 0.74 ± 0.03 |  | STAR | 159787 | 151122 | 1 | 8664 | yes | dre-miR-455-3p | abu-miR-455 |  | blast | uaugugcccuuggacuacauc | gcaguccaugggcauauacacu | uaugugcccuuggacuacaucguggaagccagcaccaugcaguccaugggcauauacacu | NODE\_361068\_length\_55440\_cov\_31.381025:1585..1645:+ |
| NODE\_266954\_length\_252\_cov\_206.527771\_18886 | 7.5e+4 | 0.74 ± 0.03 |  | STAR | 148314 | 145221 | 0 | 3093 | no | oni-miR-10643 |  |  | blast | agaacugccaacggucacuuau | aaguggcuuuggucagcuuugg | aaguggcuuuggucagcuuuggcaaaugcaccagaacugccaacggucacuuau | NODE\_266954\_length\_252\_cov\_206.527771:88..142:- |
| NODE\_14160\_length\_4312\_cov\_28.413265\_1410 | 7.4e+4 | 0.74 ± 0.03 |  | TRUE | 146339 | 145529 | 0 | 810 | yes | abu-miR-458 | abu-miR-458 |  | blast | auagcucuuuaaaugguacugc | agcgccauuuucagagcuau | agcgccauuuucagagcuauaagagugaaaguuaucauagcucuuuaaaugguacugc | NODE\_14160\_length\_4312\_cov\_28.413265:441..499:- |
| NODE\_31633\_length\_24210\_cov\_26.296076\_2813 | 7.4e+4 | 0.74 ± 0.03 |  | TRUE | 145854 | 136640 | 2 | 9212 | no | abu-miR-725 | abu-miR-725 |  | blast | uucagucauuguuucuggucgu | agcuggaaacuuugccugggaau | agcuggaaacuuugccugggaauuucugugcucaaauuucagucauuguuucuggucgu | NODE\_31633\_length\_24210\_cov\_26.296076:14020..14079:+ |
| NODE\_71492\_length\_24686\_cov\_28.957102\_5848 | 6.7e+4 | 0.74 ± 0.03 |  | TRUE | 131744 | 129524 | 3 | 2217 | yes | abu-miR-187 | abu-miR-187 |  | blast | ucgugucuuguguugcagccagu | ggcugcaacacaggacaugggu | ggcugcaacacaggacauggguccugccucuccuccccgcucgugucuuguguugcagccagu | NODE\_71492\_length\_24686\_cov\_28.957102:3809..3872:- |
| NODE\_330003\_length\_22733\_cov\_28.230062\_22572 | 6.3e+4 | 0.74 ± 0.03 |  | STAR | 124474 | 122878 | 0 | 1596 | yes | dre-miR-23b-5p | abu-miR-23c |  | blast | aucacauugccagggauuacc | ggguuccuggcgugcugauuu | ggguuccuggcgugcugauuugugacuuaugauaaaaucacauugccagggauuacc | NODE\_330003\_length\_22733\_cov\_28.230062:12223..12280:- |
| NODE\_970102\_length\_82249\_cov\_29.933956\_48302 | 6.1e+4 | 0.74 ± 0.03 |  | STAR | 120217 | 119887 | 10 | 320 | yes | ola-miR-184-5p | abu-miR-184a |  | blast | uggacggagaacugauaagggc | ccuuaucacuuuuccagcccagc | ccuuaucacuuuuccagcccagcuauagauucuguauccguuggacggagaacugauaagggc | NODE\_970102\_length\_82249\_cov\_29.933956:33665..33728:- |
| NODE\_493274\_length\_72062\_cov\_32.388943\_31735 | 4.9e+4 | 0.74 ± 0.03 |  | STAR | 97720 | 65676 | 3 | 32041 | yes | ssa-miR-734-5p | abu-miR-734 |  | blast | uaaaugcugcagaauugugcu | gaacuauucugcaacauuuguu | gaacuauucugcaacauuuguugaugugguucugcaaguaaaugcugcagaauugugcu | NODE\_493274\_length\_72062\_cov\_32.388943:64806..64865:+ |
| NODE\_605150\_length\_9835\_cov\_25.334520\_37360 | 4.6e+4 | 0.74 ± 0.03 |  | TRUE | 90922 | 83094 | 52 | 7776 | yes | oni-miR-130b-5p | oni-miR-130b-5p |  | blast | acucuuucccuguugcacuacu | cagugcaauaaugaaagggcau | acucuuucccuguugcacuacugugggagaugcagcaagcagugcaauaaugaaagggcau | NODE\_605150\_length\_9835\_cov\_25.334520:7550..7611:+ |
| NODE\_368641\_length\_8004\_cov\_24.659670\_24639 | 4.6e+4 | 0.74 ± 0.03 |  | TRUE | 90579 | 90465 | 20 | 94 | yes | abu-miR-135b | abu-miR-135b |  | blast | uauggcuuucuauuccuaugug | acauagggucuaaagccauugg | uauggcuuucuauuccuaugugauuuucucuggcaugucacauagggucuaaagccauugg | NODE\_368641\_length\_8004\_cov\_24.659670:6429..6490:- |
| NODE\_61882\_length\_8366\_cov\_29.400190\_5216 | 4.6e+4 | 0.74 ± 0.03 |  | TRUE | 90448 | 90177 | 1 | 270 | yes | abu-miR-135b | abu-miR-135b |  | blast | uauggcuuucuauuccuaugug | auguaggguuuaaagccauuggau | uauggcuuucuauuccuaugugaguucuuucuaacaugucauguaggguuuaaagccauuggau | NODE\_61882\_length\_8366\_cov\_29.400190:2206..2270:+ |
| NODE\_370046\_length\_26410\_cov\_31.608671\_24727 | 4.2e+4 | 0.74 ± 0.03 |  | STAR | 83900 | 83580 | 0 | 320 | yes | ola-miR-184-5p | abu-miR-184a |  | blast | uggacggagaacugauaaggg | ccuuaucacuuuuccagcccagc | ccuuaucacuuuuccagcccagcuaucuauuaaauguuuguuggacggagaacugauaaggg | NODE\_370046\_length\_26410\_cov\_31.608671:2038..2100:+ |
| NODE\_819352\_length\_6437\_cov\_28.091812\_45876 | 4.1e+4 | 0.74 ± 0.03 |  | STAR | 80787 | 66037 | 0 | 14750 | yes | pny-miR-101b | abu-miR-101a |  | blast | uacaguacuaugauaacugaag | ucaguuaucaugguaccggugcu | ucaguuaucaugguaccggugcugugucccucucaaguacaguacuaugauaacugaag | NODE\_819352\_length\_6437\_cov\_28.091812:2615..2674:- |
| NODE\_373244\_length\_60022\_cov\_29.876278\_25070 | 4.1e+4 | 0.74 ± 0.03 |  | STAR | 80618 | 49582 | 3 | 31033 | yes | dre-miR-22b-5p | abu-miR-22a |  | blast | aagcugccaguugaagagcugu | cguucuucacuggcuagcuuu | cguucuucacuggcuagcuuuaugucccacgccccacacuaaagcugccaguugaagagcugu | NODE\_373244\_length\_60022\_cov\_29.876278:24225..24288:- |
| NODE\_931091\_length\_50119\_cov\_32.049145\_47919 | 3.9e+4 | 0.74 ± 0.03 |  | TRUE | 76852 | 76835 | 0 | 17 | yes | oni-miR-7552 | eel-miR-7552-5p |  | blast | uuacaauuaaaggauauuucu | gaaauaucucuuaauuguuuga | uuacaauuaaaggauauuucucguggauguaauaaaaacggaaauaucucuuaauuguuuga | NODE\_931091\_length\_50119\_cov\_32.049145:37164..37226:- |
| NODE\_35690\_length\_19945\_cov\_32.644421\_3184 | 3.8e+4 | 0.74 ± 0.03 |  | TRUE | 74551 | 73016 | 0 | 1535 | yes | abu-miR-153c | abu-miR-153a |  | blast | uugcauagucauaaaaaugagc | gucauuuuugugguuugcagcu | gucauuuuugugguuugcagcuaguacucuggcuccaguugcauagucauaaaaaugagc | NODE\_35690\_length\_19945\_cov\_32.644421:14385..14445:- |
| NODE\_406774\_length\_56195\_cov\_27.250521\_26789 | 3.7e+4 | 0.74 ± 0.03 |  | TRUE | 73370 | 47871 | 21 | 25478 | yes | abu-miR-210 | abu-miR-210 |  | blast | agccacugacuaacgcacauug | cugugcgugugacagcggcu | agccacugacuaacgcacauugugccaguuuccaguuccacugugcgugugacagcggcu | NODE\_406774\_length\_56195\_cov\_27.250521:35579..35639:- |
| NODE\_346428\_length\_36481\_cov\_29.114826\_23418 | 3.6e+4 | 0.74 ± 0.03 |  | TRUE | 71724 | 71598 | 0 | 126 | yes | abu-miR-183 | hhi-miR-183 |  | blast | uauggcacugguagaauucacu | ugaauuaccauagggccauaa | uauggcacugguagaauucacugugagagcucacuaucagugaauuaccauagggccauaa | NODE\_346428\_length\_36481\_cov\_29.114826:2683..2744:+ |
| NODE\_439707\_length\_38012\_cov\_31.405767\_28675 | 3.6e+4 | 0.74 ± 0.03 |  | TRUE | 71712 | 71586 | 0 | 126 | yes | abu-miR-183 | hhi-miR-183 |  | blast | uauggcacugguagaauucacu | ugaauuaccauagggccauaa | uauggcacugguagaauucacugucacagcacacuaucagugaauuaccauagggccauaa | NODE\_439707\_length\_38012\_cov\_31.405767:15824..15885:- |
| NODE\_228282\_length\_7873\_cov\_24.512384\_16596 | 3.4e+4 | 0.74 ± 0.03 |  | TRUE | 67549 | 66374 | 46 | 1129 | yes | abu-miR-138 | abu-miR-138 |  | blast | agcugguguugugaaucaggccg | gcuauuucacaacaccagggu | agcugguguugugaaucaggccgaugacacacaccucuuauaacccggcuauuucacaacaccagggu | NODE\_228282\_length\_7873\_cov\_24.512384:4838..4906:+ |
| NODE\_572602\_length\_10371\_cov\_29.585190\_35959 | 3.3e+4 | 0.74 ± 0.03 |  | TRUE | 66434 | 65995 | 0 | 439 | yes | abu-miR-138 | abu-miR-138 |  | blast | agcugguguugugaaucaggccg | gcuuuuucacaacaccaggguu | agcugguguugugaaucaggccgcugaagugucacgcacggcuuuuucacaacaccaggguu | NODE\_572602\_length\_10371\_cov\_29.585190:869..931:- |
| NODE\_284796\_length\_21521\_cov\_29.432880\_19894 | 3.3e+4 | 0.74 ± 0.03 |  | TRUE | 65564 | 65408 | 34 | 122 | yes | abu-miR-138 | abu-miR-138 |  | blast | agcugguguugugaaucaggccg | gcuacuucccaacaccagggu | agcugguguugugaaucaggccgccacaguccagggaccggcuacuucccaacaccagggu | NODE\_284796\_length\_21521\_cov\_29.432880:1440..1501:+ |
| NODE\_346428\_length\_36481\_cov\_29.114826\_23422 | 3.2e+4 | 0.74 ± 0.03 |  | STAR | 64662 | 64280 | 1 | 381 | yes | dre-miR-182-3p | hhi-miR-182 |  | blast | uuuggcaaugguagaacucaca | ugguucuagacuugccaacu | uuuggcaaugguagaacucacacuggugagguagauggauccggugguucuagacuugccaacu | NODE\_346428\_length\_36481\_cov\_29.114826:3410..3474:+ |
| NODE\_346428\_length\_36481\_cov\_29.114826\_23420 | 3.0e+4 | 0.74 ± 0.03 |  | TRUE | 59833 | 59622 | 10 | 201 | yes | abu-miR-96 | hhi-miR-96 |  | blast | uuuggcacuagcacauuuuugcu | caauuauguguagugccaauau | uuuggcacuagcacauuuuugcuucuguauauauacuuugagcaauuauguguagugccaauau | NODE\_346428\_length\_36481\_cov\_29.114826:2974..3038:+ |
| NODE\_439707\_length\_38012\_cov\_31.405767\_28673 | 3.0e+4 | 0.74 ± 0.03 |  | TRUE | 59651 | 59650 | 0 | 1 | yes | abu-miR-96 | hhi-miR-96 |  | blast | uuuggcacuagcacauuuuugcu | caaucauguguggugccaauau | uuuggcacuagcacauuuuugcuuuguuucuccucuuuugagcaaucauguguggugccaauau | NODE\_439707\_length\_38012\_cov\_31.405767:15651..15715:- |
| NODE\_627358\_length\_7463\_cov\_29.470053\_38594 | 2.9e+4 | 0.74 ± 0.03 |  | TRUE | 57681 | 57548 | 0 | 133 | yes | abu-miR-8160a | abu-miR-8160a |  | blast | agaauaaugccagcagucgguc | ccagcacugguguuauugaga | agaauaaugccagcagucggucguggugucccagggaccagcacugguguuauugaga | NODE\_627358\_length\_7463\_cov\_29.470053:2316..2374:+ |
| NODE\_555985\_length\_33019\_cov\_32.158787\_35090 | 2.9e+4 | 0.74 ± 0.03 |  | TRUE | 57204 | 50315 | 2 | 6887 | yes | abu-miR-7132a-5p | abu-miR-7132a-5p |  | blast | gacuuggucaaagcuccucagc | ugaggcguuuagaacaaguuca | gacuuggucaaagcuccucagcagauugaagugacucugaggcguuuagaacaaguuca | NODE\_555985\_length\_33019\_cov\_32.158787:18007..18066:+ |
| NODE\_605754\_length\_30933\_cov\_29.700871\_37421 | 2.8e+4 | 0.74 ± 0.03 |  | STAR | 55071 | 51628 | 0 | 3443 | yes | ssa-miR-489-5p |  |  | blast | gugacaucauauguacggcugc | uggucguauguaugacgucauu | uggucguauguaugacgucauuuacuucaaugcuuggagugacaucauauguacggcugc | NODE\_605754\_length\_30933\_cov\_29.700871:29841..29901:+ |
| NODE\_565970\_length\_45167\_cov\_32.156639\_35622 | 2.2e+4 | 0.74 ± 0.03 |  | TRUE | 43508 | 42006 | 2 | 1500 | yes | dre-miR-722 |  |  | blast | uuuugcagaaacguuucagauu | uuugaaacguuuuagcuaaa | uuugaaacguuuuagcuaaaaauguuuccaugcucaagguguuuuuugcagaaacguuucagauu | NODE\_565970\_length\_45167\_cov\_32.156639:25680..25745:- |
| NODE\_904193\_length\_18512\_cov\_28.863333\_47606 | 2.1e+4 | 0.74 ± 0.03 |  | STAR | 42931 | 42006 | 2 | 923 | yes | ssa-miR-722-5p |  |  | blast | uuuugcagaaacguuucagauu | uuugaaacguuuuagccaaaa | uuugaaacguuuuagccaaaaauguuuccauggucaagguguuuuuugcagaaacguuucagauu | NODE\_904193\_length\_18512\_cov\_28.863333:2217..2282:- |
| NODE\_303539\_length\_39784\_cov\_31.875326\_20973 | 1.9e+4 | 0.74 ± 0.03 |  | TRUE | 38860 | 38615 | 0 | 245 | yes | abu-miR-93 | abu-miR-20a |  | blast | aaaagugcuguuugugcaggu | acugcaaaaccagcacuucagg | aaaagugcuguuugugcagguagcggucauccaccuacugcaaaaccagcacuucagg | NODE\_303539\_length\_39784\_cov\_31.875326:23211..23269:- |
| NODE\_246211\_length\_22333\_cov\_31.187391\_17762 | 1.7e+4 | 0.74 ± 0.03 |  | STAR | 33355 | 33347 | 7 | 1 | yes | ssa-miR-429-5p | abu-miR-429b |  | blast | uaauacugucugguaaugccgu | gucuuaccagacaugguuagau | gucuuaccagacaugguuagauguaauuauuggugucuaauacugucugguaaugccgu | NODE\_246211\_length\_22333\_cov\_31.187391:14806..14865:- |
| NODE\_257389\_length\_35345\_cov\_31.947687\_18271 | 1.6e+4 | 0.74 ± 0.03 |  | STAR | 31581 | 29649 | 0 | 1932 | yes | ipu-miR-460 | ccr-miR-460-5p |  | blast | ccugcauuguacacacugugc | cacagcgcauacaauguggaug | ccugcauuguacacacugugcguauaaaugucauaagcacagcgcauacaauguggaug | NODE\_257389\_length\_35345\_cov\_31.947687:12559..12618:- |
| NODE\_142891\_length\_5647\_cov\_28.864706\_10971 | 1.3e+4 | 0.74 ± 0.03 |  | TRUE | 27068 | 26150 | 23 | 895 | yes | dre-miR-730 | dre-miR-730 |  | blast | uccucauugugcaugcugugug | cacagcgccugcaauguggagg | uccucauugugcaugcuguguguauuucuguauguaccacacagcgccugcaauguggagg | NODE\_142891\_length\_5647\_cov\_28.864706:4394..4455:- |
| NODE\_303539\_length\_39784\_cov\_31.875326\_20975 | 1.2e+4 | 0.74 ± 0.03 |  | TRUE | 25463 | 23616 | 76 | 1771 | yes | abu-miR-106 | abu-miR-20a |  | blast | uaaagugcuuacagugcagguag | acugcauugugagcacuucuuu | uaaagugcuuacagugcagguagugaugauaaucuggccuacugcauugugagcacuucuuu | NODE\_303539\_length\_39784\_cov\_31.875326:23402..23464:- |
| NODE\_173406\_length\_10615\_cov\_25.613283\_13045 | 1.2e+4 | 0.74 ± 0.03 |  | TRUE | 23767 | 23208 | 0 | 559 | yes | abu-miR-99a | abu-miR-99a |  | blast | agaccuguagauacaagcuugu | caaguucggaucuacggguuu | agaccuguagauacaagcuuguggugucacuuaacacaaguucggaucuacggguuu | NODE\_173406\_length\_10615\_cov\_25.613283:9625..9682:- |
| NODE\_70677\_length\_15497\_cov\_31.035490\_5796 | 1.1e+4 | 0.74 ± 0.03 |  | STAR | 23403 | 8896 | 0 | 14507 | yes | abu-miR-190a-5p |  |  | blast | acuauauaucaaacauauuccu | ugauauguuugauauauuaggu | ugauauguuugauauauuagguuguuauucagucccaacuauauaucaaacauauuccu | NODE\_70677\_length\_15497\_cov\_31.035490:10155..10214:- |
| NODE\_657938\_length\_54945\_cov\_29.131041\_40050 | 1.1e+4 | 0.74 ± 0.03 |  | STAR | 23403 | 8896 | 0 | 14507 | yes | abu-miR-190a-5p |  |  | blast | acuauauaucaaacauauuccu | ugauauguuugauauauuaggu | ugauauguuugauauauuagguuguuauucaguccaacuauauaucaaacauauuccu | NODE\_657938\_length\_54945\_cov\_29.131041:52626..52684:- |
| NODE\_965170\_length\_60159\_cov\_30.094299\_48278 | 1.1e+4 | 0.74 ± 0.03 |  | STAR | 22197 | 17635 | 0 | 4562 | yes | abu-miR-135c-3p | abu-miR-135b |  | blast | uauggcuuuuuauuccuauguga | auguaggaacagaagccauuuu | uauggcuuuuuauuccuaugugaugaugaaaaguguucauguaggaacagaagccauuuu | NODE\_965170\_length\_60159\_cov\_30.094299:38773..38833:+ |
| NODE\_345812\_length\_10942\_cov\_30.590111\_23386 | 1.0e+4 | 0.74 ± 0.03 |  | TRUE | 19695 | 19690 | 0 | 5 | yes | abu-miR-457 | abu-miR-15a |  | blast | uagcagcacaucauuacuggua | ccaguauguuaugugcugcuucu | uagcagcacaucauuacugguauuuuucacugcauuaccaguauguuaugugcugcuucu | NODE\_345812\_length\_10942\_cov\_30.590111:8637..8697:+ |
| NODE\_749189\_length\_52450\_cov\_27.729570\_43722 | 7.7e+3 | 0.74 ± 0.03 |  | TRUE | 15201 | 15081 | 0 | 120 | yes | Aca-Mir-155\_5p | dre-miR-2194 |  | blast | uuaaugcuaaucgugauaggggu | caccuaacauguuagcauuagcu | uuaaugcuaaucgugauagggguuguucuuauuaacagacaccuaacauguuagcauuagcu | NODE\_749189\_length\_52450\_cov\_27.729570:7730..7792:+ |
| NODE\_289240\_length\_14019\_cov\_28.901491\_20157 | 6.9e+3 | 0.74 ± 0.03 |  | STAR | 13623 | 13612 | 0 | 11 | yes | ssa-miR-196a-4-3p | abu-miR-196b |  | blast | uagguaguuucauguuguuggg | ucggcaacaagaaacugccuuga | uagguaguuucauguuguugggguuggcuuccuggcucggcaacaagaaacugccuuga | NODE\_289240\_length\_14019\_cov\_28.901491:12405..12464:- |
| NODE\_109328\_length\_10974\_cov\_29.501732\_8690 | 6.7e+3 | 0.74 ± 0.03 |  | TRUE | 13213 | 13178 | 0 | 35 | yes | abu-miR-10552 | abu-miR-10552 |  | blast | uccuccaugcacuuugaugacu | ccucagaaugcauggggauggu | uccuccaugcacuuugaugacuuauuuugucaaacuguccucagaaugcauggggauggu | NODE\_109328\_length\_10974\_cov\_29.501732:246..306:+ |
| NODE\_88227\_length\_1190\_cov\_20.568068\_7197 | 6.4e+3 | 0.74 ± 0.03 |  | TRUE | 12724 | 12645 | 4 | 75 | yes | abu-miR-19c | abu-miR-19c |  | blast | ugugcaaauccaugcaaagcucu | aguuuugaugguuuguucucagc | aguuuugaugguuuguucucagcuucugaaagacucugcugugcaaauccaugcaaagcucu | NODE\_88227\_length\_1190\_cov\_20.568068:478..540:+ |
| NODE\_490675\_length\_37324\_cov\_27.685215\_31296 | 5.7e+3 | 0.74 ± 0.03 |  | STAR | 11257 | 9290 | 5 | 1962 | yes | abu-miR-7132b-3p | abu-miR-7132a-5p |  | blast | gacuugguccaagcuccucagu | ugagaaguuuugaacaaguaua | gacuugguccaagcuccucagugcuguuuauauaccugagaaguuuugaacaaguaua | NODE\_490675\_length\_37324\_cov\_27.685215:1544..1602:+ |
| NODE\_23828\_length\_4869\_cov\_33.493736\_2268 | 5.6e+3 | 0.74 ± 0.03 |  | STAR | 11018 | 6745 | 3 | 4270 | yes | mze-miR-7133 | abu-miR-7133-3p |  | blast | uaguuugauacacagcacaau | gauguugaguaucaaacuguau | gauguugaguaucaaacuguauguuauguaugcuauauauaguuugauacacagcacaau | NODE\_23828\_length\_4869\_cov\_33.493736:595..655:- |
| NODE\_597153\_length\_4469\_cov\_30.493622\_37082 | 5.6e+3 | 0.74 ± 0.03 |  | STAR | 11018 | 6745 | 3 | 4270 | yes | mze-miR-7133 | abu-miR-7133-3p |  | blast | uaguuugauacacagcacaau | gauguugaguaucaaacuguau | gauguugaguaucaaacuguauguuauguaugcuauauauaguuugauacacagcacaau | NODE\_597153\_length\_4469\_cov\_30.493622:1034..1094:- |
| NODE\_311493\_length\_4305\_cov\_33.220673\_21432 | 5.6e+3 | 0.74 ± 0.03 |  | STAR | 11016 | 6745 | 1 | 4270 | yes | mze-miR-7133 | abu-miR-7133-3p |  | blast | uaguuugauacacagcacaau | gauguugaguaucaaacuguau | gauguugaguaucaaacuguauguuauguaugcuauuuauaguuugauacacagcacaau | NODE\_311493\_length\_4305\_cov\_33.220673:1741..1801:- |
| NODE\_946148\_length\_69798\_cov\_29.425756\_48089 | 5.3e+3 | 0.74 ± 0.03 |  | STAR | 10564 | 8680 | 32 | 1852 | yes | abu-miR-147 |  |  | blast | acagaaucguuucugcacaaacu | gugugcggaaaugcuucugcuc | acagaaucguuucugcacaaacuaggacgcuuugacacucagugugcggaaaugcuucugcuc | NODE\_946148\_length\_69798\_cov\_29.425756:21531..21594:- |
| NODE\_345812\_length\_10942\_cov\_30.590111\_23384 | 5.3e+3 | 0.74 ± 0.03 |  | STAR | 10415 | 9669 | 105 | 641 | yes | abu-miR-15c-3p | abu-miR-15a |  | blast | uagcagcacaucauguuuugc | caaaucaucaugugcugccacc | uagcagcacaucauguuuugcagaugugcugaaucgcuucaaaucaucaugugcugccacc | NODE\_345812\_length\_10942\_cov\_30.590111:7589..7650:+ |
| NODE\_863631\_length\_25650\_cov\_30.187290\_47003 | 4.9e+3 | 0.74 ± 0.03 |  | STAR | 9674 | 7784 | 2 | 1888 | yes | abu-miR-29c-5p |  |  | blast | ucuagcaccauaugaaaucagu | cugauuucauuuggugacguaga | cugauuucauuuggugacguagauguuuguacacagucucuagcaccauaugaaaucagu | NODE\_863631\_length\_25650\_cov\_30.187290:16437..16497:+ |
| NODE\_756492\_length\_10799\_cov\_22.423836\_43926 | 4.5e+3 | 0.74 ± 0.03 |  | TRUE | 8973 | 7932 | 0 | 1041 | yes | abu-miR-7147 | abu-miR-7147 |  | blast | uguaccaugcugguagccagug | uggugaccagcguugugccu | uguaccaugcugguagccagugugugguaggcuuugcuggugaccagcguugugccu | NODE\_756492\_length\_10799\_cov\_22.423836:3223..3280:+ |
| NODE\_607860\_length\_14514\_cov\_29.504410\_37529 | 4.4e+3 | 0.74 ± 0.03 |  | STAR | 8738 | 8444 | 0 | 294 | yes | ssa-miR-551-5p | abu-miR-551 |  | blast | gcgacccauccuuuguuucuga | gaaaccaaguguggguguggccu | gaaaccaaguguggguguggccugaaaaagaaauauggcgacccauccuuuguuucuga | NODE\_607860\_length\_14514\_cov\_29.504410:1739..1798:+ |
| NODE\_290735\_length\_5326\_cov\_27.341156\_20266 | 4.3e+3 | 0.74 ± 0.03 |  | TRUE | 8609 | 4660 | 0 | 3949 | yes | Aca-Mir-130-P2a\_5p | dre-miR-301c-5p |  | blast | gcucugacuucauugcacuacu | uagugcaauaguauugucaaagc | gcucugacuucauugcacuacuguaucagacaacuaguagugcaauaguauugucaaagc | NODE\_290735\_length\_5326\_cov\_27.341156:929..989:+ |
| NODE\_843581\_length\_95893\_cov\_29.638430\_46541 | 4.3e+3 | 0.74 ± 0.03 |  | TRUE | 8513 | 8348 | 0 | 165 | yes | Aca-Mir-194-P1\_5p | dre-miR-194b |  | blast | uguaacagcaacuccaugugga | ccaguggaggugcuguuacu | uguaacagcaacuccauguggaagcugugucaguuccaguggaggugcuguuacu | NODE\_843581\_length\_95893\_cov\_29.638430:58381..58436:- |
| NODE\_648968\_length\_29776\_cov\_27.678164\_39648 | 3.7e+3 | 0.74 ± 0.03 |  | STAR | 7423 | 5684 | 98 | 1641 | yes | abu-miR-33-5p | mze-miR-33b |  | blast | caauguaccugcagugcaaca | gugcauuguaguugcauugc | gugcauuguaguugcauugcaugugugucucagcugagugcaauguaccugcagugcaaca | NODE\_648968\_length\_29776\_cov\_27.678164:1954..2015:- |
| NODE\_650421\_length\_14855\_cov\_31.549580\_39769 | 2.8e+3 | 0.74 ± 0.03 |  | STAR | 5515 | 3176 | 0 | 2339 | yes | abu-miR-449b-5p | abu-miR-449b-3p |  | blast | cagcucacacugcucugcuacu | uggcaguguaauguuagcuga | uggcaguguaauguuagcugacguuucuucagccagcucacacugcucugcuacu | NODE\_650421\_length\_14855\_cov\_31.549580:9501..9556:+ |
| NODE\_575362\_length\_13461\_cov\_35.437782\_36087 | 2.5e+3 | 0.74 ± 0.03 |  | TRUE | 5006 | 1470 | 0 | 3536 | yes | abu-miR-728a | abu-miR-728a |  | blast | auacuaaguauacuacguuuac | aaauguaguagacuauaaguauac | aaauguaguagacuauaaguauacaugaaacucagaaaguauacuaaguauacuacguuuac | NODE\_575362\_length\_13461\_cov\_35.437782:10820..10882:+ |
| NODE\_113246\_length\_73161\_cov\_29.120228\_8892 | 2.2e+3 | 0.74 ± 0.03 |  | TRUE | 4481 | 2292 | 0 | 2189 | yes | abu-miR-193-5p | abu-miR-193-5p |  | blast | ugggucuuugcgggcaagguga | aacuggccuacaaagucccagu | ugggucuuugcgggcaaggugaguccucauuucauucaacuggccuacaaagucccagu | NODE\_113246\_length\_73161\_cov\_29.120228:24330..24389:+ |
| NODE\_34963\_length\_5205\_cov\_31.040346\_3138 | 2.2e+3 | 0.74 ± 0.03 |  | TRUE | 4347 | 3126 | 0 | 1221 | no | abu-miR-10544 | abu-miR-10544 |  | blast | uaggcgugucacugcgugucaca | ugcgcacggggccacgcccugc | uaggcgugucacugcgugucacagucacugcuugcgcacggggccacgcccugc | NODE\_34963\_length\_5205\_cov\_31.040346:4803..4857:+ |
| NODE\_411411\_length\_56313\_cov\_31.971605\_27013 | 2.1e+3 | 0.74 ± 0.03 |  | TRUE | 4296 | 3779 | 0 | 517 | yes | abu-miR-10545-5p | abu-miR-10545-5p |  | blast | uaagucucacaccagugcaaaac | cugcucaggugugggaccaua | uaagucucacaccagugcaaaacaaaaaucaugacgcugcucaggugugggaccaua | NODE\_411411\_length\_56313\_cov\_31.971605:25167..25224:- |
| NODE\_24835\_length\_13316\_cov\_31.743092\_2331 | 2.1e+3 | 0.74 ± 0.03 |  | STAR | 4211 | 4138 | 0 | 73 | yes | oni-miR-3120-5p | abu-miR-3120 |  | blast | ugcacagcaaguguagauaggc | cugucugugccugcuguacaggu | cugucugugccugcuguacagguuggaggauauucugcacagcaaguguagauaggc | NODE\_24835\_length\_13316\_cov\_31.743092:12458..12515:+ |
| NODE\_575868\_length\_19092\_cov\_31.856483\_36108 | 1.6e+3 | 0.74 ± 0.03 |  | STAR | 3310 | 2594 | 0 | 716 | yes | abu-miR-726 | ssa-miR-23a-3-5p |  | blast | ggaauuccgcuaguucugaacu | uucacuacuagcagaacucaga | ggaauuccgcuaguucugaacuauucguguuugucaaaaguucacuacuagcagaacucaga | NODE\_575868\_length\_19092\_cov\_31.856483:5951..6013:- |
| NODE\_80209\_length\_10732\_cov\_25.379147\_6542 | 1.2e+3 | 0.74 ± 0.03 |  | TRUE | 2379 | 2058 | 0 | 321 | yes | abu-miR-196b | abu-miR-196b |  | blast | uagguagucucauguuguugggc | ccacaacacaaaacugccuuga | uagguagucucauguuguugggcuauauuauuucucccacaacacaaaacugccuuga | NODE\_80209\_length\_10732\_cov\_25.379147:6729..6787:+ |
| NODE\_479052\_length\_25577\_cov\_27.990187\_30801 | 1.1e+3 | 0.74 ± 0.03 |  | TRUE | 2173 | 1996 | 1 | 176 | yes | oni-miR-34b | oni-miR-34b |  | blast | cugguaguguaguuagugauu | ucagcuaacgacacugccuaua | cugguaguguaguuagugauuagcguguaagaacaaucagcuaacgacacugccuaua | NODE\_479052\_length\_25577\_cov\_27.990187:4185..4243:- |
| NODE\_617795\_length\_22272\_cov\_31.273573\_38022 | 1.0e+3 | 0.74 ± 0.03 |  | TRUE | 2062 | 1796 | 20 | 246 | yes | oni-miR-728b | oni-miR-728b |  | blast | aaauguaguagacuuuaaguauac | auacuaaguacacuacguuuau | aaauguaguagacuuuaaguauacauguggaacuggggaguauacuaaguacacuacguuuau | NODE\_617795\_length\_22272\_cov\_31.273573:19430..19493:+ |
| NODE\_748659\_length\_25102\_cov\_21.648952\_43684 | 1.0e+3 | 0.74 ± 0.03 |  | TRUE | 2042 | 1643 | 8 | 391 | yes | abu-miR-33-5p | abu-miR-33-5p |  | blast | gugcauuguaguugcauugc | caaugugucugcagugcaguac | gugcauuguaguugcauugcaugauccugaagccgagugcaaugugucugcagugcaguac | NODE\_748659\_length\_25102\_cov\_21.648952:22895..22956:+ |
| NODE\_303195\_length\_29610\_cov\_26.739008\_20952 | 1.0e+3 | 0.74 ± 0.03 |  | TRUE | 1968 | 1902 | 0 | 66 | yes | abu-miR-10557 | abu-miR-10557 |  | blast | acuggcauagauuugaaacuugu | ugguuucaguucaugccagaac | ugguuucaguucaugccagaacaguauaugcaaaucuacuggcauagauuugaaacuugu | NODE\_303195\_length\_29610\_cov\_26.739008:8647..8707:- |
| NODE\_733921\_length\_4407\_cov\_23.070570\_43319 | 9.4e+2 | 0.74 ± 0.03 |  | STAR | 1835 | 862 | 0 | 973 | yes | abu-miR-10559 | dre-miR-196a-3p |  | blast | cugcaacguugacuuugaaccc | cuucaaagucaacguugcaucc | cuucaaagucaacguugcauccuuggaaacaaguuggcugcaacguugacuuugaaccc | NODE\_733921\_length\_4407\_cov\_23.070570:2668..2727:+ |
| NODE\_737847\_length\_83181\_cov\_28.914331\_43401 | 6.9e+2 | 0.74 ± 0.03 |  | TRUE | 1354 | 1281 | 0 | 73 | yes | ipu-miR-7565 | ipu-miR-7565 |  | blast | uuccugcugaacugagccagu | uguuaucagcucaguaggcac | uuccugcugaacugagccagugcuuguuaacaucuaacuguuaucagcucaguaggcac | NODE\_737847\_length\_83181\_cov\_28.914331:33077..33136:- |
| NODE\_843582\_length\_56377\_cov\_27.843359\_46544 | 6.8e+2 | 0.74 ± 0.03 |  | TRUE | 1334 | 1333 | 0 | 1 | yes | ipu-miR-7565 | ipu-miR-7565 |  | blast | uuccugcugaacugagccagu | ugauaccaguucaguaggcac | uuccugcugaacugagccaguuuucagguauaacacugauaccaguucaguaggcac | NODE\_843582\_length\_56377\_cov\_27.843359:10510..10567:+ |
| NODE\_333530\_length\_15311\_cov\_29.662203\_22871 | 6.7e+2 | 0.74 ± 0.03 |  | STAR | 1309 | 1218 | 1 | 90 | yes | ssa-miR-190b-3p | abu-miR-190a-5p |  | blast | ugauauguuugauauucgguugu | acuaaauaucagacauauuccu | ugauauguuugauauucgguuguucuugugcuuuauaaugucaacuaaauaucagacauauuccu | NODE\_333530\_length\_15311\_cov\_29.662203:804..869:- |
| NODE\_653314\_length\_17074\_cov\_25.003748\_39899 | 6.4e+2 | 0.74 ± 0.03 |  | STAR | 1264 | 763 | 1 | 500 | yes | oni-miR-10606 |  |  | blast | cggccccgugauguauuugcag | ugcaaaucagaacgcggcacga | ugcaaaucagaacgcggcacgagcagacgccgcacaucuguguguuuaaucucggccccgugauguauuugcag | NODE\_653314\_length\_17074\_cov\_25.003748:8369..8443:+ |
| NODE\_388526\_length\_6635\_cov\_21.866014\_25989 | 4.6e+2 | 0.74 ± 0.03 |  | TRUE | 910 | 809 | 0 | 101 | yes | oni-miR-10555 | oni-miR-10555 |  | blast | ccucaagcugcuguugcucguc | gaaauagcagcagcuugucugg | ccucaagcugcuguugcucgucuguggcgauccagaaauagcagcagcuugucugg | NODE\_388526\_length\_6635\_cov\_21.866014:3075..3131:- |
| NODE\_996854\_length\_26195\_cov\_26.376827\_48470 | 2.9e+2 | 0.74 ± 0.03 |  | TRUE | 585 | 500 | 0 | 85 | yes | oni-miR-10585 |  |  | blast | ugaugaaaucacucuauguuccu | ggauagguugauuucauugcau | ugaugaaaucacucuauguuccugcuugcugauacaggauagguugauuucauugcau | NODE\_996854\_length\_26195\_cov\_26.376827:410..468:+ |
| NODE\_739733\_length\_16472\_cov\_25.832201\_43466 | 2.9e+2 | 0.74 ± 0.03 |  | TRUE | 578 | 571 | 0 | 7 | yes | ipu-miR-199b |  |  | blast | aaccaaugugcagacuacuguu | agguagucugaacacugggca | aaccaaugugcagacuacuguucagcugcagccugaugaacagguagucugaacacugggca | NODE\_739733\_length\_16472\_cov\_25.832201:10108..10170:+ |
| NODE\_143037\_length\_2081\_cov\_31.187410\_10978 | 2.0e+2 | 0.74 ± 0.03 |  | STAR | 408 | 395 | 0 | 13 | no | oni-miR-10659 |  |  | blast | uccucucuccaaggcgucucuu | agaggcguccuggagagguaga | agaggcguccuggagagguagagccugcgcgucucuccucucuccaaggcgucucuu | NODE\_143037\_length\_2081\_cov\_31.187410:90..147:- |
| NODE\_246094\_length\_13317\_cov\_27.872944\_17743 | 1.6e+2 | 0.74 ± 0.03 |  | TRUE | 314 | 231 | 0 | 83 | yes | abu-miR-10550-5p | abu-miR-10550-5p |  | blast | cagcacaucgcugaacugaagcc | cucaguuuagaagugugccucg | cagcacaucgcugaacugaagccgugauuucagugagcucaguuuagaagugugccucg | NODE\_246094\_length\_13317\_cov\_27.872944:6374..6433:- |
| NODE\_102620\_length\_20330\_cov\_31.291245\_8154 | 1.0e+2 | 0.74 ± 0.03 |  | TRUE | 193 | 145 | 0 | 48 | yes | oni-miR-10708 | oni-miR-10708 |  | blast | cugcuguuguguaauggguugc | agccuguuacacuacaucauac | agccuguuacacuacaucauacaauacaccacuguccugcuguuguguaauggguugc | NODE\_102620\_length\_20330\_cov\_31.291245:19978..20036:- |
| NODE\_769019\_length\_3524\_cov\_19.447502\_44331 | 9.6e+1 | 0.74 ± 0.03 |  | TRUE | 182 | 174 | 2 | 6 | yes | ipu-miR-199b | ipu-miR-199b |  | blast | uaaccaaugugcagacuacugu | agguagucugaacacugggau | uaaccaaugugcagacuacuguacaccaguaagauccugaacagguagucugaacacugggau | NODE\_769019\_length\_3524\_cov\_19.447502:2584..2647:- |
| NODE\_749189\_length\_52450\_cov\_27.729570\_43736 | 9.1e+1 | 0.74 ± 0.03 |  | TRUE | 172 | 143 | 0 | 29 | yes | oni-miR-17b | oni-miR-17b |  | blast | accugcacuguaagcacuuuggc | uguaagugccuccacugcaguag | uguaagugccuccacugcaguagguauuccauaguacuaccugcacuguaagcacuuuggc | NODE\_749189\_length\_52450\_cov\_27.729570:26827..26888:- |
| NODE\_274557\_length\_14227\_cov\_31.720743\_19367 | 7.8e+1 | 0.74 ± 0.03 |  | TRUE | 155 | 148 | 0 | 7 | no | oni-miR-10729 | oni-miR-10729 |  | blast | uaaggcugaaccaauggacu | uucaaaggucucagccucauc | uaaggcugaaccaauggacuguacguuucuaucaaguucaaaggucucagccucauc | NODE\_274557\_length\_14227\_cov\_31.720743:12690..12747:- |
| NODE\_49970\_length\_24944\_cov\_26.430405\_4337 | 6.7e+1 | 0.74 ± 0.03 |  | TRUE | 151 | 148 | 0 | 3 | no | oni-miR-10739 | oni-miR-10739 |  | blast | uuagaucaauaaguauggaau | ucaugacauuguuaaccaa | ucaugacauuguuaaccaacagaaaucggcaaugggccucucauucuuuuagaucaauaaguauggaau | NODE\_49970\_length\_24944\_cov\_26.430405:955..1024:+ |
| NODE\_302945\_length\_29936\_cov\_30.279196\_20931 | 6.6e+1 | 0.74 ± 0.03 |  | STAR | 131 | 81 | 0 | 50 | yes | oni-miR-10757 |  |  | blast | ugacgugguagcuuugcucagc | cuugucagaacucccacucaugu | cuugucagaacucccacucauguaaacuucuucuucaugacgugguagcuuugcucagc | NODE\_302945\_length\_29936\_cov\_30.279196:10316..10375:+ |
| NODE\_81529\_length\_29484\_cov\_30.358160\_6633 | 5.4e+1 | 0.74 ± 0.03 |  | TRUE | 99 | 86 | 0 | 13 | yes | oni-miR-10895 | oni-miR-10895 |  | blast | auccgggacaugcagcucucc | gcuggccguaugucccaggaca | auccgggacaugcagcucuccggaaacucaaucagcuggccguaugucccaggaca | NODE\_81529\_length\_29484\_cov\_30.358160:23540..23596:- |
| NODE\_254550\_length\_8267\_cov\_29.561026\_18084 | 5.6 | 0.75 ± 0.03 |  | TRUE | 147 | 147 | 0 | 0 | yes | oni-miR-10592 | abu-miR-132-5p |  | blast | uccguggccccugaugcggagc | cccgcaccacggccgcggagg | uccguggccccugaugcggagcugaugagcggccacagcccgcaccacggccgcggagg | NODE\_254550\_length\_8267\_cov\_29.561026:8256..8315:+ |
| NODE\_760618\_length\_35970\_cov\_25.822741\_44024 | 5.5 | 0.75 ± 0.03 |  | TRUE | 1806609 | 1806234 | 0 | 375 | yes | Aca-Mir-451\_5p | dre-miR-451 |  | blast | aaaccguuaccauuacuga | uaaugguaaggguucuacagc | aaaccguuaccauuacugaguuuuaguaaugguaaggguucuacagc | NODE\_760618\_length\_35970\_cov\_25.822741:3112..3159:+ |
| NODE\_31995\_length\_2043\_cov\_17.994125\_2853 | 5.3 | 0.75 ± 0.03 |  | STAR | 63800 | 63725 | 14 | 61 | yes | dre-miR-219-3p | abu-miR-219-5p |  | blast | ugauuguccaaacgcaauucu | ggaguuguggauggacaucacgc | ugauuguccaaacgcaauucuugcaugugcuuuugugaaaccaggaguuguggauggacaucacgc | NODE\_31995\_length\_2043\_cov\_17.994125:795..861:- |
| NODE\_438529\_length\_23636\_cov\_25.419361\_28519 | 5.3 | 0.75 ± 0.03 |  | TRUE | 453 | 449 | 0 | 4 | yes | oni-miR-10880 | oni-miR-10880 |  | blast | uuuuccucugaucugccuccucu | agagagcagggcagagggagac | uuuuccucugaucugccuccucucuuucaucaagaaaaccacagagagcagggcagagggagac | NODE\_438529\_length\_23636\_cov\_25.419361:22867..22931:- |
| NODE\_51980\_length\_8291\_cov\_27.670969\_4588 | 5.3 | 0.75 ± 0.03 |  | STAR | 3229 | 1897 | 0 | 1332 | yes | Dre-Mir-130-P2b1\_5p | ccr-miR-130a |  | blast | cagugcaauaguauugucauagc | gcuuugacgauguugcacuacu | gcuuugacgauguugcacuacuguaucaucgcugaagcagugcaauaguauugucauagc | NODE\_51980\_length\_8291\_cov\_27.670969:1227..1287:- |
| NODE\_313715\_length\_10635\_cov\_23.819370\_21561 | 5.2 | 0.75 ± 0.03 |  | STAR | 8046 | 7949 | 0 | 97 | yes | dre-miR-137-5p | abu-miR-137 |  | blast | uuauugcuuaagaauacgcgu | acggguauucuuggguggaua | acggguauucuuggguggauaauacagaucacguuguuauugcuuaagaauacgcgu | NODE\_313715\_length\_10635\_cov\_23.819370:2595..2652:+ |
| NODE\_50564\_length\_882\_cov\_24.903627\_4410 | 5.2 | 0.75 ± 0.03 |  | TRUE | 3284715 | 3279563 | 0 | 5152 | yes | Cli-Let-7-P12\_5p | abu-let-7d |  | blast | ugagguaguaguuugugcuguu | cugcgcaaucuacugccuugcu | ugagguaguaguuugugcuguuggucggguugucauauuaccccuugcggagaugacugcgcaaucuacugccuugcu | NODE\_50564\_length\_882\_cov\_24.903627:335..413:+ |
| NODE\_433108\_length\_26495\_cov\_32.568409\_28115 | 5.2 | 0.75 ± 0.03 |  | TRUE | 181 | 180 | 1 | 0 | yes | oni-miR-10890 | oni-miR-10890 |  | blast | ugcacucugcacacccacuucu | agguggaugugugguggggu | agguggauguguggugggguugaaggaaucugauuuggggauuguggucaugcuccugcacucugcacacccacuucu | NODE\_433108\_length\_26495\_cov\_32.568409:9447..9525:+ |
| NODE\_178157\_length\_5580\_cov\_29.226166\_13444 | 5.2 | 0.75 ± 0.03 |  | TRUE | 114 | 114 | 0 | 0 | yes | abu-miR-10560 | abu-miR-10560 |  | blast | gaacgcacucguacuuucagug | cuguugagcgagugggugccg | gaacgcacucguacuuucagugagcguuaacaugaaucacuguugagcgagugggugccg | NODE\_178157\_length\_5580\_cov\_29.226166:2421..2481:+ |
| NODE\_618453\_length\_89288\_cov\_30.092106\_38087 | 5.1 | 0.75 ± 0.03 |  | TRUE | 6744 | 6744 | 0 | 0 | yes | abu-miR-218b | abu-miR-218b |  | blast | uugugcuugaucuaaccaugc | augguugugccaagcaccuug | uugugcuugaucuaaccaugcagugcaucuucuguccaugguugugccaagcaccuug | NODE\_618453\_length\_89288\_cov\_30.092106:10550..10608:- |
| NODE\_716047\_length\_27522\_cov\_26.407528\_42556 | 5.1 | 0.75 ± 0.03 |  | STAR | 1229619 | 1222438 | 0 | 7181 | yes | dre-miR-338-5p | dre-miR-338-3p |  | blast | uccagcaucagugauuuuguu | aacaauauccuggugcugccugagu | aacaauauccuggugcugccugagugcucccuaaagacuccagcaucagugauuuuguu | NODE\_716047\_length\_27522\_cov\_26.407528:1855..1914:- |
| NODE\_330003\_length\_22733\_cov\_28.230062\_22566 | 5.1 | 0.75 ± 0.03 |  | TRUE | 1293 | 1281 | 0 | 12 | yes | ipu-miR-7565 | ipu-miR-7565 |  | blast | uuccugcugaacugagccagu | uuaucagcucaguaggcacaga | uuccugcugaacugagccagugauucuguaggacugauuaucagcucaguaggcacaga | NODE\_330003\_length\_22733\_cov\_28.230062:8414..8473:+ |
| NODE\_23088\_length\_9463\_cov\_36.666702\_2197 | 5.1 | 0.75 ± 0.03 |  | TRUE | 6732 | 5937 | 0 | 795 | yes | abu-miR-2187a | abu-miR-2187a |  | blast | uuaauuaguauagccuguuuu | uuuacaggcuaugcuaaucugu | uuaauuaguauagccuguuuuagugauaucagcaauucuuuacaggcuaugcuaaucugu | NODE\_23088\_length\_9463\_cov\_36.666702:5069..5129:- |
| NODE\_749189\_length\_52450\_cov\_27.729570\_43731 | 5.1 | 0.75 ± 0.03 |  | STAR | 121963 | 121592 | 1 | 370 | yes | ipu-miR-19b | abu-miR-19c |  | blast | ugugcaaauccaugcaaaacu | aguuuugcugguuugcauucagc | aguuuugcugguuugcauucagcuuuaaugguguuugcugugcaaauccaugcaaaacu | NODE\_749189\_length\_52450\_cov\_27.729570:27673..27732:+ |
| NODE\_259850\_length\_64569\_cov\_30.972200\_18470 | 5.1 | 0.75 ± 0.03 |  | TRUE | 392595 | 381368 | 1 | 11226 | yes | abu-miR-103 | abu-miR-103 |  | blast | agcagcauuguacagggcuauga | agccucuuuacagugcugccuug | agccucuuuacagugcugccuugucuaaucauguucaagcagcauuguacagggcuauga | NODE\_259850\_length\_64569\_cov\_30.972200:27610..27670:- |
| NODE\_230850\_length\_24267\_cov\_30.165575\_16753 | 5.1 | 0.75 ± 0.03 |  | TRUE | 76 | 76 | 0 | 0 | yes | oni-miR-10733 | oni-miR-10733 |  | blast | uucccagacucucauuccagc | ugagguuugaguguuuugguggaagu | ugagguuugaguguuuugguggaaguaguuuuccuuuacuucccagacucucauuccagc | NODE\_230850\_length\_24267\_cov\_30.165575:10779..10839:+ |
| NODE\_63321\_length\_37455\_cov\_34.655827\_5296 | 5.0 | 0.75 ± 0.03 |  | TRUE | 23460 | 23389 | 0 | 71 | yes | abu-miR-29d | abu-miR-29d |  | blast | uagcaccauuugaaaucgg | cugguuucagauggugucuuaga | cugguuucagauggugucuuagaguacaaaaucccaucuagcaccauuugaaaucgg | NODE\_63321\_length\_37455\_cov\_34.655827:24051..24108:- |
| NODE\_661702\_length\_31525\_cov\_26.808311\_40377 | 4.9 | 0.75 ± 0.03 |  | TRUE | 1037744 | 1037288 | 0 | 456 | yes | Asu-Mir-124\_3p | dre-miR-124-3p |  | blast | uaaggcacgcggugaaugcc | aguguucacaguggaccuugau | aguguucacaguggaccuugauuuaaaaucagucaauuaaggcacgcggugaaugcc | NODE\_661702\_length\_31525\_cov\_26.808311:4720..4777:+ |
| NODE\_203563\_length\_12708\_cov\_24.490320\_14998 | 4.9 | 0.75 ± 0.03 |  | STAR | 229 | 146 | 0 | 83 | yes | oni-miR-10795 | oni-miR-10862 |  | blast | uguccucugcugcugcuucuuc | agagucacgcugagaggaaacaauu | uguccucugcugcugcuucuucacacagaucuugugaagaagagucacgcugagaggaaacaauu | NODE\_203563\_length\_12708\_cov\_24.490320:603..668:+ |
| NODE\_142594\_length\_20943\_cov\_32.484650\_10946 | 4.9 | 0.75 ± 0.03 |  | TRUE | 68066 | 68061 | 0 | 5 | yes | Bfl-Mir-219\_5p | abu-miR-219-5p |  | blast | ugauuguccaaacgcaauucu | ggaguuguggcuggacaucaugc | ugauuguccaaacgcaauucuuguauuuaauauccauauccaggaguuguggcuggacaucaugc | NODE\_142594\_length\_20943\_cov\_32.484650:8715..8780:+ |
| NODE\_271874\_length\_6325\_cov\_29.794941\_19185 | 4.9 | 0.75 ± 0.03 |  | TRUE | 1038231 | 1037288 | 0 | 943 | yes | Asu-Mir-124\_3p | dre-miR-124-3p |  | blast | uaaggcacgcggugaaugcc | cguguucacggcggaccuuga | cguguucacggcggaccuugauuuauuagccauacaauuaaggcacgcggugaaugcc | NODE\_271874\_length\_6325\_cov\_29.794941:2996..3054:+ |
| NODE\_106232\_length\_8611\_cov\_25.181976\_8443 | 4.8 | 0.75 ± 0.03 |  | TRUE | 23537 | 23500 | 0 | 37 | yes | abu-miR-10553 | abu-miR-10553 |  | blast | gugccauaucugacuguacgca | ugcguuaucagcgauauagc | gugccauaucugacuguacgcaauuguaucauuauauugcguuaucagcgauauagc | NODE\_106232\_length\_8611\_cov\_25.181976:4216..4273:- |
| NODE\_171072\_length\_858\_cov\_18.970863\_12906 | 4.8 | 0.75 ± 0.03 |  | STAR | 1043021 | 1037288 | 0 | 5733 | yes | dre-miR-124-5p | dre-miR-124-3p |  | blast | uaaggcacgcggugaaugcc | cguguucacagcggaccuugau | cguguucacagcggaccuugauuuaaugucuuacaauuaaggcacgcggugaaugcc | NODE\_171072\_length\_858\_cov\_18.970863:336..393:- |
| NODE\_51980\_length\_8291\_cov\_27.670969\_4590 | 4.7 | 0.75 ± 0.03 |  | STAR | 27058 | 18305 | 18 | 8735 | yes | dre-miR-130c-5p | ccr-miR-130a |  | blast | cagugcaauauuaaaagggc | gcccuuuuucuguuguacuacu | gcccuuuuucuguuguacuacugugaaaucagaugagcagugcaauauuaaaagggc | NODE\_51980\_length\_8291\_cov\_27.670969:1504..1561:- |
| NODE\_374989\_length\_4954\_cov\_32.948727\_25175 | 4.7 | 0.75 ± 0.03 |  | TRUE | 284743 | 284721 | 22 | 0 | yes | Sha-Mir-129-P1\_5p | dre-miR-722 |  | blast | cuuuuugcggucugggcuugc | gaccaugauugcagcugagcg | gaccaugauugcagcugagcgcuguuguucuuuucaggucuuuuugcggucugggcuugc | NODE\_374989\_length\_4954\_cov\_32.948727:2141..2201:+ |
| NODE\_374469\_length\_4418\_cov\_26.432322\_25152 | 4.6 | 0.75 ± 0.03 |  | TRUE | 1046907 | 1037288 | 0 | 9619 | yes | Asu-Mir-124\_3p | dre-miR-124-3p |  | blast | uaaggcacgcggugaaugcc | uguguucacaguggaccuugau | uguguucacaguggaccuugauuuaauuucaauacaauuaaggcacgcggugaaugcc | NODE\_374469\_length\_4418\_cov\_26.432322:3481..3539:- |
| NODE\_749189\_length\_52450\_cov\_27.729570\_43727 | 4.6 | 0.75 ± 0.03 |  | STAR | 55600 | 55547 | 1 | 52 | yes | dre-miR-19a-5p | abu-miR-19c |  | blast | ugugcaaaucuaugcaaaacu | aguuuugcauaguugcacua | aguuuugcauaguugcacuacaagaauauaugaguugugcaaaucuaugcaaaacu | NODE\_749189\_length\_52450\_cov\_27.729570:27329..27385:+ |
| NODE\_317583\_length\_42051\_cov\_31.426601\_21816 | 4.4 | 0.75 ± 0.03 |  | STAR | 1043050 | 1037288 | 31 | 5731 | yes | dre-miR-124-5p | dre-miR-124-3p |  | blast | uaaggcacgcggugaaugcc | cguguucacagcggaccuugau | cguguucacagcggaccuugauuuaaauguccauacaauuaaggcacgcggugaaugcc | NODE\_317583\_length\_42051\_cov\_31.426601:40935..40994:- |
| NODE\_540432\_length\_66809\_cov\_33.349190\_34318 | 4.2 | 0.75 ± 0.03 |  | TRUE | 148 | 148 | 0 | 0 | yes | oni-miR-17b | oni-miR-17b |  | blast | accugcacuguaagcacuuuggc | ugaaagugccuucacugcaguag | ugaaagugccuucacugcaguagaauugaacaaaacuaccugcacuguaagcacuuuggc | NODE\_540432\_length\_66809\_cov\_33.349190:60460..60520:- |
| NODE\_450908\_length\_48195\_cov\_31.381699\_29143 | 4.2 | 0.75 ± 0.03 |  | TRUE | 81 | 81 | 0 | 0 | yes | oni-miR-132d | ccr-miR-132b |  | blast | accauggcuguagacuguuacc | uaacaaucuaaugccacgguca | accauggcuguagacuguuaccacuggugcuguuacuacaguaacaaucuaaugccacgguca | NODE\_450908\_length\_48195\_cov\_31.381699:12752..12815:- |
| NODE\_33541\_length\_42464\_cov\_30.625048\_3028 | 4.2 | 0.75 ± 0.03 |  | TRUE | 604584 | 603587 | 24 | 973 | yes | Aca-Mir-181-P2a\_5p | dre-miR-181a-5p |  | blast | aacauucauugcugucgguggg | cucacugaacgaugaaugcaa | aacauucauugcugucgguggguuuaacugugugggagagcucacugaacgaugaaugcaa | NODE\_33541\_length\_42464\_cov\_30.625048:4335..4396:+ |
| NODE\_654140\_length\_10930\_cov\_25.100000\_39930 | 4.0 | 0.75 ± 0.03 |  | TRUE | 605066 | 603425 | 0 | 1641 | yes | Aca-Mir-181-P2a\_5p | dre-miR-181a-5p |  | blast | aacauucauugcugucgguggg | cucacugaucaaugaaugcag | aacauucauugcugucgguggguuugcagcagaauaacucacugaucaaugaaugcag | NODE\_654140\_length\_10930\_cov\_25.100000:8907..8965:+ |
| NODE\_50564\_length\_882\_cov\_24.903627\_4409 | 3.8 | 0.70 ± 0.03 |  | TRUE | 3279563 | 3279563 | 0 | 0 | yes | Cli-Let-7-P12\_5p | abu-let-7d |  | blast | ugagguaguaguuugugcuguu | aagcgcaucccuggugccaagu | aagcgcaucccuggugccaaguucuggcugagguaguaguuugugcuguu | NODE\_50564\_length\_882\_cov\_24.903627:307..357:+ |
| NODE\_459453\_length\_54739\_cov\_33.289009\_29617 | 3.2 | 0.70 ± 0.03 |  | TRUE | 72687 | 72413 | 0 | 274 | yes | abu-miR-723b | gmo-miR-723-3p |  | blast | agacaucagauaaaucuguccu | aaggcaguuuugaugauguuac | aaggcaguuuugaugauguuacuuuuuuaauuuuaagaagacaucagauaaaucuguccu | NODE\_459453\_length\_54739\_cov\_33.289009:4978..5038:+ |
| NODE\_254550\_length\_8267\_cov\_29.561026\_18083 | 1.7 | 0.71 ± 0.03 |  | TRUE | 147 | 147 | 0 | 0 | no | oni-miR-10592 | abu-miR-132-5p |  | blast | uccguggccccugaugcggagc | ccugcauggcgggguggcgguga | ccugcauggcgggguggcggugagcuccguggccccugaugcggagc | NODE\_254550\_length\_8267\_cov\_29.561026:8231..8278:+ |
| NODE\_282412\_length\_27058\_cov\_31.342598\_19736 | 1.3 | 0.71 ± 0.03 |  | TRUE | 92397 | 92336 | 0 | 61 | no | mze-miR-8159 | gmo-miR-459-5p |  | blast | ucaguaacuggaaucugucccu | agggccuggcugguuacugcacc | ucaguaacuggaaucugucccugcgugguaaaagugugcagggccuggcugguuacugcacc | NODE\_282412\_length\_27058\_cov\_31.342598:19467..19529:+ |
| NODE\_71492\_length\_24686\_cov\_28.957102\_5847 | 1.0 | 0.71 ± 0.03 |  | TRUE | 129524 | 129524 | 0 | 0 | no | abu-miR-187 | abu-miR-187 |  | blast | ucgugucuuguguugcagccagu | ugucugcccacugaugacgcgugagc | ucgugucuuguguugcagccaguggagcugccugucugcccacugaugacgcgugagc | NODE\_71492\_length\_24686\_cov\_28.957102:3774..3832:- |
| NODE\_615561\_length\_2028\_cov\_24.085798\_37860 | 0.9 | 0.62 ± 0.03 |  | TRUE | 1284 | 1284 | 0 | 0 | no | ipu-miR-7565 | ipu-miR-7565 |  | blast | uuccugcugaacugagccagu | ugucagcucagaaagcacgaggg | uuccugcugaacugagccaguguauuugaucaaacugcugucagcucagaaagcacgaggg | NODE\_615561\_length\_2028\_cov\_24.085798:250..311:+ |
| NODE\_412784\_length\_6493\_cov\_22.596334\_27127 | 0.7 | 0.62 ± 0.03 |  | TRUE | 1913697 | 1913697 | 0 | 0 | no | abu-miR-27c | abu-miR-27c |  | blast | uucacagugguuaaguucugc | agggcugcagccugcaggggaca | uucacagugguuaaguucugccgccacagggcugcagccugcaggggaca | NODE\_412784\_length\_6493\_cov\_22.596334:2179..2229:- |
| NODE\_7241\_length\_8413\_cov\_24.273149\_834 | -0.2 | 0.44 ± 0.03 |  | TRUE | 5713 | 5563 | 0 | 150 | no | nbr-miR-10568 | nbr-miR-10568 |  | blast | uguccggcagaugggaacaucuc | cagauuucacacuugccacauc | uguccggcagaugggaacaucucucucuuucuaaauuuugacagauuucacacuugccacauc | NODE\_7241\_length\_8413\_cov\_24.273149:7221..7284:+ |
| NODE\_525082\_length\_27855\_cov\_28.354012\_33534 | -1.5 | 0.29 ± 0.04 |  | TRUE | 492795 | 492795 | 0 | 0 | no | Mml-Mir-153-P1\_3p | abu-miR-153a |  | blast | uugcauagucacaaaagugauc | agacugugacucuacaacc | uugcauagucacaaaagugaucauuggagacugugacucuacaacc | NODE\_525082\_length\_27855\_cov\_28.354012:21918..21964:+ |
| NODE\_66567\_length\_3308\_cov\_24.986094\_5488 | -1.9 | 0.29 ± 0.04 |  | TRUE | 3165953 | 3165953 | 0 | 0 | no | Cte-Mir-9\_5p | dre-miR-9-5p |  | blast | ucuuugguuaucuagcuguau | acacauguggauuauugcugaagauu | acacauguggauuauugcugaagauugggaaguggguuguuaucuuugguuaucuagcuguau | NODE\_66567\_length\_3308\_cov\_24.986094:53..116:- |
| NODE\_345812\_length\_10942\_cov\_30.590111\_23385 | -2.0 | 0.29 ± 0.04 |  | TRUE | 19692 | 19690 | 2 | 0 | no | abu-miR-457 | abu-miR-15a |  | blast | uagcagcacaucauuacuggua | cgugugaguguuggucaca | cgugugaguguuggucacauucagugcaucuggccguagcagcacaucauuacuggua | NODE\_345812\_length\_10942\_cov\_30.590111:8601..8659:+ |
| NODE\_749189\_length\_52450\_cov\_27.729570\_43721 | -2.2 | 0.22 ± 0.04 |  | TRUE | 15084 | 15081 | 3 | 0 | no | Aca-Mir-155\_5p | dre-miR-2194 |  | blast | uuaaugcuaaucgugauaggggu | cgacauugagguaagcaugggagu | cgacauugagguaagcaugggaguaaagcacgggagccuucaagcauggucccagguugagguuaaugcuaaucgugauaggggu | NODE\_749189\_length\_52450\_cov\_27.729570:7668..7753:+ |
| NODE\_246211\_length\_22333\_cov\_31.187391\_17763 | -2.7 | 0.22 ± 0.04 |  | TRUE | 1013300 | 1013299 | 1 | 0 | no | abu-miR-200a | abu-miR-200a |  | blast | uaacacugucugguaacgaugu | auugugaagggcagcugaacuc | uaacacugucugguaacgauguuuucugggugacugcguggucuccgguauguaaaugacaaauauugugaagggcagcugaacuc | NODE\_246211\_length\_22333\_cov\_31.187391:16104..16190:- |
| NODE\_229551\_length\_8711\_cov\_22.407415\_16682 | -2.8 | 0.22 ± 0.04 |  | TRUE | 300 | 300 | 0 | 0 | no | oni-miR-10683 |  |  | blast | augaaacguguuugauugggca | accauucaaacacgguccacc | accauucaaacacgguccaccuuuuuggcaugaaacguguuugauugggca | NODE\_229551\_length\_8711\_cov\_22.407415:5726..5777:- |
| NODE\_229551\_length\_8711\_cov\_22.407415\_16681 | -2.9 | 0.22 ± 0.04 |  | TRUE | 300 | 300 | 0 | 0 | no | oni-miR-10683 |  |  | blast | augaaacguguuugauugggca | cgucagucaauaaugcgucc | augaaacguguuugauugggcacugcgucagucaauaaugcgucc | NODE\_229551\_length\_8711\_cov\_22.407415:5703..5748:- |
| NODE\_654140\_length\_10930\_cov\_25.100000\_39927 | -3.0 | 0.22 ± 0.04 |  | TRUE | 4031069 | 4031030 | 39 | 0 | no | Aca-Mir-181-P1a\_5p | dre-miR-181a-5p |  | blast | aacauucaacgcugucggugagu | ucaacacacauucaaaugucgu | ucaacacacauucaaaugucgucacucucuggugcccguggggaacauucaacgcugucggugagu | NODE\_654140\_length\_10930\_cov\_25.100000:8594..8660:+ |
| NODE\_36833\_length\_4245\_cov\_23.718021\_3286 | -3.2 | 0.22 ± 0.03 |  | TRUE | 211930 | 211930 | 0 | 0 | no | Xtr-Mir-499\_5p | abu-miR-499 |  | blast | uuaagacuugcagugauguuu | augucacuggagcauggggacg | augucacuggagcauggggacggagguaguuaagacuugcagugauguuu | NODE\_36833\_length\_4245\_cov\_23.718021:1282..1332:- |
| NODE\_122954\_length\_12001\_cov\_28.862261\_9584 | -4.7 | 0.24 ± 0.03 |  | TRUE | 208427 | 208427 | 0 | 0 | no | abu-miR-15b | dre-miR-15c |  | blast | uagcagcgcaucaugguuuga | gaaaauagaugugcccucugccagc | gaaaauagaugugcccucugccagcccuuagacuacuauagcagcgcaucaugguuuga | NODE\_122954\_length\_12001\_cov\_28.862261:9629..9688:- |
| NODE\_49970\_length\_24944\_cov\_26.430405\_4338 | -4.7 | 0.24 ± 0.03 |  | TRUE | 152 | 148 | 0 | 4 | no | oni-miR-10739 | oni-miR-10739 |  | blast | uuagaucaauaaguauggaau | cagacauauuucuugaucuucu | uuagaucaauaaguauggaauguguuccuaacagacauauuucuugaucuucu | NODE\_49970\_length\_24944\_cov\_26.430405:1003..1056:+ |
| NODE\_18233\_length\_5886\_cov\_25.828577\_1819 | -5.4 | 0.23 ± 0.03 |  | TRUE | 3279608 | 3279601 | 6 | 1 | no | Cli-Let-7-P12\_5p | abu-let-7d |  | blast | ugagguaguaguuugugcuguu | uuuuuguacuguuucuu | uuuuuguacuguuucuuucuucguuuucgucuuauuaccacguacuggcugagguaguaguuugugcuguu | NODE\_18233\_length\_5886\_cov\_25.828577:552..623:+ |
| NODE\_617795\_length\_22272\_cov\_31.273573\_38020 | -5.9 | 0.23 ± 0.03 |  | TRUE | 30465 | 30465 | 0 | 0 | no | abu-miR-727b-3p | abu-miR-727b-3p |  | blast | guugaggcgaguugaagacu | ucacgacccggaccucagcug | guugaggcgaguugaagacuuuaagcgcugugcagauucacgacccggaccucagcug | NODE\_617795\_length\_22272\_cov\_31.273573:15357..15415:+ |
| NODE\_142594\_length\_20943\_cov\_32.484650\_10945 | -6.0 | 0.23 ± 0.03 |  | TRUE | 68063 | 68061 | 2 | 0 | no | Bfl-Mir-219\_5p | abu-miR-219-5p |  | blast | ugauuguccaaacgcaauucu | gguugcugugacuguucua | gguugcugugacuguucuaccaaaacuugacagggagucugguguugauuguccaaacgcaauucu | NODE\_142594\_length\_20943\_cov\_32.484650:8670..8736:+ |
| NODE\_158693\_length\_38410\_cov\_31.448738\_12071 | -7.8 | 0.22 ± 0.03 |  | TRUE | 166 | 166 | 0 | 0 | yes | oni-miR-9b |  |  | blast | uucgguuaucuagcuuuauga | auaaacaagaggcugagaa | auaaacaagaggcugagaauuuggaggucauuccuacuuucgguuaucuagcuuuauga | NODE\_158693\_length\_38410\_cov\_31.448738:18863..18922:+ |
| NODE\_291214\_length\_21711\_cov\_26.518124\_20295 | -8.6 | 0.22 ± 0.03 |  | TRUE | 179 | 179 | 0 | 0 | no | oni-miR-9b |  |  | blast | uucgguuaucuagcuuuauga | aggaguguaaugggauu | aggaguguaaugggauucuuguuacuuucgguuaucuagcuuuauga | NODE\_291214\_length\_21711\_cov\_26.518124:12377..12424:+ |
| NODE\_605754\_length\_30933\_cov\_29.700871\_37422 | -1.4e+1 | 0.22 ± 0.03 |  | TRUE | 51628 | 51628 | 0 | 0 | no | Xtr-Mir-489-v1\_3p |  |  | blast | gugacaucauauguacggcugc | aacugcuacaugggacaucagcuc | gugacaucauauguacggcugcuaaacugcuacaugggacaucagcuc | NODE\_605754\_length\_30933\_cov\_29.700871:29879..29927:+ |

  
  

## mature miRBase miRNAs not detected by miRDeep2

  

miRBase precursor idClicking this field will display a pdf of the structure and read signature of the miRNA. | total read countthis is the sum of read counts for the mature and star miRNAs. | mature read count(s)this is the number of reads that map to the miRNA hairpin and are contained in the sequence covered by the mature miRNA, including 2 nts upstream and 5 nts downstream. If more than one mature sequence is given this will be a comma separated list. In parenthesis are normalized read counts shown. | star read countthis is the number of reads that map to the miRNA hairpin and are contained in the sequence covered by the star miRNA, including 2 nts upstream and 5 nts downstream. This field is empty unless a reference star miRNA was given as input to quantifier.pl. If more than one mature sequence is given this will be a comman separated list | remaining readsthis is the number of reads that did not map to any of the mature and star sequences | UCSC browserif a species name was input to miRDeep2, then clicking this field will initiate a UCSC blat search of the miRNA precursor sequence against the reference genome. | NCBI blastnclicking this field will initiate a NCBI blastn search of the miRNA precursor sequence against the nr/nt database (non-redundant collection of all NCBI nucleotide sequences). | miRBase mature sequence(s)this is/are the mature miRNA sequence(s) input to quantifier.pl. | miRBase star sequence(s)this is/are the star miRNA sequence(s) input to quantifier.pl. This field is empty unless a reference star miRNA was given as input to quantifier.pl. | miRBase precursor sequencethis is the precursor miRNA sequence input to quantifier.pl. || tni-mir-152 | 471229 | 290274 |

 0 | 180955 | - | blast | |  | | --- | | ucagugcauaacagaacuuuguc | | |  | | --- | | - | | cugcucaaacucugggcuaaguucugugauacacucugacugugaauggcuaugcuagucagugcauaacagaacuuugucccgg || dre-mir-27d | 74957 | 74799 | 0 | 158 | - | blast | |  | | --- | | uucacaguggcuaaguucuucac | | |  | | --- | | - | | uucugagcgggugcagagcuuggcugauuggugaacgugcauggcuuguguuuuuguucacaguggcuaaguucuucacccgaaaagaa || tni-mir-223 | 31070 | 30184 | 0 | 886 | - | blast | |  | | --- | | ugucaguuugucaaauaccccaa | | |  | | --- | | - | | caggcccuucacuuaguguauuugacaagcuguguuugacacucuguaucugcgagugucaguuugucaaauaccccaagugagg || fru-let-7a-3 | 15899902 | 18884 | 0 | 15881018 | - | blast | |  | | --- | | cuauacagucuauugccuuccu | | |  | | --- | | - | | cagggugagguaguagguuguauaguuuggugggugggauugcccgcccaggugauaacuauacagucuauugccuuccuugaggagcucacug || ccr-mir-727 | 45470 | 16035 | 0 | 29435 | - | blast | |  | | --- | | ucagucuucaauuccucccagc | | |  | | --- | | - | | accuguaugucauuuucagucuucaauuccucccagcccguaaccauggaaacugugaguugaggcgaguugaagacuuaaagugcuguacag || abu-mir-10558 | 8605 | 8364 | 0 | 241 | - | blast | |  | | --- | | auccgcuuggcaucuuucuugg | | |  | | --- | | - | | auccgcuuggcaucuuucuuggccuucaaacaacaauucugaugguaaagauccaaacgggacaca || ssa-mir-216b | 8051 | 8051 | 0 | 0 | - | blast | |  | | --- | | uaaucucagcuggcaacugugag | | |  | | --- | | - | | uaaucucagcuggcaacugugagcaguucaguguucccucucacaguggcuacugggguucug || oni-mir-10558 | 7673 | 6776 | 0 | 897 | - | blast | |  | | --- | | auccguugggcaucuuucuuugcc | | |  | | --- | | - | | auccguugggcaucuuucuuugccuuuagacaauaauucugauggcaaaagagccaaacgggacagg || abu-mir-10556a | 5787 | 5769 | 0 | 18 | - | blast | |  | | --- | | aaagaaaagcgucuggacuucu | | |  | | --- | | - | | aaagaaaagcgucuggacuucuuuucaaguaacuuaaagaaaacccgacgcuuuucuuugc || oni-mir-10597a | 2431 | 1876 | 0 | 555 | - | blast | |  | | --- | | gaugacuggauuuguaaagcgc | | |  | | --- | | - | | gaugacuggauuuguaaagcgcuugggaccaaguaaagcgcuauacaaauacaggccauu || gmo-mir-150 | 1220 | 1220 | 0 | 0 | - | blast | |  | | --- | | ucucccaauccuuguaccaguguc | | |  | | --- | | - | | ucucccaauccuuguaccagugucguggugucggccgacgcugggcaggcuuugggggg || oni-mir-10578 | 1224 | 966 | 0 | 258 | - | blast | |  | | --- | | ugcagugagucagucugaagcu | | |  | | --- | | - | | ugcagugagucagucugaagcugucugacuguaucagcugacagauggcucucugaagu || abu-mir-10564 | 958 | 645 | 0 | 313 | - | blast | |  | | --- | | aacacaacgcgaagaaauaugc | | |  | | --- | | - | | aacacaacgcgaagaaauaugcacuacuaagugcauauuucuucgcguuguguuuu || abu-mir-10556b | 860 | 545 | 0 | 315 | - | blast | |  | | --- | | ggaagaaaaacgucuggacuuc | | |  | | --- | | - | | ggaagaaaaacgucuggacuucuuuaaguuucuugaagaugaaguccagaugcuuuucuuucca || mze-mir-10554-3 | 813 | 488 | 0 | 325 | - | blast | |  | | --- | | ucgagcgcagauucacugagc | | |  | | --- | | - | | ucaguggaucugcguuugacuacuccaccuaggcugcacugucgagcgcagauucacugagc || oni-mir-10627 | 476 | 473 | 0 | 3 | - | blast | |  | | --- | | guuucacggaccggccuuuaaggu | | |  | | --- | | - | | cuccacggaccgguuuaaugucagacaaaaguuucacggaccggccuuuaaggu || dre-mir-1306 | 458 | 375 | 0 | 83 | - | blast | |  | | --- | | ccaccuccccugcaaacgucca | | |  | | --- | | - | | uccaccaccuccccugcaaacguccagugacgcagaggaaauggacguuagcucugguggugauggaca || oni-mir-10622-1 | 1001 | 372 | 0 | 629 | - | blast | |  | | --- | | uguagagucuguguguggagcu | | |  | | --- | | - | | uguagagucuguguguggagcuucugcaguuuguguugaagcguccacacucagcucugcagc || oni-mir-132c | 634 | 313 | 0 | 321 | - | blast | |  | | --- | | agccaugacuguagacuguuacu | | |  | | --- | | - | | agccaugacuguagacuguuacuguacuuugauacuguauaagcaguaagcagucuaaagccaaggugc || abu-mir-10554-2 | 688 | 248 | 0 | 440 | - | blast | |  | | --- | | ucaguggaucugugcucgacagu | | |  | | --- | | - | | ucaguggaucugugcucgacagugcagccuaggcagaguagucgaacgcaaauccucuga || pny-mir-430a-1 | 264 | 220 | 0 | 44 | - | blast | |  | | --- | | uaagugcugauuguuguugugu | | |  | | --- | | - | | acggcaacauaggcacuuauuugaauucugaauauaguaagugcugauuguuguugugu || oni-mir-10573b | 217 | 211 | 0 | 6 | - | blast | |  | | --- | | aacuccaguccucgagggcaug | | |  | | --- | | - | | aacuccaguccucgagggcauggaaaaguugcaugacaccggccuucgaggaauggaguuu || oni-mir-10581d | 361 | 158 | 0 | 203 | - | blast | |  | | --- | | aacguccagacgaacagaauc | | |  | | --- | | - | | ucuguucaucuggacguagcguuucucccacugaaaacguccagacgaacagaauc || abu-mir-10549 | 714 | 153 | 0 | 561 | - | blast | |  | | --- | | uagguaaucaacaauccaggac | | |  | | --- | | - | | cccuguauugcugauuaccuuguggucuuccuguuagguaaucaacaauccaggaca || ipu-mir-3618 | 146 | 143 | 0 | 3 | - | blast | |  | | --- | | gauuuccaauaauugagacagu | | |  | | --- | | - | | gauuuccaauaauugagacaguaauuucuaaaaagcugucuacauuaaugaaaagaac || abu-mir-10548 | 173 | 150 | 0 | 23 | - | blast | |  | | --- | | cguggccugcggugccgugc | | cacggcaccgcaggccccgcc | | |  | | --- | | - | | cguggccugcggugccgugcaggugcguccgccuccucugcacggcaccgcaggccccgccc || oni-mir-10608a | 812 | 126 | 0 | 686 | - | blast | |  | | --- | | ucggcugcccugggaaugccu | | |  | | --- | | - | | gcagacccaggacaugcuggagagauuauaucucucggcugcccugggaaugccu || oni-mir-10605 | 256 | 120 | 0 | 136 | - | blast | |  | | --- | | caaguguaacaggacaaccugu | | |  | | --- | | - | | caaguguaacaggacaaccugucuaaagaguaacagguuguccuguuacucuuu || oni-mir-10562 | 158 | 118 | 0 | 40 | - | blast | |  | | --- | | agccaaugagcgugcggacuca | | |  | | --- | | - | | cccgcacgcucauuggauaacgaugugucaaucucgagcuauuagccaaugagcgugcggacuca || oni-mir-10697a-1 | 368 | 108 | 0 | 260 | - | blast | |  | | --- | | augggguucuguuggcuucauc | | |  | | --- | | - | | gguggccucagaaucucaugucaucuuuucacagaugaugggguucuguuggcuucauc || oni-mir-10938 | 89 | 89 | 0 | 0 | - | blast | |  | | --- | | ucuguuugugucuguuuucaga | | |  | | --- | | - | | ucuguuugugucuguuuucagauaugagucuggauagcagacucauaucugugagcauuuacaaccaccuuugaua || oni-mir-10698 | 86 | 78 | 0 | 8 | - | blast | |  | | --- | | ucaugcauggcuucuuacacu | | |  | | --- | | - | | guaagaaccauaccauucaaugucaaucacacugucaugcauggcuucuuacacu || oni-mir-10632-1 | 82 | 73 | 0 | 9 | - | blast | |  | | --- | | ucacuggacugagaggaguaucu | | |  | | --- | | - | | ucacuggacugagaggaguaucugagaagcugacgcucuccucuuugucuccggugaag || oni-mir-10848 | 84 | 70 | 0 | 14 | - | blast | |  | | --- | | uccgaccuugguccgcuggacu | | |  | | --- | | - | | acugcggcugacaggucggacgggagaggugcucugccauccucguccgaccuugguccgcuggacu || oni-mir-10902 | 179 | 62 | 0 | 117 | - | blast | |  | | --- | | ugacacugguggugcugaucgcccc | | |  | | --- | | - | | ucacagcaagaaugcaccagugauggccccucacaggaagguggugacacugguggugcugaucgcccc || oni-mir-10714 | 68 | 54 | 0 | 14 | - | blast | |  | | --- | | uacaggagcuacugccacguucc | | |  | | --- | | - | | uacaggagcuacugccacguuccacgggugaugaggcagcugugcguggcucagcucugcagc || oni-mir-10856 | 57 | 53 | 0 | 4 | - | blast | |  | | --- | | cugauccaggaccaucucagug | | |  | | --- | | - | | cugauccaggaccaucucagugcugcugcugguuguugugguuuuacugggaugguuuuggaucaggu || oni-mir-10871 | 579 | 50 | 0 | 529 | - | blast | |  | | --- | | aagggccuguauuuguauagcgc | | |  | | --- | | - | | aagggccuguauuuguauagcgcuuuacuuaguuccuaaggaccccaaagcgcuuuacauuauauacagucaucca || oni-mir-10702 | 51 | 49 | 0 | 2 | - | blast | |  | | --- | | ugaugaaggagauagcacugugc | | |  | | --- | | - | | cagugcuaucuccuucaucacacugggugugaugaaggagauagcacugugc || oni-mir-10900b | 64 | 42 | 0 | 22 | - | blast | |  | | --- | | uugaucuggagguaguucugcu | | |  | | --- | | - | | uguggaacucuaccaccagcuccuugguuuuauugguguugaucuggagguaguucugcu || oni-mir-10800 | 42 | 39 | 0 | 3 | - | blast | |  | | --- | | aaggugacaguuguuguggaca | | |  | | --- | | - | | aaggugacaguuguuguggacaccuaccuuaaccccuuggugguucucacuccgguggccaucgcuaacugucaccaaga || ccr-mir-124a | 41 | 38 | 0 | 3 | - | blast | |  | | --- | | ucaagguccgcugugaacacg | | |  | | --- | | - | | uuucuuggcauucaccgcgugccuuaauuguauggaaauuuaaaucaagguccgcugugaacacgaagaaaa || oni-mir-10934 | 39 | 38 | 0 | 1 | - | blast | |  | | --- | | ccgccgugucagaucuacugga | | |  | | --- | | - | | ccgccgugucagaucuacuggaacuggcucguccagcagauaccgacauggaucgca || oni-mir-10909 | 37 | 37 | 0 | 0 | - | blast | |  | | --- | | ugcugucuugacuuggcucuga | | |  | | --- | | - | | ugcugucuugacuuggcucugagguuuccacguucuccucagaaucagugcgaggcauccug || oni-mir-10548c | 36 | 36 | 0 | 0 | - | blast | |  | | --- | | acggcaccgcagaccacgccc | | |  | | --- | | - | | cguggucuguggagccgugcagguuuuguccgcccucccugcacggcaccgcagaccacgccc || oni-mir-10751 | 52 | 33 | 0 | 19 | - | blast | |  | | --- | | aagugcacgacuccagacuagu | | |  | | --- | | - | | agaguucgguguugugcgcucacgcucaaaaccuccacuggaagugcacgacuccagacuagu || abu-mir-10551 | 68 | 30 | 0 | 38 | - | blast | |  | | --- | | aggaaaucugcuucuguugguuu | | |  | | --- | | - | | aggaaaucugcuucuguugguuugguauuacuaaaacaucacaggagcagaguuuuucc || oni-mir-184c | 35 | 30 | 0 | 5 | - | blast | |  | | --- | | ugggcuggaaaagugauaaggag | | |  | | --- | | - | | ccuuaucaguucuccguccaacggauacagaaucuauagcugggcuggaaaagugauaaggag || oni-mir-10658 | 45 | 30 | 0 | 15 | - | blast | |  | | --- | | augagaaugcugacagggagcu | | |  | | --- | | - | | augagaaugcugacagggagcuauggcuuuagcaaugcugccugucacucuucugucu || oni-mir-10906 | 29 | 29 | 0 | 0 | - | blast | |  | | --- | | agcucggucuguguacaagacg | | |  | | --- | | - | | agcucggucuguguacaagacgcuuucugccauugugcuuuuacacggaacagcugc || abu-mir-16c | 27 | 26 | 0 | 1 | - | blast | |  | | --- | | aagcagcacauaacauacuggua | | |  | | --- | | - | | aagcagcacauaacauacugguaaugcagugaaaaauaccaguaaugaugugcugcuacg || oni-mir-10960 | 26 | 25 | 0 | 1 | - | blast | |  | | --- | | ucacuuggacacucuucuuggc | | |  | | --- | | - | | cugguagaauggcccaugauggagagcuauaaauagcuucacuuggacacucuucuuggc || oni-mir-10703 | 28 | 25 | 0 | 3 | - | blast | |  | | --- | | ucucuaccggcgcacgucgcugu | | |  | | --- | | - | | gcgacugcgugcggcagagugguuccgaaacaccgacuucucuaccggcgcacgucgcugu || oni-mir-26d | 23 | 22 | 0 | 1 | - | blast | |  | | --- | | ccuauccuggauuacuugaacc | | |  | | --- | | - | | gcaaguaaucaagcauaggccaaagcagaugcagaaagccuauccuggauuacuugaacc || oni-mir-10651 | 45 | 22 | 0 | 23 | - | blast | |  | | --- | | ugagcagggcuguguuuuugagc | | |  | | --- | | - | | uuaaaaacaggcccuggugacacaggagcugcggugcagcggugugagcagggcuguguuuuugagc || oni-mir-10655 | 25 | 20 | 0 | 5 | - | blast | |  | | --- | | uuccaccucuguguccucugcagu | | |  | | --- | | - | | uugcagugcacggagaggauugaguguuucugauuuccaccucuguguccucugcagu || oni-mir-10943 | 27 | 17 | 0 | 10 | - | blast | |  | | --- | | uuucaaauguucccucugguguu | | |  | | --- | | - | | acgcggggugccauuugauauucaugaggugucauccuguuucaaauguucccucugguguu || oni-mir-27f | 24 | 17 | 0 | 7 | - | blast | |  | | --- | | uucacauguggguuaaguccug | | |  | | --- | | - | | agaacuuaaccacugugaacauggcagacacucacuguucacauguggguuaaguccug || oni-mir-10690 | 25 | 16 | 0 | 9 | - | blast | |  | | --- | | cgugacuugaguaugggcaugca | | |  | | --- | | - | | cgugacuugaguaugggcaugcaguuuggaaagaaaaugcauccacaucucaagcuauuga || oni-mir-10638 | 15 | 15 | 0 | 0 | - | blast | |  | | --- | | cauaugugcucugcagcugacu | | |  | | --- | | - | | cauaugugcucugcagcugacuucuaaauaucccagcagcugcacugcaugcguggcu || oni-mir-10741 | 16 | 15 | 0 | 1 | - | blast | |  | | --- | | agaaagggucguuguccugcu | | |  | | --- | | - | | agaaagggucguuguccugcuugacuuaaaacccugucaggcagguuuuccucccuuuuacagag || oni-mir-10723 | 16 | 15 | 0 | 1 | - | blast | |  | | --- | | aggugacaugugcuuaggacaggu | | |  | | --- | | - | | cuguucugagucaaguccccuuguaugaacacaaacaaggugacaugugcuuaggacaggu || abu-mir-10562 | 69 | 14 | 0 | 55 | - | blast | |  | | --- | | ucaucagccagugagcgugugg | | |  | | --- | | - | | caugcucagugguuaacgacuugucaaucgccagucaucagccagugagcgugugg || oni-mir-10696 | 13 | 13 | 0 | 0 | - | blast | |  | | --- | | auggagugaacgucucagugggu | | |  | | --- | | - | | auggagugaacgucucaguggguaucagagcugauuuaagugguaaacaccugcugaacguuuccucuuccaga || oni-mir-4585 | 16 | 13 | 0 | 3 | - | blast | |  | | --- | | aaugcguuucggcuuguggccu | | |  | | --- | | - | | aaugcguuucggcuuguggccuucauuggacccugaugaaggccacaagccaaaacgcguugg || oni-mir-10803 | 13 | 13 | 0 | 0 | - | blast | |  | | --- | | acacucaguggacuuuuggacgu | | |  | | --- | | - | | acacucaguggacuuuuggacguucuaggaacauuuaggagacguucccuuuccacagagugucu || oni-mir-10663 | 13 | 12 | 0 | 1 | - | blast | |  | | --- | | uucagacggucauguagcgacu | | |  | | --- | | - | | uucagacggucauguagcgacucucacgguagaccagccgccugcagauuguucuguugc || abu-mir-10543 | 32 | 12 | 0 | 20 | - | blast | |  | | --- | | auuggcucuguagaggacugga | | |  | | --- | | - | | uuuguccucgcuuugaucuagugauuauuaccaguucauuggcucuguagaggacugga || oni-mir-10767 | 16 | 12 | 0 | 4 | - | blast | |  | | --- | | aggggaaacuuuuacuuugaag | | |  | | --- | | - | | ucagaguaaaaguuuugccuuacuguugcaaucaacagguuucaaaaggggaaacuuuuacuuugaag || oni-mir-204b | 22 | 12 | 0 | 10 | - | blast | |  | | --- | | cauaggaugacaaagggaagcc | | |  | | --- | | - | | aucccuuugcugucccugccuuauccaaguuccaggcauaggaugacaaagggaagcc || oni-mir-10953 | 14 | 11 | 0 | 3 | - | blast | |  | | --- | | aucacuucaggggacugaacc | | |  | | --- | | - | | aucacuucaggggacugaaccacugacaauuuguggcucauucuguugaugugcuuu || oni-mir-10649 | 24 | 11 | 0 | 13 | - | blast | |  | | --- | | uuuuaggucaggagaugcacacg | | |  | | --- | | - | | uguucgucuccuccugaaggcccgcaacacuacuuuuaggucaggagaugcacacg || oni-mir-3553-1 | 14 | 10 | 0 | 4 | - | blast | |  | | --- | | auggcccuaugguaauucacug | | |  | | --- | | - | | auggcccuaugguaauucacugauagugugcugugacagugaauucuaccagugccauac || oni-mir-10961 | 54 | 10 | 0 | 44 | - | blast | |  | | --- | | caccuuuaaguagaaauugacc | | |  | | --- | | - | | ucuuugucuacuucagguguuuuuguuuuucuuuuagcaccuuuaaguagaaauugacc || oni-mir-10913 | 12 | 10 | 0 | 2 | - | blast | |  | | --- | | uagcccgggcugcucugcuga | | |  | | --- | | - | | agcuguagcugugggccggggggugagcuaccuagcccgggcugcucugcuga || oni-mir-10581c | 64 | 10 | 0 | 54 | - | blast | |  | | --- | | aacguccagaugaacaaucaac | | |  | | --- | | - | | ugauuguucaucuggacguaacguuuucagugggagaaacguccagaugaacaaucaac || oni-mir-10697b-1 | 66 | 10 | 0 | 56 | - | blast | |  | | --- | | ggcggccucagaaucucaucu | | |  | | --- | | - | | ggcggccucagaaucucaucucugcuuuucuuagaugaugugguucuguuggcuuuagc || oni-mir-10937 | 10 | 10 | 0 | 0 | - | blast | |  | | --- | | uaacagaucaggguaguccucc | | |  | | --- | | - | | uaacagaucaggguaguccucccucccaggccucuguuuuguuguuuugaggauuaucaucuucugucagu || oni-mir-10877 | 11 | 10 | 0 | 1 | - | blast | |  | | --- | | agcccugcacucugauuggcu | | |  | | --- | | - | | agcccugcacucugauuggcuggugaaacagucugucugcagccaaucagaagucguggguuuga || oni-mir-10956 | 43 | 8 | 0 | 35 | - | blast | |  | | --- | | uagcuguucucuaacaacuaggccu | | |  | | --- | | - | | uagcuguucucuaacaacuaggccugcuggccauggugucucacaguucag || oni-mir-10792 | 10 | 8 | 0 | 2 | - | blast | |  | | --- | | uuuguuggaucuuggggcagc | | |  | | --- | | - | | agccccaucguccaauaaauuagaauuuguuggaucuuggggcagc || oni-mir-430b | 10 | 8 | 0 | 2 | - | blast | |  | | --- | | ucaagugcuaauuguuguugua | | |  | | --- | | - | | acagcaacaugagcacuuacgugaauucugaaaacaucaagugcuaauuguuguugua || oni-mir-10806 | 8 | 8 | 0 | 0 | - | blast | |  | | --- | | ucacauucagaucuggcaugacu | | |  | | --- | | - | | ucacauucagaucuggcaugacuuucugucuucucacagucauguuuagcuguguggugaga || oni-mir-10865 | 19 | 8 | 0 | 11 | - | blast | |  | | --- | | uucggauguagcucugccgcugcuc | | |  | | --- | | - | | uucggauguagcucugccgcugcucuaggugacucagcacagcguggagggcuguggcgaugg || oni-mir-10855 | 7 | 7 | 0 | 0 | - | blast | |  | | --- | | uucggguucagguucuccggcu | | |  | | --- | | - | | ugucggagccuccgggccugggacggacuccucaugguccgguuucggguucagguucuccggcu || oni-mir-10801 | 14 | 7 | 0 | 7 | - | blast | |  | | --- | | auggacguaccugacagcaga | | |  | | --- | | - | | auggacguaccugacagcagagcagcucgacucugcugucagguacgucacc || oni-mir-10974 | 7 | 7 | 0 | 0 | - | blast | |  | | --- | | ucugauuucugugucucuguca | | |  | | --- | | - | | acggaggugcagaacagcuucgggugacagcgcaucugauuucugugucucuguca || oni-mir-10765 | 19 | 7 | 0 | 12 | - | blast | |  | | --- | | aaugaaagcuguuugcuguaau | | |  | | --- | | - | | uugugcaaacagcuuugaucagagucugaugaaaccuaaugaaagcuguuugcuguaau || oni-mir-10863 | 10 | 6 | 0 | 4 | - | blast | |  | | --- | | ugacuucuuugugggcaggcac | | |  | | --- | | - | | ugacuucuuugugggcaggcaccaccaugcaugagccugcccacgcaguagucaug || oni-mir-10828 | 6 | 6 | 0 | 0 | - | blast | |  | | --- | | aagucaucacagaacuacacug | | |  | | --- | | - | | aagucaucacagaacuacacuguagugaaugggaggccaggguaguucugugaugacuugu || oni-mir-10576 | 10 | 6 | 0 | 4 | - | blast | |  | | --- | | uguagagcagacuggauucucu | | |  | | --- | | - | | uguagagcagacuggauucucugucucccuuuuuagaacggaggaucuuuuuugcucugcagu || oni-mir-726b | 17 | 5 | 0 | 12 | - | blast | |  | | --- | | ugaguucugcuaguagugaacu | | |  | | --- | | - | | ugaguucugcuaguagugaacuuuugacaaacacgaauaguucagaacuagcggaauuccaga || oni-mir-10548b | 5 | 5 | 0 | 0 | - | blast | |  | | --- | | cgugauugcggaugcugugcagg | | |  | | --- | | - | | cgugauugcggaugcugugcaggugcguccgccuucccugcacagcaccgcaggccacgccc || oni-mir-10864 | 7 | 5 | 0 | 2 | - | blast | |  | | --- | | uucuuagaucugaaagccuuacc | | |  | | --- | | - | | uaggguuuugagacagucuaagaacacagcugacaguguucuuagaucugaaagccuuacc || oni-mir-10876 | 5 | 4 | 0 | 1 | - | blast | |  | | --- | | cacaggcuguaauuccacugag | | |  | | --- | | - | | guggagcgacggccuguugcugcccguggauccugagugaagcacaggcuguaauuccacugag || oni-mir-10742 | 5 | 4 | 0 | 1 | - | blast | |  | | --- | | auucaggcugucaucuaucuuacc | | |  | | --- | | - | | ugagagauacuagccugacucauguggauguguucuugcauucaggcugucaucuaucuuacc || oni-mir-10620 | 6 | 4 | 0 | 2 | - | blast | |  | | --- | | gaaauaaagaauucucucgaagug | | |  | | --- | | - | | gaaauaaagaauucucucgaaguguuacuucuccaauaaucacucgaagaauuccuuguuuuag || oni-mir-10684 | 25 | 4 | 0 | 21 | - | blast | |  | | --- | | accauugaucaacaaccacugau | | |  | | --- | | - | | cagugagcauugaucaauggccaugcaaauucucauuaccauugaucaacaaccacugau || oni-mir-10957 | 4 | 4 | 0 | 0 | - | blast | |  | | --- | | aggggcagauuucugaucuggc | | |  | | --- | | - | | cauucaguucagagaucaugucugcaagagugcaggggcagauuucugaucuggc || oni-mir-26c | 8 | 4 | 0 | 4 | - | blast | |  | | --- | | gcaaguaaucacgaauaggcc | | |  | | --- | | - | | gcaaguaaucacgaauaggccauccaggauacacacagccuauccuggauuacuugaacu || oni-mir-10650 | 6 | 3 | 0 | 3 | - | blast | |  | | --- | | accccaugaugaaucucugaag | | |  | | --- | | - | | accccaugaugaaucucugaaggugaaucuccaucgagaggcuaauaucaaguucacuguggacgugcacc || oni-mir-10673 | 5 | 3 | 0 | 2 | - | blast | |  | | --- | | uuagaccagaugauaccagcccu | | |  | | --- | | - | | accgguauuguuggccuggguccugcuaagacacacgcuuagaccagaugauaccagcccu || oni-mir-10678 | 4 | 3 | 0 | 1 | - | blast | |  | | --- | | uucaaaguaaaagcugugccu | | |  | | --- | | - | | uucaaaguaaaagcugugccucaacacauuugaaaaggugcagcuguuacucugaagguga || oni-mir-10805 | 3 | 3 | 0 | 0 | - | blast | |  | | --- | | aguuucuggcugaaggagcucc | | |  | | --- | | - | | aguuucuggcugaaggagcuccaagcuccaucaacuuggacucucacagcuacgaacgcacc || oni-mir-10787 | 8 | 3 | 0 | 5 | - | blast | |  | | --- | | cucccacacuuacuuagcaccu | | |  | | --- | | - | | cucccacacuuacuuagcaccugugcagcauuacuaaaagggugcagugggugugggagug || oni-mir-10972 | 3 | 3 | 0 | 0 | - | blast | |  | | --- | | uuugcuauggucaucacuggag | | |  | | --- | | - | | ccaguuuagcccuaguguaauccauaucuuugcagagauuugcuauggucaucacuggag || oni-mir-10548a-1 | 3 | 3 | 0 | 0 | - | blast | |  | | --- | | augauuguggaugccgugcagg | | |  | | --- | | - | | augauuguggaugccgugcaggugcguccgcauucccugcaccacaccgcaggccacgc || oni-mir-10922 | 10 | 3 | 0 | 7 | - | blast | |  | | --- | | ugugcagcauagaggaacccaga | | |  | | --- | | - | | ugugcagcauagaggaacccagaccucagcuuugucucccgguuucuguugcugcuguugca || oni-mir-10808 | 4 | 2 | 0 | 2 | - | blast | |  | | --- | | acggaaagccgugcggccgggcu | | |  | | --- | | - | | cggcccgccgggucgccaucccggugcuggugcgggacggaaagccgugcggccgggcu || oni-mir-10889 | 3 | 2 | 0 | 1 | - | blast | |  | | --- | | cucagugagcuggaucagagcc | | |  | | --- | | - | | agcugaucucuuguuuucauugaagcuguuuccucucagcagcucagugagcuggaucagagcc || oni-mir-10633 | 15 | 2 | 0 | 13 | - | blast | |  | | --- | | uggcauugugggauagaagcuc | | |  | | --- | | - | | uggcauugugggauagaagcucaguaucagcugguucugugacugucucucucuaucagcuacagugccug || oni-mir-301c | 28 | 2 | 0 | 26 | - | blast | |  | | --- | | uagugcaacauugucaaagcag | | |  | | --- | | - | | uaugacaauacuauugcacugcuagaauggaugguacaguagugcaacauugucaaagcag || oni-mir-10941 | 4 | 2 | 0 | 2 | - | blast | |  | | --- | | ucacacaaaucacucgucucacu | | |  | | --- | | - | | agagaaaaggugaggggggaaguguuuagagccgaucugaaucgacuuuaugucacgcuugcucacacaaaucacucgucucacu || oni-mir-10835 | 2 | 2 | 0 | 0 | - | blast | |  | | --- | | uuccuuccucucucagucucug | | |  | | --- | | - | | uuccuuccucucucagucucugccagucauccucagaagcugagaaggaggugggcacc || oni-mir-10778 | 2 | 2 | 0 | 0 | - | blast | |  | | --- | | caacugcagggcauuggacgagc | | |  | | --- | | - | | cccucuaaagcccugcagcucaggguccuuucccaacugcagggcauuggacgagc || oni-mir-10752 | 16 | 2 | 0 | 14 | - | blast | |  | | --- | | caggacacagaaucgcuggugg | | |  | | --- | | - | | caggacacagaaucgcugguggcugacuuuacaccccccggcgugccuguccacugucu || oni-mir-10905-1 | 2 | 2 | 0 | 0 | - | blast | |  | | --- | | cuggcuagcauccugagccccgu | | |  | | --- | | - | | gaggccaggaggcagugccaagcagaugaugcagagcuggcuagcauccugagccccgu || oni-mir-10862 | 2 | 2 | 0 | 0 | - | blast | |  | | --- | | uguccucugauuggcucgga | | |  | | --- | | - | | uguccucugauuggcucggacaacccccucugacaugcccguggccaaucagaggugagu || oni-mir-10647 | 3 | 1 | 0 | 2 | - | blast | |  | | --- | | uuguuguugcugcugugaugcu | | |  | | --- | | - | | uuguuguugcugcugugaugcugaggacagacacagcaucacaguacuggaacagagcu || oni-mir-10768 | 589 | 1 | 0 | 588 | - | blast | |  | | --- | | ucacacagguuggcgaagguuu | | |  | | --- | | - | | accuuugcccaccugucugagagugauugcagugagucucacacagguuggcgaagguuu || oni-mir-6960 | 1 | 1 | 0 | 0 | - | blast | |  | | --- | | aaugaucaggaugagaugguuuggc | | |  | | --- | | - | | aaugaucaggaugagaugguuuggcuuuuuuuccacgccguccacucuccuguucccucu || oni-mir-10785 | 2 | 1 | 0 | 1 | - | blast | |  | | --- | | auagacggagccaggacacggu | | |  | | --- | | - | | cguguccucgcucauuuuguugacaauuacggcugaucauagacggagccaggacacggu || oni-mir-10573c | 586 | 1 | 0 | 585 | - | blast | |  | | --- | | aacuccaguccucgaaggccgg | | |  | | --- | | - | | aacuccaguccucgaaggccggugucaugcaucuuuuccaugcccucgaggacuggcguuugaga || oni-mir-10860 | 1 | 1 | 0 | 0 | - | blast | |  | | --- | | ugggugugggcucugcucucuacc | | |  | | --- | | - | | cuggagaguugagcccaagcaggcagcucugagguagcugggugugggcucugcucucuacc || oni-mir-10951 | 3 | 1 | 0 | 2 | - | blast | |  | | --- | | agugcugccugacuucaugccu | | |  | | --- | | - | | agugcugccugacuucaugccuuucucuucuuucaauaagucaugcagcacaga || oni-mir-10754 | 3 | 1 | 0 | 2 | - | blast | |  | | --- | | uuagcuggucugaccuuccucgccu | | |  | | --- | | - | | gugggggaagugucugucuaguuuggauuguaaaaugugaacaauuagcuggucugaccuuccucgccu || oni-mir-10775 | 2 | 1 | 0 | 1 | - | blast | |  | | --- | | caggagcuccgauuggccaagu | | |  | | --- | | - | | caggagcuccgauuggccaagugucaacucaugugggagguaauuguguaacagcuggccaauccuagccccugcu || oni-mir-10734 | 3 | 1 | 0 | 2 | - | blast | |  | | --- | | ugcuuucuuuagaauuuauc | | |  | | --- | | - | | ugcuuucuuuagaauuuaucaugucauauaaaguugcuaaacucuucagggggcuuu || oni-mir-10774 | 1 | 1 | 0 | 0 | - | blast | |  | | --- | | uuuuggaucacuguuauuaagu | | |  | | --- | | - | | uuuuggaucacuguuauuaaguggagucauuuaaaacaacugauccuucaga || oni-mir-10724 | 1 | 1 | 0 | 0 | - | blast | |  | | --- | | ucagugagcuguaucccgugaa | | |  | | --- | | - | | ucaggacugcagcucugcugguuuucgucuuuuaaaaaucagugagcuguaucccgugaa || oni-mir-10602 | 1 | 1 | 0 | 0 | - | blast | |  | | --- | | gccgagaacgcgagcacggacu | | |  | | --- | | - | | uacguacucgcguucucggugaguauguacuggccgagaacgcgagcacggacu || oni-mir-10968 | 1 | 1 | 0 | 0 | - | blast | |  | | --- | | agaacaaugaaagcagacgagg | | |  | | --- | | - | | gaggcugcuuugcugcuucuuuuaugccccaaaaagaacaaugaaagcagacgagg || oni-mir-10883 | 1 | 1 | 0 | 0 | - | blast | |  | | --- | | ccaccagcucucucuuggcac | | |  | | --- | | - | | gcuaagagagcucuguucggccagagcucaugaggccaccagcucucucuuggcac || oni-mir-10771 | 1 | 1 | 0 | 0 | - | blast | |  | | --- | | cuguuguucugaguggcuguga | | |  | | --- | | - | | ccugucacuuuaacaauauguuugauaauuacccuuuuacuguuguucugaguggcuguga || oni-mir-10709 | 3 | 1 | 0 | 2 | - | blast | |  | | --- | | uccacagcagcacuucugcacacu | | |  | | --- | | - | | gauguagagggcugacuguggacagagaugaguaugguuguccacagcagcacuucugcacacu || oni-mir-10939-1 | 1 | 1 | 0 | 0 | - | blast | |  | | --- | | uauguggacauaaggcuuaccc | | |  | | --- | | - | | uauguggacauaaggcuuacccaucuuuaccagcacaauggggaggccuaugugcacucgug || oni-mir-10674 | 4 | 1 | 0 | 3 | - | blast | |  | | --- | | aauagccgccuugagaccagga | | |  | | --- | | - | | aauagccgccuugagaccaggagggcaagguucgaaaccugcucagggcggcauucuuuu || oni-mir-10892 | 2 | 0 | 0 | 2 | - | blast | |  | | --- | | ugaauaauggaugaauggcaca | | |  | | --- | | - | | ucuuuucauccuuuauucucccacuugaucuccacguggcaggccuuuggaagcagggugaauaauggaugaauggcaca || oni-mir-10598 | 2 | 0 | 0 | 2 | - | blast | |  | | --- | | uggaccgaacaaugcauccugca | | |  | | --- | | - | | caggaugaacugugaguggcauuuuugucaggacaaauggaccgaacaaugcauccugca || oni-mir-144b | 3 | 0 | 0 | 3 | - | blast | |  | | --- | | uuacaguauaagaugauauccug | | |  | | --- | | - | | aguacaucaucuauacuguagugucucuuuaauaaacuuacaguauaagaugauauccug || oni-mir-10710 | 30 | 0 | 0 | 30 | - | blast | |  | | --- | | acgccaugacgcugacaucaggac | | |  | | --- | | - | | ccgugaugucagcaccguggagguggucacugagacggagcgacgccaugacgcugacaucaggac || oni-mir-10809 | 1 | 0 | 0 | 1 | - | blast | |  | | --- | | auagacuucccaucgucacagu | | |  | | --- | | - | | auagacuucccaucgucacaguccgaugggcagauugcgacaguggggcgccu || nbr-mir-10569 | 2 | 0 | 0 | 2 | - | blast | |  | | --- | | accgagcagaacucugacgagc | | |  | | --- | | - | | ucauuggaguucugcucggcgcuggucugaaggcgggaccgagcagaacucugacgagc || oni-mir-10857 | 2 | 0 | 0 | 2 | - | blast | |  | | --- | | cugcacugacgagcuuucaccc | | |  | | --- | | - | | augacggcuugaccgugcagcuucaaaacacugaagcugcacugacgagcuuucaccc || fru-mir-10b-2 | 4177306 | 0 | 0 | 4177306 | - | blast | |  | | --- | | caaguacgucucuacaggaaua | | |  | | --- | | - | | guugucuauauguacccuguagaaccgaauuugugugaguuccagacagucgcaaguacgucucuacaggaauacaugggcaac || oni-mir-10896 | 5 | 0 | 0 | 5 | - | blast | |  | | --- | | cuggacggcgcuaaugcguu | | |  | | --- | | - | | cgaguuagcguggaucgccccguacguggggcuggacggcgcuaaugcguu || oni-mir-10786 | 1 | 0 | 0 | 1 | - | blast | |  | | --- | | uaguccacgagcagucugugac | | |  | | --- | | - | | uaguccacgagcagucugugacugcuugcacugagucacagucuguucguggacagc || oni-mir-10745 | 1 | 0 | 0 | 1 | - | blast | |  | | --- | | uuuucaguuucccucucucagg | | |  | | --- | | - | | uuuucaguuucccucucucaggcaauuaucaauccugagagagagaagcugaaaagag || oni-mir-10829 | 1 | 0 | 0 | 1 | - | blast | |  | | --- | | uaggcccgugcagcgcggucgga | | |  | | --- | | - | | ugcugcgcgcaucaggggcuggaguggaguggauagcuucuaggcccgugcagcgcggucgga || oni-mir-10925 | 1 | 0 | 0 | 1 | - | blast | |  | | --- | | augcacugagagauuauguucu | | |  | | --- | | - | | agaacauaaucucucaguugcauguguuuccugcaugcaugcacugagagauuauguucu || oni-mir-10761 | 3 | 0 | 0 | 3 | - | blast | |  | | --- | | uugaauuuguucuuguugaauc | | |  | | --- | | - | | uugaauuuguucuuguugaaucuugagacuuuaaauuuaauucaugguuaacuuuagaagaugcaacaggaucaaauuugaaa || ccr-mir-218a | 44259 | 0 | 0 | 44259 | - | blast | |  | | --- | | acaugguuccgucaagcaccagg | | |  | | --- | | - | | ggugcagcugucucuugugcuugaucuaaccaugugccgccgccuacacaagccucacaugguuccgucaagcaccagggaccgcuggac || oni-mir-219d-2 | 1 | 0 | 0 | 1 | - | blast | |  | | --- | | agaauugcguuuggacaaucagc | | |  | | --- | | - | | agauguccaggcacaauucucggguuggauuugaaauuucacaagaauugcguuuggacaaucagc || abu-mir-729 | 13 | 0 | 0 | 13 | - | blast | |  | | --- | | ggggcuguaucauaaccaggcu | | |  | | --- | | - | | ggggcuguaucauaaccaggcugugagcugcauggagcauggguaugauacgaccuca || oni-mir-10927 | 2 | 0 | 0 | 2 | - | blast | |  | | --- | | aucaucaacucugucacguga | | |  | | --- | | - | | acgguacacagagcugguguuuguuguaguuuaaaacagcaucaucaacucugucacguga || oni-mir-10816 | 3 | 0 | 0 | 3 | - | blast | |  | | --- | | uuaauguccaucugucacugu | | |  | | --- | | - | | ccguugacagagugaagacgcuaauuaaaacacugaaaauuaauguccaucugucacugu || oni-mir-10760 | 1 | 0 | 0 | 1 | - | blast | |  | | --- | | uuaguuaucuuggcuggagcug | | |  | | --- | | - | | uuaguuaucuuggcuggagcuguuuaguucagcuccagccaagauaacuga || oni-mir-10910 | 1 | 0 | 0 | 1 | - | blast | |  | | --- | | accuccuccacugugggaaccu | | |  | | --- | | - | | accuccuccacugugggaaccuuguucccugcagccugcaaggcucuccacagggcagagucu || oni-mir-10907 | 1 | 0 | 0 | 1 | - | blast | |  | | --- | | ugccuggcauuucucuguucacu | | |  | | --- | | - | | ugccuggcauuucucuguucacuccguuuucuuagugcagauaaguguccagguuuc || oni-mir-10706 | 2 | 0 | 0 | 2 | - | blast | |  | | --- | | ugagggcugcauggugcuaacg | | |  | | --- | | - | | ugagggcugcauggugcuaacgccuuaacaagcuaaccgcguugacaccgugcagcccuugcu || oni-mir-10875 | 2 | 0 | 0 | 2 | - | blast | |  | | --- | | auguacccccuuguuccugac | | |  | | --- | | - | | auguacccccuuguuccugacaagguuuuugucagucuggaacaaggguguauauca || oni-mir-10735 | 1 | 0 | 0 | 1 | - | blast | |  | | --- | | uguagagacacaucauuauauc | | |  | | --- | | - | | cguaaugagcugucuagcugcagucugcauggcuuuguagagacacaucauuauauc || oni-mir-10886 | 48 | 0 | 0 | 48 | - | blast | |  | | --- | | uugguaccagcugagcaucauu | | |  | | --- | | - | | ugguguggggcuguuaucagcacacuuugggccucuugguaccagcugagcaucauu || oni-mir-10866 | 2 | 0 | 0 | 2 | - | blast | |  | | --- | | gugagugaaaaaggugauaauu | | |  | | --- | | - | | gugagugaaaaaggugauaauuacaacuuugacaauuauaucacuuuugcaauuuugauaaauuaucuguuuuuuugcucauag || oni-mir-10946 | 1 | 0 | 0 | 1 | - | blast | |  | | --- | | acugaauuuggcaggcugauccu | | |  | | --- | | - | | gugccugcuggauuuaaucaaauaacacuaaacauugacugaauuuggcaggcugauccu || oni-mir-10726 | 1 | 0 | 0 | 1 | - | blast | |  | | --- | | ccugggacuguuguggaggccc | | |  | | --- | | - | | ccugggacuguuguggaggcccuuucuaauaagguugcggcccuccaugacagucccaacuc || oni-mir-10970 | 3 | 0 | 0 | 3 | - | blast | |  | | --- | | uuccuggaaucauuucgacuc | | |  | | --- | | - | | gucgaaaugauuccaggaacagaaugaaguccaacuucauucuguuccuggaaucauuucgacuc |
