# Supplementary material for: Differential expression of miRNAs in the presence of B chromosome in the cichlid fish Astatotilapia latifasciata
Source: BMC Genomics. 2021 May 12;22:344. doi: 10.1186/s12864-021-07651-w (PMC8117508; doi:10.1186/s12864-021-07651-w)
Supplement: Supplementary file 4 — Additional file 4. Nucleotide sequences (Figure S1) and reads coverage (Figure S2) of pre-miRNAs sequences on the B+ assembled genome. Figure S1. Sequence descriptions: #primer>NODE_contigXXXX Mzebra scaffold_XX start:end B−/B+ reads. The primer binding sites are highlighted in bold. Figure S2. Genome assembly bias. The red rectangles on the graphs highlight the pre-miRNAs regions in the M. zebra genome and A. latifascita B− and B+ genomic reads datasets. These miRNAs were predicted in the B+ assembly using the reads that failed to align in the B− reference assembly expecting to find some B+ chromosome miRNA. [file 12864_2021_7651_MOESM4_ESM.pdf]

Nucleotide sequences (Figure S1) and reads coverage (Figure S2) of pre-miRNAs sequences on the B^+ assembled genome.

1>NODE_100054_length_260_cov_13.534616:1-255 - Mzebra scaffold_35 - 2593821-2593795 B+
ATGACCGTTAATATCTGGGTGACGGCTTAGGTTTCGCTTTTGCTTTGGACAAGAGGCCTTGGAATTGTATTTTTTTTATTTATCCACCAGGTGGCGCCCTTGCTCCAAATTTGATCCGGGGCTCG
AAAACAGCCGAAAACAGCGGGCTGCTTAGAAAGACCTGCGGGGAATGAAGCCTCAATTTCGGCGTGCAGCAGCAGCTTCGCCCGCTGATCGGGCGGGA**CCATTTTCGGCCACTAGGA**GCTT
TTGAGGGT

2>NODE_101487_length_1287_cov_10.790987:793-944 – Mzebra scaffold_440 98766-98750 B+
GGGCTA**GCTCCCTTTGATAGCTCAGC**TGGTAGAGCAGAGGACCACAACACGGATGCAACGTCATATCCTTaggtagtggaTTGGGTTGACGTCGTATCCGTGGCTATCCTCAGTCGCTGGTTCAAG
TC**CAGCTTGAAGGGAGATGTTAGTG**

3>NODE_301244_length_3618_cov_31.396351:2388-2560 – Mzebra scaffold_272 112777-112696 B+
CCG**GTATCGCTTCTCGGCCTTTT**GGCTAAGATCAAGTGTAGTATCTGTTCTTATCAGTTTAATATCTGATACGTCCCCTACCCGGGGACCatatattaaattgattttggaacagggagaTGGAATAGGGGCTTG
CTCCGTCCACTCC**ACGCATCGACCTGGTATTG**CAGT

4>NODE_930998_length_49431_cov_28.273998:36203-36369 – Mzebra scaffold_25 3400510-3400445 B+
AT**GGGAGCAATGTCACTTTTGTCT**CCCAGCACCAGCCAAACTAGGGGAAAATAATTCTGGTATGTATAAGCAGAAACAAAACAGTTACTGGCACACAATCACAGCTCACTGCATACAACCTCAAA
TTATTTAATTACCGGGCCCCGGGTGAGTATATATTTAAGCCATCATTATCTACCGAGTGCAATTGTTGTTAGGAGAAA**CTGCTGCGGCTCATAAAAATG**GTATTTAAAAGAATAGTCGTAATTCTACC
TTC

5>NODE_130674_length_183_cov_7716.606445:90-245 – Mzebra scaffold_26 1703851-1703905 B-
A**ATCAGGTGGGTGGTTGCTTTAA**TTTATTTGACTAAGCACCATAATCTTAGATAACTATCTTTGGGTGCAAAATTTGAAAAGGCTCAAATGTATGCAGCATTGGAGAGTAACAGAATACATGTACTG
CCGGATGCC**GCATGTAGTCCTGATGAGCC**GGCCCGGGTTCGAGTATGACCCGTACTTTAATTTTTAATTTTTTTTT

6>NODE_35549_length_678_cov_13.758112:33-199 – Mzebra scaffold_103 1015240-1015177 B-
GCTCGCATTTCTGTTTTTTTTAACAGAGGCAGCCCAGTTACTGTTTTTAAACTGTTCTTGTTTTTTTTT**CCAATCCCTCCTTTAGGTTCAGG**CTGCCCAGGCATCCACTGCATGTCTTCCTGTGCA
GGTG**TTTGGGGCCTGACAggaa**gggcatggtttggtttttgGCTTTGTCATTTCTGCAAATGAAAATGGAACATGTAGGGATTATTTCAGGTCTGTTTTGGTGAAACGATTACAAAAAGAATT

7>NODE_319948_length_10366_cov_33.443951:1946-2097 – Mzebra scaffold_94 – 1629-1679 B-
readsTGTGAAGTACTCTGACTGCCAGTCAGTCTGAGCAGGGTACAGGGTATAGTAGGTAGGGAATAGCGCTCAAACACAGGCTGTGTTTGAGAG**CTATACCCAGGCTACATTTCAAG**TTTCATT
CATTACTTAATAGTTACACTCTTTGACCAGT

8>NODE_80652_length_7592_cov_20.613541:1132-1289 – Mzebra scaffold_210 508222-608166 B-/B+
GGTCTTCTCACCCAGTCTCCCTTCCTGTGGACGATGCCGATCTCCGCTGAGCCAGACCGCAGCCGGAGCATCACCCATGGAGCACGACGCTCCGGCTGCGGTCCGACTCGCCGGAGACGT
GTCTGCTTCTGCATAAA**GTTTCCACCGAGGTTGCTGC**

9>NODE_120347_length_9887_cov_27.215435:4975-5136 – Mzebra scaffold_188 875231-875290 B-/B+
TTTTTTTTTT**CCTCCTTGCTGCTGTGTTG**GCAGCTCAGGGCAGCGTTCCTGAACCAAGTCATCCGGAATAGGCTGTGCTTGTCTGAACAAACCTATCCTGGATTACTTGAATCAGGCCCAGACC
CATCGTGTGTCATCGGACTC**CAAGCAGCCGTCCTCAA**

10>NODE_313069_length_5831_cov_30.092779:266-423 – Mzebra scaffold_77 2572262-2572318 B-/B+
A**ATCAGAAGGTCGGTGGTTCG**ATCCCAGGCTGCCTCCTGGCTGCCAAATATCCACACTGGCAAGCCACAATGTAGCTTCAAACTACAATGTAGCTTGCCAGTGTGAATATGTGTGTGTATGACT
GAATGTAGTGTGAAGC**GCTTTGGGGTCCTTAGG**

Figure S1 – Sequence descriptions: #primer>NODE_contigXXXX Mzebra scaffold_XX start:end B^-/B^+ reads. The primer binding sites are highlighted in bold.

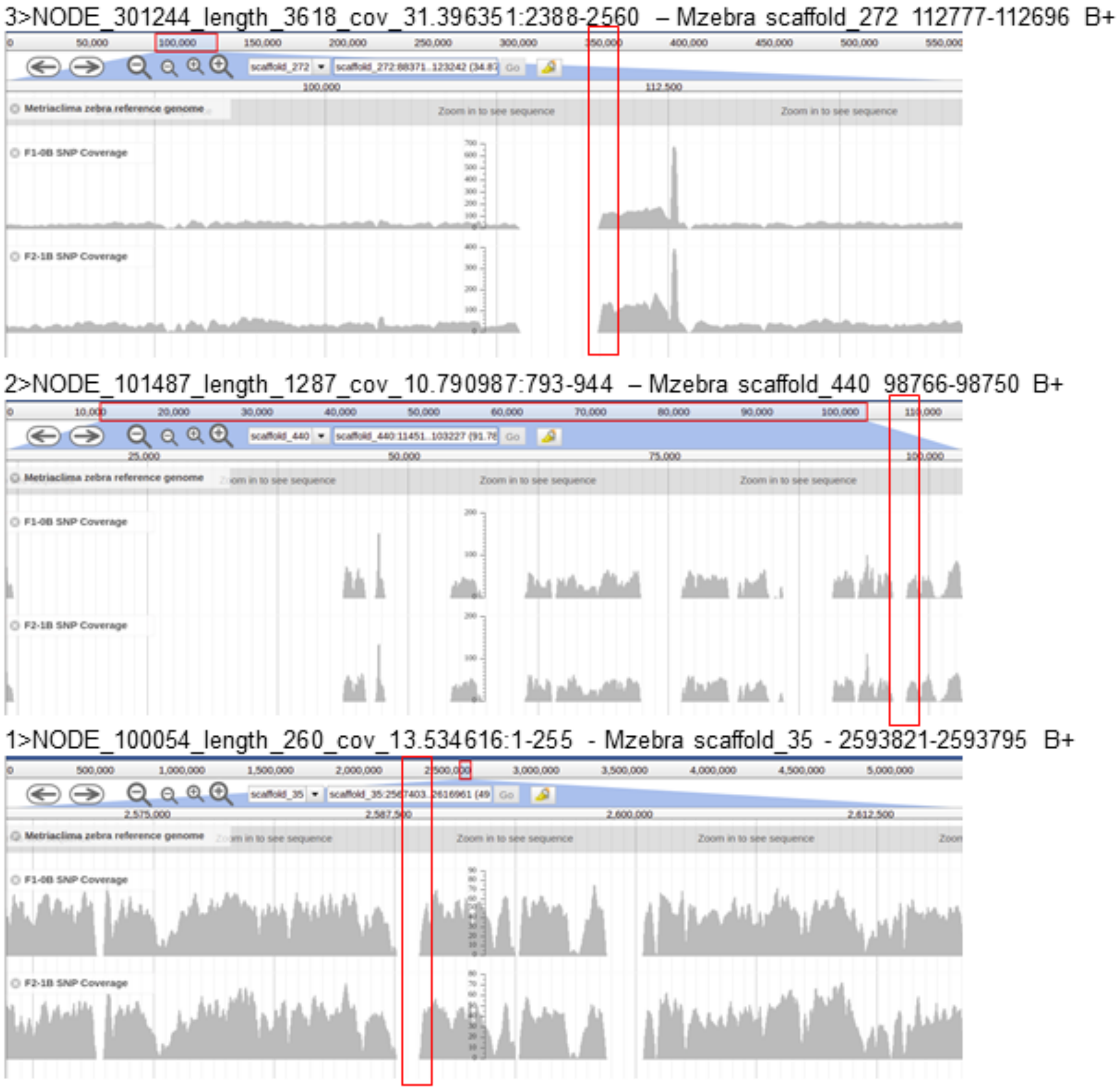

Figure S2 – Genome assembly bias. The red rectangles on the graphs highlight the pre-miRNAs regions in the *M. zebra* genome and *A. latifascita* B^- and B^+ genomic reads datasets. These miRNAs were predicted in the B^+ assembly using the reads that failed to align in the B^- reference assembly expecting to find some B^+ chromosome miRNA.
