# Supplementary material for: Differential expression of miRNAs in the presence of B chromosome in the cichlid fish Astatotilapia latifasciata
Source: BMC Genomics. 2021 May 12;22:344. doi: 10.1186/s12864-021-07651-w (PMC8117508; doi:10.1186/s12864-021-07651-w)
Supplement: Supplementary file 5 — Additional file 5. Expression of drosha and dicer genes. [file 12864_2021_7651_MOESM5_ESM.pdf]

Additional File 5 - Expression of the drosha and dicer genes.

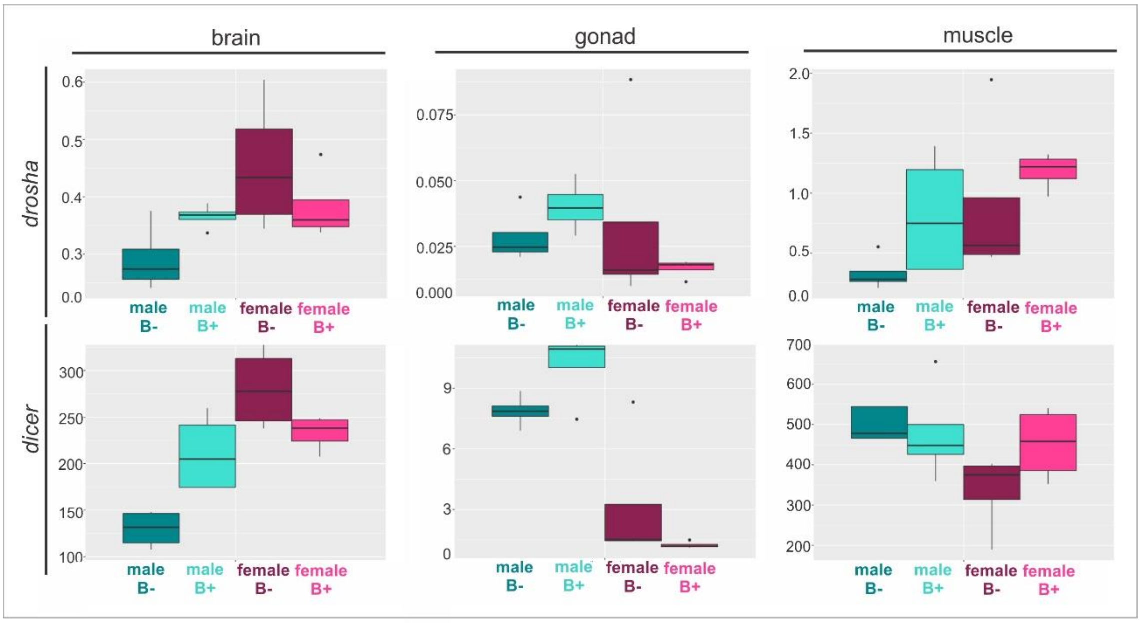

The Y axis represents the expression based on the ΔΔCq method, and the X axis represents the samples (B-, sample without the B chromosome; B+, sample with the B chromosome). The bar within the box represents the median, the boxplot represents quartiles, the vertical line represents the minimum or maximum value, and points are outliers. No significant differences in expression were observed between groups.
