# Supplementary material for: Differential expression of miRNAs in the presence of B chromosome in the cichlid fish Astatotilapia latifasciata
Source: BMC Genomics. 2021 May 12;22:344. doi: 10.1186/s12864-021-07651-w (PMC8117508; doi:10.1186/s12864-021-07651-w)
Supplement: Supplementary file 6 — Additional file 6. Html files containing miRNA prediction in B-blocks, separated by RNA-seq sample. S1 miRDeep2 – miRNA prediction in B-blocks using brain female B− RNA-seq. S2 miRDeep2 – miRNA prediction in B-blocks using brain female B+ RNA-seq. S3 miRDeep2 – miRNA prediction in B-blocks using brain male B− RNA-seq. S4 miRDeep2 – miRNA prediction in B-blocks using brain male B+ RNA-seq. S5 miRDeep2 – miRNA prediction in B-blocks using gonads female B− RNA-seq. S6 miRDeep2 – miRNA prediction in B-blocks using gonads female B+ RNA-seq. S7 miRDeep2 – miRNA prediction in B-blocks using gonads male B− RNA-seq. S8 miRDeep2 – miRNA prediction in B-blocks using gonads male RNA-seq. S9 miRDeep2 – miRNA prediction in B-blocks using muscle female B− RNA-seq. S10 miRDeep2 – miRNA prediction in B-blocks using muscle female B+ RNA-seq. S11 miRDeep2 – miRNA prediction in B-blocks using muscle male B− RNA-seq. S12 miRDeep2 – miRNA prediction in B-blocks using muscle male B+ RNA-seq. [file 12864_2021_7651_MOESM6_ESM.zip › S5.html]

miRDeep2


#### B chromosome impacts cell physiology through the differential regulation of miRNAs in the cichlid fish *Astatotilapia latifasciata*, Nascimento-Oliveira, JIN et al.

| **miRDeep2 - miRNA prediction in B-blocks using gonads female B- RNA-seq** | . |

## Parameters used

|  |  |
| --- | --- |
| miRDeep2 version | 2.0.1.2 |
  
| Program call | /home/jordana/mirdeep2-master/bin/miRDeep2.pl /home/exchangestudents/nathalie/gonads\_female\_b-\_miRNA/mapper\_results/gonads\_female\_b-\_aligned.fasta /home/exchangestudents/nathalie/duplicated\_b\_blocks /home/exchangestudents/nathalie/gonads\_female\_b-\_miRNA/mapper\_results/gonads\_female\_b-\_aligned.arf /home/exchangestudents/nathalie/mature\_miRNA\_ala.fa /home/exchangestudents/nathalie/mirbase\_reference\_mature\_miRNA.fa /home/exchangestudents/nathalie/mirbase\_reference\_precursor\_miRNA.fa /home/exchangestudents/nathalie/gonads\_female\_b-\_miRNA/mapper\_results/mapper\_logs/ |||  |  |
| --- | --- |
| Reads | /home/exchangestudents/nathalie/gonads\_female\_b-\_miRNA/mapper\_results/gonads\_female\_b-\_aligned.fasta |
| Genome | /home/exchangestudents/nathalie/duplicated\_b\_blocks |
| Mappings | /home/exchangestudents/nathalie/gonads\_female\_b-\_miRNA/mapper\_results/gonads\_female\_b-\_aligned.arf |
| Reference mature miRNAs | /home/exchangestudents/nathalie/mature\_miRNA\_ala.fa |
| Other mature miRNAs | /home/exchangestudents/nathalie/mirbase\_reference\_mature\_miRNA.fa |

  
  

**Survey of miRDeep2 performance for score cut-offs -10 to 10**


|  | novel miRNAs | | | known miRBase miRNAs | | |  |  |
| --- | --- | --- | --- | --- | --- | --- | --- | --- |
 miRDeep2 scorefor details on how the log-odds score is calculated, see Friedlander et al., Nature Biotechnology, 2008. | predicted by miRDeep2novel miRNA hairpins are here defined by not having any of the reference mature miRNAs mapping perfectly (full length, no mismatches). The numbers show how many novel miRNA hairpins have a score equal to or exceeding the cut-off. | estimated false positivesnumber of false positive miRNA hairpins predicted at this cut-off, as estimated by the miRDeep2 controls (see Friedlander et al., Nature Biotechnology, 2008). Mean and standard deviation is estimated from 100 rounds of permuted controls. | estimated true positivesthe number of true positive miRNA hairpins is estimated as t = total novel miRNAs - false positive novel miRNAs. The percentage of the predicted novel miRNAs that is estimated to be true positives is calculated as p = t / total novel miRNAs. The number of false positives is estimated from 100 rounds of permuted controls. In each of the 100 rounds, t and p are calculated, generating mean and standard deviation of t and p. The variable p can be used as an estimation of miRDeep2 positive predictive value at the score cut-off. | in speciesnumber of reference mature miRNAs for that species given as input to miRDeep2. | in datanumber of reference mature miRNAs for that species that map perfectly (full length, no mismatches) to one or more of precursor candidates that have been excised from the genome by miRDeep2. | detected by miRDeep2number of reference mature miRNAs for that species that map perfectly (full length, no mismatches) to one or more of predicted miRNA hairpins that have a score equal to or exceeding the cut-off. The percentage of reference mature miRNAs in data that is detected by miRDeep2 is calculated as s = reference mature miRNAs detected / reference mature miRNAs in data. s can be used as an estimation of miRDeep2 sensitivity at the score cut-off. | estimated signal-to-noisefor the given score cut-off, the signal-to-noise ratio is estimated as r = total miRNA hairpins reported / mean estimated false positive miRNA hairpins over 100 rounds of permuted controls. | excision gearingthis is the minimum read stack height required for excising a potential miRNA precursor from the genome in this analysis. || 10 1 2 ± 1 0 ± 0 (18 ± 39%) 457 3 0 (0%) 0.6 1 | | | | | | | | |
| 9 3 2 ± 1 1 ± 1 (43 ± 38%) 457 3 0 (0%) 1.6 1 | | | | | | | | |
| 8 3 2 ± 1 1 ± 1 (42 ± 37%) 457 3 0 (0%) 1.5 1 | | | | | | | | |
| 7 3 2 ± 1 1 ± 1 (40 ± 37%) 457 3 0 (0%) 1.5 1 | | | | | | | | |
| 6 3 2 ± 1 1 ± 1 (39 ± 36%) 457 3 0 (0%) 1.4 1 | | | | | | | | |
| 5 3 2 ± 1 1 ± 1 (37 ± 35%) 457 3 0 (0%) 1.4 1 | | | | | | | | |
| 4 4 2 ± 1 2 ± 1 (45 ± 32%) 457 3 0 (0%) 1.7 1 | | | | | | | | |
| 3 4 3 ± 2 2 ± 1 (39 ± 33%) 457 3 0 (0%) 1.5 1 | | | | | | | | |
| 2 4 3 ± 2 1 ± 1 (32 ± 32%) 457 3 0 (0%) 1.3 1 | | | | | | | | |
| 1 5 4 ± 2 2 ± 1 (33 ± 29%) 457 3 0 (0%) 1.4 1 | | | | | | | | |
| 0 11 5 ± 2 6 ± 2 (54 ± 20%) 457 3 0 (0%) 2.1 1 | | | | | | | | |
| -1 13 9 ± 3 4 ± 3 (34 ± 19%) 457 3 0 (0%) 1.5 1 | | | | | | | | |
| -2 15 13 ± 3 3 ± 2 (17 ± 15%) 457 3 0 (0%) 1.1 1 | | | | | | | | |
| -3 19 18 ± 4 2 ± 2 (12 ± 13%) 457 3 0 (0%) 1.1 1 | | | | | | | | |
| -4 25 23 ± 4 3 ± 3 (11 ± 11%) 457 3 0 (0%) 1.1 1 | | | | | | | | |
| -5 25 29 ± 5 1 ± 1 (2 ± 5%) 457 3 0 (0%) 0.9 1 | | | | | | | | |
| -6 34 40 ± 6 1 ± 2 (2 ± 4%) 457 3 0 (0%) 0.8 1 | | | | | | | | |
| -7 37 53 ± 8 0 ± 0 (0 ± 0%) 457 3 0 (0%) 0.7 1 | | | | | | | | |
| -8 39 65 ± 8 0 ± 0 (0 ± 0%) 457 3 0 (0%) 0.6 1 | | | | | | | | |
| -9 43 77 ± 9 0 ± 0 (0 ± 0%) 457 3 0 (0%) 0.6 1 | | | | | | | | |
| -10 57 85 ± 9 0 ± 0 (0 ± 0%) 457 3 0 (0%) 0.7 1 | | | | | | | | |

  
  
  
  
  

## novel miRNAs predicted by miRDeep2

  


provisional idthis is a provisional miRNA name assigned by miRDeep2. The first part of the id designates the chromosome or genome contig on which the miRNA gene is located. The second part is a running number that is added to avoid identical ids. The running number is incremented by one for each potential miRNA precursor that is excised from the genome. Clicking this field will display a pdf of the structure, read signature and score breakdown of the reported miRNA. | miRDeep2 scorethe log-odds score assigned to the hairpin by miRDeep2 | estimated probability that the miRNA candidate is a true positivethe estimated probability that a predicted novel miRNA with a score of this or higher is a true positive. To see exactly how this probability is estimated, mouse over the 'novel miRNAs, true positives' in the table at the top of the webpage. | rfam alertthis field indicates if the predicted miRNA hairpin has sequence similarity to reference rRNAs or tRNAs. Warnings in this field should overrule the estimated probability that a reported miRNA is a true positive (previous field). | total read countthis is the sum of read counts for the predicted mature, loop and star miRNAs. | mature read countthis is the number of reads that map to the predicted miRNA hairpin and are contained in the sequence covered by the predicted mature miRNA, including 2 nts upstream and 5 nts downstream. | loop read countthis is the number of reads that map to the predicted miRNA hairpin and are contained in the sequence covered by the predicted miRNA loop, including 2 nts upstream and 5 nts downstream. | star read countthis is the number of reads that map to the predicted miRNA hairpin and are contained in the sequence covered by the predicted star miRNA, including 2 nts upstream and 5 nts downstream. | significant randfold p-valuethis field indicates if the estimated randfold p-value of the excised potential miRNA hairpin is equal to or lower than 0.05 (see Bonnet et al., Bioinformatics, 2004). | miRBase miRNAthis field displays the ids of any reference mature miRNAs for the species that map perfectly (full length, no mismatches) to the reported miRNA hairpin. If this is the case, the reported miRNA hairpin is assigned as a known miRNA. If not, it is assigned as a novel miRNA. If more than one reference mature miRNA maps to the miRNA hairpin, then only the id of the reference miRBase miRNA that matches the predicted mature sequence is output. | example miRBase miRNA with the same seedthis field displays the ids of any reference mature miRNAs from related species that have a seed sequence identical to that of the reported mature miRNA. The seed is here defined as nucleotides 2-8 from the 5' end of the mature miRNA. If more than one reference mature miRNA have identical seed, then only the id of the miRNA that occurs last in the input file of reference mature miRNAs from related species is displayed. | UCSC browserif a species name was input to miRDeep2, then clicking this field will initiate a UCSC blat search of the consensus precursor sequence against the reference genome. | NCBI blastnclicking this field will initiate a NCBI blastn search of the consensus precursor sequence against the nr/nt database (non-redundant collection of all NCBI nucleotide sequences). | consensus mature sequencethis is the consensus mature miRNA sequence as inferred from the deep sequencing reads. | consensus star sequencethis is the consensus star miRNA sequence as inferred from the deep sequencing reads. | consensus precursor sequencethis is the consensus precursor miRNA sequence as inferred from the deep sequencing reads. Note that this is the inferred Drosha hairpin product, and therefore does not include substantial flanking genomic sequence as does most miRBase precursors. | precursor coordinateThe given precursor coordinates refer do absolute position in the mapped reference sequence || NODE\_59087\_length\_382\_cov\_9.583770\_7967 | 1.7e+1 | 0.18 ± 0.39 |  | 47 | 40 | 5 | 2 | no |  |  |  | blast | uccaugaaaguucugugcuaac | uugguuuggagacucuggacgcuuuu | uccaugaaaguucugugcuaacccuuguucucuguaugcagauuucucguguugguuuggagacucuggacgcuuuu | NODE\_59087\_length\_382\_cov\_9.583770:331..408:+ |
| NODE\_599881\_length\_498\_cov\_12.104418\_9535 | 9.7 | 0.43 ± 0.38 |  | 37 | 31 | 0 | 6 | no |  |  |  | blast | uaacacagaaacucaccagcucggc | ucuggcagguaggaucuaugagc | uaacacagaaacucaccagcucggcuucaucagucauaacagcgauaucaaugaaaagaagaucuggcagguaggaucuaugagc | NODE\_599881\_length\_498\_cov\_12.104418:50..135:- |
| NODE\_172442\_length\_943\_cov\_10.770944\_6159 | 9.0 | 0.43 ± 0.38 |  | 31 | 24 | 0 | 7 | no |  |  |  | blast | ugcaccaguugaaggcgauguagau | uaccuuggccuccaguaacgcgguga | ugcaccaguugaaggcgauguagauguannnnnnnnnnuaccuuggccuccaguaacgcgguga | NODE\_172442\_length\_943\_cov\_10.770944:333..397:- |
| NODE\_690213\_length\_195\_cov\_65.420509\_250 | 4.2 | 0.45 ± 0.32 |  | 10 | 9 | 0 | 1 | yes |  |  |  | blast | ugggaacuucugcaacagaua | uucuauuguagaaagaaugcc | ugggaacuucugcaacagauauuguaagaacuuuuugaagaauauuuaauuucuauuguagaaagaaugcc | NODE\_690213\_length\_195\_cov\_65.420509:92..163:+ |
| NODE\_37068\_length\_205\_cov\_180.956100\_4256 | 1.3 | 0.33 ± 0.29 |  | 54 | 53 | 0 | 1 | yes |  |  |  | blast | uaacucagacgaacaggaggccgcc | cguucauuugggucagg | cguucauuugggucaggugccaucuaauaacucagacgaacaggaggccgcc | NODE\_37068\_length\_205\_cov\_180.956100:128..180:- |
| NODE\_136010\_length\_190\_cov\_158.552628\_7921 | 0.9 | 0.54 ± 0.20 |  | 11 | 10 | 1 | 0 | yes |  |  |  | blast | uggacuuggaucaggauguacugcc | gaguacagugguguguccagu | uggacuuggaucaggauguacugcccauagagagagcacugggaguacagugguguguccagu | NODE\_136010\_length\_190\_cov\_158.552628:39..102:- |
| NODE\_122862\_length\_1691\_cov\_19.710230\_6010 | 0.9 | 0.54 ± 0.20 |  | 2 | 1 | 0 | 1 | no |  | oni-miR-10853 |  | blast | cugagcuguuucucuggucucc | gucagagaugagcagcuc | cugagcuguuucucuggucuccauccucaccugugccgugaaggugaaucauuaagucagagaugagcagcuc | NODE\_122862\_length\_1691\_cov\_19.710230:677..750:+ |
| NODE\_284204\_length\_181\_cov\_61.469612\_459 | 0.9 | 0.54 ± 0.20 |  | 11 | 11 | 0 | 0 | yes |  |  |  | blast | ucauugccuggcguaucucuggaga | ucuauagcagucagugcaguggcc | ucauugccuggcguaucucuggagacauaaaugcccaccguuguuuucucuauagcagucagugcaguggcc | NODE\_284204\_length\_181\_cov\_61.469612:52..124:- |
| NODE\_125621\_length\_664\_cov\_16.481928\_4672 | 0.1 | 0.54 ± 0.20 |  | 11 | 9 | 2 | 0 | yes |  |  |  | blast | uuauuucauggggaacaggccgucc | auggccaauuauguuccucccccuuuuccu | auggccaauuauguuccucccccuuuuccuucuuuuggucagguuaaaaccugccucauccacaaagauuauuucauggggaacaggccgucc | NODE\_125621\_length\_664\_cov\_16.481928:323..416:- |
| NODE\_299827\_length\_550\_cov\_13.832727\_1693 | 0 | 0.54 ± 0.20 |  | 13 | 13 | 0 | 0 | no |  | oni-miR-10771 |  | blast | uuguuguuguuguuguuguuguaca | ugcauuccgcgaaaggcaacaacaaac | uuguuguuguuguuguuguuguacaccacggggcugcauuccgcgaaaggcaacaacaaac | NODE\_299827\_length\_550\_cov\_13.832727:473..534:- |
| NODE\_870074\_length\_765\_cov\_11.513725\_3455 | 0 | 0.54 ± 0.20 |  | 1 | 1 | 0 | 0 | yes |  | dre-miR-139-3p |  | blast | uggggagggaaggaaggg | cuggggccagcucccccgcauc | uggggagggaaggaagggaggggcaagugaccccucccuggggccagcucccccgcauc | NODE\_870074\_length\_765\_cov\_11.513725:768..827:- |

  
  
  

## mature miRBase miRNAs detected by miRDeep2

  


tag idthis is a tag id assigned by miRDeep2. The first part of the id designates the chromosome or genome contig on which the miRNA gene is located. The second part is a running number that is added to avoid identical ids. The running number is incremented by one for each potential miRNA precursor that is excised from the genome. Clicking this field will display a pdf of the structure, read signature and score breakdown of the miRNA. | miRDeep2 scorethe log-odds score assigned to the hairpin by miRDeep2 | estimated probability that the miRNA is a true positivethe estimated probability that a predicted miRNA with a score of this or higher is a true positive. To see exactly how this probability is estimated, mouse over the 'novel miRNAs, true positives' in the table at the top of the webpage. For miRBase miRNAs, this reflects the support that the data at hand lends to the miRNA. | rfam alertthis field indicates if the miRNA hairpin has sequence similarity to reference rRNAs or tRNAs. Warnings in this field should overrule the estimated probability that a reported miRNA is a true positive (previous field). | predicted mature seq. in accordance with miRBase mature seq.If the predicted miRDeep2 sequence overlaps with the miRBase annotated mature sequence than this is indicated by 'TRUE'. If the predicted miRDeep2 star sequence overlaps with the miRBase annotated mature sequence this is inidicated by 'STAR'. | total read countthis is the sum of read counts for the mature, loop and star miRNAs. | mature read countthis is the number of reads that map to the miRNA hairpin and are contained in the sequence covered by the consensus mature miRNA, including 2 nts upstream and 5 nts downstream. | loop read countthis is the number of reads that map to the miRNA hairpin and are contained in the sequence covered by the consensus miRNA loop, including 2 nts upstream and 5 nts downstream. | star read countthis is the number of reads that map to the miRNA hairpin and are contained in the sequence covered by the consensus star miRNA, including 2 nts upstream and 5 nts downstream. | significant randfold p-valuethis field indicates if the estimated randfold p-value of the miRNA hairpin is equal to or lower than 0.05 (see Bonnet et al., Bioinformatics, 2004). | mature miRBase miRNAthis field displays the ids of any reference mature miRNAs for the species that map perfectly (full length, no mismatches) to the reported miRNA hairpin. If this is the case, the reported miRNA hairpin is assigned as a known miRNA. If not, it is assigned as a novel miRNA. If more than one reference mature miRNA maps to the miRNA hairpin, then only the id of the reference miRBase miRNA that matches the predicted mature sequence is output. | example miRBase miRNA with the same seedthis field displays the ids of any reference mature miRNAs from related species that have a seed sequence identical to that of the reported mature miRNA. The seed is here defined as nucleotides 2-8 from the 5' end of the mature miRNA. If more than one reference mature miRNA have identical seed, then only the id of the miRNA that occurs last in the input file of reference mature miRNAs from related species is displayed. | UCSC browserif a species name was input to miRDeep2, then clicking this field will initiate a UCSC blat search of the consensus precursor sequence against the reference genome. | NCBI blastnclicking this field will initiate a NCBI blastn search of the consensus precursor sequence against the nr/nt database (non-redundant collection of all NCBI nucleotide sequences). | consensus mature sequencethis is the consensus mature miRNA sequence as inferred from the deep sequencing reads. | consensus star sequencethis is the consensus star miRNA sequence as inferred from the deep sequencing reads. | consensus precursor sequencethis is the consensus precursor miRNA sequence as inferred from the deep sequencing reads. Note that this is the inferred Drosha hairpin product, and therefore does not include substantial flanking genomic sequence as does most miRBase precursors. | precursor coordinateThe given precursor coordinates refer do absolute position in the mapped reference sequence || NODE\_251462\_length\_250\_cov\_44.320000\_16 | -1.3e+1 | 0.54 ± 0.20 |  | TRUE | 1046 | 1046 | 0 | 0 | no | abu-miR-10558 |  |  | blast | uccgcuuggcaucuuucuuggc | caugaugauccacuugguaa | caugaugauccacuugguaaaauaaauccgcuuggcaucuuucuuggc | NODE\_251462\_length\_250\_cov\_44.320000:290..338:- |

  
  

## mature miRBase miRNAs not detected by miRDeep2

  

miRBase precursor idClicking this field will display a pdf of the structure and read signature of the miRNA. | total read countthis is the sum of read counts for the mature and star miRNAs. | mature read count(s)this is the number of reads that map to the miRNA hairpin and are contained in the sequence covered by the mature miRNA, including 2 nts upstream and 5 nts downstream. If more than one mature sequence is given this will be a comma separated list. In parenthesis are normalized read counts shown. | star read countthis is the number of reads that map to the miRNA hairpin and are contained in the sequence covered by the star miRNA, including 2 nts upstream and 5 nts downstream. This field is empty unless a reference star miRNA was given as input to quantifier.pl. If more than one mature sequence is given this will be a comman separated list | remaining readsthis is the number of reads that did not map to any of the mature and star sequences | UCSC browserif a species name was input to miRDeep2, then clicking this field will initiate a UCSC blat search of the miRNA precursor sequence against the reference genome. | NCBI blastnclicking this field will initiate a NCBI blastn search of the miRNA precursor sequence against the nr/nt database (non-redundant collection of all NCBI nucleotide sequences). | miRBase mature sequence(s)this is/are the mature miRNA sequence(s) input to quantifier.pl. | miRBase star sequence(s)this is/are the star miRNA sequence(s) input to quantifier.pl. This field is empty unless a reference star miRNA was given as input to quantifier.pl. | miRBase precursor sequencethis is the precursor miRNA sequence input to quantifier.pl. || dre-mir-143 | 632961 | 632891 |

 0 | 70 | - | blast | |  | | --- | | ggugcagugcugcaucucuggu | | ugagaugaagcacuguagcuc | | |  | | --- | | - | | gaucuacagucgucuggcccgcggugcagugcugcaucucuggucaacugggagucugagaugaagcacuguagcucgggaggacaacacugucagcuc || dre-mir-22a | 157630 | 153558 | 0 | 4072 | - | blast | |  | | --- | | aagcugccagcugaagaacugu | | |  | | --- | | - | | gcugaccugcagcaguucuucacuggcaagcuuuauguccuuguguaccagcuaaagcugccagcugaagaacuguugugguuggc || ipu-let-7e-2 | 121713 | 121323 | 0 | 390 | - | blast | |  | | --- | | ugagguaguagauugaauaguu | | |  | | --- | | - | | ugagguaguagauugaauaguuguggaguauaaaaccucccuuugagauaacuauacaaucuacugucuuucu || tni-mir-204a | 99137 | 99054 | 0 | 83 | - | blast | |  | | --- | | uucccuuugucauccuaugccu | | |  | | --- | | - | | ugugaccugugggcuucccuuugucauccuaugccuggagcucggauaaggcagggacagcaaagggaugcucagccgucaccacugacuuca || tni-mir-181a-2 | 89149 | 89131 | 0 | 18 | - | blast | |  | | --- | | aacauucaacgcugucggugaguuu | | |  | | --- | | - | | caguuuuuucaggucuuuggggaacauucaacgcugucggugaguuuggugacucagaaaaaaaccaucgagcguugaguguaccuuuagg || fru-mir-27c | 84009 | 83544 | 0 | 465 | - | blast | |  | | --- | | uucacagugguuaaguucugcc | | |  | | --- | | - | | ggcuguguggcggcaggacuuaacccacaugugagcagugagugugugccauguucacagugguuaaguucugccgcc || gmo-mir-145 | 85273 | 85270 | 0 | 3 | - | blast | |  | | --- | | guccaguuuucccaggaaucccu | | ggauuccuggaaauacuguucu | | |  | | --- | | - | | guccaguuuucccaggaaucccuuguugaacaugaaagcgggauuccuggaaauacuguucu || hhi-mir-199a | 162867 | 54114 | 0 | 108753 | - | blast | |  | | --- | | uacaguagucugcacauugguua | | |  | | --- | | - | | gcccgccugcccaguguucagacuaccuguucaggaaguagugguuguacaguagucugcacauugguuaggcuggcuggggaagcacggg || gmo-mir-30e-2 | 48044 | 46847 | 0 | 1197 | - | blast | |  | | --- | | uguaaacauccuugacuggaagcu | | |  | | --- | | - | | uguaaacauccuugacuggaagcuagugcuuuguccuggagcuuucagucggauguuugcag || dre-let-7b | 40562 | 39077 | 0 | 1485 | - | blast | |  | | --- | | ugagguaguagguugugugguuu | | |  | | --- | | - | | ucggacagggugagguaguagguugugugguuucaggguuguguuuuugccccaucaggaguuaacuauacaaccuacugccuucccugaaggg || dre-mir-30c | 32923 | 32923 | 0 | 0 | - | blast | |  | | --- | | uguaaacauccuacacucucagcu | | |  | | --- | | - | | cuucagggaguguaaacauccuacacucucagcuggagcgcagccgaggccgggagugggauguuugcgcucucuggcu || dre-mir-451 | 24563 | 24550 | 0 | 13 | - | blast | |  | | --- | | aaaccguuaccauuacugaguuu | | |  | | --- | | - | | agaggcggcgaaaccguuaccauuacugaguuuaguaaugguaaggguucugcugccuuu || ssa-mir-462a | 20285 | 20285 | 0 | 0 | - | blast | |  | | --- | | uaacggaacccauaaugcagcug | | |  | | --- | | - | | uaacggaacccauaaugcagcugugucaaugaacagcaccuggauguggguuccgucu || ssa-let-7i-1 | 18866 | 18866 | 0 | 0 | - | blast | |  | | --- | | ugagguaguaguuugugcuguug | | |  | | --- | | - | | ugagguaguaguuugugcuguuggucggguugugacauggccugcuguggagauaacugcgcaaacgacuaccuugcc || fru-mir-200a | 15985 | 15773 | 0 | 212 | - | blast | |  | | --- | | uaacacugucugguaacgauguu | | |  | | --- | | - | | ucucaggauccaucuuacccgacagugcuggauuguacuacuguuguucuaacacugucugguaacgauguuuucugggugac || ssa-mir-25-1 | 15658 | 15656 | 0 | 2 | - | blast | |  | | --- | | cauugcacuugucucggucuga | | |  | | --- | | - | | aggcggagucuugggcaauugccggcaaucuuaagaggcauugcacuugucucggucuga || dre-mir-456 | 15237 | 15234 | 0 | 3 | - | blast | |  | | --- | | caggcugguuagaugguuguca | | |  | | --- | | - | | gcugguugugugugcaggcaucuuuccagucuacauguggauccaggagucugcaggcugguuagaugguugucacguacccagc || hhi-mir-1 | 13399 | 13396 | 0 | 3 | - | blast | |  | | --- | | acauacuucuuuauguacccau | | uggaauguaaagaaguauguau | | |  | | --- | | - | | aagauuaccuccuuggugcacauacuucuuuauguacccauaugaacauaugauagcuauggaauguaaagaaguauguauuccuggugaggugcg || tni-mir-192 | 13258 | 13258 | 0 | 0 | - | blast | |  | | --- | | ccugucaguucuguaggccacug | | augaccuaugaauugacagcca | | |  | | --- | | - | | cacgaggugaugaccuaugaauugacagccaguaacuggagccucugccugucaguucuguaggccacugcugcguccguccc || ssa-mir-8159 | 12629 | 12629 | 0 | 0 | - | blast | |  | | --- | | ucaguaacuggaaucugucccugc | | |  | | --- | | - | | ucaguaacuggaaucugucccugcagacacacuguagagcagggccggcugguuacugcgc || dre-mir-222a | 15048 | 12189 | 0 | 2859 | - | blast | |  | | --- | | agcuacaucuggcuacugggucuc | | |  | | --- | | - | | gccgguggcuuguucgggugcucaugagaugcucaguagucaguguagauccugugucacaaucagcagcuacaucuggcuacugggucucugauggcauuuucugcu || ssa-mir-15c-1 | 12053 | 12053 | 0 | 0 | - | blast | |  | | --- | | uagcagcgcaucaugguuuga | | |  | | --- | | - | | uagcagcgcaucaugguuugaaacaguguugaaaagaugcgaaucaucauuugcugcuuua || tni-mir-10d | 11622 | 11619 | 0 | 3 | - | blast | |  | | --- | | uacccuguagaaccgaaugugugug | | |  | | --- | | - | | gccggugaggugcucgucgucuauacauacccuguagaaccgaaugugugugcagcugacuugaucacagauuggguucuaggggagucuaugggcgcugaauaaucaucgaugaacggc || tni-mir-24-1 | 11183 | 11183 | 0 | 0 | - | blast | |  | | --- | | uggcucaguucagcaggaacagg | | |  | | --- | | - | | cuuccugugccuacugagcugauaugcaguuguacagaaccacuggcucaguucagcaggaacaggaguc || tni-mir-181b-2 | 10650 | 10649 | 0 | 1 | - | blast | |  | | --- | | aacauucauugcugucgguggguu | | |  | | --- | | - | | gcucgcaauaaacauucauugcugucgguggguuuccauggcaauagcucgcugaccaaugaaugaagacugcgg || fru-mir-221 | 11068 | 11068 | 0 | 0 | - | blast | |  | | --- | | accuggcauacaauguagauuucugu | | agcuacauugucugcuggguuuc | | |  | | --- | | - | | ccugaaccuggcauacaauguagauuucugugugcuuggauucuacagcuacauugucugcuggguuuccggauagc || fru-mir-128-2 | 9795 | 9792 | 0 | 3 | - | blast | |  | | --- | | ucacagugaaccggucucuuuu | | |  | | --- | | - | | gaagagggggccguuacacugucagagauguagucugagggucucacagugaaccggucucuuuuccugcug || ssa-mir-731 | 9651 | 9639 | 0 | 12 | - | blast | |  | | --- | | aaugacacguuuucucccggauug | | |  | | --- | | - | | aaugacacguuuucucccggauugccaaacacacuauaaggcaccgggaauuucgugucagca || dre-mir-126b | 8251 | 8089 | 0 | 162 | - | blast | |  | | --- | | cauuauuacuuuugguacgcg | | |  | | --- | | - | | gagucuuaccggccucacgguucauuauuacuuuugguacgcgcuaugccacucucaacucguaccgugaguaauagugcacugugacuggugaacacaacua || tni-mir-200b | 8018 | 8016 | 0 | 2 | - | blast | |  | | --- | | caucuuacgaggcagcauugga | | uaauacugccugguaaugaugau | | |  | | --- | | - | | ccaucuuacgaggcagcauuggauagcaucacuuuuucuaauacugccugguaaugaugaugaucgucgucugcagg || tni-mir-152 | 13078 | 7351 | 0 | 5727 | - | blast | |  | | --- | | ucagugcauaacagaacuuuguc | | |  | | --- | | - | | cugcucaaacucugggcuaaguucugugauacacucugacugugaauggcuaugcuagucagugcauaacagaacuuugucccgg || fru-mir-27e | 6077 | 6068 | 0 | 9 | - | blast | |  | | --- | | uucacaguggcuaaguucagug | | |  | | --- | | - | | cacuugcuaaaggcaccgagcuuagcuaauuggugagcauugaucccugcuauguguuguucacaguggcuaaguucagugccugaggugaaauaguuga || ccr-mir-101a | 6079 | 6041 | 0 | 38 | - | blast | |  | | --- | | uacaguacugugauaacugaag | | |  | | --- | | - | | ggcugcccugguucaguuaucacagugcugaugcuguccaucuuaaagguacaguacugugauaacugaaggauggcugcc || nbr-mir-725 | 5402 | 5192 | 0 | 210 | - | blast | |  | | --- | | uucagucauuguuucuggucgu | | |  | | --- | | - | | agcuggaaacuuugccugcgaauuucugugcucaaaaaauuucagucauuguuucuggucgu || dre-mir-365-3 | 4957 | 4927 | 0 | 30 | - | blast | |  | | --- | | uaaugccccuaaaaauccuuauu | | |  | | --- | | - | | aagucagcaagaaaagugagggacuuuuaggggcagcuguguuauacugacccagucauaaugccccuaaaaauccuuauugcucuugcagugcucaac || fru-mir-15a | 4824 | 4823 | 0 | 1 | - | blast | |  | | --- | | uagcagcacggaaugguuugug | | |  | | --- | | - | | cuggugaugcuguagcagcacggaaugguuuguggguuacacugagauacaggccauacugugcugccgca || gmo-mir-142-2 | 5290 | 4229 | 0 | 1061 | - | blast | |  | | --- | | cauaaaguagaaagcacuacua | | |  | | --- | | - | | cauaaaguagaaagcacuacuaaacuucaaugcaaaguguaguguuuccuacuuuaugg || dre-mir-2188 | 4117 | 4085 | 0 | 32 | - | blast | |  | | --- | | aagguccaaccucacauguccu | | |  | | --- | | - | | ggacaauaucagcaaugugagaaagguccaaccucacauguccugugaggcugaaggaaggcugugugagguuagaccuaucccacacggcccguauucuuccc || abu-mir-19c | 4127 | 4072 | 0 | 55 | - | blast | |  | | --- | | ugugcaaauccaugcaaagcucu | | |  | | --- | | - | | aguuuugaugguuuguucucagcuucugaaagacucugcugugcaaauccaugcaaagcucu || abu-mir-1388 | 4325 | 4040 | 0 | 285 | - | blast | |  | | --- | | aggacuguccaaccugagaaugg | | |  | | --- | | - | | aggacuguccaaccugagaauggugauuauaggcucaaucucagguucgucagcccaug || dre-mir-140 | 4324 | 4324 | 0 | 0 | - | blast | |  | | --- | | cagugguuuuacccuaugguag | | accacaggguagaaccacggacg | | |  | | --- | | - | | guguuugucuccugugucccgucagugguuuuacccuaugguagguuacgucaugcuguucuaccacaggguagaaccacggacgggaugucuggaggugucugc || tni-mir-103 | 3395 | 3388 | 0 | 7 | - | blast | |  | | --- | | agcagcauuguacagggcuauga | | |  | | --- | | - | | uugcucuacgcuuuuagccucuuuacagugcugccuugcucuacucauguucaagcagcauuguacagggcuaugacagcguagag || mze-mir-10553 | 3345 | 3342 | 0 | 3 | - | blast | |  | | --- | | gugccauaucugacuguacgca | | |  | | --- | | - | | gugccauaucugacuguacgcaauuguaucauuauauugcguuaucagcgauauagcaaug || ssa-mir-93a-2 | 3317 | 3317 | 0 | 0 | - | blast | |  | | --- | | aaaagugcuguuugugcagguag | | |  | | --- | | - | | aaaagugcuguuugugcagguagcugacucacccgcccuacugcaaaaccagcacuucccga || pol-mir-144 | 5152 | 3310 | 0 | 1842 | - | blast | |  | | --- | | cuacaguauagaugauguacua | | |  | | --- | | - | | gcggggauaucaucuuauacuguaaguuuauuauagagacacuacaguauagaugauguacuaucccg || ssa-mir-205b-3 | 2994 | 2994 | 0 | 0 | - | blast | |  | | --- | | uccuucauuccaccggagucugu | | |  | | --- | | - | | uccuucauuccaccggagucugugccuuuauccaaacagauuucagugaagugaagugua || hhi-mir-737 | 2836 | 2836 | 0 | 0 | - | blast | |  | | --- | | guuuuuuuagguuuugauuuuu | | |  | | --- | | - | | guucuacucuucugugguuuuuuuagguuuugauuuuuguaaacguuggaugagaaaaucaaaaccuaaa || ccr-mir-130b | 2853 | 2853 | 0 | 0 | - | blast | |  | | --- | | cagugcaauaaugaaagggcauc | | acucuuucccuguugcacuacu | | |  | | --- | | - | | guuugucuguugccugacacucuuucccuguugcacuacugugggagcugcagcuagcagugcaauaaugaaagggcaucaguccacuggaccagac || dre-mir-19a | 3381 | 3381 | 0 | 0 | - | blast | |  | | --- | | cuaguuuugcauaguugcacua | | ugugcaaaucuaugcaaaacugau | | |  | | --- | | - | | uggugcaguucucugcuaguuuugcauaguugcacuacaagaaaacgggaguugugcaaaucuaugcaaaacugaugguggccugcugc || abu-mir-458 | 2092 | 2081 | 0 | 11 | - | blast | |  | | --- | | auagcucuuuaaaugguacugc | | |  | | --- | | - | | agcgccauuuucagagcuauaagagugaaaguuaucauagcucuuuaaaugguacugc || oni-mir-106 | 2170 | 2052 | 0 | 118 | - | blast | |  | | --- | | uaaagugcuuacagugcagguag | | |  | | --- | | - | | uaaagugcuuacagugcagguagugaugauaaucuggccuacugcagugugagcacuucuuuc || ssa-mir-129-2 | 2156 | 2156 | 0 | 0 | - | blast | |  | | --- | | cuuuuugcggucugggcuugcu | | aagcccuuaccccaaaaagcau | | |  | | --- | | - | | cuuuuugcggucugggcuugcuguuaacacaccucaacccaggaagcccuuaccccaaaaagcau || dre-mir-142a | 9078 | 9061 | 0 | 17 | - | blast | |  | | --- | | uguaguguuuccuacuuuauggau | | cauaaaguagaaagcacuacua | | |  | | --- | | - | | cguacagugcagucauccauaaaguagaaagcacuacuaaaccccucgccacaguguaguguuuccuacuuuauggaugaguguacuguug || hhi-mir-187 | 1681 | 1642 | 0 | 39 | - | blast | |  | | --- | | ucgugucuuguguugcagccagu | | |  | | --- | | - | | gugaccucucuggccgggccaagggcugcaacacaggacauggguccugcucuccuccccgcucgugucuuguguugcagccaguggagcugccug || dre-mir-27d | 1612 | 1609 | 0 | 3 | - | blast | |  | | --- | | uucacaguggcuaaguucuucac | | |  | | --- | | - | | uucugagcgggugcagagcuuggcugauuggugaacgugcauggcuuguguuuuuguucacaguggcuaaguucuucacccgaaaagaa || tni-mir-223 | 1625 | 1564 | 0 | 61 | - | blast | |  | | --- | | ugucaguuugucaaauaccccaa | | |  | | --- | | - | | caggcccuucacuuaguguauuugacaagcuguguuugacacucuguaucugcgagugucaguuugucaaauaccccaagugagg || tni-let-7h | 11046 | 1559 | 0 | 9487 | - | blast | |  | | --- | | cuauacaacuuacugccuuccu | | |  | | --- | | - | | aauuggcuuugcuguggugagguaguaaguuguguuguuguuggggaucaagauugugcacccugucaaggagauaacuauacaacuuacugccuuccu || hhi-mir-183 | 1299 | 1294 | 0 | 5 | - | blast | |  | | --- | | uauggcacugguagaauucacug | | |  | | --- | | - | | ccucauaucuccuccuguucuguguauggcacugguagaauucacugucacagcacacaaucagugaauuaccauagggccauaaacagaguagagacagaucc || oni-mir-10558 | 1329 | 1109 | 0 | 220 | - | blast | |  | | --- | | auccguugggcaucuuucuuugcc | | |  | | --- | | - | | auccguugggcaucuuucuuugccuuuagacaauaauucugauggcaaaagagccaaacgggacagg || ssa-mir-7132b | 1244 | 1244 | 0 | 0 | - | blast | |  | | --- | | gacuuggucaaagcuccucagc | | ugaggcguuuagaacaaguuca | | |  | | --- | | - | | gacuuggucaaagcuccucagcagauguuucagagaauccugaggcguuuagaacaaguuca || ssa-mir-26d | 2206 | 1070 | 0 | 1136 | - | blast | |  | | --- | | ccuauucgggaugacuugguuc | | |  | | --- | | - | | uucaaguaaucuaggauaggcuuguuuaagcagggagagccuauucgggaugacuugguuc || fru-let-7a-3 | 333301 | 1032 | 0 | 332269 | - | blast | |  | | --- | | cuauacagucuauugccuuccu | | |  | | --- | | - | | cagggugagguaguagguuguauaguuuggugggugggauugcccgcccaggugauaacuauacagucuauugccuuccuugaggagcucacug || gmo-mir-8160a | 1027 | 1027 | 0 | 0 | - | blast | |  | | --- | | agaauaaugccagcagucgguc | | |  | | --- | | - | | agaauaaugccagcagucggucagcguguagcagagacccacccugguguuauuagga || nbr-mir-135b-2 | 994 | 994 | 0 | 0 | - | blast | |  | | --- | | uauggcuuucuauuccuauguga | | cauguaggguuuaaagccauug | | |  | | --- | | - | | uauggcuuucuauuccuaugugaguucuuucuaacaugucauguaggguuuaaagccauuggau || dre-mir-1388 | 4936 | 906 | 0 | 4030 | - | blast | |  | | --- | | aucucagguucgucagcccauga | | |  | | --- | | - | | ugauggaugagcagugcuuuccaggacuguccaaccugagaaugcuugaguuuuggucaaucucagguucgucagcccaugaaaaacugucucgcucaaagcc || dre-mir-21-1 | 487454 | 810 | 0 | 486644 | - | blast | |  | | --- | | cgacaacagucuguaggcuguc | | |  | | --- | | - | | uuaugugucuuuauuggcguggauauaagucuuucccagugugucagauagcuuaucagacugguguuggcuguuacauucgcccggcgacaacagucuguaggcugucugacauuuugggcauuuucuucuccgauuaaaaauauga || oni-mir-10597a | 1035 | 798 | 0 | 237 | - | blast | |  | | --- | | gaugacuggauuuguaaagcgc | | |  | | --- | | - | | gaugacuggauuuguaaagcgcuugggaccaaguaaagcgcuauacaaauacaggccauu || fru-mir-96 | 801 | 801 | 0 | 0 | - | blast | |  | | --- | | uuuggcacuagcacauuuuugcu | | caauuauguguagugccaauau | | |  | | --- | | - | | cucuucuucgcccauuuuggcacuagcacauuuuugcuucuguauguauacuuugagcaauuauguguagugccaauauaggagaagacagacuuucaaccu || oni-mir-457 | 770 | 769 | 0 | 1 | - | blast | |  | | --- | | uagcagcacaucauuacuggua | | |  | | --- | | - | | uagcagcacaucauuacugguauuucuuuucacugcauuaccaguauguuaugugcugcuuca || dre-mir-190a | 1105 | 764 | 0 | 341 | - | blast | |  | | --- | | ugauauguuugauauauuagguug | | |  | | --- | | - | | ucuggaggugagguagaccuggaagccuuucugcaggccucuguuugauauguuugauauauuagguuguuauucuguccaacuauauaucaaacauauuccuacaguguccugcucugucuccag || abu-mir-7133-2 | 838 | 834 | 0 | 4 | - | blast | |  | | --- | | uaguuugauacacagcacaaua | | gauguugaguaucaaacuguau | | |  | | --- | | - | | gauguugaguaucaaacuguauguuauguaugcuauauauaguuugauacacagcacaaua || fru-mir-210 | 1555 | 646 | 0 | 909 | - | blast | |  | | --- | | agccacugacuaacgcacauug | | |  | | --- | | - | | ucuaaaagcaggugagccacugacuaacgcacauugcgccaguugacaguuccacugugcgugugacagcggcuaaccugguuuugggaacgcuucug || ipu-mir-499-2 | 624 | 624 | 0 | 0 | - | blast | |  | | --- | | aacaucacuuuaagucugugcu | | uuaagacuugcagugauguuuag | | |  | | --- | | - | | uuaagacuugcagugauguuuagguaaucugacccaugaacaucacuuuaagucugugcu || ccr-mir-722 | 659 | 659 | 0 | 0 | - | blast | |  | | --- | | auuugaaacguuuuagccaaa | | uuuuuugcagaaacguuucagauu | | |  | | --- | | - | | acuggaacagaauggaauuugaaacguuuuagccaaaaauguuuccaugguaaagguguuuuuugcagaaacguuucagauuucguucuguucu || ssa-mir-139-2 | 560 | 560 | 0 | 0 | - | blast | |  | | --- | | ucuacagugcaugugucuccagu | | |  | | --- | | - | | ucuacagugcaugugucuccagugaauaugagcuacuggagaggcaguucugugugagu || tni-mir-429 | 556 | 555 | 0 | 1 | - | blast | |  | | --- | | gucuuaccagacaugguuag | | uaauacugucugguaaugccgu | | |  | | --- | | - | | agccuguugauaggcgucuuaccagacaugguuagauguaauuauuguugucuaauacugucugguaaugccguccauuaaauggca || gmo-mir-29c | 552 | 552 | 0 | 0 | - | blast | |  | | --- | | cuagcaccauaugaaaucagugu | | |  | | --- | | - | | cugauuuccucuggugguuuagguguugcuaauuacagucuagcaccauaugaaaucagugu || fru-mir-214 | 7127 | 517 | 0 | 6610 | - | blast | |  | | --- | | ugccugucuacacuugcugugc | | |  | | --- | | - | | caggaugagacuguuguggugugucugccugucuacacuugcugugcagaccuucugcuccuguacagcaggcacagacaggcagacagauggcagccc || dre-mir-101b | 2929 | 499 | 0 | 2430 | - | blast | |  | | --- | | caguuaucaugguaccggugcugu | | |  | | --- | | - | | gcuccuccgucaugaauuguccauuuucaguuaucaugguaccggugcugugugccugucaaguacaguacuaugauaacugaagauugacggugccaaacaucaguggagu || ccr-mir-724 | 471 | 471 | 0 | 0 | - | blast | |  | | --- | | cagccacaccuuccuuuuaaga | | uuaaagggaauuugcgacuguu | | |  | | --- | | - | | cagcagacuggauuuaaagggaauuugcgacuguuuagucacauuauuaaaacagccacaccuuccuuuuaagaucuugccugcug || ssa-mir-155-1 | 409 | 409 | 0 | 0 | - | blast | |  | | --- | | uuaaugcuaaucgugauagggguu | | |  | | --- | | - | | uuaaugcuaaucgugauagggguucuguuguuuaaaaaaaagacaccuagcauguaagcauuagc || abu-mir-29c | 958 | 958 | 0 | 0 | - | blast | |  | | --- | | cugauuucauuuggugacguaga | | cuagcaccauaugaaaucagugu | | |  | | --- | | - | | cugauuucauuuggugacguagauguuuguacacagucucuagcaccauaugaaaucagugu || ccr-mir-727 | 622 | 396 | 0 | 226 | - | blast | |  | | --- | | ucagucuucaauuccucccagc | | |  | | --- | | - | | accuguaugucauuuucagucuucaauuccucccagcccguaaccauggaaacugugaguugaggcgaguugaagacuuaaagugcuguacag || dre-mir-27b | 98866 | 373 | 0 | 98493 | - | blast | |  | | --- | | agagcuuagcugauuggugaaca | | |  | | --- | | - | | ucuuuucuagcaggugcagagcuuagcugauuggugaacagugauugaacucuuuguucacaguggcuaaguucugcaucugaggagagga || tni-mir-194 | 365 | 365 | 0 | 0 | - | blast | |  | | --- | | uguaacagcaacuccauguggaa | | |  | | --- | | - | | gcgcgcuggauguaacagcaacuccauguggaagcuguauguguguuccaguggaagugcuguuaccugcagagauccacc || oni-mir-7565 | 340 | 335 | 0 | 5 | - | blast | |  | | --- | | uuccugcugaacugagccagu | | |  | | --- | | - | | uuccugcugaacugagccagugauucuguaggacugauuaucagcucaguaggcacag || abu-mir-734 | 602 | 602 | 0 | 0 | - | blast | |  | | --- | | gaacuauucugcaacauuuguu | | uaaaugcugcagaauugugcuc | | |  | | --- | | - | | ugaacuauucugcaacauuuguugaugugguucugcaaguaaaugcugcagaauugugcuc || dre-mir-2187 | 394 | 368 | 0 | 26 | - | blast | |  | | --- | | uuacaggcuaugcuaaucuaugc | | uuaauuaguauagccuguuuuag | | |  | | --- | | - | | ucugaauugaugauucuggcuuuaauuaguauagccuguuuuagugaucacaucaauucuuuacaggcuaugcuaaucuaugccagaauuagcaaugcaugugg || fru-mir-489 | 290 | 288 | 0 | 2 | - | blast | |  | | --- | | gugacaucauauguacggcugcu | | |  | | --- | | - | | cuucugauacuggugguggcuugguggucguaugcaugacgucauuuacuucgaugauuggagugacaucauauguacggcugcuaaacugcuacaagagac || dre-mir-130c-1 | 856 | 278 | 0 | 578 | - | blast | |  | | --- | | gcccuuuuucuguuguacuacu | | |  | | --- | | - | | gggagaggucuguacggugauauugaccauguuuuguccauugcccuuuuucuguuguacuacuggccaaucagaagagcagugcaauauuaaaagggcauuggcugauagaacagaguccuucaccucucacccucuccu || gmo-mir-10545 | 274 | 273 | 0 | 1 | - | blast | |  | | --- | | uaagucucacaccagugcaaaac | | |  | | --- | | - | | uaagucucacaccagugcaaaacaaaacacaucaagcugcucgggugugggaccauga || fru-mir-24-1 | 11456 | 11454 | 0 | 2 | - | blast | |  | | --- | | uggcucaguucagcaggaacagg | | gugccuacugaacugguaucagu | | |  | | --- | | - | | ggguugugcccuccugugccuacugaacugguaucagugucauacuggaaaacuggcucaguucagcaggaacaggagu || dre-mir-153a | 290 | 290 | 0 | 0 | - | blast | |  | | --- | | uugcauagucacaaaagugaucau | | gucauuuuugugauguugcagcu | | |  | | --- | | - | | gguugccagugucauuuuugugauguugcagcuaguuauaugagcccaguugcauagucacaaaagugaucauuggaaacu || tni-mir-9-1 | 494 | 494 | 0 | 0 | - | blast | |  | | --- | | ucuuugguuaucuagcuguauga | | auaaagcuagauaaccgaaagu | | |  | | --- | | - | | cagagggguugucuguuaucuuugguuaucuagcuguaugagugacguacaaucuucauaaagcuagauaaccgaaaguaacaagaaucccauua || abu-mir-29d | 261 | 245 | 0 | 16 | - | blast | |  | | --- | | uagcaccauuugaaaucgguguu | | |  | | --- | | - | | acugguuucagauggugucuuagaguacaaaauaccaucuagcaccauuugaaaucgguguu || nbr-mir-99a | 233 | 228 | 0 | 5 | - | blast | |  | | --- | | agaccuguagauacaagcuugu | | |  | | --- | | - | | agaccuguagauacaagcuuguggugucacuuaacacaaguucggaucuacggguuugg || mze-mir-731 | 9969 | 9969 | 0 | 0 | - | blast | |  | | --- | | aaugacacguuuucucccggauug | | aaccgggaauuucgugucagcc | | |  | | --- | | - | | aaugacacguuuucucccggauugcugugagcaggaaacgcaaccgggaauuucgugucagcca || fru-mir-24-2 | 11375 | 11374 | 0 | 1 | - | blast | |  | | --- | | ugccugcugugcugauaaucagu | | uggcucaguucagcaggaacagg | | |  | | --- | | - | | gggucagucuccugugccugcugugcugauaaucagugugugacguuggcuggcucaguucagcaggaacaggggacugguc || ssa-mir-138-3 | 164 | 164 | 0 | 0 | - | blast | |  | | --- | | agcugguguugugaaucaggccg | | |  | | --- | | - | | agcugguguugugaaucaggccgcugugguguugagaccggcuacuucccaacaccaggguc || abu-mir-10556a | 153 | 153 | 0 | 0 | - | blast | |  | | --- | | aaagaaaagcgucuggacuucu | | |  | | --- | | - | | aaagaaaagcgucuggacuucuuuucaaguaacuuaaagaaaacccgacgcuuuucuuugc || gmo-mir-727b | 303 | 152 | 0 | 151 | - | blast | |  | | --- | | guugaggcgaguugaagacuuu | | |  | | --- | | - | | ucagucuucaguuccucccagcccaugagcauggcaaagguguguugaggcgaguugaagacuuua || dre-mir-132-2 | 233 | 151 | 0 | 82 | - | blast | |  | | --- | | accguggcuuuagauuguuacu | | |  | | --- | | - | | ucccuuccucugcugucuccaugcugaccguggcuuuagauuguuacuguagcguuggcacgugguaacagucuacagccauggucgccaaggggcagguguuacagcgacuacuggga || dre-mir-455-2 | 188 | 188 | 0 | 0 | - | blast | |  | | --- | | augcaguccaugggcauauacac | | guaugugcccuuggacuacauu | | |  | | --- | | - | | aucguuguucccugaagugaggguaugugcccuuggacuacauuguggaggccagcaccaugcaguccaugggcauauacacuuacuucauggccuacaucaaca || oni-mir-7147 | 153 | 140 | 0 | 13 | - | blast | |  | | --- | | uguaccaugcugguagccagug | | |  | | --- | | - | | uguaccaugcugguagccagugugugguaggcuuugcuggugaccagcguugugccuca || tni-mir-22b | 335 | 137 | 0 | 198 | - | blast | |  | | --- | | cguucuucacuggcuagcuuua | | |  | | --- | | - | | guugccucacagucguucuucacuggcuagcuuuauguccuccgaccccacgcuaaagcugccaguugaagagcuguuguguguaac || oni-mir-34b | 155 | 137 | 0 | 18 | - | blast | |  | | --- | | cugguaguguaguuagugauu | | |  | | --- | | - | | cugguaguguaguuagugauuaguguguaagaacaaucagcuaacgacacugccuaua || fru-mir-193 | 177 | 177 | 0 | 0 | - | blast | |  | | --- | | ugggucuuugcgggcaagguga | | aacuggccuacaaagucccagu | | |  | | --- | | - | | ugugucagaagcugggucuuugcgggcaaggugaguccucaguuuguucaacuggccuacaaagucccagucucuggcucacguaacc || hhi-mir-196 | 145 | 135 | 0 | 10 | - | blast | |  | | --- | | uagguagucucauguuguugggc | | |  | | --- | | - | | gcggacuguugagugguuuagguagucucauguuguugggcuaaauuauuucucccacaacacgaaacugccuugauuaccucagua || oni-mir-10643 | 122 | 121 | 0 | 1 | - | blast | |  | | --- | | aguggcuuuggucagcuuugg | | |  | | --- | | - | | aguggcuuuggucagcuuugguaaaugucaugcaccugagcaacaaaaggccucuua || nbr-mir-10568 | 122 | 118 | 0 | 4 | - | blast | |  | | --- | | uguccggcagaugggaacaucucuc | | |  | | --- | | - | | uguccggcagaugggaacaucucucucuuucuaaauuuugacagauuucacacuugccacauc || ssa-mir-7a-6 | 11879 | 115 | 0 | 11764 | - | blast | |  | | --- | | caacaagucccagucugccucu | | |  | | --- | | - | | uggaagacuagugauuuuguugugaugcugagcaaugagaauaacaacaagucccagucugccucu || mze-mir-10554-3 | 151 | 96 | 0 | 55 | - | blast | |  | | --- | | ucgagcgcagauucacugagc | | |  | | --- | | - | | ucaguggaucugcguuugacuacuccaccuaggcugcacugucgagcgcagauucacugagc || dre-mir-133a-1 | 6590 | 89 | 0 | 6501 | - | blast | |  | | --- | | agcugguaaaauggaaccaaauc | | |  | | --- | | - | | caucaaaccacaaugcuuugcuaaagcugguaaaauggaaccaaaucaccucuucaauggauuugguccccuucaaccagcuguagcuaugcuuugaug || ssa-mir-212b-1 | 111 | 87 | 0 | 24 | - | blast | |  | | --- | | accuuggcucuagacugcuuacu | | |  | | --- | | - | | accuuggcucuagacugcuuacugcuuaaaacucagugaaacuacaguaacagucuacagucauggcu || dre-mir-338-3 | 3832 | 86 | 0 | 3746 | - | blast | |  | | --- | | aacaauauccuggugcugccugagu | | |  | | --- | | - | | ggugucuccuggcaacaauauccuggugcugccugaguacaucucacagacuccagcaucagugauuuuguugccgggggaaaac || ssa-mir-301b | 83 | 83 | 0 | 0 | - | blast | |  | | --- | | gcuuugacgauguugcacuacu | | |  | | --- | | - | | gcuuugacgauguugcacuacuguuccaucggcugaagcagugcaacaguauugucauggc || dre-mir-24b | 8065 | 82 | 0 | 7983 | - | blast | |  | | --- | | gugccuacugagcugauaacagu | | |  | | --- | | - | | gguucaaccuccugugccuacugagcugauaacaguugauguauuuagcacuggcucaguucagcaggaaccggaguuaagccc || ssa-mir-730a-1 | 76 | 76 | 0 | 0 | - | blast | |  | | --- | | uccucauugugcaugcugugugu | | cacagcgccugcaauguggagg | | |  | | --- | | - | | uccucauugugcaugcuguguguguuucuuauggguccacacagcgccugcaauguggagg || ccr-mir-301a | 139 | 63 | 0 | 76 | - | blast | |  | | --- | | gcucugacuucauugcacuacu | | |  | | --- | | - | | acaucaagugcuguuaacaggugcucugacuucauugcacuacuguauuggacagcuagcagugcaauaguauugucaaagcaucugagagcagc || dre-mir-181a-3 | 89188 | 89188 | 0 | 0 | - | blast | |  | | --- | | accaucgaguguugaguguacc | | aacauucaacgcugucggugaguuu | | |  | | --- | | - | | gcgagucucagagaacauucaacgcugucggugaguuugcaagugagaaaaaccaucgaguguugaguguacccugccucucga || dre-mir-124-6 | 53 | 53 | 0 | 0 | - | blast | |  | | --- | | uaaggcacgcggugaaugccaa | | |  | | --- | | - | | ggguggugacacaggcccgccacucugcguguucacggcggaccuugauuuaauauccauacaauuaaggcacgcggugaaugccaagagaggggucuuaaaacgacaaaccc || abu-mir-10545 | 322 | 322 | 0 | 0 | - | blast | |  | | --- | | cugcucaggugugggaccaua | | uaagucucacaccagugcaaaac | | |  | | --- | | - | | uaagucucacaccagugcaaaacaaaaaucaugacgcugcucaggugugggaccauaa || abu-mir-728a | 180 | 47 | 0 | 133 | - | blast | |  | | --- | | auacuaaguauacuacguuuac | | |  | | --- | | - | | aaauguaguagacuauaaguauacaugaaacucagaaaguauacuaaguauacuacguuuac || oni-mir-10683 | 42 | 42 | 0 | 0 | - | blast | |  | | --- | | ugaaacguguuugauugggca | | |  | | --- | | - | | accaaucaaacacguuccaccuuuuugccgugaaacguguuugauugggca || fru-mir-10b-1 | 181040 | 41 | 0 | 180999 | - | blast | |  | | --- | | acagauucgauucuaggggagu | | |  | | --- | | - | | auauauacccuguagaaccgaauuugugugauggcgucaaagucacagauucgauucuaggggaguauau || oni-mir-10729 | 41 | 41 | 0 | 0 | - | blast | |  | | --- | | uaaggcugaaccaauggacu | | |  | | --- | | - | | uaaggcugaaccaauggacugugcauuucuaucaaguucaagggucucggccucaucu || fru-let-7a-1 | 316351 | 41 | 0 | 316310 | - | blast | |  | | --- | | cuguacagccuccuagcuuucc | | |  | | --- | | - | | cagguugagguaguagguuguauaguugagagugacaccacaggagaugacuguacagccuccuagcuuucccu || abu-mir-10554-2 | 75 | 39 | 0 | 36 | - | blast | |  | | --- | | ucaguggaucugugcucgacagu | | |  | | --- | | - | | ucaguggaucugugcucgacagugcagccuaggcagaguagucgaacgcaaauccucuga || oni-mir-10627 | 39 | 39 | 0 | 0 | - | blast | |  | | --- | | guuucacggaccggccuuuaaggu | | |  | | --- | | - | | cuccacggaccgguuuaaugucagacaaaaguuucacggaccggccuuuaaggu || fru-mir-218b | 37 | 37 | 0 | 0 | - | blast | |  | | --- | | uugugcuugaucuaaccaugca | | |  | | --- | | - | | caggaccccauugugcuugaucuaaccaugcagugcaucguguguccaugguugugccaagcaccuuggaggcuug || oni-mir-10622-1 | 56 | 35 | 0 | 21 | - | blast | |  | | --- | | uguagagucuguguguggagcu | | |  | | --- | | - | | uguagagucuguguguggagcuucugcaguuuguguugaagcguccacacucagcucugcagc || pny-mir-430a-1 | 34 | 34 | 0 | 0 | - | blast | |  | | --- | | uaagugcugauuguuguugugu | | |  | | --- | | - | | acggcaacauaggcacuuauuugaauucugaauauaguaagugcugauuguuguugugu || dre-mir-489 | 322 | 322 | 0 | 0 | - | blast | |  | | --- | | gugacaucauauguacggcugcu | | uggucguauguaugacgucauu | | |  | | --- | | - | | acugacuugagugguggccugguggucguauguaugacgucauuuacuucaaaguuuggagugacaucauauguacggcugcuaaacugcuacauggcucauca || oni-mir-10902 | 63 | 33 | 0 | 30 | - | blast | |  | | --- | | ugacacugguggugcugaucgcccc | | |  | | --- | | - | | ucacagcaagaaugcaccagugauggccccucacaggaagguggugacacugguggugcugaucgcccc || fru-mir-212 | 120 | 120 | 0 | 0 | - | blast | |  | | --- | | uaacagucuacagucauggcuac | | accuuggcucuagacugcuuacu | | |  | | --- | | - | | ggucagcgcaucaacaccuuggcucuagacugcuuacugcuaaaaccgcucauaaguacaguaacagucuacagucauggcuacugacgccugac || abu-mir-10556b | 70 | 31 | 0 | 39 | - | blast | |  | | --- | | ggaagaaaaacgucuggacuuc | | |  | | --- | | - | | ggaagaaaaacgucuggacuucuuuaaguuucuugaagaugaaguccagaugcuuuucuuucca || mze-mir-10552 | 32 | 31 | 0 | 1 | - | blast | |  | | --- | | uccuccaugcacuuugaugacu | | |  | | --- | | - | | uccuccaugcacuuugaugacuuauuuugucaaacuguccucagaaugcauggggaugg || dre-mir-23b-2 | 2072 | 31 | 0 | 2041 | - | blast | |  | | --- | | ggguuccuggcgugcugauuu | | |  | | --- | | - | | cugcuggcuguguggguuccuggcgugcugauuugugacuuaagauaaaaaucacauugccagggauuaccacacaaccgccac || abu-mir-15c | 304 | 31 | 0 | 273 | - | blast | |  | | --- | | caaaucaucaugugcugccacc | | |  | | --- | | - | | uagcagcacaucauguuuugcagaugugcugaaucgcuucaaaucaucaugugcugccacc || fru-mir-460 | 498 | 29 | 0 | 469 | - | blast | |  | | --- | | cacagcgcauacaauguggaugc | | |  | | --- | | - | | caguuccugcauuguacacacugugcaaaucauuucuggaagcacagcgcauacaauguggaugcugug || gmo-mir-33b-1 | 27 | 27 | 0 | 0 | - | blast | |  | | --- | | gugcauuguaguugcauugcau | | |  | | --- | | - | | gugcauuguaguugcauugcauguguuucugccgggugcaaugugucugcagcgcaaca || fru-let-7e | 124484 | 124483 | 0 | 1 | - | blast | |  | | --- | | cuauacaaucuacugucuuucc | | ugagguaguagauugaauaguu | | |  | | --- | | - | | gcuguccuugggguugagguaguagauugaauaguugugggguugugugaccucuuagugagauaacuauacaaucuacugucuuuccuaaggagacagc || abu-mir-449b | 32 | 32 | 0 | 0 | - | blast | |  | | --- | | uggcaguguaauguuagcugac | | cagcucacacugcucugcuacu | | |  | | --- | | - | | uggcaguguaauguuagcugacguuucuucagccagcucacacugcucugcuacu || abu-mir-10557 | 29 | 23 | 0 | 6 | - | blast | |  | | --- | | acuggcauagauuugaaacuug | | |  | | --- | | - | | ugguuucaguucaugccagaacaguauaugcaaaucuacuggcauagauuugaaacuug || pol-mir-206 | 22 | 22 | 0 | 0 | - | blast | |  | | --- | | uggaauguaaggaagugugugg | | |  | | --- | | - | | acaugcuuccuuauauccccauauucauacagcacuuauggaauguaaggaagugugugg || oni-mir-10562 | 24 | 19 | 0 | 5 | - | blast | |  | | --- | | agccaaugagcgugcggacuca | | |  | | --- | | - | | cccgcacgcucauuggauaacgaugugucaaucucgagcuauuagccaaugagcgugcggacuca || oni-mir-10608a | 86 | 19 | 0 | 67 | - | blast | |  | | --- | | ucggcugcccugggaaugccu | | |  | | --- | | - | | gcagacccaggacaugcuggagagauuauaucucucggcugcccugggaaugccu || oni-mir-7552 | 18 | 18 | 0 | 0 | - | blast | |  | | --- | | uuacaauuaaaggauauuucuc | | |  | | --- | | - | | uuacaauuaaaggauauuucucguggauguaauaaaaacggaaauaucucuuaauuguuuggu || dre-mir-1306 | 21 | 17 | 0 | 4 | - | blast | |  | | --- | | ccaccuccccugcaaacgucca | | |  | | --- | | - | | uccaccaccuccccugcaaacguccagugacgcagaggaaauggacguuagcucugguggugauggaca || oni-mir-10585 | 21 | 17 | 0 | 4 | - | blast | |  | | --- | | cugaugaaaucacucuauguuccu | | |  | | --- | | - | | cugaugaaaucacucuauguuccugcuugcugauacaggauagguugauuucauugcau || hhi-mir-728 | 19 | 16 | 0 | 3 | - | blast | |  | | --- | | aaauguaguagacuuuaaguaua | | |  | | --- | | - | | ggcgcccuggggaaauguaguagacuuuaaguauacguguggaaccggagaguauacuaaguacacuacguuuauccagggg || abu-mir-10544 | 16 | 15 | 0 | 1 | - | blast | |  | | --- | | uaggcgugucacugcgugucaca | | |  | | --- | | - | | uaggcgugucacugcgugucacagucacugcuugcgcacggggccacgcccugc || fru-mir-135b | 210 | 15 | 0 | 195 | - | blast | |  | | --- | | uauagggauggaagccaugc | | |  | | --- | | - | | augcccagugcacuguauggcuuuuuauuccuaucugacuguacugauaguucauauagggauggaagccaugcacugcgcug || oni-mir-10578 | 18 | 14 | 0 | 4 | - | blast | |  | | --- | | ugcagugagucagucugaagcu | | |  | | --- | | - | | ugcagugagucagucugaagcugucugacuguaucagcugacagauggcucucugaagu || oni-mir-199b-2 | 14 | 14 | 0 | 0 | - | blast | |  | | --- | | uaaccaaugugcagacuacugu | | |  | | --- | | - | | uaaccaaugugcagacuacuguacaccaguaagauccugaacagguagucugaacacugggauga || tni-mir-19b | 4643 | 14 | 0 | 4629 | - | blast | |  | | --- | | aguuuugcugguuugcauucagc | | |  | | --- | | - | | caaucaucguuuggucaguuuugcugguuugcauucagcuuuguugcugugcugugcaaauccaugcaaaacugacu || pny-mir-10555 | 13 | 13 | 0 | 0 | - | blast | |  | | --- | | gaaauagcagcagcuugucugg | | ccucaagcugcuguugcucgucu | | |  | | --- | | - | | ccucaagcugcuguugcucgucuguggcgauccagaaauagcagcagcuugucugga || gmo-mir-150 | 13 | 13 | 0 | 0 | - | blast | |  | | --- | | ucucccaauccuuguaccaguguc | | |  | | --- | | - | | ucucccaauccuuguaccagugucguggugucggccgacgcugggcaggcuuugggggg || oni-mir-10960 | 12 | 12 | 0 | 0 | - | blast | |  | | --- | | ucacuuggacacucuucuuggc | | |  | | --- | | - | | cugguagaauggcccaugauggagagcuauaaauagcuucacuuggacacucuucuuggc || fru-mir-205 | 3015 | 3015 | 0 | 0 | - | blast | |  | | --- | | uccuucauuccaccggagucugu | | gauuucaguggugugaagugua | | |  | | --- | | - | | uguguucuauccuucauuccaccggagucuguauguguauuuaaccagauuucaguggugugaaguguaagagacaugg || tni-mir-184 | 1524 | 11 | 0 | 1513 | - | blast | |  | | --- | | uccuuaucacuuuuccagccca | | |  | | --- | | - | | cgcucacaucuccuuaucacuuuuccagcccagcuauagcuucugaauccguuggacggagaacugauaaggg || abu-mir-7132b | 44 | 11 | 0 | 33 | - | blast | |  | | --- | | ugagaaguuuugaacaaguaua | | |  | | --- | | - | | gacuugguccaagcuccucagugcuguuuauauaccugagaaguuuugaacaaguaua || oni-mir-10714 | 10 | 10 | 0 | 0 | - | blast | |  | | --- | | uacaggagcuacugccacguucc | | |  | | --- | | - | | uacaggagcuacugccacguuccacgggugaugaggcagcugugcguggcucagcucugcagc || oni-mir-10658 | 11 | 10 | 0 | 1 | - | blast | |  | | --- | | augagaaugcugacagggagcu | | |  | | --- | | - | | augagaaugcugacagggagcuauggcuuuagcaaugcugccugucacucuucugucu || oni-mir-10708 | 10 | 9 | 0 | 1 | - | blast | |  | | --- | | cugcuguuguguaauggguugc | | |  | | --- | | - | | agccuguuacacuacaucauacaauacaccacuguccugcuguuguguaauggguugc || abu-mir-10549 | 41 | 9 | 0 | 32 | - | blast | |  | | --- | | uagguaaucaacaauccaggac | | |  | | --- | | - | | cccuguauugcugauuaccuuguggucuuccuguuagguaaucaacaauccaggaca || oni-mir-10581d | 38 | 8 | 0 | 30 | - | blast | |  | | --- | | aacguccagacgaacagaauc | | |  | | --- | | - | | ucuguucaucuggacguagcguuucucccacugaaaacguccagacgaacagaauc || oni-mir-10605 | 13 | 6 | 0 | 7 | - | blast | |  | | --- | | caaguguaacaggacaaccugu | | |  | | --- | | - | | caaguguaacaggacaaccugucuaaagaguaacagguuguccuguuacucuuu || oni-mir-10606 | 10 | 6 | 0 | 4 | - | blast | |  | | --- | | ugcaaaucagaacgcggcacga | | |  | | --- | | - | | ugcaaaucagaacgcggcacgagcagacaccgcaaaucucuguuuaaucucggcccugugauguauuugcagc || oni-mir-10663 | 7 | 6 | 0 | 1 | - | blast | |  | | --- | | uucagacggucauguagcgacu | | |  | | --- | | - | | uucagacggucauguagcgacucucacgguagaccagccgccugcagauuguucuguugc || dre-mir-737 | 2809 | 2809 | 0 | 0 | - | blast | |  | | --- | | aaucaaaaccuaaagaaaaua | | guuuuuuuagguuuugauuuuu | | |  | | --- | | - | | ccacagcugcugugcuguuguuuuuuuagguuuugauuuuugugaaaugucgaugagaaaaucaaaaccuaaagaaaauacugcgcagauagaugg || oni-mir-10741 | 6 | 6 | 0 | 0 | - | blast | |  | | --- | | agaaagggucguuguccugcu | | |  | | --- | | - | | agaaagggucguuguccugcuugacuuaaaacccugucaggcagguuuuccucccuuuuacagag || oni-mir-10871 | 110 | 5 | 0 | 105 | - | blast | |  | | --- | | aagggccuguauuuguauagcgc | | |  | | --- | | - | | aagggccuguauuuguauagcgcuuuacuuaguuccuaaggaccccaaagcgcuuuacauuauauacagucaucca || abu-mir-551 | 5 | 5 | 0 | 0 | - | blast | |  | | --- | | gcgacccauccuuuguuucug | | |  | | --- | | - | | gaaaccaaguguggguguggucugaaaaagaaauauggcgacccauccuuuguuucug || ssa-mir-33a-1 | 33 | 5 | 0 | 28 | - | blast | |  | | --- | | caaugugucugcagugcagua | | |  | | --- | | - | | gugcauuguaguugcauugcacugggcugcaggaucaugcaaugugucugcagugcagua || oni-mir-10880 | 5 | 5 | 0 | 0 | - | blast | |  | | --- | | uuuuccucugaucugccuccucu | | |  | | --- | | - | | uuuuccucugaucugccuccucucuuucaucaagaaaaccacagagagcagggcagagggagacgaa || oni-mir-9b-5 | 6 | 5 | 0 | 1 | - | blast | |  | | --- | | uuucgguuaucuagcuuuauga | | |  | | --- | | - | | uuucgguuaucuagcuuuaugaugcaugauuaucacucauacagcuagauaaccaaaga || ssa-mir-216b | 5 | 5 | 0 | 0 | - | blast | |  | | --- | | uaaucucagcuggcaacugugag | | |  | | --- | | - | | uaaucucagcuggcaacugugagcaguucaguguucccucucacaguggcuacugggguucug || oni-mir-10934 | 6 | 5 | 0 | 1 | - | blast | |  | | --- | | ccgccgugucagaucuacugga | | |  | | --- | | - | | ccgccgugucagaucuacuggaacuggcucguccagcagauaccgacauggaucgca || oni-mir-10895 | 4 | 4 | 0 | 0 | - | blast | |  | | --- | | auccgggacaugcagcucucc | | |  | | --- | | - | | auccgggacaugcagcucuccggaaacucaaucagcuggccguaugucccaggaca || oni-mir-10573b | 5 | 4 | 0 | 1 | - | blast | |  | | --- | | aacuccaguccucgagggcaug | | |  | | --- | | - | | aacuccaguccucgagggcauggaaaaguugcaugacaccggccuucgaggaauggaguuu || oni-mir-17b-2 | 4 | 4 | 0 | 0 | - | blast | |  | | --- | | accugcacuguaagcacuuuggc | | |  | | --- | | - | | ugaaagugccuucacugcaguagaauugaacaaaacuaccugcacuguaagcacuuuggc || oni-mir-10795 | 11 | 4 | 0 | 7 | - | blast | |  | | --- | | agaagagucacgcugagaggaaaca | | |  | | --- | | - | | cguccucugcugcugcuucuucacacagaucuugugaagaagagucacgcugagaggaaaca || gmo-mir-181b-1 | 10653 | 10653 | 0 | 0 | - | blast | |  | | --- | | aacauucauugcugucgguggguu | | cucacugaacgaugaaugca | | |  | | --- | | - | | aacauucauugcugucgguggguuuggcuaugaagaaaagcucacugaacgaugaaugca || oni-mir-10698 | 4 | 4 | 0 | 0 | - | blast | |  | | --- | | ucaugcauggcuucuuacacu | | |  | | --- | | - | | guaagaaccauaccauucaaugucaaucacacugucaugcauggcuucuuacacu || oni-mir-10696 | 3 | 3 | 0 | 0 | - | blast | |  | | --- | | auggagugaacgucucagugggu | | |  | | --- | | - | | auggagugaacgucucaguggguaucagagcugauuuaagugguaaacaccugcugaacguuuccucuuccaga || oni-mir-10767 | 6 | 3 | 0 | 3 | - | blast | |  | | --- | | aggggaaacuuuuacuuugaag | | |  | | --- | | - | | ucagaguaaaaguuuugccuuacuguugcaaucaacagguuucaaaaggggaaacuuuuacuuugaag || abu-mir-10559 | 3 | 3 | 0 | 0 | - | blast | |  | | --- | | cuucaaagucaacguugcaucc | | |  | | --- | | - | | cuucaaagucaacguugcauccuuggaaacaaguuggcugcaacguugacuuugaacc || oni-mir-10956 | 7 | 3 | 0 | 4 | - | blast | |  | | --- | | uagcuguucucuaacaacuaggccu | | |  | | --- | | - | | uagcuguucucuaacaacuaggccugcuggccauggugucucacaguucag || hhi-mir-182 | 1596 | 3 | 0 | 1593 | - | blast | |  | | --- | | ugguucuagacuugccaacua | | |  | | --- | | - | | cucucuggugguguuuggcaaugguagaacucacacuggugagguagauggauccggugguucuagacuugccaacuacugccugagagcgucgac || abu-mir-10564 | 14 | 3 | 0 | 11 | - | blast | |  | | --- | | aacacaacgcgaagaaauaugc | | |  | | --- | | - | | aacacaacgcgaagaaauaugcacuacuaagugcauauuucuucgcguuguguuuu || oni-mir-10938 | 3 | 3 | 0 | 0 | - | blast | |  | | --- | | ucuguuugugucuguuuucaga | | |  | | --- | | - | | ucuguuugugucuguuuucagauaugagucuggauagcagacucauaucugugagcauuuacaaccaccuuugaua || oni-mir-10739 | 3 | 3 | 0 | 0 | - | blast | |  | | --- | | uuagaucaauaaguauggaaugu | | |  | | --- | | - | | uuagaucaauaaguauggaauguguuccuaacagacauauuucuugaucuucuag || oni-mir-10913 | 3 | 3 | 0 | 0 | - | blast | |  | | --- | | uagcccgggcugcucugcuga | | |  | | --- | | - | | agcuguagcugugggccggggggugagcuaccuagcccgggcugcucugcuga || oni-mir-10620 | 3 | 2 | 0 | 1 | - | blast | |  | | --- | | gaaauaaagaauucucucgaagug | | |  | | --- | | - | | gaaauaaagaauucucucgaaguguuacuucuccaauaaucacucgaagaauuccuuguuuuag || oni-mir-10865 | 4 | 2 | 0 | 2 | - | blast | |  | | --- | | uucggauguagcucugccgcugcuc | | |  | | --- | | - | | uucggauguagcucugccgcugcucuaggugacucagcacagcguggagggcuguggcgaugg || oni-mir-10548c | 2 | 2 | 0 | 0 | - | blast | |  | | --- | | acggcaccgcagaccacgccc | | |  | | --- | | - | | cguggucuguggagccgugcagguuuuguccgcccucccugcacggcaccgcagaccacgccc || oni-mir-4585 | 2 | 2 | 0 | 0 | - | blast | |  | | --- | | aaugcguuucggcuuguggccu | | |  | | --- | | - | | aaugcguuucggcuuguggccuucauuggacccugaugaaggccacaagccaaaacgcguugg || oni-mir-26d | 2 | 2 | 0 | 0 | - | blast | |  | | --- | | ccuauccuggauuacuugaacc | | |  | | --- | | - | | gcaaguaaucaagcauaggccaaagcagaugcagaaagccuauccuggauuacuugaacc || tni-mir-219-1 | 8 | 2 | 0 | 6 | - | blast | |  | | --- | | ugauuguccaaacgcaauucuugu | | |  | | --- | | - | | ucugagcacuagcagcugauuguccaaacgcaauucuugugaaaauccaaagccuacccgagaauugugccuggacaucuguugcuggacgcucc || oni-mir-10702 | 2 | 2 | 0 | 0 | - | blast | |  | | --- | | ugaugaaggagauagcacugugc | | |  | | --- | | - | | cagugcuaucuccuucaucacacugggugugaugaaggagauagcacugugc || abu-mir-3120 | 2 | 2 | 0 | 0 | - | blast | |  | | --- | | cugucugugccugcuguacagg | | ugcacagcaaguguagauaggc | | |  | | --- | | - | | cugucugugccugcuguacagguuggaggauauucugcacagcaaguguagauaggc || ssa-mir-551 | 2 | 2 | 0 | 0 | - | blast | |  | | --- | | gaaaccaaguguggguguggccu | | |  | | --- | | - | | gaaaccaaguguggguguggccuggaaugacaauauggcgacccauccuugguuucug || oni-mir-430b | 4 | 2 | 0 | 2 | - | blast | |  | | --- | | ucaagugcuaauuguuguugua | | |  | | --- | | - | | acagcaacaugagcacuuacgugaauucugaaaacaucaagugcuaauuguuguugua || ipu-mir-3618 | 2 | 2 | 0 | 0 | - | blast | |  | | --- | | gauuuccaauaauugagacagu | | |  | | --- | | - | | gauuuccaauaauugagacaguaauuucuaaaaagcugucuacauuaaugaaaagaac || oni-mir-10690 | 3 | 2 | 0 | 1 | - | blast | |  | | --- | | cgugacuugaguaugggcaugca | | |  | | --- | | - | | cgugacuugaguaugggcaugcaguuuggaaagaaaaugcauccacaucucaagcuauuga || oni-mir-10890 | 2 | 2 | 0 | 0 | - | blast | |  | | --- | | ugcacucugcacacccacuucu | | |  | | --- | | - | | agguggauguguggugggguuaaaggaaucugauuuugagauuguggucaugcuccugcacucugcacacccacuucu || abu-mir-10548 | 2 | 2 | 0 | 0 | - | blast | |  | | --- | | cacggcaccgcaggccccgcc | | cguggccugcggugccgugc | | |  | | --- | | - | | cguggccugcggugccgugcaggugcguccgccuccucugcacggcaccgcaggccccgccc || oni-mir-10752 | 4 | 2 | 0 | 2 | - | blast | |  | | --- | | caggacacagaaucgcuggugg | | |  | | --- | | - | | caggacacagaaucgcugguggcugacuuuacaccccccggcgugccuguccacugucu || oni-mir-10697b-1 | 11 | 2 | 0 | 9 | - | blast | |  | | --- | | ggcggccucagaaucucaucu | | |  | | --- | | - | | ggcggccucagaaucucaucucugcuuuucuuagaugaugugguucuguuggcuuuagc || oni-mir-184c | 2 | 2 | 0 | 0 | - | blast | |  | | --- | | ugggcuggaaaagugauaaggag | | |  | | --- | | - | | ccuuaucaguucuccguccaacggauacagaaucuauagcugggcuggaaaagugauaaggag || oni-mir-10697a-1 | 2 | 1 | 0 | 1 | - | blast | |  | | --- | | augggguucuguuggcuucauc | | |  | | --- | | - | | gguggccucagaaucucaugucaucuuuucacagaugaugggguucuguuggcuucauc || oni-mir-10651 | 1 | 1 | 0 | 0 | - | blast | |  | | --- | | ugagcagggcuguguuuuugagc | | |  | | --- | | - | | uuaaaaacaggcccuggugacacaggagcugcggugcagcggugugagcagggcuguguuuuugagc || oni-mir-726b | 2 | 1 | 0 | 1 | - | blast | |  | | --- | | ugaguucugcuaguagugaacu | | |  | | --- | | - | | ugaguucugcuaguagugaacuuuugacaaacacgaauaguucagaacuagcggaauuccaga || oni-mir-10650 | 2 | 1 | 0 | 1 | - | blast | |  | | --- | | accccaugaugaaucucugaag | | |  | | --- | | - | | accccaugaugaaucucugaaggugaaucuccaucgagaggcuaauaucaaguucacuguggacgugcacc || oni-mir-10863 | 2 | 1 | 0 | 1 | - | blast | |  | | --- | | ugacuucuuugugggcaggcac | | |  | | --- | | - | | ugacuucuuugugggcaggcaccaccaugcaugagccugcccacgcaguagucaug || oni-mir-10632-1 | 1 | 1 | 0 | 0 | - | blast | |  | | --- | | ucacuggacugagaggaguaucu | | |  | | --- | | - | | ucacuggacugagaggaguaucugagaagcugacgcucuccucuuugucuccggugaag || oni-mir-10939-1 | 1 | 1 | 0 | 0 | - | blast | |  | | --- | | uauguggacauaaggcuuaccc | | |  | | --- | | - | | uauguggacauaaggcuuacccaucuuuaccagcacaauggggaggccuaugugcacucgug || oni-mir-10953 | 3 | 1 | 0 | 2 | - | blast | |  | | --- | | aucacuucaggggacugaacc | | |  | | --- | | - | | aucacuucaggggacugaaccacugacaauuuguggcucauucuguugaugugcuuu || oni-mir-10703 | 1 | 1 | 0 | 0 | - | blast | |  | | --- | | ucucuaccggcgcacgucgcugu | | |  | | --- | | - | | gcgacugcgugcggcagagugguuccgaaacaccgacuucucuaccggcgcacgucgcugu || oni-mir-10659 | 9 | 1 | 0 | 8 | - | blast | |  | | --- | | agaggcguccuggagagguaga | | |  | | --- | | - | | agaggcguccuggagagguagagccugcgcgucucuccucucuccaaggcgucucuuc || abu-mir-10550 | 1 | 1 | 0 | 0 | - | blast | |  | | --- | | cagcacaucgcugaacugaagcc | | cucaguuuagaagugugccucg | | |  | | --- | | - | | cagcacaucgcugaacugaagccgugauuucagugggcucaguuuagaagugugccucg || oni-mir-10771 | 1 | 1 | 0 | 0 | - | blast | |  | | --- | | cuguuguucugaguggcuguga | | |  | | --- | | - | | ccugucacuuuaacaauauguuugauaauuacccuuuuacuguuguucugaguggcuguga || oni-mir-132c | 109 | 1 | 0 | 108 | - | blast | |  | | --- | | agccaugacuguagacuguuacu | | |  | | --- | | - | | agccaugacuguagacuguuacuguacuuugauacuguauaagcaguaagcagucuaaagccaaggugc || oni-mir-10751 | 1 | 1 | 0 | 0 | - | blast | |  | | --- | | aagugcacgacuccagacuagu | | |  | | --- | | - | | agaguucgguguugugcgcucacgcucaaaaccuccacuggaagugcacgacuccagacuagu || oni-mir-10573c | 90 | 1 | 0 | 89 | - | blast | |  | | --- | | aacuccaguccucgaaggccgg | | |  | | --- | | - | | aacuccaguccucgaaggccggugucaugcaucuuuuccaugcccucgaggacuggcguuugaga || oni-mir-10649 | 2 | 1 | 0 | 1 | - | blast | |  | | --- | | uuuuaggucaggagaugcacacg | | |  | | --- | | - | | uguucgucuccuccugaaggcccgcaacacuacuuuuaggucaggagaugcacacg || oni-mir-10856 | 1 | 1 | 0 | 0 | - | blast | |  | | --- | | cugauccaggaccaucucagug | | |  | | --- | | - | | cugauccaggaccaucucagugcugcugcugguuguugugguuuuacugggaugguuuuggaucaggu || fru-mir-23a-2 | 6339 | 1 | 0 | 6338 | - | blast | |  | | --- | | ggauuccuggcagagugauuug | | |  | | --- | | - | | agcuggagggauuccuggcagagugauuuggcugugauguaauguaaaucacauugccagggauuuccaaccagcug || ccr-mir-124a | 1 | 1 | 0 | 0 | - | blast | |  | | --- | | ucaagguccgcugugaacacg | | |  | | --- | | - | | uuucuuggcauucaccgcgugccuuaauuguauggaaauuuaaaucaagguccgcugugaacacgaagaaaa || ccr-mir-132b | 1 | 1 | 0 | 0 | - | blast | |  | | --- | | accauggcuguagacuguuacc | | |  | | --- | | - | | gaaguuucugucccauagcgaccauggcuguagacuguuaccagaaguacuguugcuacaguaacaaucuaaugccacggucgccauggagacagcaagag || oni-mir-10778 | 1 | 1 | 0 | 0 | - | blast | |  | | --- | | caacugcagggcauuggacgagc | | |  | | --- | | - | | cccucuaaagcccugcagcucaggguccuuucccaacugcagggcauuggacgagc || oni-mir-10723 | 1 | 1 | 0 | 0 | - | blast | |  | | --- | | aggugacaugugcuuaggacaggu | | |  | | --- | | - | | cuguucugagucaaguccccuuguaugaacacaaacaaggugacaugugcuuaggacaggu || oni-mir-10860 | 1 | 1 | 0 | 0 | - | blast | |  | | --- | | ugggugugggcucugcucucuacc | | |  | | --- | | - | | cuggagaguugagcccaagcaggcagcucugagguagcugggugugggcucugcucucuacc || oni-mir-27f | 1 | 1 | 0 | 0 | - | blast | |  | | --- | | uucacauguggguuaaguccug | | |  | | --- | | - | | agaacuuaaccacugugaacauggcagacacucacuguucacauguggguuaaguccug || oni-mir-10800 | 1 | 1 | 0 | 0 | - | blast | |  | | --- | | aaggugacaguuguuguggaca | | |  | | --- | | - | | aaggugacaguuguuguggacaccuaccuuaaccccuuggugguucucacuccgguggccaucgcuaacugucaccaaga || abu-mir-10543 | 2 | 1 | 0 | 1 | - | blast | |  | | --- | | auuggcucuguagaggacugga | | |  | | --- | | - | | uuuguccucgcuuugaucuagugauuauuaccaguucauuggcucuguagaggacugga || oni-mir-10900b | 3 | 1 | 0 | 2 | - | blast | |  | | --- | | uugaucuggagguaguucugcu | | |  | | --- | | - | | uguggaacucuaccaccagcuccuugguuuuauugguguugaucuggagguaguucugcu || oni-mir-10946 | 1 | 0 | 0 | 1 | - | blast | |  | | --- | | acugaauuuggcaggcugauccu | | |  | | --- | | - | | gugccugcuggauuuaaucaaauaacacuaaacauugacugaauuuggcaggcugauccu || fru-mir-219-2 | 2 | 0 | 0 | 2 | - | blast | |  | | --- | | ggaguuguggauggacaucacgc | | |  | | --- | | - | | ucggagcugauuguccaaacgcaauucuugcgucugccuuugugaaaccaggaguuguggauggacaucacgccccugac || oni-mir-10857 | 1 | 0 | 0 | 1 | - | blast | |  | | --- | | cugcacugacgagcuuucaccc | | |  | | --- | | - | | augacggcuugaccgugcagcuucaaaacacugaagcugcacugacgagcuuucaccc || oni-mir-10816 | 1 | 0 | 0 | 1 | - | blast | |  | | --- | | uuaauguccaucugucacugu | | |  | | --- | | - | | ccguugacagagugaagacgcuaauuaaaacacugaaaauuaauguccaucugucacugu || oni-mir-10786 | 1 | 0 | 0 | 1 | - | blast | |  | | --- | | uaguccacgagcagucugugac | | |  | | --- | | - | | uaguccacgagcagucugugacugcuugcacugagucacagucuguucguggacagc || oni-mir-10876 | 1 | 0 | 0 | 1 | - | blast | |  | | --- | | cacaggcuguaauuccacugag | | |  | | --- | | - | | guggagcgacggccuguugcugcccguggauccugagugaagcacaggcuguaauuccacugag || oni-mir-26c | 1 | 0 | 0 | 1 | - | blast | |  | | --- | | gcaaguaaucacgaauaggcc | | |  | | --- | | - | | gcaaguaaucacgaauaggccauccaggauacacacagccuauccuggauuacuugaacu || dre-mir-124-5 | 53 | 53 | 0 | 0 | - | blast | |  | | --- | | cguguucacagcggaccuugau | | uaaggcacgcggugaaugccaa | | |  | | --- | | - | | ggguuuugcucgugcguucuuuuugaguucucgcucugcguguucacagcggaccuugauuuaauguccauacaauuaaggcacgcggugaaugccaagagaagaaucucuccagcaacgaguuugcgc || oni-mir-10808 | 1 | 0 | 0 | 1 | - | blast | |  | | --- | | acggaaagccgugcggccgggcu | | |  | | --- | | - | | cggcccgccgggucgccaucccggugcuggugcgggacggaaagccgugcggccgggcu || oni-mir-10927 | 2 | 0 | 0 | 2 | - | blast | |  | | --- | | aucaucaacucugucacguga | | |  | | --- | | - | | acgguacacagagcugguguuuguuguaguuuaaaacagcaucaucaacucugucacguga || oni-mir-10910 | 1 | 0 | 0 | 1 | - | blast | |  | | --- | | accuccuccacugugggaaccu | | |  | | --- | | - | | accuccuccacugugggaaccuuguucccugcagccugcaaggcucuccacagggcagagucu || ccr-mir-218a | 897 | 0 | 0 | 897 | - | blast | |  | | --- | | acaugguuccgucaagcaccagg | | |  | | --- | | - | | ggugcagcugucucuugugcuugaucuaaccaugugccgccgccuacacaagccucacaugguuccgucaagcaccagggaccgcuggac || oni-mir-10673 | 1 | 0 | 0 | 1 | - | blast | |  | | --- | | uuagaccagaugauaccagcccu | | |  | | --- | | - | | accgguauuguuggccuggguccugcuaagacacacgcuuagaccagaugauaccagcccu || oni-mir-10787 | 1 | 0 | 0 | 1 | - | blast | |  | | --- | | cucccacacuuacuuagcaccu | | |  | | --- | | - | | cucccacacuuacuuagcaccugugcagcauuacuaaaagggugcagugggugugggagug || oni-mir-10581c | 8 | 0 | 0 | 8 | - | blast | |  | | --- | | aacguccagaugaacaaucaac | | |  | | --- | | - | | ugauuguucaucuggacguaacguuuucagugggagaaacguccagaugaacaaucaac || oni-mir-144b | 1 | 0 | 0 | 1 | - | blast | |  | | --- | | uuacaguauaagaugauauccug | | |  | | --- | | - | | aguacaucaucuauacuguagugucucuuuaauaaacuuacaguauaagaugauauccug || oni-mir-10961 | 3 | 0 | 0 | 3 | - | blast | |  | | --- | | caccuuuaaguagaaauugacc | | |  | | --- | | - | | ucuuugucuacuucagguguuuuuguuuuucuuuuagcaccuuuaaguagaaauugacc || oni-mir-204b | 1 | 0 | 0 | 1 | - | blast | |  | | --- | | cauaggaugacaaagggaagcc | | |  | | --- | | - | | aucccuuugcugucccugccuuauccaaguuccaggcauaggaugacaaagggaagcc || oni-mir-10768 | 74 | 0 | 0 | 74 | - | blast | |  | | --- | | ucacacagguuggcgaagguuu | | |  | | --- | | - | | accuuugcccaccugucugagagugauugcagugagucucacacagguuggcgaagguuu || oni-mir-10674 | 2 | 0 | 0 | 2 | - | blast | |  | | --- | | aauagccgccuugagaccagga | | |  | | --- | | - | | aauagccgccuugagaccaggagggcaagguucgaaaccugcucagggcggcauucuuuu || oni-mir-10684 | 4 | 0 | 0 | 4 | - | blast | |  | | --- | | accauugaucaacaaccacugau | | |  | | --- | | - | | cagugagcauugaucaauggccaugcaaauucucauuaccauugaucaacaaccacugau || ssa-mir-190b | 5 | 0 | 0 | 5 | - | blast | |  | | --- | | acuaaauaucagacauauuccua | | |  | | --- | | - | | ugauauguuugauauucgguuguucuccugcguaaucaagucaacuaaauaucagacauauuccua || oni-mir-10633 | 1 | 0 | 0 | 1 | - | blast | |  | | --- | | uggcauugugggauagaagcuc | | |  | | --- | | - | | uggcauugugggauagaagcucaguaucagcugguucugugacugucucucucuaucagcuacagugccug || oni-mir-10647 | 1 | 0 | 0 | 1 | - | blast | |  | | --- | | uuguuguugcugcugugaugcu | | |  | | --- | | - | | uuguuguugcugcugugaugcugaggacagacacagcaucacaguacuggaacagagcu || fru-mir-10b-2 | 180763 | 0 | 0 | 180763 | - | blast | |  | | --- | | caaguacgucucuacaggaaua | | |  | | --- | | - | | guugucuauauguacccuguagaaccgaauuugugugaguuccagacagucgcaaguacgucucuacaggaauacaugggcaac || oni-mir-10757 | 1 | 0 | 0 | 1 | - | blast | |  | | --- | | cuugucagaacucccacucaug | | |  | | --- | | - | | cuugucagaacucccacucauguaaacuucuucuucaugacgugguagcuuugcucagc || oni-mir-10970 | 1 | 0 | 0 | 1 | - | blast | |  | | --- | | uuccuggaaucauuucgacuc | | |  | | --- | | - | | gucgaaaugauuccaggaacagaaugaaguccaacuucauucuguuccuggaaucauuucgacuc || oni-mir-10875 | 1 | 0 | 0 | 1 | - | blast | |  | | --- | | auguacccccuuguuccugac | | |  | | --- | | - | | auguacccccuuguuccugacaagguuuuugucagucuggaacaaggguguauauca |
