# Supplementary material for: Differential expression of miRNAs in the presence of B chromosome in the cichlid fish Astatotilapia latifasciata
Source: BMC Genomics. 2021 May 12;22:344. doi: 10.1186/s12864-021-07651-w (PMC8117508; doi:10.1186/s12864-021-07651-w)
Supplement: Supplementary file 11 — Additional file 11. Samples and primers information. Table S1. RNA-seq quality and filtering. Table S2. Normalized reads mapped in the genome. Table S3. miRBase fish database entries used to create the reference miRNA list. Table S4. Primers used for genomic PCR, genomic qPCR and RT-qPCR. [file 12864_2021_7651_MOESM11_ESM.pdf]

Table S1 RNAseq quality and filtering. Br- brain, Go- gonads, Mu- muscle; FB- female B+, Fb- female without B-, MB- male B+, Mb- male, B-. The numbers meaning the biological replicates.

RNAseq sample	Raw reads	Filtered reads	Filtered reads (%)
Br-FB-1	13,950,507	12,899,609	92.47
Br-FB-2	18,245,415	8,483,048	46.49
Br-FB-3	16,470,250	12,186,072	73.99
Br-Fb-1	16,038,123	11,730,340	73.14
Br-Fb-2	14,597,485	13,479,974	92.34
Br-Fb-3	19,878,364	11,544,813	58.08
Br-MB-1	15,599,135	11,757,289	75.37
Br-MB-2	14,950,748	9,883,886	66.11
Br-MB-3	18,418,396	13,734,438	74.57
Br-Mb-1	13,218,667	10,767,397	81.46
Br-Mb-2	13,396,271	11,236,968	83.88
Br-Mb-3	14,462,144	11,213,256	77.54
Go-FB-1	20,357,731	9,767,262	47.98
Go-FB-2	24,067,280	13,157,076	54.67
Go-FB-3	13,883,524	8,265,707	59.54
Go-Fb-1	13,684,230	7,858,365	57.43
Go-Fb-2	14,017,168	8,077,051	57.62
Go-Fb-3	19,560,926	11,616,614	59.39
Go-MB-1	15,985,926	6,640,759	41.54
Go-MB-2	16,173,228	6,600,334	40.81
Go-MB-3	24,888,369	9,741,359	39.14
Go-Mb-1	15,415,060	6,766,093	43.89
Go-Mb-2	19,567,332	7,383,530	37.73
Go-Mb-3	15,972,791	7,121,332	44.58
Mu-FB-1	23,788,846	9,221,077	38.76
Mu-FB-2	18,143,462	10,736,418	59.18
Mu-Fb-1	25,030,699	14,799,691	59.13
Mu-Fb-2	16,617,575	13,410,630	80.70
Mu-MB-1	20,630,448	11,569,042	56.08

Mu-MB-2	12,479,370	7,798,086	62.49
Mu-Mb-1	16,459,529	9,930,843	60.33
Mu-Mb-2	23,882,108	14,441,392	60.47

Table S2 Normalized reads mapped in the genome. Br- brain, Go- gonads, Mu- muscle; FB- female B+, Fb- female without B-, MB- male B+, Mb- male, B-

Sample	Total reads	Mapped reads	Mapped reads (%)
Br-Fb	26,342,977	23,205,950	88.09
Br-FB	25,399,499	22,663,007	89.23
Br-MB	24,911,453	21,800,249	87.51
Br-Mb	25,228,301	22,977,679	91.08
Go-FB	26,159,086	7,242,201	27.69
Go-Fb	23,217,731	6,435,588	27.72
Go-MB	19,020,567	4,457,475	23.44
Go-Mb	17,684,510	3,682,046	20.82
Mu-FB	15,590,884	11,077,022	71.05
Mu-Fb	22,503,951	19,735,616	87.70
Mu-MB	14,596,696	10,241,158	70.16
Mu-Mb	18,466,577	12,744,712	69.02

Table S3 miRbase fish database entries used to create the reference miRNA list.

Species	number of precursors	number of matures
Astatotilapia burtoni	298	236
Cyprinus carpio	134	146
Danio rerio	355	373
Electrophorus electricus	20	34
Fugu rubripes	131	108
Gadus morhua	401	516
Hippoglossus hippoglossus	39	36
Ictalurus punctatus	281	205
Metriaclima zebra	256	184
Neolamprologus brichardi	251	182
Oryzias latipes	168	146
Oreochromis niloticus	812	695
Pundamilia nyererei	250	182
Paralichthys olivaceus	20	38
Salmo salar	371	497
Tetraodon nigroviridis	132	109

Table S4 Primers used to genomic PCR, genomic qPCR and RT-qPCR

Name	sequence
1B+read-novel-Fw	ATGACCGTTAATATCTGGGTG
1B+read-novel-Rv	TCCTAGTGGCCGAAAATGG
2B+read-novel-Fw	GCTCCCTTTGATAGCTCAGC
2B+read-novel-Rv	CAGCTTGAAGGGAGATGTTAGTG
3B+read-novel-Fw	GTATCGCTTCTCGGCCTTTT
3B+read-novel-Rv	CAATACCAGGTCGATGCGT
4B+read-novel-Fw	GGGAGCAATGTCACTTTTGTCT
4B+read-novel-Rv	ATTTTTATGAGCCGCAGCAG
5b-read-novel-Bbl-Fw	ATCAGGTGGGTGGTTGCTTTA
5b-read-novel-Bbl-Rv	ATGGCTCATCAGGACTACATGC
6b-read-novel-Bbl-Fw	CCAATCCCTCCTTTAGGTTCAG
6b-read-novel-Bbl-Rv	TTCCTGTCAGGCCCCAAA
7b-read-novel-ctrl-Fw	AGATGTGAAGTACTCTGACTGCGA
7b-read-novel-ctrl-Rv	CTTGAAATGTAGCCTGGGTATAGC
8b-B+read-novel-Fw	AAGGTCTTCTCACCCAGTCTCC
8b-B+read-novel-Rv	CAGCAACCTCGGTGGAAAC
9b-B+read-novel-Fw	CCTCCTTGCTGCTGTGTTG
9b-B+read-novel-Rv	AAGTGAGGACGGCTGCTTG
10b-B+read-novel-Fw	ATCAGAAGGTCGGTGGTTCG
10b-B+read-novel-Rv	ACAACCTAAGGACCCCAAAGC
HPRT-Fw-gen-ctrl	TCTGTGTGCTGAAAGGGGGC
HPRT-Rv-gen-ctrl	GCTCTCTGTGTGCTGAAAGG
Dicer_F	CCTGGACAGATACGCCTCTC
Dicer_R	GTCATTGAGGACCTGTTTGGAG
Drosha_F	GACCAAGTGGGAGACTCTACAG
Drosha_R	CTGAGTCGCTGCTGTAACCTG
UBCE_Fw	GTCCGTTTCAATCCCAACTT
UBCE_Rv	GTTCTCCGTCATCAGAGACT
